# A Randomized Clinical Trial of Kidney Autologous Cell Therapy in Diabetic Kidney Disease

**DOI:** 10.2215/CJN.0000000969

**Published:** 2026-01-02

**Authors:** Borut Čižman, Emily L. Butler, Joseph Stavas, Rachita Prakash, Theodore Saad, Arnold Silva, Thomas Wooldridge, Ahmed Aqeel, Hongxia Yan, Constance M. Barysauskas, Bruce Culleton

**Affiliations:** 1 ProKidney, Winston-Salem, North Carolina; 2 Kidney Associates of Colorado, PC, Englewood, Colorado; 3 Nephrology Associates—Delaware Kidney, Newark, Delaware; 4 Boise Kidney and Hypertension Institute, Boise, Idaho; 5 Nephrology and Hypertension Assoc, Ltd, Tupelo, Mississippi; 6 Paragon Health Nephrology Center, Kalamazoo, Michigan

**Keywords:** CKD, clinical trial, diabetes mellitus

## Abstract

**Key Points:**

- Bilateral kidney injections with rilparencel was associated with a 78% improvement in the annual eGFR decline in kidney function.
- There were no serious adverse events related to rilparencel therapy.
- The interventional procedures were well tolerated and had an acceptable safety profile.

**Background:**

Despite new treatments to delay disease progression in patients with diabetes and CKD, many patients continue to lose kidney function and progress to kidney failure. Additional therapeutic strategies, perhaps targeting multiple deleterious pathways, are necessary to preserve kidney function in patients with advanced CKD.

**Methods:**

A multicenter, randomized, phase 2 clinical trial (NCT05018416) assessed rilparencel (an autologous cell therapy composed of cells obtained by kidney biopsy), in participants with diabetes and estimated glomerular filtration rate (eGFR) 20–50 ml/min per 1.73 m^2^. Participants were randomized 1:1 to two cohorts. Cohort 1 received two rilparencel injections percutaneously into the kidney cortex, 3 months apart, one in each kidney. Cohort 2 received one injection and a second injection only upon a sustained decline in eGFR or increase in urinary albumin creatinine ratio. Participants were followed up to 18 months after their last injection. The primary efficacy end point was change in eGFR slope from the preinjection period to the period after the last injection. The primary safety end point was the percentage of participants with procedure or rilparencel-related treatment emergent adverse events (TEAEs).

**Results:**

Fifty-three participants were randomized and 49 received at least one injection. In Cohort 1, annual eGFR slope (ml/min per 1.73 m^2^/year) in the preinjection period was -5.84 (SEM 1.07) versus −1.27 (SEM 1.36) in the period after the last injection (difference 4.57 [95% confidence interval, 1.95 to 7.18]). In Cohort 2, annual eGFR slope in the pre-injection period was −3.40 (SEM 0.81) versus −1.71 (SEM 1.04) in the period after the last injection (difference 1.70 [95% confidence interval, −0.24 to 3.63]). No interactions were observed across multiple predefined baseline subgroups. Of 87 injections, procedure-related TEAEs occurred in 16 participants and rilparencel-related TEAEs occurred in six participants. No product-related serious adverse events and no procedure-related or product-related deaths were reported.

**Conclusions:**

Bilateral kidney injection of rilparencel may preserve kidney function with an acceptable safety profile. A multicenter, phase 3, randomized, sham-controlled study is ongoing.

## Visual Abstract

**Figure:**
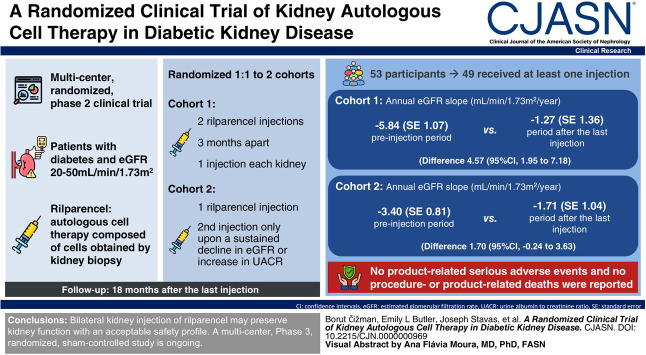

## Introduction

Progression of CKD is a major area of clinical investigation, including several recent phase 3 clinical trials in participants with diabetic kidney disease. Multiple new drugs have been approved in the United States and worldwide adding to the well-established renin-angiotensin system blockers standard of care in CKD. These new medications include sodium-glucose cotransporter-2 inhibitors, glucagon-like peptide-1 receptor agonists (GLP-1 RA), and nonsteroidal mineralocorticoid receptor antagonists. Positive results from these landmark clinical trials have changed the practice of kidney medicine, establishing now four pillars of treatment options used in a step-wise approach.^1^ Despite this tremendous success, a substantial residual risk of progressive CKD remains with many treated patients progressing to ESKD. Additional therapeutic strategies, perhaps targeting multiple deleterious pathways, may be necessary to halt or preserve kidney function in patients with advanced CKD.

Among the many novel therapeutic approaches, cellular therapies have emerged as an investigational option because of the potential to address multiple maladaptive processes simultaneously.^2^ In this phase 2 study, we hypothesized that among participants with diabetes mellitus and advanced CKD, treatment with rilparencel, an autologous cellular therapy composed of kidney cells obtained from a kidney biopsy, would be safe and well tolerated, and would preserve kidney function.

## Methods

### Study Population

REGEN-007 (registered at ClinicalTrials.gov, NCT05018416, Aug 17, 2021) was a phase 2, multicenter, randomized, open-label trial of rilparencel in persons with diabetes mellitus and CKD; local Institutional Review Boards and the United States Food and Drug Administration approved the protocol Supplemental Material Clinical Protocol, which adhered to the principles of the Declaration of Helsinki. Written informed consent was obtained from all participants. Eligible participants were age 30–80 years, with type 1 or type 2 diabetes mellitus, hemoglobin >10 g/dl, glycated hemoglobin (HbA1c) <10%, eGFR 20–50 ml/min per 1.73 m^2^, and urine albumin to creatinine ratio (UACR) 30–5000 mg/g. Key exclusion criteria were history of kidney transplant, recent AKI, known bleeding disorder, and participants otherwise judged by the interventional radiologist to be a poor candidate for kidney biopsy or rilparencel injection.

### Treatment Arms

**Figure 1:**
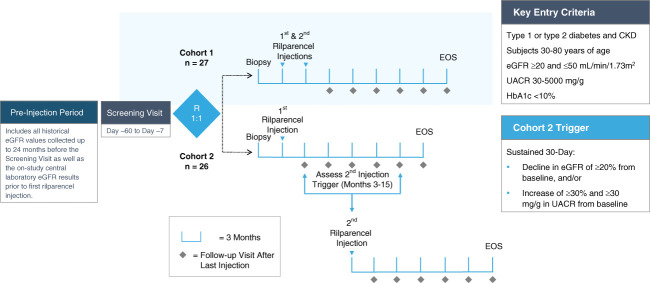
**Schematic of study design and key inclusion criteria.** Eligible participants were randomized to two treatment Cohorts. In Cohort 1, participants received two scheduled rilparencel injections with the second injection in the contralateral kidney 3 months after the first injection. In Cohort 2, a second injection was only administered if a participant experienced a sustained decline in eGFR or a sustained increase in UACR. EOS, end of study visit, HbA1c, glycated hemoglobin; R, randomization; UACR, urine-albumin/creatinine ratio.

Eligible participants were randomized in a 1:1 ratio to one of two cohorts using the Randomization Module by Interactive Response Technology. The randomization sequence was created using SAS 9.4 statistical software (SAS Institute Inc, Cary, NC) with a block size of 2. Cohort 1 received two rilparencel injections, 3 months apart, one in each kidney. Cohort 2 received one injection and a second only if there was a sustained eGFR decline of ≥20% and/or increase in UACR ≥30% and ≥30 mg/g within 3–15 months after the first injection. If these criteria were not met, participants in Cohort 2 completed their participation with one injection; if criteria were met, a second dose was given within 30 days of meeting the criteria (Figure 1).

Kidney cells were obtained through a standard percutaneous kidney biopsy using image-guided ultrasound or computerized tomography. After transport to ProKidney's manufacturing facility, enzymatic digestion of the biopsy was followed by cellular *ex-vivo* expansion using standard cell culture techniques. Epithelial kidney cells were selected by density-gradient centrifugation and formulated to produce rilparencel, as previously reported.^3^ The dose of rilparencel was 3×10^6^ cells/g of estimated kidney weight, determined by magnetic resonance imaging. The concentration of cells in rilparencel is 100×10^6^ cells/ml ±20%; the dosing volume was 3.0 ml for each 100 g of kidney weight. Rilparencel was injected percutaneously into the kidney cortex with computerized tomography image guidance. All the procedures were performed in an outpatient setting, as described elsewhere.^3^

### Study Procedures

Participants were followed every 3 months until 18 months after the last injection (the last injection was defined as the second injection if a participant received two injections and as the first injection if a participant received one injection). To expedite data analysis and reporting, the study was closed on December 17th, 2024. By that date, all injections were near complete in both cohorts, and the closure was timed such that all participants would have at least 6 months' follow-up after their last injection for safety and efficacy evaluations. All participants were offered to roll over into a long-term follow-up study and 34 of 49 treated participants elected to rollover.

### Outcome Measurements, Safety Assessments, and Statistical Analysis

All randomized participants who received at least one rilparencel injection made up the modified intent-to-treat and the safety set populations. Efficacy analyses were performed on the modified intent-to-treat population and safety analyses on the safety set (Statistical Analysis Plan in Supplemental Materials). The primary efficacy end point (change from slope of eGFR in the preinjection period to the slope in the period after the last injection) was analyzed using a linear mixed model. The preinjection period included all historical eGFR results from ≤24 months before screening up to the date of first injection, with the last result prior to first injection as baseline. The median number of historical eGFR data points per participant was four (interquartile range, 3–7). These historical data points spanned a median of 19 months before screening (interquartile range, 16–21 months). The period after the last injection (termed post-last injection period in Supplement) included the visits from the date of last injection to end of study visit, with baseline defined as the average of eGFR measurements taken within 21 days before the last injection, excluding postprocedure measurements on date of biopsy or injection. The median number of eGFR data points after last injection per participant was seven (interquartile range, of 7–9). These data points spanned a median of 18 months after last injection (interquartile range, of 12–18 months). An unstructured covariance matrix was used for the model, which included eGFR change from baseline as the dependent variable, time, study period, and interaction between time and study period as fixed effects and baseline eGFR as covariate. To account for between-participant variability in eGFR trajectories, time and intercepts were included as random effects. For each study period, the estimate of eGFR slope (95% confidence interval [CI]) was reported. The slope difference between the two study periods was reported as the coefficient of the interaction term in the model, including 95% CI. No statistical significance threshold was prespecified.

A secondary end point of slope of eGFR using the period after the first injection, rather than the period after the last injection, was also performed. Other prespecified secondary end points included time from first injection to the following: (*1*) ≥40% reduction in eGFR, sustained for 30 days, (*2*) eGFR <15 ml/min per 1.73 m^2^, sustained for 30 days, (*3*) ≥30% and 30 mg/g increase in UACR sustained for 90 days, and (*4*) kidney or cardiovascular death. Secondary end points also included three-component and four-component composite end points made up of components 1, 2, and 4 above or all components, respectively. In addition, summary of the percentage of participants who had the same or reduced 5-year and 2-year risk of ESKD per the eight-variable Kidney Failure Risk Equation^4^ at 12 and 18 months after the first injection was a prespecified secondary end point, including changes from baseline by visit. Participants who had missing eGFR measurements at 12-month and 18-month time points after the first injection were counted as having increased risk of ESKD.

If at least half the participants experienced an event, Kaplan–Meier methodology was used to estimate median time to event, including 95% CI and lower and upper quartiles, for the relevant secondary end points. Otherwise, the probability of not experiencing the events at landmark time points was estimated with corresponding 2-sided 95% CIs.

A *post hoc* analysis of change from baseline in eGFR after the last injection was performed for this publication. For all efficacy analyses, eGFR measurements taken after a kidney transplant, the initiation of dialysis, or post procedure on the dates of biopsy and injection were removed. Prespecified subgroup analyses of the primary end point were conducted for: the number of rilparencel injections received, diabetes type, and by the following baseline variables: CKD Stage (G3a, G3b, or G4), body mass index, HbA1c values, GLP-1 RA use, nonsteroidal mineralocorticoid receptor antagonists or MRA use, and albuminuria category (A2 or A3). A *post hoc* analysis of sodium glucose co-transporter 2 inhibitor use was added to these subgroup analyses for this publication (Supplemental Figure 1).

The primary safety end point was the percentage of participants with procedure-related and investigational product-related treatment-emergent adverse event (AEs; treatment-emergent adverse events [TEAEs]). A secondary safety end point was the percentage of participants with procedure-related death.

## Results

**Figure 2:**
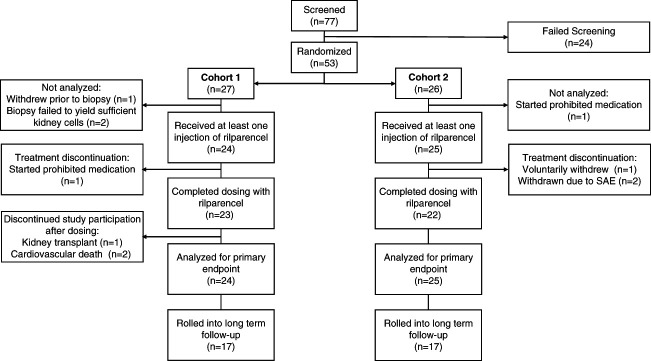
**Participants screened and treated in study REGEN-007.** Twenty-seven participants were randomized to Cohort 1; 24 received at least one injection with rilparencel and were included in the primary efficacy and safety analyses. Twenty-six participants were randomized to Cohort 2; 25 received at least one injection with rilparencel and were included in the primary efficacy and safety analyses.

Seventy-seven subjects were screened from July 30th, 2021, to March 21st, 2023; 53 participants were randomized at five sites in the United States, 27 participants to Cohort 1 and 26 to Cohort 2 (Figure 2). Four participants discontinued before receiving rilparencel. Thus, 49 participants were treated with rilparencel and included in the efficacy and safety analyses. Of these, 45 participants completed study treatment (*n*=23, Cohort 1; *n*=22, Cohort 2).

Most participants were male (69%) and of White race (88%) with a mean age of 60 years (Table 1). At baseline, 38 of 49 participants (78%) had type 2 diabetes mellitus, and 39 (80%) were taking an angiotensin-converting enzyme inhibitor or angiotensin II receptor blocker; the mean eGFR was 33 ml/min per 1.73 m^2^±10, and the mean HbA1c level was 7.5%±1.3. Notably, the UACR in Cohort 2 was less than half of that in Cohort 1 (792.0, Cohort 1 versus 229.0, Cohort 2).

**Table 1:** Demographics and baseline characteristics

Variable	Cohort 1 (*n*=24)	Cohort 2 (*n*=25)	Overall (*N*=49)
Age (yr), mean (SD)	62 (11)	58 (11)	60 (11)
**Age category, *n* (%)**			
Younger than 65 yr	13 (54)	16 (64)	29 (59)
65 yr or older	11 (46)	9 (36)	20 (41)
**Sex, *n* (%)**			
Male	16 (67)	18 (72)	34 (69)
Female	8 (33)	7 (28)	15 (31)
**Race, *n* (%)**			
Black	2 (8)	4 (16)	6 (12)
White	22 (92)	21 (84)	43 (88)
**Ethnicity, *n* (%)**			
Hispanic or Latino	0	1 (4)	1 (2)
Not Hispanic or Latino	24 (100)	24 (96)	48 (98)
**Diabetes type, *n* (%)**			
Type 1	3 (13)	8 (32)	11 (22)
Type 2	21 (88)	17 (68)	38 (78)
**BMI (kg/m** ^**2**^ **)**			
Mean (SD)	33.6 (6.3)	31.6 (5.5)	32.6 (6.0)
eGFR, ml/min per 1.73 m^2^			
Mean (SD)	31 (8)	34 (12)	33 (10)
**UACR (mg/g)**			
Median (IQR)	792 (71 to 1955)	229 (77 to 780)	421 (77 to 1546)
**HbA1c, (%)**			
Mean (SD)	7.2 (1.3)	7.8 (1.4)	7.5 (1.3)
**ACEi or ARBs usage, *n* (%)**			
Yes	18 (75)	21 (84)	39 (80)
No	6 (25)	4 (16)	10 (20)
**GLP-1 RA usage, *n* (%)**			
Yes	8 (33)	11 (44)	19 (39)
No	16 (67)	14 (56)	30 (61)
**SGLT2i usage, *n* (%)**			
Yes	10 (42)	8 (32)	18 (37)
No	14 (58)	17 (68)	31 (63)
**MRA/nsMRA usage, *n* (%)**			
Yes	4 (17)	1 (4)	5 (10)
No	20 (83)	24 (96)	44 (90)

eGFR was calculated using the 2009 CKD Epidemiology Collaboration equation.

Race and ethnicity was self-reported by participants.

ACEi, angiotensin-converting enzyme inhibitor; ARB, angiotensin II receptor blocker; BMI, body mass index; GLP-1 RA, glucagon-like peptide 1 receptor agonist; HbA1c, glycated hemoglobin; MRA, mineralocorticoid receptor agonist; nsMRA, non-steroidal mineralocorticoid receptor agonist; SGLT2i, sodium glucose co-transporter 2 inhibitor; UACR, urine-albumin/creatinine ratio.

### Extent of Exposure

In Cohort 1, 23 of 24 treated participants (96%) received two injections of rilparencel, and one (4%) received one injection. The median (min–max) time between injections in Cohort 1 was 4 months (3–13).

In Cohort 2, 15 of 25 treated participants (60%) received two doses of rilparencel; ten participants never met redosing criteria to qualify for a second injection. The median time between injections for those who received two doses of rilparencel was 11 months (5–15).

### Primary End Point

**Figure 3:**
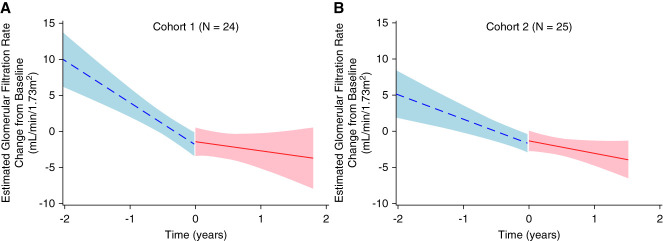
**Change in slope of eGFR from preinjection period to the period after the last rilparencel injection.** Dashed lines and solid lines represent regression of eGFR change over time (slope) in the preinjection and postinjection periods, respectively, for Cohort 1 (A) and Cohort 2 (B); shaded areas represent 95% CIs. CI, confidence interval.

In Cohort 1, the annual change in kidney function in the preinjection period, as measured by slope of eGFR, was −5.84 (SEM 1.07) ml/min per 1.73 m^2^ (95% CI, −7.97 to −3.70). In the period after the last injection, the annual change was −1.27 (SEM 1.36) ml/min per 1.73 m^2^ (95% CI, −3.97 to 1.43). The difference in the slope of eGFR between treatment periods was 4.57 ml/min per 1.73 m^2^ (95% CI, 1.95 to 7.18; Figure 3A).

In Cohort 2, the annual change in kidney function in the preinjection period, as measured by slope of eGFR, was −3.40 (SEM 0.81) ml/min per 1.73 m^2^ (95% CI, −5.03 to −1.77). In the period after the last injection, the annual change was −1.71 (SEM, 1.04) ml/min per 1.73 m^2^ (95% CI, −3.78 to 0.36). The difference in the slope of eGFR between treatment periods was 1.70 ml/min per 1.73 m^2^ (95% CI, −0.24 to 3.63; Figure 3B).

### Secondary End Points

#### Mean Change From Baseline in eGFR by Analysis Visit after the Last Injection

**Figure 4:**
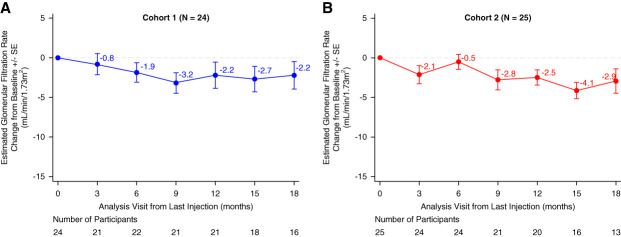
**Mean change from baseline in eGFR by analysis visit after last rilparencel injection.** The mean change in eGFR and corresponding SEM are shown at different study visits for Cohort 1 (A) and Cohort 2 (B). Baseline was defined as the average eGFR measurements taken within 21 days before the last rilparencel injection, excluding measurement on the biopsy or injection day postprocedure.

For Cohort 1, the mean change (SEM) from baseline in eGFR after the last injection ranged from −0.8 ml/min per 1.73 m^2^ (1.3) at Month 3 to −2.2 ml/min per 1.73 m^2^ (1.7) at month 18 (Figure 4A). For Cohort 2, the mean change (SEM) from baseline ranged from −2.1 ml/min per 1.73 m^2^ (1.1) to −2.9 ml/min per 1.73 m^2^ (1.5; Figure 4B).

#### Change in Slope of eGFR From First Injection to End of Study

The change in slope of eGFR from the first injection of rilparencel to the end of study visit was similar in both cohorts: −2.85 ml/min per 1.73 m^2^ (−5.35 to −0.34) in Cohort 1 and −2.19 ml/min per 1.73 m^2^ (−3.85 to −0.52) in Cohort 2.

#### Time to Event End Points

The number of participants in each cohort who experienced a secondary outcome are shown in Table 2. Apart from the four-component composite end point in Cohort 2, the limited number of events was insufficient to generate the Kaplan–Meier estimates of median event-free times (Table 3).

**Table 2:** Summary of same or reduced 5-year and 2-year risk of ESKD

Category	Cohort 1 (*N*=24)	Cohort 2 (*N*=25)
Same or reduced 5-yr risk at mo 12 after the first injection, *n* (%)	7 (29)	4 (16)
Same or reduced 5-yr risk at mo 18 after the first injection, *n* (%)	7 (29)	7 (28)
Same or reduced 2-yr risk at mo 12 after the first injection, *n* (%)	7 (29)	4 (16)
Same or reduced 2-yr risk at mo 18 after the first injection, *n* (%)	7 (29)	7 (28)

*n*, number of participants with same or reduced risk of ESKD.

Percentages are based on the number of participants who received at least one rilparencel injection in each randomized treatment group.

**Table 3:** Other secondary end point parameters

Category	Cohort 1 (*N*=24)	Cohort 2 (*N*=25)
**Time from first injection to ≥40% reduction in eGFR, sustained for 30 d**		
Number of participants with an event^a^, (*n* [%])	4 (17)	2 (8)
Probability of experiencing <40% reduction in eGFR (95% CI)^b^		
12 mo	0.87 (0.65 to 0.96)	0.96 (0.75 to 0.99)
18 mo	0.83 (0.60 to 0.93)	0.96 (0.75 to 0.99)
**Time from first injection to eGFR <15 ml/min per 1.732 m, sustained for 30 d and/or chronic dialysis and/or KRT**		
Number of participants with an event^a^ (*n* [%])	4 (17)	4 (16)
Probability of maintaining eGFR ≥15 ml/min per 1.73^2^ m and free from KRT (95% CI)^b^		
12 mo	0.91 (0.69 to 0.98)	0.92 (0.72 to 0.98)
18 mo	0.87 (0.65 to 0.96)	0.92 (0.72 to 0.98)
**Time from first injection to increase in UACR of ≥30% and ≥30 mg/g, sustained for 90 d**		
Number of participants with an event^a^, (*n* [%])	6 (25)	12 (48)
Probability of not experiencing UACR trigger (95% CI)^b^		
12 mo	0.75 (0.52 to 0.88)	0.63 (0.40 to 0.79)
18 mo	0.75 (0.52 to 0.88)	0.53 (0.31 to 0.71)
**Time from first injection to kidney or cardiovascular death**		
Number of participants with event^a^, (*n* [%])	2 (8)	0
Probability of not experiencing kidney or cardiovascular death (95% CI)		
12 mo	0.96 (0.74 to 0.99)	1.00 (1.00–1.00)
18 mo	0.96 (0.74 to 0.99)	1.00 (1.00–1.00)
**Time from first injection to earliest of three-component composite end point**		
Number of participants with an event (*n* [%])	7 (29)	5 (20)
Reason for event^a^ (*n* [%])		
At least 40% reduction in eGFR sustained for 30 d	4 (17)	2 (8)
eGFR <15 ml/min per 1.73^2^ sustained for 30 d and/or chronic dialysis, and/or kidney transplant	3 (13)	3 (12)
Kidney or cardiovascular death	2 (8)	0
Probability of not experiencing the three-component composite end point^b^		
12 mo	0.83 (0.61 to 0.93)	0.88 (0.67 to 0.96)
18 mo	0.79 (0.57 to 0.91)	0.88 (0.67 to 0.96)
**Time from first injection to earliest of four-component composite end point**		
Number of participants with an event (*n* [%])	10 (42)	15 (60)
Reason for event^a^ (*n* [%])		
At least 40% reduction in eGFR sustained for 30 d	2 (8)	2 (8)
eGFR <15 ml/min per 1.732 sustained for 30 d and/or chronic dialysis, and/or kidney transplant	2 (8)	3 (12)
At least 30% and 30 mg/g increase in UACR sustained for 90 d	6 (25)	10 (40)
Kidney or cardiovascular death	1 (4)	0
Probability of not experiencing the four-component composite end point^b^		
12 mo	0.67 (0.44 to 0.82)	0.55 (0.34 to 0.72)
18 mo	0.63 (0.40 to 0.79)	0.46 (0.26 to 0.64)

CI, confidence interval; UACR, urine-albumin/creatinine ratio.

a Percentages of participants with events are based on the number of participants who received at least one rilparencel injection in each randomized cohort.

b Calculated using Kaplan–Meier product-limit estimates with 95% confidence interval.

Seven of 24 (29%) participants in Cohort 1 and 5 out of 25 (20%) in Cohort 2 met criteria for the three-component composite end point (Table 3).

Ten of 24 participants (42%) in Cohort 1 and 15 out of 25 (60%) in Cohort 2 met criteria for the four-component composite end point (Table 3). In Cohort 2, the median event-free time for the four-component composite end point was 17 months.

#### Five-Year and Two-Year Risk of ESKD

The numbers and percentages of participants observed with the same or reduced 2-year risk of ESKD at Month 12 and Month 18 after the first rilparencel injection were the same as those observed for 5-year risk of ESKD (Table 2).

#### Safety

Of 87 rilparencel injections, 31 procedure-related TEAEs occurred in 16 participants (33%), and 14 product-related TEAEs occurred in six participants (12%; Table 4). Three participants experienced six biopsy-related serious adverse events (SAEs): Two participants experienced a subcapsular kidney hematoma, two participants experienced AKI, and one participant experienced hematuria resulting in hydronephrosis. One participant experienced an injection procedure-related SAE of subcapsular kidney hematoma. No product-related SAEs and no procedure-related or product-related deaths were reported. No biopsy-related, injection procedure-related, or product-related events led to treatment or study discontinuation. Tables presenting incidence of all AEs related to biopsy, injection procedure, and investigative product are located in the Supplemental Tables 1-6.

**Table 4:** Overall summary of adverse events, including primary and secondary safety end points

AE category	Cohort 1 *n* (%) E		Cohort 2 *n* (%) E		Overall *n* (%) E	
	Postbiopsy Period (*N*1=26)	Postinjection Period (*N*2=24)	Postbiopsy Period (*N*1=25)	Postinjection Period (*N*2=25)	Postbiopsy Period (*N*1=51)	Postinjection Period (*N*2=49)
Any AE	14 (54) 28	22 (92) 165	16 (64) 53	24 (96) 158	30 (59) 81	46 (94) 323
AE leading to treatment discontinuation	0	0	0	1 (4) 1	0	1 (2) 1
AE leading to study discontinuation	0	2 (8) 2	0	2 (8) 2	0	4 (8) 4
AE by relationship^a^ (investigator assessed)						
Biopsy-related AE	5 (19) 10	0	7 (28) 10	0	12 (24) 20	0
Injection procedure-related TEAE^b^	N/A	9 (38) 18	N/A	7 (28) 13	N/A	16 (33) 31
Product-related AE^b^	N/A	4 (17) 9	N/A	2 (8) 5	N/A	6 (12) 14
Any SAE	2 (8) 4	8 (33) 27	2 (8) 4	7 (28) 18	4 (8) 8	15 (31) 45
SAE leading to treatment discontinuation	0	0	0	1 (4) 1	0	1 (2) 1
SAE leading to study discontinuation	0	2 (8) 2	0	2 (8) 2	0	4 (8) 4
**SAE by relationship**^a^ **(investigator assessed)**						
Biopsy-related SAE	2 (8) 4	0	1 (4) 2	0	3 (6) 6	0
Injection procedure-related SAE^c^	N/A	1 (4) 1	N/A	0	N/A	1 (2) 1
Product-related SAE	N/A	0	N/A	0	N/A	0
SAE with outcome of death^d^ (investigator-assessed)	0	2 (8) 2	0	0	0	2 (4) 2
Biopsy-related death	0	N/A	0	N/A	0	N/A
Injection procedure-related death^b^	N/A	0	N/A	0	N/A	0
Product-related death	N/A	0	N/A	0	N/A	0
Any AESI	5 (19) 10	9 (38) 18	7 (28) 10	7 (28) 13	12 (24) 20	16 (33) 31
Kidney-specific AE	5 (19) 8	8 (33) 16	6 (24) 7	8 (32) 12	11 (22) 15	16 (33) 28
Biopsy-related hemorrhage	5 (19) 7	0	5 (20) 5	0	10 (20) 12	0
Injection procedure-related hemorrhage	N/A	2 (8) 3	N/A	4 (16) 5	N/A	6 (12) 8

AE, adverse event; AESI, adverse event of special interest; E, number of events; *n*, number of participants with adverse events; N/A, not applicable; N1, number of participants in the randomized cohort who received a biopsy; N2, number of randomized participants who received a biopsy and ≥1 one rilparencel injection; SAE, serious adverse event; TEAE, treatment-emergent adverse event (adverse events were considered treatment-emergent if they occurred after the first rilparencel injection).

a All adverse events judged by the Investigator to be possibly related or related are considered related adverse events.

b The primary safety end point was the percentage of participants with procedure-related and product-related treatment-emergent adverse events, and secondary safety end point was the summary of procedure-related death.

c One participant in Cohort 1 experienced an injection procedure-related serious adverse event of right subcapsular kidney hematoma.

d Two participants in Cohort 1 experienced cardiovascular death.

## Discussion

Slope of eGFR decline has been accepted by clinical and research communities and regulatory agencies as a valid surrogate clinical end point for CKD progression in clinical trials.^5,6^ This acceptance largely relies upon data from clinical trials in which participants were concurrently randomly assigned to either intervention or control groups. Although this phase 2 study did not use this rigorous design, use of historical control data do allow for a description of change in eGFR slope after an interventional treatment. This study demonstrated a clinically meaningful difference in CKD progression, as measured by change in eGFR slope before and after treatment with rilparencel, in participants assigned to scheduled, bilateral kidney dosing. The difference in eGFR slope of 4.6 ml/min per 1.73 m^2^ (95% CI, 1.95 to 7.18) per year in this cohort supports the potential clinical benefit of this treatment approach. The difference in eGFR slope in Cohort 2 was less pronounced at 1.7 ml/min per 1.73 m^2^, likely because of the different exposure to rilparencel versus Cohort 1. Twenty-three of 24 participants in Cohort 1 versus 15 of 25 participants in Cohort 2 received 2 injections of rilparencel with median time between injections much less in Cohort 1 versus Cohort 2 (4 months versus 11 months). The historical eGFR slope decline in Cohort 1 was also greater than the historical decline in Cohort 2, potentially because of more severe kidney injury in Cohort 1 as illustrated by a higher baseline UACR levels in Cohort 1.

The results of the current study are supported by high baseline use of kidney-protective medications; 80% of patients used angiotensin converting enzyme inhibitors or angiotensin receptor blockers. Although only 37% of study participants were treated with SGLT2 inhibitors at baseline, this utilization was far superior to prescribing patterns in the United States at a similar point in time.^7^ GLP-1 RA use at baseline (39%) was also relatively high given the lack of CKD guidelines supporting their use during this period. While these agents have been shown to slow the progression of CKD and represent a new pillar of standard-of-care treatment in type 2 diabetes mellitus, their specific effect on eGFR slope in randomized controlled trials is modest and highlights the need for additional therapeutic options to preserve kidney function. For example, semaglutide and SGLT2 inhibitors have demonstrated average differences in total eGFR slope decline versus placebo of 1.16–1.24 ml/min per 1.73 m^2^/year.^7,8^

The secondary end points in our study are less insightful because of the limited number of clinical events during follow-up and the use of a negligible change in UACR as a component of the four-component composite end point. Few participants met the three-component composite end point (29% and 20% in Cohorts 1 and 2, respectively). Notably, the probability of not meeting the end point was ≥80% for both cohorts through the 18-month follow-up period.

Continuation of the ongoing phase 3 study program is supported by the reassuring safety data in this report because no rilparencel-related SAEs and few rilparencel-related TEAEs were observed. Furthermore, no procedure-related AEs resulted in death. The incidence of biopsy-related procedures was less than or equal to those observed in a similar CKD population with diabetic kidney disease who underwent kidney biopsy to define kidney disease or assess rapid progression of CKD.^9^

Despite data supporting safety and efficacy of rilparencel, there are important study limitations to consider when interpreting the results. First, this was not a randomized, parallel arm, placebo-controlled study. Historical control data were used to calculate the pretreatment eGFR slope. Although some pretreatment data consisted of in-study eGFR measurements at predefined study visits, some control data were obtained by chart review and may be affected by collection and indication bias. Second, eGFR decline after study participant enrollment may have improved for reasons unrelated to the interventional cell therapy because of either a Hawthorne effect and/or cointerventions with newer therapeutic agents. Importantly, in a *post hoc* analysis, we were unable to find any significant imbalance in the addition or discontinuation of renoprotective medications in the pretreatment period versus the period after treatment with rilparencel. Third, because of the relatively small sample size and few clinical events, all secondary and subgroup analyses should be carefully interpreted. Finally, the study was conducted in the United States at five clinical study sites and enrolled principally White male participants, thereby limiting the generalizability of the findings.

A limited number of clinical trials have addressed the potential benefits of cell therapy in patients with CKD. Previous initial trials included use of different cell types,^10^ including CD34^+^,^11^ stem cells,^12^ mesenchymal stromal cells,^13,14^ and fat-tissue-derived cells.^15^ These were largely small studies that did not progress beyond phase 2 because of disappointing results.

Two studies warrant more detail. In a Phase 1b/2a in 30 participants with type 2 diabetes mellitus and moderate CKD, a single intravenous infusion of allogeneic mesenchymal precursor cells (rexlemestrocel-L) was safe and well tolerated. A trend to stabilized or improved GFR in participants allocated to rexlemestrocel-L was observed.^14^

Preliminary results from another Phase 1b/2a randomized clinical trial reported similar findings. In 16 participants (*n*=4 assigned to placebo) with type 2 diabetes mellitus and moderate progressive CKD, a single intravenous infusion of 80×10^6^ cells of the allogeneic mesenchymal stromal cell therapy, ORBCEL-M, was well tolerated. Compared with placebo, the median annual rate of eGFR decline was significantly lower with ORBCEL-M. Unfortunately, the full study was terminated before completion because of operational issues associated with the coronavirus disease 2019 pandemic.^13^

We have previously reported the preliminary results of an earlier Phase 2 study of rilparencel in patients with type 2 diabetes mellitus and moderate to advanced CKD.^16^ Unlike the current study, rilparencel was injected only in the biopsied kidney with the second injection occurring 6 months after the initial injection. Most of the study visits occurred during the coronavirus disease 2019 pandemic. Dropout rates were high, which limits the overall interpretation of the results. Rilparencel was well tolerated, and AEs were consistent with expected events from percutaneous kidney interventions. Although no difference in 12-month kidney function decline was observed in participants assigned rilparencel (versus standard-of-care), a strong efficacy signal was observed in patients with baseline eGFR <30 ml/min per 1.73 m^2^ and UACR >300 mg/g. No effect of cell therapy on urine albumin excretion was seen in any of these three cell therapy trials.

In summary, in this phase 2 clinical trial, bilateral kidney administration of rilparencel was associated with slowing the progression of CKD as shown by the difference in pretreatment eGFR slope versus eGFR slope after treatment with rilparencel. Ultimately, the placebo-controlled phase 3 trial will determine whether rilparencel represents a new treatment option for patients with advanced type 2 diabetes mellitus and CKD (ClinicalTrials.gov, NCT05099770).

## Supplementary Material

## Data Availability

Original data cannot be shared. Detailed Explanation Why Data Cannot Be Shared: Original data are a large data set from a clinical trial. Sharing of individual participant data with third parties was not specifically included in the informed consent of the study, and unrestricted diffusion of such data may pose a potential threat of revealing participants’ identities, as permanent data anonymization was not carried out (participants’ records were instead deidentified per protocol during the data retention process). To minimize this risk, individual participant data that underlie the results reported in this article may be available after completion of the long-term follow-up safety trial.

## Declarative Statements

This study includes clinical experimentation and received Institutional Review Board or Ethics Committee approval. All patients provided written informed consent. This study includes clinical experimentation and complies with the Declaration of Helsinki.

## Supplemental Material

Supplemental Figure 1. Difference in slope of eGFR in the period after last injection, by baseline characteristics.

Supplemental Table 1. AEs related to biopsy by preferred term (biopsied set).

Supplemental Table 2. Treatment-emergent AEs related to injection procedure by preferred term (safety set).

Supplemental Table 3. Treatment-emergent AEs related to investigative product by preferred term (safety set).

Supplemental Table 4. SAEs related to biopsy by preferred term (biopsied set).

Supplemental Table 5. Serious treatment-emergent AEs related to injection procedure by preferred term (safety set).

Supplemental Table 6. Serious treatment-emergent AEs related to investigative product by preferred term (safety set).

Statistical Analysis Plan.

Clinical Protocol.
