# Supplementary material for: A Randomized Clinical Trial of Kidney Autologous Cell Therapy in Diabetic Kidney Disease
Source: Clin J Am Soc Nephrol. 2026 Jan 2;21(5):744–53. doi: 10.2215/CJN.0000000969 (PMC13143438; doi:10.2215/CJN.0000000969)
Supplement: Supplementary file 1 [file cjasn-21-744-s001.pdf]

ASN American Society of Nephrology
JASN Journal of the American Society of Nephrology
KIDNEY360 Accessing Our World From Every Angle
CJASN Clinical Journal of the American Society of Nephrology

ASN Journal Disclosure Form

As per ASN journal policy, I have disclosed any financial relationships or commitments I have held in the past 36 months as included below. I have listed my Current Employer below to indicate there is a relationship requiring disclosure. If no relationship exists, my Current Employer is not listed.

A. Aqeel reports the following:
Employer: Nephrology Center; Ownership Interest: Ineligible companies; and Research Funding: Ineligible companies.

I understand that the information above will be published within the journal article, if accepted, and that failure to comply and/or to accurately and completely report the potential financial conflicts of interest could lead to the following: 1) Prior to publication, article rejection, or 2) Post-publication, sanctions ranging from, but not limited to, issuing a correction, reporting the inaccurate information to the authors' institution, banning authors from submitting work to ASN journals for varying lengths of time, and/or retraction of the published work.

Name: Ahmed Aqeel
Manuscript ID: (JASN-2025-001161)
Manuscript Title: A Randomized Clinical Trial,
Date of Completion: September 5, 2025
Disclosure Updated Date: September 5, 2025

ASN American Society of Nephrology
JASN Journal of the American Society of Nephrology
KIDNEY360 Accessing Our World From Every Angle
CJASN Clinical Journal of the American Society of Nephrology

ASN Journal Disclosure Form

As per ASN journal policy, I have disclosed any financial relationships or commitments I have held in the past 36 months as included below. I have listed my Current Employer below to indicate there is a relationship requiring disclosure. If no relationship exists, my Current Employer is not listed.

C. Barysauskas reports the following:
Employer: ProKidney; and Ownership Interest: ProKidney.

I understand that the information above will be published within the journal article, if accepted, and that failure to comply and/or to accurately and completely report the potential financial conflicts of interest could lead to the following: 1) Prior to publication, article rejection, or 2) Post-publication, sanctions ranging from, but not limited to, issuing a correction, reporting the inaccurate information to the authors' institution, banning authors from submitting work to ASN journals for varying lengths of time, and/or retraction of the published work.

Name: Constance M Barysauskas
Manuscript ID: CJASN-2025-001547R1
Manuscript Title: Renal Autologous Cell Therapy in Diabetes Mellitus and Chronic Kidney Disease: A Randomized Clinical Trial
Date of Completion: October 8, 2025
Disclosure Updated Date: October 8, 2025

ASN American Society of Nephrology | JASN Journal of the American Society of Nephrology | KIDNEY360 Accessing Our World From Every Angle | CJASN Clinical Journal of the American Society of Nephrology

ASN Journal Disclosure Form

As per ASN journal policy, I have disclosed any financial relationships or commitments I have held in the past 36 months as included below. I have listed my Current Employer below to indicate there is a relationship requiring disclosure. If no relationship exists, my Current Employer is not listed.

B. Cizman reports the following:
Employer: ProKidney-employee; Consultancy: ProKidney Corp.; and Ownership Interest: Stocks: GSK, ProKidney, Inozyme Pharma.

I understand that the information above will be published within the journal article, if accepted, and that failure to comply and/or to accurately and completely report the potential financial conflicts of interest could lead to the following: 1) Prior to publication, article rejection, or 2) Post-publication, sanctions ranging from, but not limited to, issuing a correction, reporting the inaccurate information to the authors' institution, banning authors from submitting work to ASN journals for varying lengths of time, and/or retraction of the published work.

Name: Borut Cizman
Manuscript ID: JASN-2025-001161
Manuscript Title: Renal Autologous Cell Therapy in Diabetes Mellitus and Chronic Kidney Disease: A Randomized Clinical Trial
Date of Completion: September 5, 2025
Disclosure Updated Date: September 5, 2025

ASN
American Society of Nephrology
JASN
Journal of the American Society of Nephrology
KIDNEY360
Accessing Our World From Every Angle
CJASN
Clinical Journal of the American Society of Nephrology

ASN Journal Disclosure Form

As per ASN journal policy, I have disclosed any financial relationships or commitments I have held in the past 36 months as included below. I have listed my Current Employer below to indicate there is a relationship requiring disclosure. If no relationship exists, my Current Employer is not listed.

E. Butler reports the following:
Employer: ProKidney; and Ownership Interest: ProKidney.

I understand that the information above will be published within the journal article, if accepted, and that failure to comply and/or to accurately and completely report the potential financial conflicts of interest could lead to the following: 1) Prior to publication, article rejection, or 2) Post-publication, sanctions ranging from, but not limited to, issuing a correction, reporting the inaccurate information to the authors' institution, banning authors from submitting work to ASN journals for varying lengths of time, and/or retraction of the published work.

Name: Emily Lynn Butler
Manuscript ID: CJASN-2025-001547R2
Manuscript Title: Renal Autologous Cell Therapy in Diabetes Mellitus and Chronic Kidney Disease: A Randomized Clinical Trial.
Date of Completion: November 21, 2025
Disclosure Updated Date: September 5, 2025

ASN American Society of Nephrology JASN Journal of the American Society of Nephrology KIDNEY360 Accessing Our World From Every Angle CJASN Clinical Journal of the American Society of Nephrology

ASN Journal Disclosure Form

As per ASN journal policy, I have disclosed any financial relationships or commitments I have held in the past 36 months as included below. I have listed my Current Employer below to indicate there is a relationship requiring disclosure. If no relationship exists, my Current Employer is not listed.

B. Culleton reports the following:
Employer: 1. ProKidney; 2. Sarepta Therapeutics; Ownership Interest: 1. ProKidney; 2. Sarepta Therapeutics; Research Funding: ProKidney; and Advisory or Leadership Role: Kidney Health Initiative (Board Member); Ellen Medical (Scientific Advisory Board member); NUCATS (External advisory board member); NUGoKidney (External advisory board member).

I understand that the information above will be published within the journal article, if accepted, and that failure to comply and/or to accurately and completely report the potential financial conflicts of interest could lead to the following: 1) Prior to publication, article rejection, or 2) Post-publication, sanctions ranging from, but not limited to, issuing a correction, reporting the inaccurate information to the authors' institution, banning authors from submitting work to ASN journals for varying lengths of time, and/or retraction of the published work.

Name: Bruce F. Culleton
Manuscript ID: JASN-2025-001161
Manuscript Title: Renal Autologous Cell Therapy in Diabetes Mellitus and Chronic Kidney Disease: A Randomized Clinical Trial
Date of Completion: September 5, 2025
Disclosure Updated Date: September 5, 2025

ASN American Society of Nephrology | JASN Journal of the American Society of Nephrology | KIDNEY360 Accessing Our World From Every Angle | CJASN Clinical Journal of the American Society of Nephrology

ASN Journal Disclosure Form

As per ASN journal policy, I have disclosed any financial relationships or commitments I have held in the past 36 months as included below. I have listed my Current Employer below to indicate there is a relationship requiring disclosure. If no relationship exists, my Current Employer is not listed.

R. Prakash reports the following:
Employer: Kidney Associates of Colorado; Ownership Interest: Fresenius Medical Care Joint Venture; and Research Funding: Prokidney; Novartis; Eli Lilly; Alnyam; Pathalys; Astra Zeneca; CSI Behring; Reata.

I understand that the information above will be published within the journal article, if accepted, and that failure to comply and/or to accurately and completely report the potential financial conflicts of interest could lead to the following: 1) Prior to publication, article rejection, or 2) Post-publication, sanctions ranging from, but not limited to, issuing a correction, reporting the inaccurate information to the authors' institution, banning authors from submitting work to ASN journals for varying lengths of time, and/or retraction of the published work.

Name: Rachita Prakash
Manuscript ID: JASN-2025-001161
Manuscript Title: Renal Autologous Cell Therapy in Diabetes Mellitus and Chronic Kidney Disease: A Randomized Clinical Trial
Date of Completion: September 5, 2025
Disclosure Updated Date: September 5, 2025

ASN
American Society of Nephrology
JASN
Journal of the American Society of Nephrology
KIDNEY360
Accessing Our World From Every Angle
CJASN
Clinical Journal of the American Society of Nephrology

ASN Journal Disclosure Form

As per ASN journal policy, I have disclosed any financial relationships or commitments I have held in the past 36 months as included below. I have listed my Current Employer below to indicate there is a relationship requiring disclosure. If no relationship exists, my Current Employer is not listed.

T. Saad reports the following:
Employer: Nephrology Associates, PA; Consultancy: HUMACYTE; WL GORE & ASSOC; VENOVA MEDICAL; Ownership Interest: OMNIAB INC ; AXSOME THERAPEUTICS INC; CORMEDIX INC; LIGAND PHARMA; TRAVERE THERAPEUTICS; VERA THERAPEUTICS; Research Funding: ASTRA ZENECA; NOVARTIS PHARMACEUTICALS; PATHALYS PHARMA; PROKIDNEY; MERIT MEDICAL SYSTEMS; Honoraria: CALLIDITAS PHAMACEUTICALS; and Advisory or Leadership Role: NEPHROLOGY ASSOCIATES, P.A., BOARD MEMBER UNPAID.

I understand that the information above will be published within the journal article, if accepted, and that failure to comply and/or to accurately and completely report the potential financial conflicts of interest could lead to the following: 1) Prior to publication, article rejection, or 2) Post-publication, sanctions ranging from, but not limited to, issuing a correction, reporting the inaccurate information to the authors' institution, banning authors from submitting work to ASN journals for varying lengths of time, and/or retraction of the published work.

Name: Theodore F. Saad
Manuscript ID: JASN-2025-001161
Manuscript Title: Renal Autologous Cell Therapy in Diabetes Mellitus and Chronic Kidney Disease: A Randomized Clinical Trial
Date of Completion: September 12, 2025
Disclosure Updated Date: September 12, 2025

ASN
American Society of Nephrology
JASN
Journal of the American Society of Nephrology
KIDNEY360
Accessing Our World From Every Angle
CJASN
Clinical Journal of the American Society of Nephrology

ASN Journal Disclosure Form

As per ASN journal policy, I have disclosed any financial relationships or commitments I have held in the past 36 months as included below. I have listed my Current Employer below to indicate there is a relationship requiring disclosure. If no relationship exists, my Current Employer is not listed.

A. Silva reports the following:
Employer: Boise Kidney & Hypertension Institute; CardioRenal Research Institute (CaRe); Consultancy: ProKidney, Vera Therapeutics, Caliditas, Apellis; Research Funding: Akebia; ProKidney; Ardelyx ; Novartis; Travere; Takeda; Vertex; Bayer; Enyo; Amgen;; Advisory or Leadership Role: ProKidney; Travere; Vera; and Speakers Bureau: Boehringer-Ingelheim; Astra Zeneca, Bayer; Travere; Apellis; Prime.

I understand that the information above will be published within the journal article, if accepted, and that failure to comply and/or to accurately and completely report the potential financial conflicts of interest could lead to the following: 1) Prior to publication, article rejection, or 2) Post-publication, sanctions ranging from, but not limited to, issuing a correction, reporting the inaccurate information to the authors' institution, banning authors from submitting work to ASN journals for varying lengths of time, and/or retraction of the published work.

Name: Arnold L. Silva
Manuscript ID: CJASN-2025-001547R1
Manuscript Title: Renal Autologous Cell Therapy in Diabetes Mellitus and Chronic Kidney Disease: A Randomized Clinical Trial
Date of Completion: October 9, 2025
Disclosure Updated Date: September 5, 2025

ASN American Society of Nephrology | JASN Journal of the American Society of Nephrology | KIDNEY360 Accessing Our World From Every Angle | CJASN Clinical Journal of the American Society of Nephrology

ASN Journal Disclosure Form

As per ASN journal policy, I have disclosed any financial relationships or commitments I have held in the past 36 months as included below. I have listed my Current Employer below to indicate there is a relationship requiring disclosure. If no relationship exists, my Current Employer is not listed.

J. Stavas reports the following:
Employer: ProKidney; Ownership Interest: ProKindey (individual stocks and options); and Research Funding: ProKidney.

I understand that the information above will be published within the journal article, if accepted, and that failure to comply and/or to accurately and completely report the potential financial conflicts of interest could lead to the following: 1) Prior to publication, article rejection, or 2) Post-publication, sanctions ranging from, but not limited to, issuing a correction, reporting the inaccurate information to the authors' institution, banning authors from submitting work to ASN journals for varying lengths of time, and/or retraction of the published work.

Name: Joseph Stavas
Manuscript ID: JASN-2025-001161
Manuscript Title: Renal Autologous Cell Therapy in Diabetes Mellitus and Chronic Kidney Disease: A Randomized Clinical Trial
Date of Completion: September 5, 2025
Disclosure Updated Date: September 5, 2025

ASN
American Society of Nephrology
JASN
Journal of the American Society of Nephrology
KIDNEY360
Accessing Our World From Every Angle
CJASN
Clinical Journal of the American Society of Nephrology

ASN Journal Disclosure Form

As per ASN journal policy, I have disclosed any financial relationships or commitments I have held in the past 36 months as included below. I have listed my Current Employer below to indicate there is a relationship requiring disclosure. If no relationship exists, my Current Employer is not listed.

T. Wooldridge reports the following:
Employer: Nephrology & Hypertension Associates, LTD; and Research Funding: Clinical research sponsored by Fresenius.

I understand that the information above will be published within the journal article, if accepted, and that failure to comply and/or to accurately and completely report the potential financial conflicts of interest could lead to the following: 1) Prior to publication, article rejection, or 2) Post-publication, sanctions ranging from, but not limited to, issuing a correction, reporting the inaccurate information to the authors' institution, banning authors from submitting work to ASN journals for varying lengths of time, and/or retraction of the published work.

Name: Thomas D. Wooldridge
Manuscript ID: CJASN-2025-001547R2
Manuscript Title: renal autologous cell therapy in diabetes mellitus and Chronic kidney disease
Date of Completion: November 21, 2025
Disclosure Updated Date: September 12, 2025

ASN American Society of Nephrology | JASN Journal of the American Society of Nephrology | KIDNEY360 Accessing Our World From Every Angle | CJASN Clinical Journal of the American Society of Nephrology

ASN Journal Disclosure Form

As per ASN journal policy, I have disclosed any financial relationships or commitments I have held in the past 36 months as included below. I have listed my Current Employer below to indicate there is a relationship requiring disclosure. If no relationship exists, my Current Employer is not listed.

H. Yan reports the following:
Employer: ProKidney Corp.; Fortrea

I understand that the information above will be published within the journal article, if accepted, and that failure to comply and/or to accurately and completely report the potential financial conflicts of interest could lead to the following: 1) Prior to publication, article rejection, or 2) Post-publication, sanctions ranging from, but not limited to, issuing a correction, reporting the inaccurate information to the authors' institution, banning authors from submitting work to ASN journals for varying lengths of time, and/or retraction of the published work.

Name: Hongxia Yan
Manuscript ID: JASN-2025-001161
Manuscript Title: Renal Autologous Cell Therapy in Diabetes Mellitus and Chronic Kidney Disease: A Randomized Clinical Trial
Date of Completion: September 8, 2025
Disclosure Updated Date: September 8, 2025
