# Supplementary material for: A Randomized Clinical Trial of Kidney Autologous Cell Therapy in Diabetic Kidney Disease
Source: Clin J Am Soc Nephrol. 2026 Jan 2;21(5):744–53. doi: 10.2215/CJN.0000000969 (PMC13143438; doi:10.2215/CJN.0000000969)
Supplement: Supplementary file 2 [file cjasn-21-744-s002.pdf]

Supplemental Material

A Randomized Clinical Trial of Kidney Autologous Cell Therapy in Diabetic Kidney Disease

Borut Čižman, Emily Butler, Joseph Stavas, Rachita Prakash, Theodore Saad, Arnold Silva, Thomas Wooldridge, Ahmed Aqeel, Hongxia Yan, Constance Barysauskas, Bruce Culleton

TABLE OF CONTENTS

Supplement Figure 1: Difference in Slope of Estimated Glomerular Filtration Rate between the Preinjection Period and the Period After the Last Injection, by Baseline Characteristic 2
Supplement Table 1: Adverse Events Related to Biopsy by Preferred Term (Biopsied Set) [a] 3
Supplement Table 2: Treatment-Emergent Adverse Events Related to Injection Procedure by Preferred Term (Safety Set) [a]....................................4
Supplement Table 3: Treatment-Emergent Adverse Events Related to Investigative Product by Preferred Term (Safety Set) [a]...5
Supplement Table 4: Serious Adverse Events Related to Biopsy by Preferred Term (Biopsied Set) [a] 5
Supplement Table 5: Serious Treatment-Emergent Adverse Events Related to Injection Procedure by Preferred Term (Safety Set) [a]....................................6
Supplement Table 6: Serious Treatment-Emergent Adverse Events Related to Investigative Product by Preferred Term (Safety Set) [a]...................6
STATISTICAL ANALYSIS PLAN..7
CLINICAL PROTOCOL...8

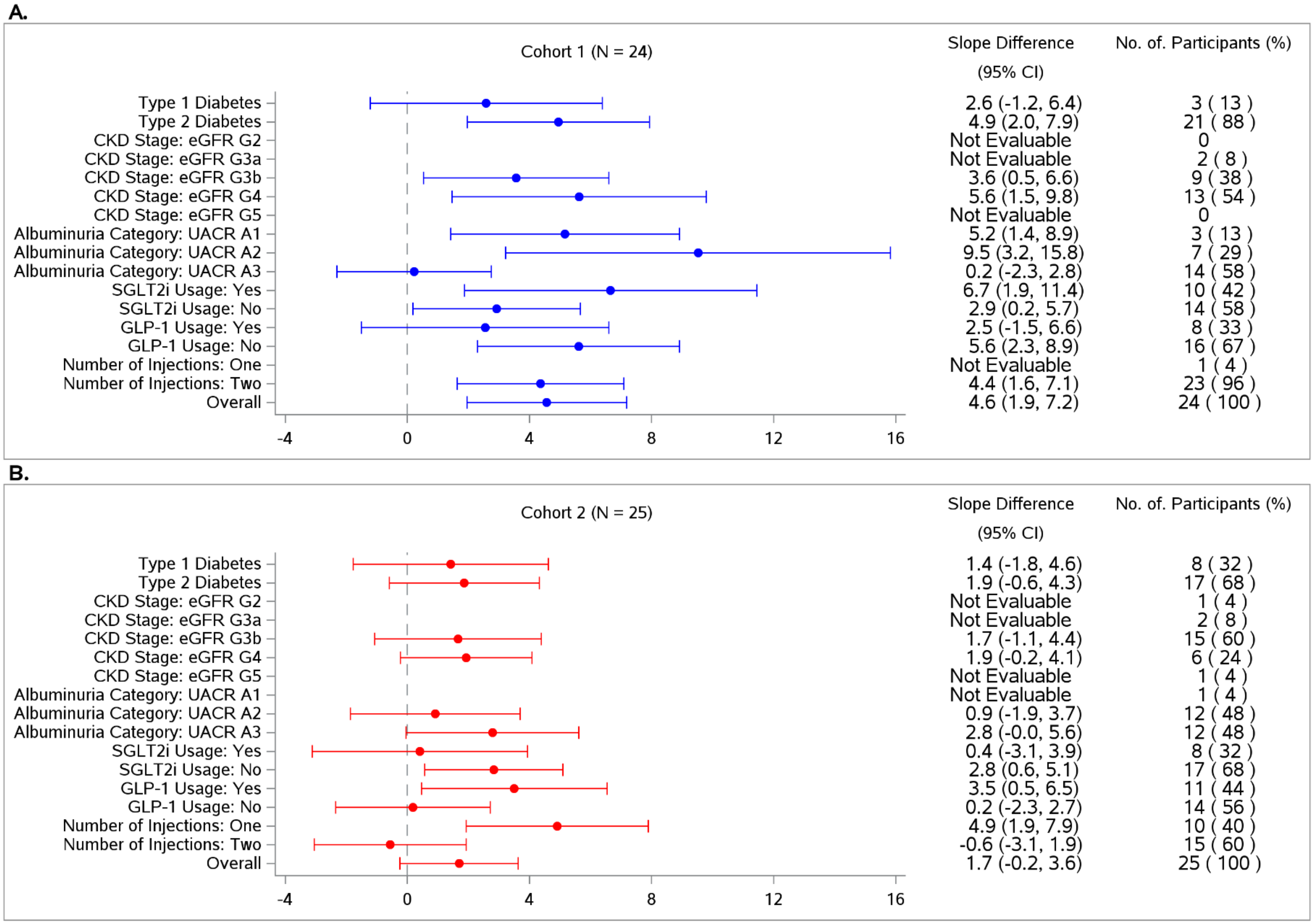

Supplement Figure 1: Difference in Slope of Estimated Glomerular Filtration Rate Between the Preinjection Period and the Period After the Last Injection, by Baseline Characteristic

Difference in eGFR slope and corresponding 95% confidence intervals are shown for different subgroups of participants as defined at Baseline for Cohort 1 (Panel A) and Cohort 2 (Panel B). CI = confidence interval; CKD = chronic kidney disease; eGFR = estimated glomerular filtration rate; GLP-1 = glucagon-like peptid-1; SGLT2i = sodium-glucose cotransporter-2 inhibitor; UACR = urine albumin-creatinine ratio

Supplement Table 1: Adverse Events Related to Biopsy by Preferred Term (Biopsied Set) [a]

Preferred Term	Cohort 1 (N=26) n (%)	Cohort 2 (N=25) n (%)	Overall (N=51) n (%)
Post-Biopsy Period [b]			
Any adverse event related to biopsy	5 (19)	7 (28)	12 (24)
Subcapsular renal haematoma	4 (15)	4 (16)	8 (16)
Acute kidney injury	1 (4)	1 (4)	2 (4)
Post procedural haemorrhage	1 (4)	1 (4)	2 (4)
Haematuria	1 (4)	0	1 (2)
Hydronephrosis	1 (4)	0	1 (2)
Injection site pain	0	1 (4)	1 (2)
Neck pain	0	1 (4)	1 (2)
Procedural pain	0	1 (4)	1 (2)
Retroperitoneal haematoma	1 (4)	0	1 (2)
Skin induration	1 (4)	0	1 (2)
Vomiting	0	1 (4)	1 (2)

a. Biopsy Set: All participants who had a biopsy were included in the Biopsy Set.

b. Post-Biopsy Period: The period from the start day of the biopsy up to but not including the date of the first rilparencel injection.

Note: Percentages are based on the number of participants in the preinjection period who had a biopsy.

Note: Adverse events are coded using MedDRA v28.0. At each level of preferred term, participants reporting more than 1 event are included only once.

Note: Causality for adverse events was assigned by the investigator. All adverse events judged by the investigator to be “possibly related” or “related” are considered related events.

Supplement Table 2: Treatment-Emergent Adverse Events Related to Injection Procedure by Preferred Term (Safety Set) [a]

Preferred Term	Cohort 1 (N=24) n (%)	Cohort 2 (N=25) n (%)	Overall (N=49) n (%)
Post-Injection Period [b]			
Any treatment-emergent adverse event (TEAE) related to injection procedure	9 (38)	7 (28)	16 (33)
Injection site pain	1 (4)	2 (8)	3 (6)
Subcapsular renal haematoma	2 (8)	1 (4)	3 (6)
Chills	2 (8)	0	2 (4)
Flank pain	1 (4)	1 (4)	2 (4)
Headache	2 (8)	0	2 (4)
Nausea	2 (8)	0	2 (4)
Procedural nausea	0	2 (8)	2 (4)
Renal haematoma	0	2 (8)	2 (4)
Abdominal pain	1 (4)	0	1 (2)
Back pain	1 (4)	0	1 (2)
Blood glucose increased	1 (4)	0	1 (2)
Blood loss anaemia	1 (4)	0	1 (2)
Dizziness	1 (4)	0	1 (2)
Fatigue	0	1 (4)	1 (2)
Oxygen saturation decreased	1 (4)	0	1 (2)
Pain	1 (4)	0	1 (2)
Perinephric collection	0	1 (4)	1 (2)
Post procedural contusion	0	1 (4)	1 (2)
Procedural vomiting	0	1 (4)	1 (2)
Retroperitoneal haematoma	0	1 (4)	1 (2)
Vomiting	1 (4)	0	1 (2)

a. Safety Set: All participants who had at least 1 rilparencel injection were included in the Safety Set.

b. Post-Injection Period: The period from the date of the first rilparencel injection to the date of study withdrawal or completion.

Note: Adverse events are coded using MedDRA v28.0. At each level of preferred term, participants reporting more than 1 event are included only once.

Note: Causality for adverse events was assigned by the investigator. All adverse events judged by the investigator to be “possibly related” or “related” are considered related events.

Supplement Table 3: Treatment-Emergent Adverse Events Related to Investigative Product by Preferred Term (Safety Set) [a]

Preferred Term	Cohort 1 (N=24) n (%)	Cohort 2 (N=25) n (%)	Overall (N=49) n (%)
Post-Injection Period [b]			
Any treatment-emergent adverse event (TEAE) related to investigative product	4 (17)	2 (8)	6 (12)
Procedural nausea	0	2 (8)	2 (4)
Chills	1 (4)	0	1 (2)
Dizziness	1 (4)	0	1 (2)
Fatigue	0	1 (4)	1 (2)
Headache	1 (4)	0	1 (2)
Injection site pain	1 (4)	0	1 (2)
Nausea	1 (4)	0	1 (2)
Oxygen saturation decreased	1 (4)	0	1 (2)
Pain	1 (4)	0	1 (2)
Procedural vomiting	0	1 (4)	1 (2)
Proteinuria	1 (4)	0	1 (2)
Renal haematoma	0	1 (4)	1 (2)
Vomiting	1 (4)	0	1 (2)

a. Safety Set: All participants who had at least 1 rilparencel injection were included in the Safety Set.

b. Post-Injection Period: The period from the date of the first rilparencel injection to the date of study withdrawal or completion.

Note: Adverse events are coded using MedDRA v28.0. At each level of preferred term, participants reporting more than 1 event are included only once.

Note: Causality for adverse events was assigned by the investigator. All adverse events judged by the investigator to be “possibly related” or “related” are considered related events.

Supplement Table 4: Serious Adverse Events Related to Biopsy by Preferred Term (Biopsied Set) [a]

Preferred Term	Cohort 1 (N=26) n (%)	Cohort 2 (N=25) n (%)	Overall (N=51) n (%)
Post-Biopsy Period [b]			
Any serious adverse event related to biopsy	2 (8)	1 (4)	3 (6)
Acute kidney injury	1 (4)	1 (4)	2 (4)
Haematuria	1 (4)	0	1 (2)
Hydronephrosis [c]	1 (4)	0	1 (2)
Subcapsular renal haematoma	1 (4)	1 (4)	2 (4)

a. Biopsied Set: All participants who had a biopsy.

b. Post-Biopsy Period: The period from the start day of the biopsy up to but not including the date of the first rilparencel injection. Biopsy Set: All participants who had a biopsy were included in the Biopsy Set.

c. The hydronephrosis serious adverse event causality was related to post-biopsy hematuria in the same patient.

Note: Adverse events are coded using MedDRA v28.0. At each level of preferred term, participants reporting more than 1 event are included only once.

Note: Causality for adverse events was assigned by the investigator. All adverse events judged by the investigator to be “possibly related” or “related” are considered related events.

Supplement Table 5: Serious Treatment-Emergent Adverse Events Related to Injection Procedure by Preferred Term (Safety Set) [a]

Preferred Term	Cohort 1 (N=24 n (%))	Cohort 2 (N=25) n (%)	Overall (N=49) n (%)
Post-Injection Period [b]			
Any serious treatment-emergent adverse event related to injection procedure	1 (4)	0	1 (2)
Subcapsular renal haematoma	1 (4)	0	1 (2)

a. Safety Set: All participants who had at least 1 rilparencel injection were included in the Safety Set.
b. Post-Injection Period: The period from the date of the first rilparencel injection to the date of study withdrawal or completion.

Note: Adverse events are coded using MedDRA v28.0. At each level of preferred term, participants reporting more than 1 event are included only once.

Note: Causality for adverse events was assigned by the investigator. All adverse events judged by the investigator to be “possibly related” or “related” are considered related events.

Supplement Table 6: Serious Treatment-Emergent Adverse Events Related to Investigative Product by Preferred Term (Safety Set) [a]

Preferred Term	Cohort 1 (N=24) n (%)	Cohort 2 (N=25) n (%)	Overall (N=49) n (%)
Post-Injection Period [b]			
Any serious treatment-emergent adverse event related to investigative product	0	0	0

a. Safety Set: All participants who had at least 1 rilparencel injection were included in the Safety Set.
b. Post-Injection Period: The period from the date of the first rilparencel injection to the date of study withdrawal or completion.

STATISTICAL ANALYSIS PLAN

A Phase 2, Randomized, Open-Label, Repeat Dose, Safety and Efficacy Study of Renal Autologous Cell Therapy (React) in Subjects with Type 1 Or 2 Diabetes and Chronic Kidney Disease (REGEN-007), Version 1.0

CLINICAL PROTOCOL

A Phase 2, Randomized, Open-Label, Repeat Dose, Safety and Efficacy Study of Renal Autologous Cell Therapy (React) in Subjects with Type 1 or 2 Diabetes and Chronic Kidney Disease (REGEN-007), Version 3.0

STATISTICAL ANALYSIS PLAN

Version 1.0

Date: 05 June 2025

A PHASE 2, RANDOMIZED, OPEN-LABEL, REPEAT DOSE, SAFETY AND EFFICACY STUDY OF RENAL AUTOLOGOUS CELL THERAPY (REACT) IN SUBJECTS WITH TYPE 1 or 2 DIABETES AND CHRONIC KIDNEY DISEASE (REGEN-007)

**Protocol Number:**	**REGEN-007**
**Protocol Version:**	**3.0**
**Protocol Date:**	**31 January 2023**

1 STATISTICAL ANALYSIS PLAN SIGNATURE PAGE

Author:

<table>
<tr><th>Name</th><th>Signature & Date</th></tr>
<tr><td>Hongxia Yan</td><td>Signed by Hongxia Yan
Hongxia Yan
I am the author of this document
05 June 2025 | 11:06 EDT
768D9C9B55584A0AB90D1D5001C20B3D
05 June 2025 | 11:06 EDT</td></tr>
<tr><td colspan="2">Title: Principal Biostatistician</td></tr>
<tr><td colspan="2">Company: ProKidney</td></tr>
</table>

Approved By:

<table>
<tr><th>Name</th><th>Signature & Date</th></tr>
<tr><td>Connie Barysauskas</td><td>Signed by Connie Barysauskas
Connie Barysauskas
I approve this document
05 June 2025 | 11:03 EDT
36B9E414B1664577B425C81A9376A78B
05 June 2025 | 11:05 EDT</td></tr>
<tr><td colspan="2">Approval Role: Biostatistics Reviewer</td></tr>
<tr><td colspan="2">Title: Director, Biostatistics</td></tr>
<tr><td colspan="2">Company: ProKidney</td></tr>
</table>

<table>
<tr><th>Name</th><th>Signature & Date</th></tr>
<tr><td>Lillie Wang</td><td>Signed by Lillie Wang
Lillie Wang
I approve this document
05 June 2025 | 11:12 EDT
3CB9DFBD74684E8E918754A947148B32
05 June 2025 | 11:13 EDT</td></tr>
<tr><td colspan="2">Approval Role: Statistical Programming Reviewer</td></tr>
<tr><td colspan="2">Title: Senior Principal Programmer</td></tr>
<tr><td colspan="2">Company: ProKidney</td></tr>
</table>

<table>
<tr><th>Name</th><th>Signature & Date</th></tr>
<tr><td>Lauren Weintraub</td><td>Signed by Lauren Weintraub
Lauren Weintraub
I approve this document
05 June 2025 | 11:23 EDT
97811429206347759C050BA6055E7938
05 June 2025 | 11:23 EDT</td></tr>
<tr><td colspan="2">Approval Role: Clinical Development (or designee)</td></tr>
<tr><td colspan="2">Title: Sr. Medical Director</td></tr>
<tr><td colspan="2">Company: ProKidney</td></tr>
</table>

REVISION HISTORY

Version	Date of Version	Summary of Changes
1.0	05JUN2025	Initial version

TABLE OF CONTENTS

1 STATISTICAL ANALYSIS PLAN SIGNATURE PAGE 2

REVISION HISTORY 3

LIST OF TABLES 8

LIST OF FIGURES 9

LIST OF APPENDICES 9

LIST OF ABBREVIATIONS 10

2 INTRODUCTION 12

3 STUDY OBJECTIVES AND ENDPOINTS 12

4 STUDY DESIGN 13

4.1 General Description 13

4.2 Geographic Locations of Sites 15

4.3 Schedule of Events 15

4.4 Dosing 15

4.5 Participants Withdrawal and Replacement 16

4.6 Randomization 16

4.7 Blinding 16

4.8 Cohort 2 Redosing Verification Committee 17

5 POWER AND SAMPLE SIZE 17

6 PLANNED ANALYSIS TIME POINTS 17

6.1 Data Monitoring Committee (DMC) 17

6.2 Interim Analysis 17

6.3 Final Analysis 17

7 CHANGES TO ANALYSIS FROM PROTOCOL 18

8 GENERAL CONSIDERATIONS FOR DATA ANALYSIS 19

8.1 Analysis Sets 20

8.2 Method for Handling Missing Data 20

8.2.1 Partial or Missing Concomitant Medication (CM), Concomitant Procedures (CP), and Adverse Event (AE) Date Handling 21

8.2.2 Algorithm for Partial or Missing Event Dates for Time to Event Analyses 22
8.2.3 Algorithm for Missing Adverse Event Information 22
8.3 Derivations 22
8.3.1 Reference Date and Study Day 22
8.3.2 Baseline Definition 23
8.3.3 Analysis Periods 23
8.3.4 Common Derivations 24
8.3.5 Derivation of Length of Follow-Up 24
8.3.6 Derivation of eGFR 25
8.3.7 Derivation of UACR 26
8.3.8 Stage of Chronic Kidney Disease (eGFR and Albuminuria Category) 26
8.3.9 Derivation of 5-year and 2-year Risk of ESRD 26
8.3.10 Derivation of Incidence and Time from First REACT Injection to at Least 40% Reduction in eGFR 27
8.3.11 Derivation of Incidence and Time from First REACT Injection to eGFR <15 mL/min/1.73m^2 or Renal Replacement Therapy (RRT) 28
8.3.12 Derivation of Incidence and Time from First REACT Injection to at least 30% and 30 mg/g Increase in UACR Sustained for 90 Days 29
8.3.13 Derivation of Incidence and Time from First REACT Injection to Renal or Cardiovascular Death 31
8.3.14 Derivation of Incidence and Time from First REACT Injection to Four Composite Endpoint 31
8.3.15 Derivation of Incidence and Time from First REACT Injection to Three Composite Endpoint 33
8.3.16 Derivation of Incidence and Time from First REACT Injection to All-Cause Mortality 34
8.4 Site Pooling Strategies 35
8.5 Visit and Unscheduled Visits 35

8.6 Windowing Conventions....35
8.7 Laboratory Data....37
8.8 Statistical Tests....37
8.9 Multiple Comparisons and Multiplicity....37
8.10 Software Version....37
8.11 Example Table Column Presentation....38
8.11.1 Outputs for 14.1.X Demographic Data....39
8.11.2 Outputs for 14.2.X Efficacy Data....39
8.11.3 Outputs for 14.3.X Safety Data....39
9 EXAMINATION OF SUBGROUPS....41
10 PARTICIPANT SUMMARY....41
10.1 Participant Disposition....41
10.2 Protocol Deviations....42
10.3 Demographic and Screening, Baseline Characteristics....43
10.4 Medical History....44
10.5 Medical History of Chronic Kidney Disease....44
10.6 Study Intervention – Biopsy....44
10.7 Study Treatment Exposure, Compliance and Relative Intensity - REACT Injections....44
10.8 Redosing Trigger....45
10.9 Prior and Concomitant Medications....45
10.9.1 Prior and Concomitant Medications of Special Interest....46
10.10 Prior and Concomitant Procedures....47
11 EFFICACY ANALYSIS....47
11.1 Primary Efficacy Endpoint and Analysis....47
11.2 Secondary Efficacy Endpoints and Analyses....50
11.2.1 Slope of Estimated Glomerular Filtration Rates Change from Baseline....50

11.2.2 5-year and 2-year risk of ESRD at 12 months and 18 months after first injection....52
11.2.3 Time from First REACT Injection to at least 40% Reduction in eGFR 53
11.2.4 Time from First REACT Injection to eGFR <15 mL/min/1.73m^2 or Renal Replacement Therapy 54
11.2.5 Time from First REACT Injection to at least 30% and 30 mg/g Increase in UACR 54
11.2.6 Time from First REACT Injection to Renal or Cardiovascular Death 54
11.2.7 Time from First REACT Injection to Four Composite Endpoint....55
11.2.8 Time from First REACT Injection to Three Composite Endpoint ..55
11.3 Exploratory Efficacy Endpoints and Analyses....56
11.3.1 Efficacy Laboratory Measurements....56
11.3.2 Time from First REACT Injection to All-cause Mortality 57
12 SAFETY ANALYSIS 57
12.1 Adverse Events....57
12.1.1 Treatment-Emergent Adverse Events....58
12.1.2 Adverse Event Intensity....58
12.1.3 Adverse Event Relationship to Biopsy, Investigational Product, or Injection Procedure....58
12.1.4 Serious Adverse Events 58
12.1.5 Adverse Events of Special Interest (AESI)....59
12.1.6 Renal-specific Adverse Events 59
12.1.7 Adverse Events Leading to Treatment Discontinuation, Study Withdrawal or Death 59
12.1.8 Adverse Event Summaries....59
12.2 Clinical Laboratory Assessments 62
12.3 Pregnancy Test 64
12.4 Research Samples....65

12.5 Vital Signs 65
12.6 Electrocardiogram (ECG) Assessments 65
12.7 Physical Examination 66
12.8 Renal Safety Ultrasound 66
12.9 Renal Imaging 66
12.10 Dialysis and Renal Transplant 66
12.11 COVID-19 Impact 67
13 REFERENCES 68
14 APPENDICES 69

LIST OF TABLES

Table 1: Study Objectives and Endpoints 12
Table 2: REACT Dosing Relative to Estimated Kidney Weight 15
Table 3: Analysis Sets 20
Table 4: Categorization of eGFR 26
Table 5: Categorization of Albuminuria (Urinary Albumin/Creatinine Ratio) 26
Table 6: Time from First REACT Injection to at least 40% Reduction in eGFR 28
Table 7: Time from First REACT Injection to eGFR <15 mL/min/1.73m^2 or RRT Definitions 29
Table 8: Time from First REACT Injection to at least 30% and 30 mg/g Increase in UACR 30
Table 9: Time from First REACT Injection to Renal or Cardiovascular Death Definitions 31
Table 10: Time from First REACT Injection to Four Composite Endpoint Definitions ..32
Table 11: Time from First REACT Injection to Three Composite Endpoint Definitions 33
Table 12: Time from First REACT Injection to All-Cause Mortality Definitions 35
Table 13: Analysis Visit Window Mapping for Cohort 1 with two REACT Injections...36
Table 14: Analysis Visit Window Adjustment for Cohort 2 36
Table 15: Character to Numeric Conversion for LOQ results 37
Table 16: Models used to Estimate Slopes of eGFR Change from Pre-injection to Post-last Injection 49
Table 17: Model used to Estimate Slope of Estimated Glomerular Filtration Rates Change from Baseline 51

Table 18: Models used to Estimate Slopes of eGFR Change from Pre-injection to Post-First Injection 52
Table 19: Censoring Reason for Time to least 40% Reduction in eGFR 53
Table 20: Censoring Reason for Time to Renal Replacement Therapy 54
Table 21: Censoring Reason for Time to Renal or Cardiovascular Death 55
Table 22: Censoring Reason for Time to Four Composite Endpoint 55
Table 23: Clinical Laboratory Evaluations 63

LIST OF FIGURES

Figure 1: Schematic of Study Design 14
Figure 2: Analysis Period Diagram....23

LIST OF APPENDICES

Appendix 1: Time and Events Table 70
Appendix 2: Laboratory Time and Events Appendix 75
Appendix 3: Sample SAS Code for Statistical Analyses....81
Appendix 4: Renal-specific Adverse Events by Preferred Term....83

LIST OF ABBREVIATIONS

Abbreviation	Definition
AE	adverse event
AESI	adverse event of special interest
ATC	Anatomical, Therapeutic, and Chemical
BMI	body mass index
BS	biopsied set
CI	confidence interval
CKD	chronic kidney disease
CKD-EPI	Chronic Kidney Disease Epidemiology Collaboration
CM	concomitant medication
CP	concomitant procedures
CRF	case report form
CSR	clinical study report
CT	computed tomography
CTCAE	Common Terminology Criteria for Adverse Events
DMC	Data Monitoring Committee
ECG	electrocardiogram
EDC	Electronic Data Capture
eGFR	estimated glomerular filtration rate
EOS	end of study
ESRD	end-stage renal disease
FAS	full analysis set
FDA	Food and Drug Administration
g	gram(s)
Hb	hemoglobin
HbA1c	glycosylated hemoglobin
HBV	hepatitis B virus
HCV	hepatitis C virus
HIV	human immunodeficiency virus
ICH	International Conference on Harmonisation
iPTH	intact parathyroid hormone
ITT	intent-to-treat analysis set
MDRD	Modification of Diet in Renal Disease
MedDRA	Medical Dictionary for Regulatory Activities
mITT	modified intent-to-treat analysis set
nsMRA	non-steroidal mineralocorticoid
KDIGO	Kidney Disease Improving Global Outcomes
K-M	Kaplan-Meier
MRI	magnetic resonance imaging
NCI	National Cancer Institute
PT	preferred term
REACT	renal autologous cell therapy
RRT	renal replacement therapy
SAE	serious adverse event
SAF	safety analysis set

Abbreviation	Definition
SAP	statistical analysis plan
SCr	serum creatinine
SI	International System of Units
SOC	system organ class
SRC	selected renal cells
STD	standard deviation
T1DM	type 1 diabetes mellitus
T2DM	type 2 diabetes mellitus
TEAE	treatment-emergent adverse event
UACR	urine albumin to creatinine ratio
WHO	World Health Organization
WHODrug	World Health Organization Drug Dictionary

2 INTRODUCTION

This statistical analysis plan (SAP) describes the statistical methods, rules, and conventions to be used in the presentation and analysis of efficacy and safety data for protocol REGEN-007. It describes the data to be summarized and analyzed, including the specifics of statistical tests to be performed. The SAP should be read in conjunction with the study protocol and case report forms (CRFs).

This SAP is based on the protocol number REGEN-007 v 3.0*,* dated 31January 2023. Any changes to the protocol and/or CRFs may necessitate an update to the SAP prior to database lock. If the SAP and final protocol are different, the SAP prevails. Any deviations from this plan will be documented in the clinical study report (CSR).

3 STUDY OBJECTIVES AND ENDPOINTS

The objectives and endpoints for the study are summarized in the Table 1 as below.

Table 1: Study Objectives and Endpoints

Objective	Endpoint(s)
**---**	**Primary Efficacy Endpoint**
To evaluate the safety, efficacy and durability of up to two REACT injections delivered percutaneously into biopsied and non-biopsied contralateral kidneys on renal function progression in 2 different cohorts of participants with type 1 diabetes mellitus (T1DM) or type 2 diabetes mellitus (T2DM) diabetes and chronic kidney disease (CKD).	Change from pre-injection to post-last injection total (acute + chronic) slope of estimated glomerular filtration rate (eGFR) using the 2009 Chronic Kidney Disease Epidemiology Collaboration (CKD-EPI 2009) serum creatinine equation.
	**Secondary Efficacy Endpoints**
	Slope of eGFR, determined using CKD-EPI 2009, change from first injection to End of Study (EOS).
	Time from first injection to at least 40% reduction in eGFR, using the 2009 CKD-EPI serum creatinine equation, sustained for 30 days.
	Time from first injection to eGFR <15 mL/min/1.73m^2 using the 2009 CKD-EPI serum creatinine equation, sustained for 30 days and/or chronic dialysis, and/or renal transplant.
	Time from first injection to increase of urine microalbumin to creatinine ratio (UACR) of at least 30% and of at least 30 mg/g, using the urine microalbumin/urine creatinine ratio sustained for 90 days.
	Time from first injection to renal or cardiovascular death.
	The time from first injection to earliest of the following:<br>1. At least 40% reduction in eGFR, using the 2009 CKD-EPI serum creatinine equation, sustained for 30 days or<br>2. eGFR <15 mL/min/1.73m^2 using the 2009 CKD-EPI serum creatinine equation, sustained for 30 days and/or chronic dialysis, and/or renal transplant or<br>3. Increase of UACR of at least 30% and of at least 30 mg/g, using the urine microalbumin/urine creatinine ratio sustained for 90 days or<br>4. Renal or cardiovascular death.

Objective	Endpoint(s)
	The time from first injection to earliest of the following:<br>1. At least 40% reduction in eGFR, using the 2009 CKD-EPI serum creatinine equation, sustained for 30 days or<br>2. eGFR <15 mL/min/1.73m² using the 2009 CKD-EPI serum creatinine equation, sustained for 30 days and/or chronic dialysis, and/or renal transplant or<br>3. Renal or cardiovascular death.
	Percent of participants who have the same or reduced 5-year risk of End-Stage Renal Disease (ESRD) and 2-year risk of ESRD at 12 and 18 months after the first injection.
	**Exploratory Efficacy Endpoints**
	Change from baseline on following parameters:<br>• blood hemoglobin<br>• blood hematocrit<br>• serum calcium<br>• serum phosphorus<br>• plasma parathyroid hormone<br>• serum potassium<br>• serum bicarbonate<br>• eGFR in 2009 CKD-EPI<br>• eGFR in 2012 CKD-EPI<br>• UACR.
	Time from first injection to all-cause mortality.
	**Primary Safety Endpoint**
	Percentage of participants with procedure and investigational product-related treatment-emergent adverse events (TEAEs).
	**Secondary Safety Endpoint**
	Summary of procedure-related death.
	**Exploratory Safety Endpoints**
	The collected value and the change from baseline of follows:<br>• Physical examination<br>• Vital signs<br>• Safety laboratory analytes<br>• 12-lead electrocardiogram (ECG)<br>• Renal volume<br>• Renal cortical thickness.

4 STUDY DESIGN

4.1 General Description

This is a multi-center, prospective, open-label study, eligible participants will be randomized (1:1) to one of two treatment groups (Cohort 1 or Cohort 2). Participants randomized to Cohort 1 will receive two scheduled REACT injections given 3 months apart (+60 days). The first REACT injection will be given in the biopsied kidney and the second injection will be given in the non-biopsied contralateral kidney (i.e., the kidney that was not injected with the first injection). Participants will be followed every 3 months for at least 18 months after the last

REACT injection, or until the global study end date, which is the date when all participants complete at least 6 months of study post-last REACT injection follow-up.

Participants randomized to Cohort 2 will receive one scheduled REACT injection with a possible second REACT injection upon meeting a redose trigger. A second injection would be within at least 30 days of meeting a renal function redose trigger with sustainment, participants will be followed every 3 months until the end of study. The redose trigger will be evaluated beginning at Month 3 post-first REACT injection visit through Month 15 post-first REACT injection visit. The first REACT injection will be given in the biopsied kidney and the second injection will be given in the non-biopsied contralateral kidney (i.e., the kidney that was not injected with the first injection). If a redose trigger is not met within 15 months after the first REACT injection, participants will complete their participation in this study having received only 1 REACT injection.

Redose triggers for Cohort 2 are defined below:

1. Thirty-day sustained decline in eGFR by at least 20% from Baseline value confirmed with repeat laboratory testing and/or
2. Increase of greater than or equal to 30% and of at least 30 mg/g in UACR from Baseline, using a standard urine chemistry, sustained for at least 30 days with two repeat central laboratory testing 7 days apart at least 30 days after initial event for confirmation.

A schematic of the study design is presented in Figure 1.

Figure 1: Schematic of Study Design

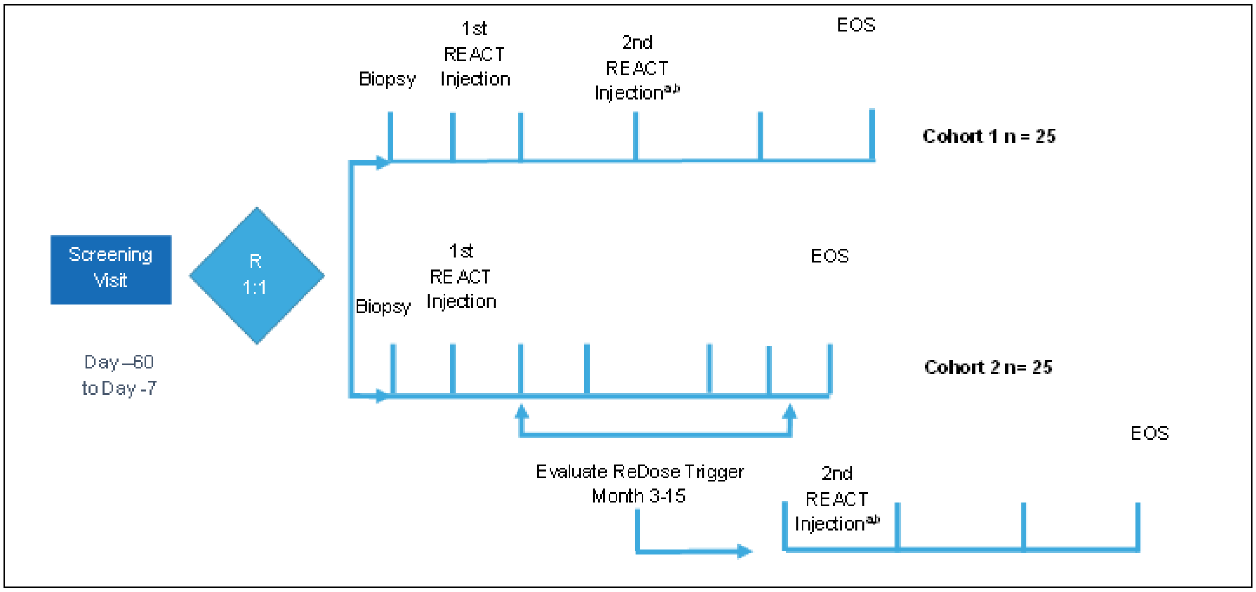

a. The second REACT injection will be performed in the contralateral kidney (i.e., the kidney not injected with the first REACT injection).
b. The second REACT injection will take place no sooner than 3 months after the first REACT injection.

4.2 Geographic Locations of Sites

This study will be conducted at 5 sites in the U.S.

4.3 Schedule of Events

The schedule of events can be found in Appendix 1. The laboratory schedule of events can be found in Appendix 2.

4.4 Dosing

The dose of REACT will be 3 × 10^6 cells/g estimated kidney weight (g KW^{est}). Since the concentration of selected renal cells (SRC) per mL of REACT is 100 × 10^6 cells/mL ± 20%, the dosing volume will be 3.0 mL for each 100 g of kidney weight. Using this dosing paradigm, the following table (Table 2) shows the dosing volume and number of SRC to be delivered relative to estimated kidney weight. The maximum volume of REACT injected into the kidney during an injection will be 8.0 mL. The volume of REACT to be administered will be determined by pre-procedure renal magnetic resonance imaging (MRI) or computed tomography (CT) volumetric 3-dimensional evaluation.

Table 2: REACT Dosing Relative to Estimated Kidney Weight

Estimated Kidney Weight (g KW^{est}) [a,b]		REACT Dosing Volume (mL)	SRC Delivered (Number of Cells × 10^6)
Median Weight (g)	Weight Range (g)		
100	95 – 108	3.0	300
117	109 – 125	3.5	350
133	126 – 141	4.0	400
150	142 – 158	4.5	450
167	159 – 175	5.0	500
183	176 – 191	5.5	550
200	192 – 208	6.0	600
217	209 – 225	6.5	650
233	226 – 241	7.0	700
250	242 – 258	7.5	750
— — —	>259	8.0 [c]	800

Abbreviations: CT = computed tomography; g KW_{est} = grams estimated kidney weight; MRI = magnetic resonance imaging; REACT = renal autologous cell therapy; SRC = selected renal cells.

a. The dose of REACT will be 3 ×10^6 cells/g estimated kidney weight.
b. Kidney weight will be estimated from the results of an MRI study performed on or after the Screening Visit until Day 0 (renal biopsy). If a participant cannot undergo MRI then CT will be substituted to obtain kidney size and volume.
c. 8 mL will be the maximum dosing volume (mL).

The dose of REACT will be based on kidney volume calculated from the results of the MRI study performed from the time of the Screening Visit prior to the renal biopsy. As a conservative approach, the REACT dose will be calculated using a conversion of 1g equals 1 mL.

4.5 Participants Withdrawal and Replacement

Participants may withdraw from the study at any time and for any reason without penalty or prejudice, and without jeopardizing access to future medical care. If the Investigator determines that continuing in the study is no longer in the best interest of the participant, then the Investigator should withdraw the participant from the study. If a participant voluntarily withdraws from the study, the reason(s) should be documented. Participants who withdraw from the study or are withdrawn by the Investigator following randomization should undergo procedures specified at the End of Study (EOS) Visit.

Participants who withdraw from the study before randomization will be considered screen failures; screen failures and the participants who withdraw from the study after randomization, but before having the renal biopsy will be replaced. Participants who withdraw from the study following the renal biopsy but before the first REACT injection may be replaced.

4.6 Randomization

Approximately 50 participants will be randomized to 1 of 2 cohorts in a 1:1 ratio (25 participants per cohort). Eligible participants will be assigned unique numbers (randomization number) in sequential order. The randomization number codes the participant’s cohort assignment according to the randomization schedule generated prior to the study starting. Randomization follows a permuted block design with no stratification factors.

Randomization numbers will not be re-used once assigned. If a participant is replaced, the replaced participant will receive a new cohort assignment at random, per the randomization schedule. The replacement participant may or may not have the same cohort assignment as the replaced participant. Once a participant is assigned a randomization number, that number is retained throughout the entirety of the study, including should the participant move to a different site.

4.7 Blinding

Not applicable as this is an open-label study.

4.8 Cohort 2 Redosing Verification Committee

The determination of whether the redose trigger criteria has been met will be performed by an independent verification committee, as described by the Verification Committee Charter. Only participants with confirmed eligibility of the redose trigger will receive the second REACT injection in Cohort 2.

5 POWER AND SAMPLE SIZE

Approximately 50 participants will be treated with REACT. As this is a Phase 2 exploratory safety and efficacy study, no formal statistical testing will be performed.

6 PLANNED ANALYSIS TIME POINTS

6.1 Data Monitoring Committee (DMC)

An independent Data Monitoring Committee (DMC) has been chartered to ensure the safety of participants enrolled in this study, especially as it relates to unexpected investigational product-related events. The DMC will consist of members who have expertise directly related to protocol-specified activities. It will function independently, and its members will have no other engagement with ProKidney. The DMC will meet by teleconference (or other means) at regular intervals, depending on the speed of participant enrollment and the amount of new data generated. The DMC will advise ProKidney on aspects concerning the safety of participants participating in the clinical trial. Other specific activities, responsibilities, and composition of the DMC will be described in the DMC charter.

DMC analyses are outside the scope of this SAP.

6.2 Interim Analysis

No interim analyses are planned for this study.

6.3 Final Analysis

All participants will be followed until the end of study. The final analysis will occur when the last participant completes the 6 months of post-last REACT injection follow-up, or when the last participant is considered lost to follow-up, withdraws consent, or dies, and the database has been locked.

7 CHANGES TO ANALYSIS FROM PROTOCOL

Changes	Protocol	SAP
Definition of Biopsied Set	In Section 9.2.2.1, the Biopsied Set is defined as: all participants who had a biopsy, but did not receive any REACT injections.	In SAP, the Biopsied Set is defined as all participants who had received a biopsy.
Baseline definition	Per protocol version 3.0, baseline is defined as the last non-missing value prior to the first REACT injection.	In SAP, the baseline is defined as:Unless otherwise specified, the baseline value is defined as the latest non-missing value collected prior to the first REACT injection, excluding Post Procedure Biopsy Day.
Secondary efficacy endpoint	Estimated glomerular filtration rates, determined using CKD-EPI 2009, from first injection to at least 18 months after the last REACT injection.	Slope of eGFR, determined using CKD-EPI 2009, from first injection to End of Study.
Secondary efficacy endpoint	The time from first injection to increase of UACR of at least 30% and of at least 30 mg/g, using the random urine microalbumin/urine creatinine ratio sustained for 90 days.	The time from first injection to increase of UACR of at least 30% and of at least 30 mg/g, using the urine microalbumin/urine creatinine ratio sustained for 90 days.
Secondary efficacy endpoint	Percent of subjects who have the same or reduced 5 year risk of ESRD and 2 year risk of ESRD at 12 and 18 months after the last injection.	Percent of subjects who have the same or reduced 5 year risk of ESRD and 2 year risk of ESRD at 12 and 18 months after the first injection.
Exploratory efficacy endpoints	The exploratory efficacy endpoints include the endpoints of incidence of changes, and time to increase the event of follows:<br>• Antihypertensive therapy<br>• Acidosis therapy<br>• Hyperkalemia therapy<br>• Anemia therapy<br>• Bone and mineral dysmetabolism	These endpoints were removed.
Exploratory efficacy endpoints	Change from baseline on following parameters:<br>• renal volume<br>• cortical thickness<br>• hemoglobin<br>• hematocrit<br>• calcium<br>• phosphorus<br>• parathyroid hormone<br>• systolic blood pressure<br>• diastolic blood pressure<br>• potassium<br>• bicarbonate	Change from baseline on following parameters<br>• blood hemoglobin<br>• blood hematocrit<br>• serum calcium<br>• serum phosphorus<br>• plasma parathyroid hormone<br>• serum potassium<br>• serum bicarbonate<br>• eGFR in 2009 CKD-EPI<br>• eGFR in 2012 CKD-EPI<br>• UACR

Changes	Protocol	SAP
Primay safety endpoints	Procedure and investigational product-related treatment-emergent adverse events (TEAEs) obtained through at least 18 months after the last REACT injection.	Percentage of participants with procedure and investigational product-related treatment-emergent adverse events (TEAEs).
Exploratory safety endpoints	Change from baseline on following parameters:<br>• renal volume<br>• cortical thickness<br>• hemoglobin<br>• hematocrit<br>• calcium<br>• phosphorus<br>• parathyroid hormone<br>• systolic blood pressure<br>• diastolic blood pressure<br>• potassium<br>• bicarbonate	The collected value and the change from baseline of follows are added in SAP:<br>• Physical examination<br>• Vital signs<br>• Safety laboratory analytes<br>• 12-lead ECG<br>• Renal volume<br>• Renal cortical thickness
Usage of ITT Analysis Set on sensitivity analysis	Sensitivity analysis apply on all efficacy endpoints if ITT analysis set is different than the mITT	Sensitivity analysis on ITT Analysis Set is removed from the SAP
Grouping of laboratory parameters	As in Protocol Table 13	As in SAP Table 23

8 GENERAL CONSIDERATIONS FOR DATA ANALYSIS

In general, all efficacy and safety variables will be summarized using descriptive statistics, as appropriate. Unless otherwise specified, continuous variables will be summarized by presenting the number of non-missing observations (n), mean, standard deviation (STD), median, minimum, and maximum. The mean and standard deviation will be excluded for laboratory summaries of non-symmetrically distributed results (e.g., UACR, urine protein to creatinine ratio); the non-missing observations (n), minimum, 25th percentile, median, 75th percentile, and maximum will be presented in these cases. Unless otherwise specified, for the summary statistics of all numerical variables, minimum and maximum will be displayed to the same level of precision as reported but no more than four decimal places. Mean, median, 25th and 75th percentiles will be displayed to one level of precision greater than the data collected; standard deviation will be displayed to two levels of precision greater than the data collected.

Categorical variables will be summarized by presenting frequency count and percentage for each category. Unless otherwise stated, the counts will consist of the number of participants in a particular category and the percentage of the total number of participants presented to one decimal place. If the count is 0, no percentage will be presented. If the percentage is 100%, the decimal place will be excluded (presented as "100%" instead of "100.0%"). Values of

percentages less than 0.1% will be presented as "<0.1%". Any rounding will be done after all calculations are made.

Summaries will be presented by treatment group Cohort 1, Cohort 2 and overall for all disposition, demographic and baseline data, and safety data; summaries will be presented by Cohort 1 and Cohort 2 on efficacy data.

The time to event endpoints will be summarized using the Kaplan-Meier (KM) method, including descriptive statistics of median, the lower and upper quartiles, and the 95% confidence interval (CI).

Participants' data will be listed, sorted by Cohort, participant ID, analytes/ tests by alphabetical order, and the sample collection date or visit date. All data and the non-imputed values/results will be presented in the listings.

8.1 Analysis Sets

Analysis Sets defined in this study are in Table 3.

Table 3: Analysis Sets

Analysis Set	Definition	Use	How Analyzed
Intent-to-Treat (ITT)	All participants randomized.	Demographic and screening characteristics summaries	According to randomized cohort assigned
Modified Intent-to-Treat (mITT)	All participants randomized who received at least one REACT injection.	All efficacy analyses – as main analysis set	According to randomized cohort assigned
Safety Set (SAF)	All participants randomized who received at least one REACT injection.	Safety analyses	According to actual cohort received
Biopsied Set (BS)	All participants who received a biopsy.	AE summaries	According to actual cohort received for participants who received REACT injection, and to randomized cohort assigned for participants who only received a biopsy but not REACT injection.

8.2 Method for Handling Missing Data

There will be no imputation of incomplete or missing data unless otherwise specified as follows.

8.2.1 Partial or Missing Concomitant Medication (CM), Concomitant Procedures (CP), and Adverse Event (AE) Date Handling

For inclusion in prior and/or concomitant medications, procedures, and adverse events, missing or incomplete medication, procedure, and AE start and stop dates will be imputed as follows:

Start Dates (where UK, UNK and UNKN indicate unknown or missing day, month or year respectively):

- UK-MMM-YYYY:
  - If the month and year are different from the month and year of the first REACT injection date, assume 01-MMM-YYYY.
  - If the month and year are the same as the first REACT injection date month and year, then impute start date as the first REACT injection date or end date of medication/adverse event (whichever occurred first).
- DD-UNK-YYYY/UK-UNK-YYYY:
  - If the year is different from the year of the first REACT injection date, assume 01-JAN-YYYY of the collected year.
  - If the year is the same as the first REACT injection date year, then impute start date as the first REACT injection date or end date of medication/adverse event (whichever occurred first).
- DD-MMM-UNKN or completely missing start date UN-UNK-UNKN:
  - If the year is missing, or if the start date is completely missing, impute the start date as the first REACT injection date or end date of medication/adverse event (whichever occurred first).

Stop Dates (where UK, UNK and UNKN indicate unknown or missing day, month and year respectively):

- UK-MMM-YYYY: Assume the last day of the month.
- DD-UNK-YYYY/UK-UNK-YYYY: Assume 31-DEC-YYYY, or data cutoff/database lock date, whichever is earliest.
  - Exception: if the event is marked as not ongoing and the date of discontinuation or death is before 31-DEC-YYYY, then the end date will be set to the date of discontinuation or death.
  - Completely missing stop dates will not be imputed.

The imputed dates will be used to determine if the AE is treatment-emergent, or the medication, or procedure is prior or concomitant.

In the situation where the concomitant medication, or procedure, and/or adverse event date is partial or missing, the date will be presented as is (partial or missing) in listings. Study day and any corresponding duration will be presented as missing.

8.2.2 Algorithm for Partial or Missing Event Dates for Time to Event Analyses

In the case of partial or missing dates for time to event analyses, all efforts will be made to query the site and obtain the missing information. In the case of partial or missing dates, then the median imputation rule will be used. For example, if the day is missing and the month is July then Day 16 is chosen. If the number of potential values is even, the lower of the 2 middle numbers is taken.

If both a non-fatal event and death have partially missing dates, then non-fatal event takes precedence and will be imputed as defined.

8.2.3 Algorithm for Missing Adverse Event Information

If the grade is missing, then the adverse event is assumed to be "**Grade 3**" or as appropriate following CTCAE grading for analysis purposes.

If the relationship to biopsy, REACT injection(s), or REACT product is missing, then the adverse event is assumed to be "**related**" for analysis purposes unless the AE start date is complete and is prior to the biopsy and/or injection(s), in which "**not related**" will be assumed for analysis purposes.

8.3 Derivations

8.3.1 Reference Date and Study Day

The date of the first injection is the reference date. Study day will be calculated in reference to the date of the first injection. Study Day 1 will be the day of the first REACT injection. There is no Study Day 0. Study day for the day of biopsy will be a negative value. That is, study day is not defined for participants who do not receive a REACT injection.

For assessments/events on or after the first injection date, then:

Study Day = (date of assessment/event – date of first injection) + 1.

For assessments/events before the first injection date, then:

Study Day = (date of assessment/event – the date of first injection).

8.3.2 Baseline Definition

Unless otherwise specified, the baseline value is defined as the latest non-missing value collected prior to the first REACT injection, excluding Post Procedure Biopsy Day.

Vital signs are taken throughout the procedure during biopsy and REACT injections. As peri-procedure vital signs (not including weight, height, BMI, and temperature) will be impacted by the procedure (e.g., sedation), the baseline value for these vital signs will be defined as the last non-missing value taken on a date prior to the date of first REACT injection, excluding Biopsy Day.

Blood pressure measurements are taken in triplicate at Screening; therefore, the average of the triplicate values will be used for baseline identification.

The baseline used for efficacy analyses on eGFR using CKD-EPI 2009 Equation and UACR are defined as the average of measurements collected within 21 days prior to the first REACT injection, excluding the measurement collected on Biopsy Day post procedure.

8.3.3 Analysis Periods

The following analysis periods will be defined as follows and as shown in Figure 2.
Pre-Biopsy Period: from the date of randomization up to but not including the date of biopsy.

- **Pre-Injection Period**: from the start day of biopsy up to but not including the date of the first REACT injection.
- **Post-Injection Period**: from the date of the first REACT injection, post-injection, to the date of study withdrawal or completion.

Figure 2: Analysis Period Diagram

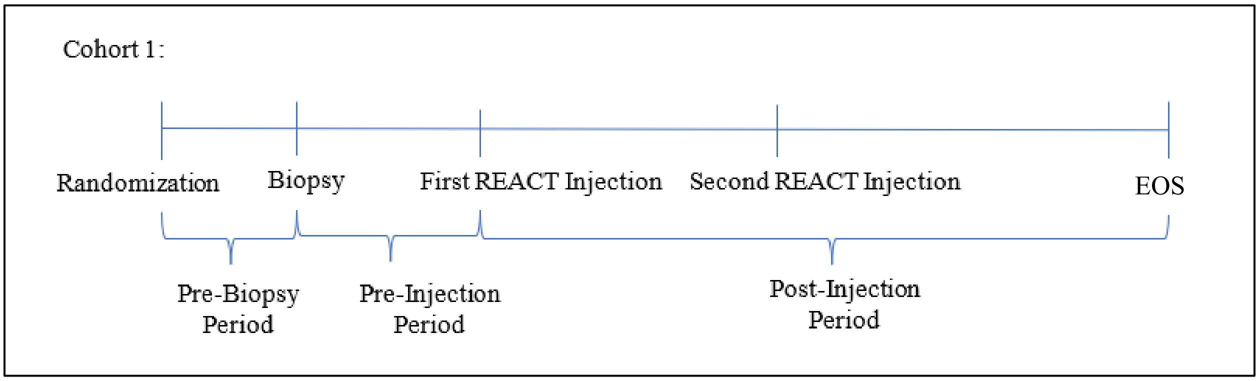

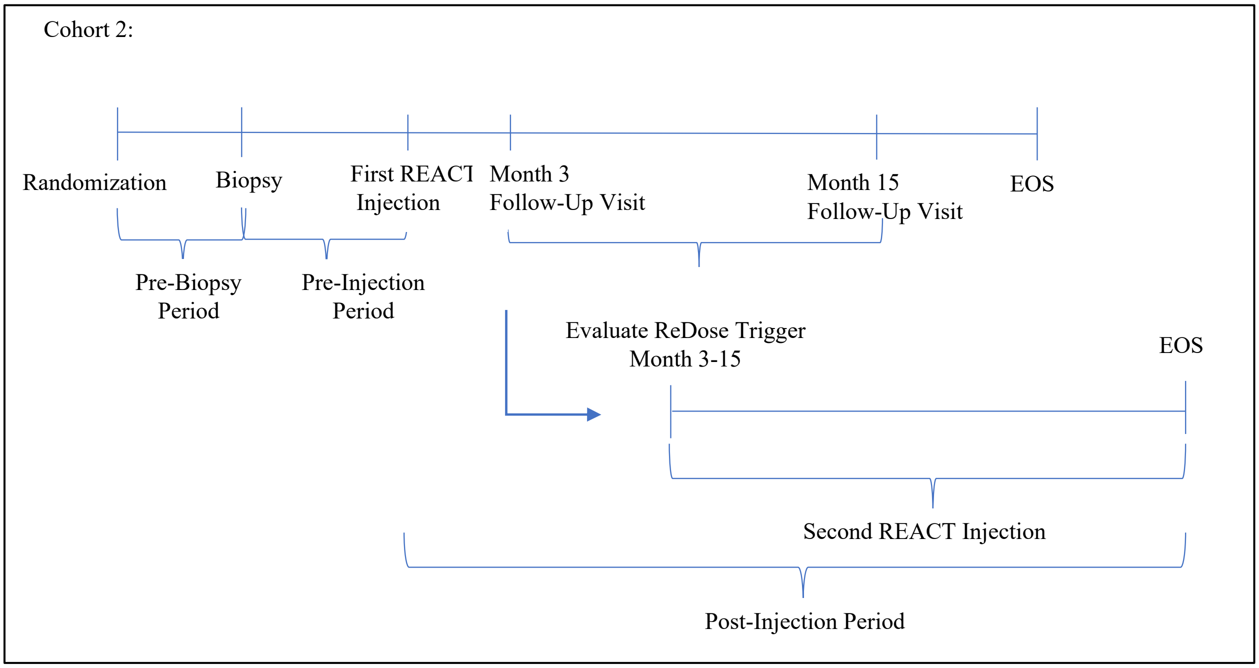

Abbreviations: EOS = End of Study

8.3.4 Common Derivations

- A month = 30.4375 days
- A year = 365.25 days
- Change from baseline = value – baseline value
- Relative change from baseline (also known as Percent change from baseline) = [(value – baseline value) / baseline value] * 100
- Age in years at informed consent is calculated by the EDC (Electronic Data Capture) as the difference between the date of the informed consent and the date of birth.
- Body mass index (BMI) (kg/ m^2) is calculated by EDC as weight (kg)/ height $(m)^2$
- Time to event (months) = (in days, date of event or censoring – date of first REACT injection + 1) / 30.4375; calculated to 2 decimal places.

8.3.5 Derivation of Length of Follow-Up

For participants who are still ongoing or discontinued with a reason of 'Lost to Follow-Up', the total length of follow-up will be defined as the time, in months, between the randomization date and the latest of: the date of the last study visit where an assessment occurred (e.g. laboratory results, vital signs, physical exam), date of death, or AE or concomitant medication start or end dates. For all other participants, the total length of follow-up will be defined as the time in

months between the randomization date and the date of completion or discontinuation from the End of Study CRF. The total length of follow-up will be calculated to 2 decimal places.

8.3.6 Derivation of eGFR

For all analyses, eGFR will be reported in mL/min/1.73m² using the 2009 equation: 2009 CKD-EPI serum creatinine equation. The on study eGFR measurements using the 2009 equation will be provided from the central laboratory. The historical eGFR using the 2009 equation collected from the Medical History of CKD Progression Form will be used for analyses, if the eGFR is collected by using other methods, then the result will be converted using 2009 equation as follows.

If serum creatinine is collected in Medical History of CKD Progression Form, then the eGFR will be derived using 2009 CKD-EPI serum creatinine equation as below.

- eGFR = 141 × min (serum creatinine (SCr)/κ, 1)$^{\alpha}$ × max (SCr/κ, 1)$^{-1.209}$ × 0.993^{Age} × 1.018 [if female] × 1.159 [if African American] rounded to the nearest whole number.

Where:

- eGFR unit is mL/min/1.73 m2
- SCr unit is mg/dL
- κ = [0.7 females] or [0.9 males]
- α = [-0. 329 females] or [-0. 411 males]
- min (SCr/κ, 1) indicates the minimum of SCr/κ or 1
- max (SCr/κ, 1) indicates the maximum of SCr/κ or 1
- age at collection unit is years.

If the historical eGFR is collected using Modification of Diet in Renal Disease (MDRD) equation, then the serum creatinine will be calculated by below equation, then derive the eGFR using above the 2009 CKD-EPI serum creatinine equation.

Modification of Diet in Renal Disease (MDRD) equation: eGFR = 175 × (SCr)$^{-1.154}$ × (age)$^{-0.203}$ × 0.742 [if female] ×1.212 [if African American]

SCr = exp (log ((eGFR collected using MDRD) / 175 × (age)$^{-0.203}$ × 0.742 [if female] ×1.212 [if African American]) / (-1.154))

If the historical eGFR is collected using other equations or the equation is unknown, then the historical data will not be used for analysis.

8.3.7 Derivation of UACR

Urine microalbumin to creatinine ratio (UACR) will be reported in mg/g from central laboratory. The UACR value from morning void urine sample will be used for analyses, if available. If for a certain visit, the morning void UACR result is not available, then the UACR value from random urine sample will be taken for analysis. If both morning void and random sample are not available, then the UACR will be calculated from Urine Albumin/Creatinine when it is applicable.

8.3.8 Stage of Chronic Kidney Disease (eGFR and Albuminuria Category)

The stage of CKD is classified based on eGFR category and albuminuria category; both will be calculated at screening and baseline. eGFR category is derived by using the 2009 CKD-EPI serum creatinine equation and albuminuria category is derived using urine albumin to urine creatinine ratio according to Kidney Disease Improving Global Outcomes (KDIGO) guidelines[1]. The CKD is categorized as below in Table 4 and Table 5.

Table 4: Categorization of eGFR

eGFR Category	eGFR (ml/min/1.73m^2)	Terms
G1	≥ 90	Normal or high
G2	60-89	Mildly decreased
G3a	45-59	Mildly to moderately decreased
G3b	30-44	Moderately to severely decreased
G4	15-29	Severely decreased
G5	<15	Kidney failure

Table 5: Categorization of Albuminuria (Urinary Albumin/Creatinine Ratio)

Albuminuria Category	UACR (mg/g)	UACR (mg/mmol)	Terms
A1	<30	<3.39	Normal to mildly increased
A2	30-300	3.39-33.9	Moderately increased
A3	>300	>33.9	Severely increased

8.3.9 Derivation of 5-year and 2-year Risk of ESRD

The 5-year and 2-year risk of ESRD will be calculated based on the 8-variable Kidney Failure Risk Equation[2]. The risk will be calculated as a percentage; the value will be reported to 1 decimal place.

- Kidney Failure Risk Equation 8-variable equation, Patient 5-year risk:

[1 – 0.9301^ exp (-0.1992 × (age/10 – 7.036) + 0.1602 × (male – 0.5642) – 0.4919 × (eGFR/5 – 7.222) + 0.3364 × (log(UACR) – 5.137) – 0.3441 × (albumin – 3.997) + 0.2604 × (phosphorous – 3.916) – 0.07354 × (bicarbonate – 25.57) – 0.2228 × (calcium – 9.355))] x 100.

- Kidney Failure Risk Equation 8-variable equation, Patient 2-year risk:

[1 – 0.9780 ^ exp (-0.1992 × (age/10 – 7.036) + 0.1602 × (male – 0.5642) – 0.4919 × (eGFR/5 – 7.222) + 0.3364 × (log(UACR) – 5.137) – 0.3441 × (albumin – 3.997) + 0.2604 × (phosphorous – 3.916) – 0.07354 × (bicarbonate – 25.57) – 0.2228 × (calcium – 9.355))] x 100.

Where:

- Age unit is years
- Male=1 for male participants; 0 for female participants
- All laboratory values are reported in conventional units
- eGFR is in mL/min/1.73 m^2
- log(UACR) = natural of UACR in mg/g
- albumin is in g/dL
- phosphorous is in mg/dL
- bicarbonate is in mEq/L
- calcium is in mg/dL.

8.3.10 Derivation of Incidence and Time from First REACT Injection to at Least 40% Reduction in eGFR

Time from the first REACT injection to at least 40% reduction in eGFR is defined as the time, in months, from the date of first REACT injection to at least 40% reduction in eGFR sustained for 30 days or censor date, whichever occurs first. The start date is defined as the date of the first REACT injection. Missing values of eGFR are not considered in the confirmation of sustainment.

Table 6 provides a summary of the rules to be used for time to at least 40% reduction in eGFR. Censoring rules will be applied in a sequential order, moving from the top row to bottom row until a situation is met. The date of event or censoring for the first situation met will be selected.

Table 6: Time from First REACT Injection to at least 40% Reduction in eGFR

Situation	Date of Event or Censoring	Outcome
Participant received the first REACT injection and does not have a non-missing baseline value for eGFR.	Date of first REACT injection	Censored
Participant received the first REACT injection, chronic dialysis or renal transplantation is initiated when at least 40% reduction in eGFR criterion haven't been met.	Date of last available eGFR laboratory result prior to the initiation of dialysis or renal transplantation.	Censored
Participant received the first REACT injection and does not have any post-baseline laboratory results for eGFR.<br><br>OR<br><br>Participant received the first REACT injection and has post-baseline laboratory results eGFR, and none of the laboratory measurements meet the condition of 40% decline (including sustainment).	Date of the first REACT injection<br><br>OR<br><br>Date of the last available eGFR laboratory result	Censored
Participant received the first REACT injection, did not meet prior criteria, and had death due to any cause including the categorization of cardiovascular or renal.	Date of the last available eGFR laboratory result prior to death	Censored
Participant received the first REACT injection, did not meet prior criteria, and had no death.	Date of the last available eGFR laboratory result	Censored
Participant has non-missing baseline and post-baseline laboratory results for eGFR meets the condition of an event: 40% decline (including sustainment).	Date of the earliest eGFR laboratory result that meets the event condition. Event date will be the index date, not the sustainment date.	Event

8.3.11 Derivation of Incidence and Time from First REACT Injection to eGFR <15 mL/min/1.73m^2 or Renal Replacement Therapy (RRT)

Renal replacement therapy is defined as eGFR <15 mL/min/1.73m^2 sustained for 30 days and/or the initiation of chronic dialysis or renal transplantation. Time to RRT is defined as the time, in months, from the date of the first REACT injection to the date of eGFR decline to < 15 mL/min/1.73m^2, initiation of dialysis or transplant, or censor date, whichever occurs first. The start date is defined as the date of the first REACT injection. Missing values of eGFR are

not considered in the confirmation of sustainment. Table 7 provides a summary of the rules to be used for time to eGFR <15 mL/min/1.73m^2 or RRT.

Table 7: Time from First REACT Injection to eGFR <15 mL/min/1.73m^2 or RRT Definitions

Situation	Date of Event or Censoring	Outcome
Participant did not meet the criteria of < 15 mL/min/1.73m^2 prior to the 1st injection, received the first REACT injection, and does not have any post-baseline laboratory results for eGFR.<br><br>OR<br><br>Participant did not meet the criteria of < 15 mL/min/1.73m^2 prior to the 1st injection, received the first REACT injection, and has post-baseline laboratory results eGFR, and none of the laboratory measurements meet the condition of < 15 mL/min/1.73m^2 (including sustainment) and had no initiation of chronic dialysis or renal transplant.	Date of first REACT injection<br><br>OR<br><br>Date of the last available eGFR laboratory result	Censored
Participant received the first REACT injection, did not meet prior criteria, and had death due to any other cause including the categorization of cardiovascular or renal.	Date of the last available eGFR laboratory result prior to death	Censored
Participant received the first REACT injection, did not meet prior criteria, and had no death.	Date of the last available eGFR laboratory result	Censored
Participant received the first REACT injection and met laboratory result criteria for eGFR prior to first REACT injection, baseline.	Date of first REACT injection	Event
Participant received the first REACT injection and has non-missing baseline and post-baseline laboratory results for eGFR, and at least one of the results meets the condition for the event: < 15 mL/min/1.73m^2 (including sustainment).	Date of the earliest laboratory collection for eGFR that meets the event condition	Event
Chronic dialysis sustained for 30 days	Initiation of chronic dialysis	Event
Renal transplant	Date of renal transplant	Event

8.3.12 Derivation of Incidence and Time from First REACT Injection to at least 30% and 30 mg/g Increase in UACR Sustained for 90 Days

Time to 30% and 30 mg/g increase in UACR is defined as the time, in months, from the date of first REACT injection visit to 30% and 30 mg/g increase in UACR sustained for 90 days or censor date, whichever occurs first. The start date is defined as the date of the first REACT injection visit. Missing values of UACR are not considered in the confirmation of sustainment.

Table 8 provides a summary of the rules to be used for time to at least 30% and 30 mg/g increase in UACR. Censoring rules will be applied in a sequential order, moving from top row to bottom row until a Situation is met. The Date of Event or Censoring for the first Situation met will be selected.

Table 8: Time from First REACT Injection to at least 30% and 30 mg/g Increase in UACR

Situation	Date of Event or Censoring	Outcome
Participant received the first REACT injection and does not have a non-missing baseline value for UACR.	Date of first REACTinjection	Censored
Participant received the first REACT injection, dialysis or renal transplantation is initiated when at least 30% and 30 mg/g increase in UACR criterion haven't been met.	Date of last available UACR laboratory result prior to the initiation of dialysis or renal transplantation.	Censored
Participant received the first REACT injection and does not have any post-baseline laboratory results for UACR.<br><br>OR<br><br>Participant received the first REACT injection and has post-baseline laboratory results UACR, and none of the laboratory measurements meet the condition of 30% and 30 mg/g increase (including sustainment).	Date of first REACT injection<br><br>OR<br><br>Date of the last available UACR laboratory result	Censored
Participant received the first REACT injection, did not meet prior criteria, and had death due to any cause including the categorization of cardiovascular or renal.	Date of the last available UACR laboratory result prior to death	Censored
Participant received the first REACT injection, did not meet prior criteria, and had no death.	Date of the last available UACR laboratory result	Censored
Participant has non-missing baseline and post-baseline laboratory results for UACR meets the condition of an event: 30% and 30 mg/g increase (including sustainment).	Date of the earliest laboratory collection for 30% and 30 mg/g increase that meets the event condition. Event date will be the index date, not the sustainment date.	Event

8.3.13 Derivation of Incidence and Time from First REACT Injection to Renal or Cardiovascular Death

Cause of death defined as renal or cardiovascular as collected on the adverse event form. The time to renal or cardiovascular death is defined as the time, in months, from the reference start date to the date of renal or cardiovascular death. Table 9 provides a summary of the rules to be used for time to renal or cardiovascular death.

Table 9: Time from First REACT Injection to Renal or Cardiovascular Death Definitions

Situation	Date of Event or Censoring	Outcome
No death and lost-to-follow-up	Date of last visit where a clinical assessment was performed	Censored
No death and not lost-to-follow-up	Date of last visit (End of Study Visit for those known to be alive, or otherwise at the date last known to be alive [date of last visit where a phone visit or clinical assessment was performed such as in-clinic visit or laboratory assessment])	Censored
Death due to any other cause (other than renal or cardiovascular)	Date of death	Censored
Death with a cause of death categorized as renal	Date of death	Event
Death with a cause of death categorized as cardiovascular	Date of death	Event

8.3.14 Derivation of Incidence and Time from First REACT Injection to Four Composite Endpoint

This secondary efficacy endpoint is defined as the time from first injection to earliest of the following:

- At least 40% reduction in eGFR, using the 2009 CKD-EPI serum creatinine equation, sustained for 30 days or
- eGFR <15 mL/min/1.73m² using the 2009 CKD-EPI serum creatinine equation, sustained for 30 days and/or chronic dialysis, and/or renal transplant or
- Increase of UACR of at least 30% and of at least 30 mg/g, using the urine microalbumin/urine creatinine ratio sustained for 90 days or
- Renal or cardiovascular death.

The time to the four composite endpoint is defined as the time, in months, from the date of first REACT injection visit to the date of earliest event date, if no event occurred, then to the earliest censor date among these four endpoints. The start date is defined as the date of the first REACT injection visit. Missing values of eGFR or UACR are not considered in the confirmation of

sustainment. Table 10 provides a summary of the rules to be used for the four composite endpoint.

Table 10: Time from First REACT Injection to Four Composite Endpoint Definitions

Situation	Date of Event or Censoring	Outcome
Participant received the first REACT injection and does not have a non-missing baseline value for eGFR/UACR.	Date of first REACT injection	Censored
Participant received the first REACT injection and does not have any post-baseline laboratory results for eGFR/UACR.<br><br>OR<br><br>Participant received the first REACT injection and has post-baseline laboratory results eGFR/UACR, and none of the laboratory measurements meet either the conditions of 40% decline or < 15 mL/min/1.73m^2 or 30% increase (including sustainment).<br><br>OR<br><br>Had initiation of chronic dialysis or renal transplant.	Date of first REACT injection<br><br>OR<br><br>Date of last available eGFR laboratory result or last available UACR laboratory result, whichever occurred earlier	Censored
Participant received the first REACT injection, did not meet prior criteria, and had death due to any other cause without the categorization of cardiovascular or renal.	Date of last available eGFR laboratory result, or last available UACR laboratory result prior to death, whichever occurred earlier	Censored
Participant received the first REACT injection, did not meet prior criteria, and had no death.	Date of last available eGFR laboratory result, or last available UACR laboratory result,<br>whichever occurred earlier	---
Participant met laboratory result criteria (including sustainment) for eGFR < 15 mL/min/1.73m^2 prior to first REACT injection, baseline<br><br>OR<br><br>Participant met chronic dialysis criteria sustained for 30 days prior to first REACT injection.<br><br>OR<br><br>Participant met renal transplant criteria prior to first REACT injection.	Date of first REACT injection visit	Event
Participant has non-missing baseline and post-baseline laboratory results for eGFR/UACR, and at least one of the results meets one of the	Date of the earliest laboratory collection for eGFR/UACR that meets the event condition. Event date will be the index date, not the	Event

Situation	Date of Event or Censoring	Outcome
conditions of an event: 40% decline or < 15 mL/min/1.73m^2 or 30% increase (including sustainment).	sustainment date.	
Chronic dialysis sustained for 30 days	Initation of chronic dialysis	Event
Renal transplant	Date of renal transplant	Event
Participant death with a cause of death categorized as cardiovascular<br><br>OR<br><br>Participant death with a cause of death categorized as renal	Date of death	Event

8.3.15 Derivation of Incidence and Time from First REACT Injection to Three Composite Endpoint

This secondary efficacy endpoint is defined as the time from first injection to earliest of the following:

- Any 40% reduction in eGFR, using the 2009 CKD-EPI serum creatinine equation, sustained for 30 days or
- eGFR <15 mL/min/1.73m² using the 2009 CKD-EPI serum creatinine equation, sustained for 30 days and/or chronic dialysis, and/or renal transplant or
- Renal or cardiovascular death.

The time to the three composite endpoint is defined as the time, in months, from the date of first REACT injection visit to the date of earliest event date, if no event occurred, then to the earliest censor date among these three events. The start date is defined as the date of the first REACT injection visit. Missing values of eGFR are not considered in the confirmation of sustainment. Table 11 provides a summary of the rules to be used for the three composite endpoint.

Table 11: Time from First REACT Injection to Three Composite Endpoint Definitions

Situation	Date of Event or Censoring	Outcome
Participant received the first REACT injection and does not have a non-missing baseline value for eGFR.	Date of first REACT injection	Censored
Participant received the first REACT injection and does not have any post-baseline laboratory results for eGFR.<br><br>OR<br><br>Participant received the first REACT injection and has post-baseline laboratory results eGFR, and none of the laboratory measurements meet either the conditions of 40% decline or < 15 mL/min/1.73m^2 (including sustainment).	Date of first REACT injection<br><br>OR<br><br>Date of the last available eGFR laboratory result	Censored

Situation	Date of Event or Censoring	Outcome
OR<br>Had initiation of chronic dialysis or renal transplant.		
Participant received the first REACT injection, did not meet prior criteria, and had death due to any other cause without the categorization of cardiovascular or renal.	Date of the last available eGFR laboratory result prior to death	Censored
Participant received the first REACT injection, did not meet prior criteria, and had no death.	Date of the last available eGFR laboratory result	Censored
Participant met laboratory result criteria (including sustainment) for eGFR < 15 mL/min/1.73m^2 prior to first REACT injection, baseline<br><br>OR<br><br>Participant met chronic dialysis criteria sustained for 30 days prior to first REACT injection.<br><br>OR<br><br>Participant met renal transplant criteria prior to first REACT injection.	Date of first REACT injection visit	Event
Participant has non-missing baseline and post-baseline laboratory results for eGFR, and at least one of the results meets either the conditions of an event: 40% decline or < 15 mL/min/1.73m^2 (including sustainment).	Date of the earliest laboratory collection for eGFR that meets the event condition. Event date will be the index date, not the sustainment date.	Event
Chronic dialysis sustained for 30 days	Initation of chronic dialysis	Event
Renal transplant	Date of renal transplant	Event
Participant death with a cause of death categorized as cardiovascular<br><br>OR<br><br>Participant death with a cause of death categorized as renal	Date of death	Event

8.3.16 Derivation of Incidence and Time from First REACT Injection to All-Cause Mortality

Time to all-cause mortality is defined as the time, in months, from the date of the first REACT injection to the date of death from any cause. Table 12 provides a summary of the rules to be used for time to all-cause mortality.

Table 12: Time from First REACT Injection to All-Cause Mortality Definitions

Situation	Date of Event or Censoring	Outcome
No death and lost-to-follow-up	Date of last visit where a clinical assessment was performed (i.e. participant was seen in-clinic and laboratory assessments, vital signs, or other clinical assessments were performed).	Censored
No death and not lost-to-follow-up	Date of last visit (End of Study Visit for those known to be alive, or otherwise at the date last known to be alive [date of last visit where a phone visit or clinical assessment was performed such as in-clinic visit or laboratory assessment])	Censored
Death from any cause	Date of death	Event

8.4 Site Pooling Strategies

This is a multi-center study, with 5 sites participating in the study. All tabulations will be based on pooled data across all sites.

8.5 Visit and Unscheduled Visits

For by visit summaries, data recorded at the nominal visit will be presented. For the measurements that are scheduled multiple times at certain visits, e.g. Vital signs, ECG, the average value will be used for analyses.

Unscheduled measurements will not be included in by visit summaries but will be included in the minimum and maximum post-baseline summaries and will contribute to the values where applicable in shift tables. Unscheduled visits will be included in the listings. Unscheduled visits will also be considered when deriving the baseline value.

8.6 Windowing Conventions

For the analysis which refers to the 1st injection (eg.5-year and 2-year risk of ESRD at 12 months and 18 months after first injection, and the exploratory laboratory endpoints), the visits post the 2nd injection will be mapped to the analysis visits referencing the 1st injection as follows.

For Cohort 1 participants who receive two scheduled REACT injections given 3 months apart, the visits post the 2nd injection will be mapped to the analysis visits as Table 13. For the visits conducted prior to the 2nd injection or are not listed in Table 13, the analyses will be conducted based on the collected visits. For the Cohort 1 participant who doesn't receive the 2nd injection, the collected visits terminology will be standardized to be consistent with the analysis visit in Table 13.

Table 13: Analysis Visit Window Mapping for Cohort 1 with two REACT Injections

Collected Visit	Analysis Visit Referring 1st REACT Injection
INJ 1 Month 03 FU	INJ 1 Month 03 FU
INJ 2 Month 03 FU	INJ 1 Month 06 FU
INJ 2 Month 06 FU	INJ 1 Month 09 FU
INJ 2 Month 09 FU	INJ 1 Month 12 FU
INJ 2 Month 12 FU	INJ 1 Month 15 FU
INJ 2 Month 15 FU	INJ 1 Month 18 FU
EOS/Month 18	INJ 1 Month 21 FU

For Cohort 2 participants, whose timing of the second REACT injection depends upon when meeting a redose trigger, Analysis Window will be calculated based on the study day by referring to Table 14. For the visits conducted prior to the 2nd injection, the analyses will be conducted based on the collected visits.

Table 14: Analysis Visit Window Adjustment for Cohort 2

Analysis Visit Referring 1st Injection	Target Day Post the 1st REACT Injection (Study Day)	Analysis Visit Window (Study Day)
INJ 1 Month 03 FU	91	36 -137
INJ 1 Month 06 FU	182	138-228
INJ 1 Month 09 FU	273	229-319
INJ 1 Month 12 FU	365	320-411
INJ 1 Month 15 FU	456	412-502
INJ 1 Month 18 FU	547	503-593
INJ 1 Month 21 FU	639	594-685
INJ 1 Month 24 FU	731	686-777
INJ 1 Month 27 FU	822	778-868
INJ 1 Month 30 FU	913	869-959
INJ 1 Month 33 FU	1004	960-1050
INJ 1 Month 36 FU	1096	1051-1142

For presentations of data over time at each analysis visit, windows around assessment times are based on the midpoint between the adjusted analysis study visits. Study days corresponding to measurements are calculated as Section 8.3.1.

Unless otherwise stated, all other analyses except the above cases will use the analysis windows as planned for the study regarding data collection, i.e.. all other data will be analyzed and presented based on the visit as it is recorded in the database.

8.7 Laboratory Data

Unless otherwise specified, only the data provided by the central laboratory will be used for analysis. All laboratory data will be presented in Conventional Units, and ADaM datasets may contain both Conventional and International System of Units (SI), unless otherwise specified.

For results that are reported as above or below the limit of quantification (LOQ) (<LLOQ or >ULOQ), the numeric results will be converted following the rules in Table 15.

Table 15: Character to Numeric Conversion for LOQ results

Limit of Quantification	Condition	Conversion Rule
<LLOQ	LLOQ value is less than 2	LLOQ * 0.99
	LLOQ value is greater than or equal to 2	LLOQ - 1
>ULOQ	ULOQ value is less than 2	ULOQ * 1.01
	ULOQ value is greater than or equal to 2	ULOQ + 1

Laboratory abnormalities will be defined using the National Cancer Institute (NCI) Common Terminology Criteria for Adverse Events (CTCAE) v5.0 grading scheme[3]. Abnormal laboratory values will be flagged as above or below the normal range.

Results will be reported by category for safety analyses. For efficacy analyses, measurements taken on the day of biopsy or injection post the procedure, or measurements taken after a kidney transplant, or the initiation of dialysis will be removed from the summaries. Listings of laboratory assessments will include all measurements taken on study to be used for both safety and efficacy analyses; a notation for assessments taken after a kidney transplant or the initiation of dialysis will be included in the listings.

8.8 Statistical Tests

Statistical analyses will be primarily descriptive in nature, and no formal statistical hypothesis testing is planned for the study.

8.9 Multiple Comparisons and Multiplicity

No adjustments for multiplicity will be made in this study.

8.10 Software Version

All efficacy and safety statistical analyses will be performed using SAS version 9.4 or higher (SAS, Cary, NC), except where other software may be deemed more appropriate.

8.11 Example Table Column Presentation

Example column presentation may change over the life cycle of table development. Adjustments to the table layout will not necessitate a SAP amendment.

8.11.1 Outputs for 14.1.X Demographic Data

Overall summary of participant disposition, demographics and screening characteristics, and medical history.

	Cohort 1 (N=xxx)	Cohort 2 (N=xxx)	Overall (N=xxx)

Prior medications

	Cohort 1 (N=xxx)			Cohort 2 (N=xxx)			Total (N=xxx)		
	Pre-Injection Period (N1=xx)	Post-Injection Period (N2=xx)	Overall (N=xx)	Pre-Injection Period (N1=xx)	Post-Injection Period (N2=xx)	Overall (N=xx)	Pre-Injection Period (N1=xx)	Post-Injection Period (N2=xx)	Overall (N=xx)

N1 = Number of participants who received a biopsy.
N2 = Number of participants who received at least one REACT injection.

8.11.2 Outputs for 14.2.X Efficacy Data

	Cohort 1 (N=xxx)	Cohort 2 (N=xxx)

8.11.3 Outputs for 14.3.X Safety Data

By visit numeric and other categorical summaries:

	Cohort 1 (N=xxx)	Cohort 2 (N=xxx)	Overall (N=xxx)

AE overall summaries by analysis period:

	Cohort 1			Cohort 2			Overall		
	Pre-Biopsy Period (N1=xx)	Pre-Injection Period (N1=xx)	Post-Injection Period (N2=xx)	Pre-Biopsy Period (N1=xx)	Pre-Injection Period (N1=xx)	Post-Injection Period (N2=xx)	Pre-Biopsy Period (N1=xx)	Pre-Injection Period (N1=xx)	Post-Injection Period (N2=xx)

N1 = Number of participants who received a biopsy.
N2 = Number of participants who received at least one REACT injection.

9 EXAMINATION OF SUBGROUPS

Subgroup analyses may be conducted in an exploratory manner for both efficacy and safety summaries. All subgroup analyses will be predefined prior to database lock. Below subgroup categorization may be re-evaluated to align with the data collected during the study.

- Number of injections received (1 injection, 2 injections)
- CKD stage at Baseline (G3a, G3b, G4)
- Diabetes mellitus type (I, II)
- Glucagon-like peptide-1 (GLP-1) use at baseline (Yes, No)
- Non-steroidal mineralocorticoid (nsMRA) use at Baseline (Yes, No)
- Body mass index (BMI) (kg/m^2) (< 30, ≥30)
- HbA1c at baseline (%) (≤7.5, >7.5)
- Albuminuria category at Baseline (A2, A3).

Planned subgroup analyses may be planned for the following efficacy endpoints:

- The change from pre-injection to post-last injection total (acute + chronic) slope of eGFR using CKD-EPI 2009 serum creatinine equation
- Slope of eGFR, determined using CKD-EPI 2009, change from first injection to End of Study
- Time from the first REACT injection to three composite endpoint.

10 PARTICIPANT SUMMARY

10.1 Participant Disposition

A detailed description of participant disposition will be provided for all enrolled participants who signed the Informed Consent Form and will include the following.

- The number and percentage of participants screened, screen failures, randomized, randomized but not biopsied and not treated, randomized and received biopsy but not treated, biopsied and treated, and number of participants with 1 or 2 REACT injections
- The number and percentage of participants in each of the study populations
- The number and percentage of participants who completed study treatment, and those who discontinued study treatment and the reason for treatment discontinuation.
- The number and percentage of participants who completed study, those who were discontinuation from the study, the reason discontinuation from the study and the stage of the study as participant discontinuation
- The number and percentage of participants who died and the reason for death (renal, cardiovascular, other)
- Summary of length of follow-up.

Listings will include randomization schedule assignment and actual treatment received, disposition (biopsy date/time, injection date/time(s), the reasons for treatment discontinuation, stage of discontinuation, study discontinuation reasons, and death summary), follow-up duration and analysis sets.

10.2 Protocol Deviations

Protocol deviations will be captured and categorized by type (important, non-important), category, sub-category, and protocol deviation term as appropriate.

Prior to the database lock, the Sponsor or designee will identify and review any deviations from the study protocol. Any protocol deviations that might significantly affect the completeness, accuracy, and/or reliability of the study data or that might significantly affect a participant's rights, safety, or well-being will be classified as important protocol deviations (FDA, ICH, 2013, January). Important protocol deviations will be defined prior to database lock for the final analysis.

Tabulation of the number and percentage of participants with important protocol deviations, and number of important protocol deviations by category and sub-category will be presented for the ITT Analysis Set. The categories used for the summary will be the categorizations determined in the clinical trial monitoring plan. Categories and sub-category within each category will be sorted by frequency from high to low. The important protocol deviation includes, but is not limited to the following:

- Deviations related to study inclusion or exclusion criteria
- Receipt of any prohibited therapies as defined in the protocol
- Significant deviations in investigational product administration and management
- Participants were not withdrawn after developing withdrawal criteria during the study.Failure to collect data to evaluate important study endpoints (e.g., primary or secondary endpoints)
- Failure to conduct study procedures designed to assess participant safety or failure to adequately monitor participants
- Failure to obtain informed consent or meet other applicable requirements under FDA regulations for the protection of human subjects under 21 CFR part 50
- Failure to protect a participant’s identifiable private protected health information

A listing of all protocol deviations will be present for ITT Analysis Set.

10.3 Demographic and Screening, Baseline Characteristics

Demographic and disease characteristics at both screening and baseline will be listed by participant and tabulated.

Demographic characteristics will include age at informed consent (years), age at the first injection, age groups (<65, >= 65), sex (male, female), childbearing potential (yes, no), race, and ethnicity. Additionally, the following measurements will be summarized using descriptive statistics for continuous variables and using number and percentage of participants for categorical variables. The measurements taken at screening visit will be summarized for ITT analysis set, the measurements taken at baseline will be summarized for mITT analysis set, if there are any cases in which the actual cohort is different from the planned cohort, then the measurements taken at baseline will be summarized for SAF.

- Type of diabetes (Type 1, Type 2)

  Under each Type 1 and Type 2 diabetes, below summary will be summrized:
  - Number of lines of medication used for diabetes treatment under each Type 1 and Type 2 diabetes (1, 2, 3, ≥4). The number of lines of medication will be counted by category of the medications used for diabetes treatment, which include Sodium-Glucose Cotransporter-2 (SGLT2) Inhibitors, Glucagon-Like Peptide-1 (GLP-1) agonist, Dipeptidyl Peptidase IV (DPP-4) Inhibitors, Biguanides, Sulfonylureas, Thiazolidinediones, Alpha Glucosidase Inhibitors, Insulin, and Other Blood Glucose Lowering Drugs Excluding Insulins.
  - Usage of SGLT2i (Yes, No)
  - Usage of Angiotensin II Receptor Blockers (ARBs) and/or Angiotensin-converting enzyme inhibitors (ACEi) (Yes, No)
  - Usage of GLP-1 (Yes, No)
  - Usage of MRA or nsMRA (Yes, No)
  - Usage of SGLT2 Inhibitor and MRA
  - Usage of insulin (Yes, No). The Insulin will be futher categorized by Short-Acting, Intermediate-Acting, and Long-Acting.
- Time since Type 1 or Type 2 Diabetes Mellitus diagnosis (years)
- eGFR as continuous variable (mL/min/1.73m2) and in CKD stage (G3a, G3b, and G4)
- UACR as continuous variable (mg/g) and in albuminuria category (A1, A2, A3)
- Body mass index (BMI) (kg/m^2) and in category (< 30, ≥30 kg/m^2)
- Systolic blood pressure (mmHg)
- Diastolic blood pressure (mmHg)
- HbA1c (%) and in category (≤7.5, >7.5%)

- Serum calcium (mg/dL)
- Serum phosphate (mg/dL)
- Serum bicarbonate (mEq/L)
- Serum potassium (mEq/L)
- Intact parathyroid hormone (pg/mL)
- Hemoglobin (g/dL)
- Biopsied kidney volume (mL).

10.4 Medical History

All medical conditions will be classified by system organ class (SOC) and preferred term (PT) using the Medical Dictionary for Regulatory Activities (MedDRA) version 28.0 or later.

The number and percentage of participants of SAF Analysis Sets with each medical condition will be presented for each SOC and PT. Participants will only be counted once within a particular SOC (PT) even if they have multiple conditions/diseases in the same SOC (PT). The summary will be ordered by descending order of incidence of SOC and PT within each SOC, and then alphabetically for ties on the overall column.

A listing of all medical history will also be created.

10.5 Medical History of Chronic Kidney Disease

The medical history of CKD information will be collected at screening on type of diabetes, the test type, eGFR calculation equation used, the test date and result. If the eGFR result is collected from other equations rather than 2009 CKD-EPI serum creatinine equation, then the eGFR using 2009 CKD-EPI serum creatinine equation will also be calculated and listed by referring Section 8.3.6.

A listing of all the medical history of CKD will also be created for ITT Analysis set.

10.6 Study Intervention – Biopsy

All biopsy data will be listed for BS Analysis Set.

10.7 Study Treatment Exposure, Compliance and Relative Intensity - REACT Injections

The exposure of REACT injection will be summarized on the data below for SAF:

- Number and percentage of participants with 1 and 2 injections.

- Duration of treatment. The duration of treatment will be calculated by the date of end of study day - the date of the 1st REACT injection + 1.
- Received volume per injection (mL). The volume will be using volume of injection collected in EDC.
- Time from the first to second REACT injection. Time from the first to second REACT injection will be calculated by date of the second injection – date of the first injection + 1.
- Received volume per each of the two REACT injections (mL).

Details of REACT administration and the exposure calculation will be listed.

10.8 Redosing Trigger

The information collected from the Verification Committee for the redose trigger of Cohort 2 participants will be present in the listing. It will include the redose trigger criterion met, the start date trigger event occurred, and the date(s) of trigger sustainment occurred.

10.9 Prior and Concomitant Medications

Prior and concomitant medications will be coded using the World Health Organization (WHO) drug dictionary WHODrug Global Drug Dictionary B3 format, March 2025 release version or later.

A prior medication is defined as those medications which started prior to the first REACT injection. A concomitant medication is defined as those medications which have a start date on or after the first REACT injection or have a start date prior to the first REACT injection and have an end date after the first REACT injection. A medication can be both a prior and concomitant medication.

The number and percentage of participants using prior and concomitant medications will be tabulated for SAF by study period (only for prior medication), by Anatomical, Therapeutic, and Chemical (ATC) class level 2, ATC class 4, and by preferred term (PT). Participants taking the same medication multiple times will only be counted once for that particular ATC (PT). Each summary will be alphabetically ordered by ATC 2, ATC 4 within each ATC 2, and PT within each ATC 4 on overall column.

The medication with start day prior to Biopsy date will be counted to pre-biopsy period; the medication with start day prior to the first REACT injection and on or after the biopsy will be counted to pre-injection period; the medication with start day on and post the first REACT injection will be counted as the post-injection period.

A listing of all prior and concomitant medications recorded in the database will be provided for ITT Analysis Set.

10.9.1 Prior and Concomitant Medications of Special Interest

Additionally, there are several classes of medications that are of special interest as follows. The final list of special interest medications will be identified by medical review before DBL.

- Angiotensin-converting enzyme (ACE) inhibitors
- Angiotensin II receptor blockers (ARBs)
- Sodium-glucose cotransporter-2 (SGLT2) inhibitors
- Mineralocorticoid receptor antagonists (MRA)
- Other Antihypertensive drugs
- Other drugs for the treatment of diabetes
  - Insulins
  - Biguanides
  - Sulfonamides
  - Glucagon-like peptide-1(GLP-1) agonist
  - Thiazolidinediones
  - Alpha glucosidase inhibitors
  - Other blood glucose lowering drugs
  - Dipeptidyl Peptidase IV (DPP-4) inhibitors
- Other drugs for treatment of CKD
  - Hyperkalemia therapy
  - Hyperphosphatemia therapy
  - 2nd Hyperparathyroidism therapy
  - Antacids
  - Anemia Therapy
  - Diuretics
- Lipid modifying agents.

The data listing of prior and concomitant medications will identify whether each medication listed meets criteria for inclusion into one of the categories of special interest.

The number and percentage of participants using concomitant medications of special interest at baseline will be tabulated for SAF, by category of special interest, and by PT.

10.10 Prior and Concomitant Procedures

Prior and concomitant procedures will be coded using the Medical Dictionary for Regulatory Activities (MedDRA) version 28.0 or later.

A prior procedure is defined as procedures being conducted before the first REACT injection. Concomitant procedures are procedures taken on or after the first REACT injection. If the start and stop dates of the procedures do not clearly define the period during which a procedure was conducted, it will be assumed to be a concomitant procedure.

A summary of procedures will be presented as the number and percentage of participants by SOC and PT term for SAF. Participants having the same procedure multiple times will only be counted once for that SOC and PT. The summary will be ordered by descending order of incidence of SOC and PT term, and then alphabetically for ties on overall column.

A listing of all prior and concomitant procedures will also be created for ITT Analysis Set.

11 EFFICACY ANALYSIS

All efficacy analyses will use the mITT Analysis Set.

11.1 Primary Efficacy Endpoint and Analysis

The primary endpoint is defined as the change from pre-injection to post-last injection total (acute + chronic) slope of eGFR using CKD-EPI 2009 serum creatinine equation.

The pre-injection period includes both historical eGFR values collected in the Medical History of CKD Progression form, and the on study central laboratory eGFR results prior to first REACT injection, calculated using the CKD-EPI 2009 serum creatinine equation. Historical data collected more than 24 months before the Screening visit will be excluded from analyses. The on study central laboratory data for pre-injection period includes eGFR values collected from the Screening visit to the last visit prior to the first REACT injection. The last CKD-EPI 2009 eGFR result prior to the first REACT injection, obtained from either the historical date or central laboratory result, excluding the measurements taken on the biopsy day post procedure, will be taken as the baseline for the pre-injection period.

If historical eGFR values were originally calculated using an equation other than the 2009 CKD-EPI serum creatinine equation, they will be recalculated using the 2009 CKD-EPI method as

described in Section 8.3.6. When multiple historical eGFR values are available for the same day, the average of those results will be used for analysis.

The post-last injection period includes the visits from the last REACT injection to EOS visit, with the baseline of this period defined as the average of eGFR measurements collected within 21 days prior to the last injection, excluding the measurement collected on Injection Day post procedure.

For both analysis periods, unscheduled visits will be included; all measurements taken on the biopsy or injection day post the procedure, and all measurements taken after the start of dialysis and renal transplant will be excluded.

The estimated annualized slope for each study period and the slope difference of the two periods, as well as their 95% CI from linear mixed model, will be presented. Table 16 details the slopes of interest, the models to be fit, and the data to be included in each model. Likelihood-based comparisons of goodness of fit will be used to determine which model should fit in Table 16, and the model will only be reported if the fit is good. The difference will only be presented if the interaction term in Model 3 is significant. If the interaction term is not significant, the model will be uninterpretable due to overfit and the comparation of eGFR slope during the two study periods will not be reported, and eGFR slope for each study period will be estimated from the Model 1 and Model 2 as in Table 16. If the interaction term is significant, then the eGFR slope for each study period will be estimated from the main effect of Model 3 as in Table 16.

The following covariance structures will be tried in the order listed below (decreasing number of parameters) to fit the models outlined in Table 16 until one of them achieves model convergence:

1. Unstructured (UN)
2. First-order autoregressive moving average (ARMA(1,1))
3. First-order autoregressive (AR(1))
4. Compound symmetry (CS)
5. Variance Components (VC).

Table 16: Models used to Estimate Slopes of eGFR Change from Pre-injection to Post-last Injection

Slope of Interest	Model	Data to be Included in Model
Model 1: The eGFR slope in pre-injection period. (will only be applied when the interaction term in Model 3 is not significant)	$Y_{ij} = \beta_0 + \beta_1(BL_i) + \beta_2(t_{ij}) + b_{0i} + b_{1i}(t_{ij}) + \varepsilon_{ij}$<br>Where<br>$Y_{ij}$ is the eGFR change from baseline as estimated by the 2009 CKD-EPI serum creatinine equation<br>BL_i is the baseline eGFR value as estimated by the 2009 CKD-EPI serum creatinine equation in this period<br>t_{ij} is the number of days from baselinedivided by 365.25<br>$\beta_.$ indicates fixed effects<br>$b_{.i}$ indicates random effects<br>β_2 is the slope of interest	• mITT Analysis Set<br>• Pre-injection period measurements include historical eGFR and on study eGFR measurements taken prior to the 1st REACT injection<br>• Includes unscheduled visits<br>• Includes only the last measurement if there was a retest on the same day for the on study central laboratory eGFR and take the average value if there are multiple eGFR collection on the same day for the historical eGFR.<br>• Excluding measurements taken on the biopsy day post procedure.
Model 2: The eGFR slope in post-last injection period. (will only be applied when the interaction term in Model 3 is not significant)	$Y_{ij} = \beta_0 + \beta_1(BL_i) + \beta_2(t_{ij}) + b_{0i} + b_{1i}(t_{ij}) + \varepsilon_{ij}$<br>Where<br>$Y_{ij}$ is the eGFR change from baseline as estimated by the 2009 CKD-EPI serum creatinine equation<br>BL_i is the baseline eGFR value as estimated by the 2009 CKD-EPI serum creatinine equation<br>t_{ij} is the number of days from last injection divided by 365.25<br>$\beta_.$ indicates fixed effects<br>$b_{.i}$ indicates random effects<br>β_2 is the slope of interest	• mITT Analysis Set<br>• On study post-last injection period measurements through end of study<br>• Includes unscheduled visits<br>• Includes only the last measurement if there was a retest on the same day.<br>• Excluding measurements taken on the first or second injection day post procedure.<br>• Excluding measurements taken after the start of dialysis or renal transplantation.
Model 3: the eGFR slope for each study period and the slope difference during the two study periods (pre-injection period vs. post-last injection period) (will be	$Y_{ij} = \beta_0 + \beta_1(BL_i) + \beta_2(t_{ij}) + \beta_3(s_{ij}) + \beta_4(t_{ij} * s_{ij}) + b_{0i} + b_{1i}(t_{ij}) + \varepsilon_{ij}$<br>Where	• mITT Analysis Set<br>• On two study periods: pre-injection study period includes historical eGFR and on study eGFR measurement

Slope of Interest	Model	Data to be Included in Model
applied when the interaction term is significant)	Y_{ij} is the eGFR change from baseline as estimated by the 2009 CKD-EPI serum creatinine equation<br>BL_i is the baseline eGFR value as estimated by the 2009 CKD-EPI serum creatinine equation<br>t_{ij} is the number of days from last injection for the post-last injection period, or the number of days from baseline for the pre-injection period, divided by 365.25<br>s_{ij} indicates the study period<br>$\beta_{.}$ indicates fixed effects<br>$b_{.i}$ indicates random effects<br>β_4 is the slope of interest	taken prior to the 1st REACT injection.<br>• post-last injection period includes measurements from the last injection through end of study<br>• Includes unscheduled visits<br>• Includes only the last measurement if there was a retest on the same day for the on study central laboratory eGFR and take the average value if there are multiple eGFR collection on the same day for the historical eGFR.<br>• Excluding measurements taken on the biopsy or the first or second injection day post procedure.<br>• Excluding measurements taken after the start of dialysis or renal transplantation.

Scatter plots with regression line fit to the eGFR change from baseline in pre-injection period and post-last injection period will be provided, with time on the x-axis and the eGFR change from baseline on the y-axis.

11.2 Secondary Efficacy Endpoints and Analyses

The secondary endpoints are listed in Section 3. Secondary efficacy analyses will be performed on mITT analysis sets, unless otherwise specified.

11.2.1 Slope of Estimated Glomerular Filtration Rates Change from Baseline

Slope of eGFR, determined using CKD-EPI 2009, change from first injection to EOS will be analyzed using a linear mixed model.

Table 17 details the models to be fit to estimate the slope of interest, and the data to be included in the model.

The estimated annualized slope of eGFR and the 95% confidence interval will be provided for each cohort. The annualized slope of eGFR as estimated by the models outlined in Table 17 will also be presented in figures as detailed in Section 11.1.

Table 17: Model used to Estimate Slope of Estimated Glomerular Filtration Rates Change from Baseline

Slope of Interest	Model	Data to be Included in Model
The eGFR slope in post-first injection period	$Y_{ij} = \beta_0 + \beta_1(BL_i) + \beta_2(t_{ij}) + b_{0i} + b_{1i}(t_{ij}) + \varepsilon_{ij}$<br>Where<br>$Y_{ij}$ is the observed eGFR change from baseline as estimated by the 2009 CKD-EPI serum creatinine equation<br>BL_i is the baseline eGFR defined as the average of measurements collected within 21 days prior to the first injection, excluding the measurement collected on Biopsy Day post procedure.<br>t_{ij} is the number of days from first injection divided by 365.25<br>$\beta_{.}$ indicates fixed effects<br>$b_{.i}$ indicates random effects<br>β_2 is the slope of interest	• mITT Analysis Set<br>• On study post-injection period measurements from the 1st REACT injection through end of study<br>• Includes unscheduled visits<br>• Includes only the last measurement if there was a retest on the same day.<br>• Excluding measurements taken on the biopsy day post procedure, and the first or second injection post procedure.<br>• Excluding measurements taken after the start of dialysis or renal transplantation.

A set of subgroup analyses as described in Section 9 will be conducted for supportive analyses of this endpoint.

In addition, a supplemental analysis will be conducted to estimate the change from pre-injection to post-first injection total (acute + chronic) slope of eGFR using CKD-EPI 2009 serum creatinine equation. The estimated annualized slope for each study period and the slope difference of the two periods as well as their 95% CI from linear mixed model will be analyzed in a similar fashion as the primary efficacy endpoint as detailed in Section 11.1, with the model defined in Table 18. If the interaction term is not significant, the comparison of eGFR slope during the two study periods will not be reported, and the supplemental analysis will not be applied.

Table 18: Models used to Estimate Slopes of eGFR Change from Pre-injection to Post-First Injection

Slope of Interest	Model	Data to be Included in Model
The eGFR slope for each study period and the slope difference during the two study periods (pre-injection period vs. post-first injection period)	$Y_{ij} = \beta_0 + \beta_1(BL_i) + \beta_2(t_{ij}) + \beta_3(s_{ij}) + \beta_4(t_{ij} * s_{ij}) + b_{0i} + b_{1i}(t_{ij}) + \varepsilon_{ij}$<br>Where<br>$Y_{ij}$ is the eGFR change from baseline as estimated by the 2009 CKD-EPI serum creatinine equation<br>BL_i is the baseline eGFR value as estimated by the 2009 CKD-EPI serum creatinine equation<br>○ BL_i for the pre-injection period is defined as the last measurement prior to the first REACT injection collected in the historical and central laboratory eGFR, excluding the measurements taken on the biopsy day post procedure<br>○ BL_i for the post-first injection period is defined as the average of measurements collected within 21 days prior to the first injection, excluding the measurement collected on Biopsy Day post procedure<br>t_{ij} is the number of days from the first injection for the post-first injection period, or the number of days from baseline for the pre-injection period, divided by 365.25<br>s_{ij} indicates the study period<br>$\beta_.$ indicates fixed effects<br>$b_{.i}$ indicates random effects<br>β_4 is the slope of interest	• mITT Analysis Set<br>• On two study periods: pre-injection study period includes historical eGFR and the on study eGFR from screening to the last measurement taken prior to the 1st REACT injection<br>• post-first injection period includes measurements from the first injection through end of study<br>• Includes unscheduled visits<br>• If there are multiple eGFR measurements taken on the same day, then include only the last measurement for the on study central laboratory eGFR, and the mean value for the historical eGFR Excluding measurements taken on the same day of biopsy or injection post the procedure.<br>• Excluding measurements taken after the start of dialysis or renal transplantation.

11.2.2 5-year and 2-year risk of ESRD at 12 months and 18 months after first injection

The 5-year and 2-year risk of ESRD indicates the 5-year and 2-year probability of treated kidney failure, the lower risk indicates the better prognosis. It will be derived by referring to Section 8.3.9.

For the risk of ESRD at 12 months after first injection, the number and percentage of participants with same or reduced 5-year and 2-year ESRD risk at 12 months post-first injection versus baseline will be summarized. The risk of ESRD at 18 months after the first injection will be calculated in the same manner as the analysis at 12 months. Additionally, the 5-year and 2-year risk of ESRD and changes from baseline will be summarized descriptively by analysis visits

(Month 3, 6, 9 ,12, 15, 18, etc) post-first injection. The visit windowing convention will be conducted by referring to Section 8.6.

All measurements taken after the start of dialysis and renal transplant will be excluded from analysis. If more than 1 assessment falls into the same analysis visit window, then the one that is closer to the target day will be used. If 2 assessments within the same analysis visit window are equidistant from the target day, then the analysis will use the later assessment.

The 5-year and 2-year risk of ESRD on each visit will also be presented using box plots to present mean, median, first and third quantiles, the upper boundary defined as third quartile plus 1.5 times the interquartile range (third quantile – first quantile), the lower boundary defined as first quartile minus 1.5 times the interquartile range, and the outliers which fall significantly outside the boundaries that are 1.5 times the interquartile range. The box plot will be present from baseline plotted over time by analysis visits which are mapped from the first REACT injection referenced by Section 8.6. A listing of all calculated values will be presented.

11.2.3 Time from First REACT Injection to at least 40% Reduction in eGFR

This secondary efficacy endpoint is defined as the time from first injection to at least 40% reduction in eGFR sustained for 30 days. Events for inclusion in the analysis will be counted from the day of the first REACT injection until the end of study. Participants without an event will be censored as rules described in Section 8.3.10.

The number and percentage of participants with each event type and censoring reasons will be presented by cohort. Reasons for censoring will be listed and summarized according to the categories following the hierarchy shown in the Table 19.

Table 19: Censoring Reason for Time to least 40% Reduction in eGFR

Hierarchy	Condition	Censoring Reason
1	No adequate baseline assessment	No adequate baseline assessment
2	Start of chronic dialysis	Start of chronic dialysis
3	Start of renal transplant	Start of renal transplant
4	Death	Death
5	Discontinued from study without event occurring	Discontinued from study
6	Completed study without event occurring	Complete study

Kaplan-Meier methodology will be used to estimate median time to event for each cohort. The 95% CI for the median time to event along with lower and upper quartiles for each cohort will be calculated. The probability of experiencing less than a 40% reduction in eGFR at landmark times (e.g., 3, 6, 9, 12, 18, … months from first injection) will be estimated with corresponding 2-sided

95% CIs. A Kaplan-Meier plot will be generated by cohort. The figures will include the number of participants at risk and number of events along the horizontal axis during each time interval per cohort.

11.2.4 Time from First REACT Injection to eGFR <15 mL/min/1.73m^2 or Renal Replacement Therapy

Time from first REACT injection to eGFR <15 mL/min/1.73m^2 sustained for 30 days and/or the initiation of dialysis or renal transplantation will be analyzed using the same analytical method as Section 11.2.3. Participants without an event will be censored as rules described in Section 8.3.11.

The number and percentage of participants with each event type and censoring reasons will be presented by cohort. Reasons for censoring will be listed and summarized according to the categories following the hierarchy shown in Table 20.

Table 20: Censoring Reason for Time to Renal Replacement Therapy

Hierarchy	Condition	Censoring Reason
1	Discontinued from study without event occurring	Reason discontinued from study
2	Completed study without event occurring	Complete study

11.2.5 Time from First REACT Injection to at least 30% and 30 mg/g Increase in UACR

Time from first REACT injection to at least 30% and 30 mg/g increase in UACR sustained for 90 days will be analyzed using the same analytical method as Section 11.2.3. Participants without an event will be censored as rules described in Section 8.3.12.

The number and percentage of participants with each event type and censoring reasons will be presented by cohort. Reasons for censoring will be listed and summarized according to the categories following the hierarchy shown in Table 19.

11.2.6 Time from First REACT Injection to Renal or Cardiovascular Death

Time from first REACT injection to time to renal or cardiovascular death will be analyzed using the same analytical method as Section 11.2.3. Participants without an event will be censored as rules described in Section 8.3.13.

The number and percentage of participants with each event type and censoring reasons will be presented by cohort. Reasons for censoring will be listed and summarized according to the categories following the hierarchy shown in Table 21.

Table 21: Censoring Reason for Time to Renal or Cardiovascular Death

Hierarchy	Condition	Censoring Reason
1	Death due to any other cause	Death due to any other cause
2	Discontinued from study without event occurring	Reason discontinued from study
3	Completed study without event occurring	Complete study

11.2.7 Time from First REACT Injection to Four Composite Endpoint

Time from first REACT injection to the four composite endpoints will be analyzed using the same analytical method as Section 11.2.3. Participants without an event will be censored as rules described in Section 8.3.14.

- Any 40% reduction in eGFR, using the 2009 CKD-EPI serum creatinine equation, sustained for 30 days or
- eGFR <15 mL/min/1.73m² using the 2009 CKD-EPI serum creatinine equation, sustained for 30 days and/or chronic dialysis, and/or renal transplant or
- Increase of UACR of at least 30% and of at least 30 mg/g, using the urine microalbumin/urine creatinine ratio sustained for 90 days or
- Renal or cardiovascular death.

The number and percentage of participants with each event type and censoring reasons will be presented by cohort. Reasons for censoring will be listed and summarized according to the categories following the hierarchy shown in Table 22.

Table 22: Censoring Reason for Time to Four Composite Endpoint

Hierarchy	Condition	Censoring Reason
1	No adequate baseline assessment	No adequate baseline assessment
2	Death due to any other cause except the categorization of cardiovascular or renal.	Death other than cardiovascular or renal.
3	Discontinued from study without event occurring	Discontinued from study
4	Completed study without event occurring	Complete study

11.2.8 Time from First REACT Injection to Three Composite Endpoint

Time from first REACT injection to the three composite endpoints will be analyzed using the same analytical method as Section 11.2.3. Participants without an event will be censored as rules described in Section 8.3.15.

- Any 40% reduction in eGFR, using the 2009 CKD-EPI serum creatinine equation, sustained for 30 days or
- eGFR <15 mL/min/1.73m² using the 2009 CKD-EPI serum creatinine equation, sustained for 30 days and/or chronic dialysis, and/or renal transplant or
- Renal or cardiovascular death.

The number and percentage of participants with each event type and censoring reasons will be presented by cohort. Reasons for censoring will be listed and summarized according to the categories following the hierarchy shown in Table 22.

In addition, a set of subgroup analyses as described in Section 9 will be conducted for supportive analyses of this endpoint.

11.3 Exploratory Efficacy Endpoints and Analyses

The exploratory efficacy endpoints are listed in Section 3.

11.3.1 Efficacy Laboratory Measurements

Laboratory values for the laboratory parameters: eGFR, UACR, blood hemoglobin, blood hematocrit, serum calcium, serum phosphorus, plasma parathyroid hormone, serum potassium, and serum bicarbonate will be summarized by presenting descriptive statistics (including number of non-missing observations (n), mean, STD, median, 25th percentile, and 75th percentile, minimum, and maximum) of actual values and changes from baseline by cohort at analysis visit following the first REACT injection. The visit windowing convention will be conducted by referring to Section 8.6.

For efficacy analyses, measurements taken after a kidney transplant, or the initiation of dialysis, or on the biopsy or injection date post procedure will be removed from the summaries. If more than 1 assessment falls into the same analysis visit window, then the one that is closer to the target day will be used. If 2 assessments within the same analysis visit window are equidistant from the target day, then the analysis will use the later assessment.

A box plot will be generated for eGFR using 2009 CKD-EPI serum creatinine equation and UACR, to present mean, median, first and third quantiles, the upper boundary defined as third quartile plus 1.5 times the interquartile range (third quantile – first quantile), the lower boundary defined as first quartile minus 1.5 times the interquartile range, and the outliers which fall significantly outside the boundaries that are 1.5 times the interquartile range. The box plot will

be present over time by analysis visits which are mapped from the first REACT injection referenced by Section 8.6.

These efficacy laboratory values will be listed with a notation for assessments taken after a kidney transplant or the initiation of dialysis.

11.3.2 Time from First REACT Injection to All-cause Mortality

The time to all-cause mortality is defined as the time (months) between the date of first injection to all-cause mortality and will be analyzed using the same analytical method as Section 11.2.3. Participants without an event will be censored as rules described in Section 8.3.16. Participants without documented death at the time of analysis will be censored at the date last known to be alive.

Frequency (number and percentage) of participants with event and censored will be presented by cohort.

12 SAFETY ANALYSIS

Safety assessments will include adverse events, clinical laboratory assessments, pregnancy tests, physical examinations, vital signs, renal safety ultrasounds, renal imaging, and electrocardiograms (ECG). Safety evaluation will be performed according to the schedule of events presented in Appendix 1 and Appendix 2. Descriptive statistics will be tabulated to summarize safety variables. No inferential analyses of safety data are planned unless otherwise specified. The BS and SAF will be used for AE summary, and the SAF will be used for other safety summaries.

12.1 Adverse Events

An AE is a change from baseline medical status at study entry and typified by the development of an untoward medical condition (including abnormal ancillary diagnostic, imaging, physical examination, or laboratory values found clinically significant by the principal investigator) or the deterioration of a pre-existing medical condition following signing of informed consent (regardless of whether deterioration is expected for the natural history of disease state).

The Investigator is responsible for ensuring all AEs that occur from the day of randomization through the end of the study, whether observed by the Investigator or reported by the participant, are monitored and recorded in the participant's AE CRF.

12.1.1 Treatment-Emergent Adverse Events

Treatment-emergent AEs (TEAEs) are defined as any AE that started on or after the first injection of REACT to the end of the study.

The analysis periods defined in Section 8.3.3 will be used for analysis of the AEs and TEAEs, the AE occurred during the post-injection period are the TEAEs. In the case of partial or missing onset or end dates, the rules stated in Section 8.2.1 will be followed to determine the analysis period to which the AE belongs, and if the event is treatment-emergent.

12.1.2 Adverse Event Intensity

Intensity will be assessed by the Investigator using the CTCAE version 5.0, from the US National Cancer Institute. If the AE is not included in the CTCAE, then the Investigator will determine the intensity of the AE according to the following criteria:

- **Grade 1**: Mild; asymptomatic or mild symptoms; clinical or diagnostic observations only; intervention not indicated.
- **Grade 2**: Moderate; minimal, local, or noninvasive intervention indicated; limiting age-appropriate instrumental Activities of Daily Living.
- **Grade 3**: Severe or medically significant but not immediately life-threatening; hospitalization or prolongation of hospitalization indicated; disabling; limiting self-care ADL.
- **Grade 4**: Life-threatening consequences; urgent intervention indicated.
- **Grade 5**: Death related to AE.

12.1.3 Adverse Event Relationship to Biopsy, Investigational Product, or Injection Procedure

An Investigator may make the determination of relationship to the biopsy, REACT injection procedure or REACT investigational product for each AE.

For safety analyses, all AEs judged by the Investigator to be "possibly related" or "related" will be considered related AEs.

12.1.4 Serious Adverse Events

A serious adverse event (SAE) will be defined as any AE meeting one or more of the following criteria:

- Results in death

- Is life-threatening
- Requires in-patient hospitalization or prolongation of existing hospitalization that deviates from institutional standard of care or practice
- Results in persistent or significant disability or incapacity (or substantial disruption in the ability to conduct normal life functions)
- Results in a congenital anomaly or birth defect
- Is an important medical event that may jeopardize the participant and may require a medical or surgical intervention to prevent one of the outcomes listed above.

12.1.5 Adverse Events of Special Interest (AESI)

The AEs of special interest (AESI) for REACT are defined in Protocol Section 8.1.1.2 and are collected in the CRF. The AESI will be listed and summarized.

12.1.6 Renal-specific Adverse Events

The Renal-specific adverse events as follows are defined by the PT list in Appendix 4.

- Procedure-related Bleeding Events
- Acute Kidney Injury
- Renal and Urinary Tract Infections
- Renal Neoplasms.

12.1.7 Adverse Events Leading to Treatment Discontinuation, Study Withdrawal or Death

Adverse events leading to treatment discontinuation are collected on the CRF as “Drug Withdrawn” to question “Action taken with study treatment”.

AEs leading to study discontinuation are defined as AEs that have a “Yes” for the following question on the eCRF: “Did this AE cause the participant to discontinue from the study?”.

AEs leading to death are defined as AEs with an outcome of “Fatal” and/or CTCAE Grade equal to Death (Grade 5).

12.1.8 Adverse Event Summaries

All AEs will be classified by SOC and PT using MedDRA version 28.0 or later. Unless otherwise stated, the overall summary of AEs will be presented for the BS and SAF on pre-biopsy period, pre-injection period, and post-injection period for each of the study cohorts and overall, when it is applicable.

The AEs with start day prior to Biopsy date will be counted as the pre-biopsy period; the AEs with start day prior to the first REACT injection and on or after the biopsy will be counted as the pre-injection period; the AEs with start day on and post the first REACT injection will be counted as the post-injection period.

An overall summary of AEs will be presented on the number and percentage of participants experiencing the following:

- Any AE/TEAEs
- Any SAE/serious TEAEs
- Any AESI
- Any biopsy-related bleeding events (haemorrhage)
- Any injection-related bleeding events (haemorrhage)
- Any renal-specific AE/TEAEs
- Any AE/TEAEs with grade ≥3
- Any AE/TEAEs related to biopsy
- Any TEAE related to REACT injection procedure during the post-injection period
- Any TEAE related to REACT product during post-injection period
- Any SAE/serious TEAEs related to biopsy
- Any SAE/serious TEAEs related to REACT injection procedure during the post-injection period
- Any SAE/serious TEAEs related to REACT product during post-injection period
- Any AE/TEAEs related to biopsy with grade ≥3
- Any TEAE related to REACT injection procedure with grade ≥3 during post-injection period
- Any TEAE related to REACT product with grade ≥3 during post-injection period
- Any AE/TEAEs leading to treatment discontinuation
- Any AE/TEAEs leading to study discontinuation
- Any AE/TEAEs leading to death
- Any AE/TEAEs with outcome of death related to biopsy procedure
- Any AE/TEAEs with outcome of death related to REACT injection procedure during post-injection period
- Any AE/TEAEs with outcome of death related to REACT product during post-injection period.

In addition to tabulating the number and percentage of participants who experience AEs, the total number of AE episodes will also be provided. If a participant has repeated episodes of a particular AE, all episodes will be counted in the overall summary of adverse events table.

Unless otherwise stated, the number and percentage of participants reporting events as follows will be tabulated by MedDRA SOC, PT and CTCAE grade for the pre-biopsy, pre-injection period, and for post-injection period on the BS and SAF. In the table summarizing AEs by SOC, PT and CTCAE grade, the AEs will be presented in descending order of the SOC in the Grade ≥3 column of each cohort during each study period. Within each SOC, AEs will be sorted in descending order of the PT, and then alphabetically for ties.

- AEs/TEAEs
- AEs/TEAEs related to biopsy
- TEAE related to REACT injection procedure during the post-injection period
- TEAEs related to REACT product during post-injection period
- Serious AEs/TEAEs
- Serious AEs/TEAEs related to biopsy
- Serious TEAEs related to REACT injection procedure during post-injection period
- Serious TEAEs related to REACT product during post-injection period
- AESI.

Unless otherwise stated, the number and percentage of participants reporting events as follows will be tabulated by MedDRA SOC and PT for the pre-biopsy, pre-injection period, and for post-injection period on the BS and SAF.

- AEs/TEAEs leading to treatment discontinuation
- AEs/TEAEs related to biopsy leading to treatment discontinuation
- TEAEs related to REACT injection procedure leading to treatment discontinuation during post-injection period
- TEAEs related to REACT product leading to treatment discontinuation during post-injection period
- AEs/TEAEs leading to study discontinuation
- AEs/TEAEs related to biopsy leading to study discontinuation
- TEAEs related to REACT injection procedure leading to study discontinuation during post-injection period
- TEAEs related to REACT product leading to study discontinuation during post-injection period
- AEs/TEAEs leading to death.
- AEs/TEAEs related to biopsy leading to death
- TEAEs related to REACT injection procedure leading to death
- TEAEs related to REACT product leading to death.

At each level of summarization, a participant will be counted only once for each reported AE. In the tabulations of grade, the maximum reported toxicity grade will be counted for each participant. For the tabulations of relationship, the highest association to study treatment will be summarized. Refer to Section 8.2.3 when the intensity/grade, the relationship to biopsy, REACT injection, or REACT product is missing. In the by SOC and PT table, the AEs will be presented in tables in descending order of the SOC in the post-injection period column. Within each SOC, AEs will be sorted in descending order of the PT, and then alphabetically for ties in the overall summary.

The following adverse event summaries will be by cohort and study period sorted by PT only for Biopsied Set. Preferred terms will be sorted by descending frequency in the Post-injection period, and then alphabetically for ties in the overall summary.

- AEs/TEAEs
- AEs/TEAEs with CTCAE Grade ≥ 3
- AEs/TEAEs related to biopsy
- TEAE related to REACT injection procedure during the post-injection period
- TEAEs related to REACT product during post-injection period
- SAE/serious TEAEs
- Renal-specific AEs/TEAEs
- Biopsy-related bleeding event (haemorrhage)
- Injection-related bleeding event (haemorrhage).

The occurrences of all AEs will be listed for each participant. Listings will contain all collected information using the ITT Analysis Set. Listings will be sorted by Cohort, participant identification number, onset date/time, SOC, and PT. The listing will display the dates as they are collected. That is, if imputed dates were used to determine if the AE is treatment-emergent, the imputed dates will not be displayed in the listing. The listings will contain the analysis period assigned to each AE per Section 8.3.3.

12.2 Clinical Laboratory Assessments

Blood samples and urine samples will be collected for chemistry, hematology, coagulation, urinalysis, urine macro panel, lipid panel, electrolyte, other assay, and pregnancy tests in females of childbearing potential. The results will be summarized for the SAF; a summary of the laboratory parameters at the pre-baseline visits, baseline, each scheduled post-baseline visits result, and change from baseline will be provided by test category as in Table 23.

Table 23: Clinical Laboratory Evaluations

Chemistry	Hematology	Electrolyte
eGFR (calculated)<br>Alkaline phosphatase<br>ALT<br>AST<br>Bilirubin<br>Creatinine kinase<br>GGT<br>LDH<br>Albumin<br>Calcium<br>Calcium Corrected<br>Creatinine<br>Phosphate<br>Glucose<br>Urea Nitrogen	Hematocrit<br>Hemoglobin<br>RBC count & indices<br>WBC count & differential<br>**Pregnancy**<br>hCG (serum) – confirmatory<br>FSH<br>**Serology**<br>HBV surface antigen<br>HCV antibody<br>HCV RNA PCR - confirmatory<br>HIV antibodies	Anion gap<br>Bicarbonate<br>Chloride<br>Potassium<br>Sodium<br>**Urine Macro Panel**<br>pH<br>Ketones<br>Protein<br>Blood<br>Glucose<br>Leukocyte Cell Clumps
**Urinalysis**	**Coagulation Status**	**Other Assay**
BUN<br>CO2, total<br>Creatinine<br>Cystatin C<br>CRP<br>Phosphorus<br>Potassium<br>Chloride<br>Bicarbonate<br>Glucose<br>Albumin<br>Creatinine<br>Albumin/Creatinine (UACR)<br>Protein<br>Protein/Creatinine<br>Beta-2 Microglobulin<br>NGAL<br>Microscopic analysis (test stick)	APTT<br>PT-INR<br>Platelet count<br>Lipid Panel<br>Cholesterol<br>LDL<br>HDL<br>LDL:HDL ratio<br>Triglycerides	Beta-2 Microglobulin<br>CRP<br>Cystatin C<br>HbA1c<br>Parathyroid Hormone, Intact<br>iPTH<br>**Drug Screen**<br>Amphetamine<br>Barbiturates<br>Benzodiazepines<br>Cocaine<br>Opiates<br>Tetrahydrocannabinol<br>Phencyclidine

Abbreviations: ALT (Alanine Aminotransferase); APTT (Activated Partial Thromboplastin Time); AST (Aspartate Aminotransferase); BUN (Blood Urea Nitrogen); CO_2 (Carbon Dioxide); CRP (C-Reactive Protein); eGFR (Estimated Glomerular Filtration Rate); FSH (Follicle-Stimulating Hormone); GGT (Gamma-glutamyl Transferase); HbA1c (Glycosylated Hemoglobin); HBV (Hepatitis B Virus); hCG (Human Chorionic Gonadotropin); HCV (Hepatitis C Virus); HDL (High Density Lipoprotein); HIV (Human Immuno-deficiency Virus); LDH (Lactate Dehydrogenase); LDL (Low Density Lipoprotein); NGAL (Neutrophil Gelatinase-Associated Lipocalin); iPTH (intact Parathyroid Hormone); PT-INR (Prothrombin Time-International Normalized Ratio); RBC (Red Blood Cell); WBC (White Blood Cell).

Continuous clinical laboratory values will be summarized by presenting descriptive statistics, categorical parameters will be summarized by frequency and percentages.

Clinical laboratory results will be graded according to CTCAE criteria Version 5 for the analytes which are applicable for grading.

Laboratory results that are within normal ranges and are not graded as 1-4, will be summarized as ‘Grade 0’ which is defined as normal. Any assessment for which CTCAE toxicity grades are not available will not be included in any analysis for which toxicity grades are required. A shift in grade from baseline to the worst post-baseline value will be summarized. Both the scheduled and unscheduled assessments will be used to identify the worst post-baseline values.

A set of clinically relevant laboratory parameters will be summarized in shift tables with low-normal-high at baseline versus low-normal-high at follow-up in a 3-by-3 contingency table and will be provided to assess changes in laboratory values from baseline to each post-baseline visit. Reference ranges established by the central lab will be used to determine shifts.

All laboratory assessments will be listed with the corresponding normal ranges for each parameter.

Unless otherwise specified, clinical laboratory results will be converted to Conventional Units for analyses. Within each listing, laboratory values outside the normal ranges will be flagged as either high or low.

Results from all clinical laboratory parameters will be presented in data listings for the ITT Analysis Set.

Drug screening evaluations for each participant will be presented in data listings for the ITT analysis set. The protocol defined drug screening evaluations include: Amphetamine, Barbiturates, Benzodiazepines, Cocaine, Opiates, Phencyclidine, Methadone, and Propoxyphene.

Local laboratory tests on hemoglobin, hematocrit, and the urine spot test will be listed for the ITT Analysis Set.

12.3 Pregnancy Test

All pregnancies experienced by female participants and/or female partners of male participants enrolled in the study will be presented in data listings for the ITT analysis set. Results from all clinical laboratory parameters including pregnancy test will be presented in data listings for the ITT Analysis Set.

12.4 Research Samples

All pre- and post-dose research sample collection will be presented in data listings for the ITT Analysis Set.

12.5 Vital Signs

Vital signs will be collected on pulse rate (beats per minute), respiratory rate (breaths per minute), body temperature (°C), height (cm), weight (kg), body mass index (kg/m^2) and blood pressure measurements (mmHg). A summary of the vital sign parameters at the pre-baseline visits, baseline, each scheduled post-baseline visits result, and change from baseline will be provided for SAF. Blood pressure measurements are taken in triplicate at screening; blood pressure measurements, pulse rate and respiratory rate are taken multiple times during procedure day. For these cases, the average value of the pre and post procedure measurements will be summarized for the corresponding visit timepoint.

A listing of all the vital sign measures will also be presented for ITT Analysis Set.

12.6 Electrocardiogram (ECG) Assessments

The ECGs will be performed during Screening and at the EOS Visit. Descriptive summaries (values and changes from baseline) of the following electrocardiogram measurements including heart rate (HR)(bpm), PR interval (msec), RR interval (msec), QRS duration (msec), uncorrected QT interval (msec), and derived corrected QT interval from Fridericia formula as follows will be prepared by visit for the SAF.

QTcF will be derived from the QT interval and RR interval in msec based on Fridericia's formula. RR in sec will be derived from the HR as 60/HR in bpm when it is not collected.

$$QTcF(msec) = \frac{QT(msec)}{\sqrt[3]{RR(sec)}}$$

Overall interpretation results for ECG will be summarized using shift tables (Normal, Abnormal Not CS, Abnormal CS) comparing baseline to EOS.

A listing of all collected ECG measures will be provided. The listing will also include the calculated QTcF values.

12.7 Physical Examination

The Investigator or designee will perform the physical examination. The full comprehensive examination will assess all pertinent body systems at the Screening visit to establish baseline. Interim physical examinations will be performed to assess any post-baseline abnormalities and must include at least the following components: general appearance, chest/respiratory, cardiovascular, abdominal, and neurologic. If clinically indicated, a full PE may be performed.

The by visit physical examination results will be summarized for each body system using shift tables (Normal, Abnormal Not CS, Abnormal CS) comparing baseline to the post-baseline visit.

A listing of all physical examination data will be provided.

12.8 Renal Safety Ultrasound

The number and percentage of participants with each of the renal echogenicity categories, with each new or worsening abnormalities present on the renal safety ultrasound will be summarized at each visit for each kidney for BS.

Listings of renal safety ultrasound data will be provided.

12.9 Renal Imaging

The measurements of renal volume and cortical thickness for the biopsied and unbiopsied kidneys will be summarized descriptively at each visit for the BS Analysis Set. In addition, the number and percentage of participants will be summarized on the overall interpretation of each visit. For the by visit analysis, the values from the MRI will be used, if available. If MRI values are not available, then the measurements from the CT scan will be used.

The details of the collected renal imaging will be listed for ITT Analysis Set.

12.10 Dialysis and Renal Transplant

Dialysis and renal transplant will be summarized separately including the number of participants and the number of events with each type of dialysis, the duration of dialysis, and the duration from the first REACT injection to the first dialysis. Similarly, the number of participants with renal transplant and the duration from the first REACT injection to the first transplant will be summarized.

12.11 COVID-19 Impact

If a subject is tested and found positive for COVID-19, biopsies and injections will be postponed for a minimum of 30 days and maximum of 90 days from onset of symptoms/diagnosis and subjects must be cleared by the Investigator and Medical Monitor to proceed with procedures. A listing will be presented for any other scheduled visit missed or conducted virtually due to hospital policy (e.g., due to COVID-19 symptoms or positivity, or contact with a positive COVID-19 person), for ITT Analysis Set.

13 REFERENCES

1. Kidney Disease Improving Global Outcomes (KDIGO) CKD Work Group. KDIGO 2012Clinical Practice Guideline for the Evaluation and Management of Chronic Kidney Disease.*Kidney Int.* 2013; Suppl. 3:1-150.
2. Tangri, Navdeep, Morgan E. Grams, Andrew S. Levey, Josef Coresh, Lawrence J. Appel, Brad C. Astor, Gabriel Chodick, et al. "Multinational Assessment of Accuracy of Equations for Predicting Risk of Kidney Failure: A Meta-Analysis." *JAMA 315, no. 2* (January 12, 2016): 164–74.
3. CTCAE v5.0 https://ctep.cancer.gov/protocoldevelopment/electronic applications/docs/CTCAE_**v5**_Quick_Reference_8.5x11.pdf.

14 APPENDICES

Appendix 1: Time and Events Table

Table 1: Time and Events Table – Cohort 1

Clinical Assessment	Screening Visit	Randomization	Renal Biopsy****			Preparation and Shipment of REACT Product	First REACT Injection**			Follow-up First REACT Injection				Interval between REACT Injections = 3 Months (+60) Days***	Second REACT Injection [b]		Follow-Up Long-Term				EOS[c]
	Day −60 to −7*		Day −7 to −1*	Day 0* Biopsy +14 days	Day 1 Post Biopsy		Day −21 to −14*	Day 0* REACT Injection +30 days	Day 1	Day 7 Follow-Up	Day 14 ±7 days	Day 28 ±7 days	Month 3* (pre-2nd Injection) ±10 days		Day 0* REACT Injection +60 days	Day 1 Follow-up	Day 7 Follow-Up	Day 14 ±7 days	Day 28 ±7 days	Months 3,6, 9, 12, 15 ±10 days	Month 18 ±10 days
Obtain Informed Consent [d]	X																				
Verify I/E Criteria	X																				
Obtain Demographic Data	X																				
Obtain Medical History	X																				
Record Concomitant Medications	X		X	X	X		X	X	X	X	X	X	X		X	X	X	X	X	X	X
Perform Comprehensive PE[e]	X																				
Perform Interim PE [e]			X				X				X	X	X					X	X	X	X
Measure Vital Signs [f]	X		X	X [g]	X		X	X [g]	X	X	X	X	X		X [g]	X	X	X	X	X	X
Conduct Laboratory Tests	X		X	X	X		X	X	X	X	X	X	X		X	X	X	X	X	X	X
Perform 12-Lead ECG	X																				X
Perform Ultrasound				X [h]	X [h]			X [h]	X [h]	X [h]	X [h]				X [h]	X [h]	X [h]	X [h]			
Perform MRI Study	X [i]																				X
Admit to Hospital/Discharge				X [j]				X [j]							X [j]						
Perform Kidney Biopsy				X																	
Monitor/Record AEs			X	X	X		X	X	X	X	X	X	X		X	X	X	X	X	X	X
Inject Autologous REACT								X [k]							X [k]						
CT Scan								X							X						

Abbreviations: AE = adverse event; COVID-19 = coronavirus disease 2019; CT = computed tomography; ECG = electrocardiogram; EOS = End of Study; I/E = inclusion/exclusion; IgG = immunoglobulin G; MRI = magnetic resonance imaging; PE = physical examination; REACT = renal autologous cell therapy.

* If a subject is tested for COVID-19 and is found positive, biopsies and injections will need to be postponed for a minimum of 30 days and a maximum of 90 days from onset of symptoms/diagnosis and subjects must be cleared by Investigator and Medical Monitor for proceeding with procedures.

** **Every attempt should be made to ensure that the first REACT injection is administered 12 weeks (+30 days) after biopsy.** In the event that the subject cannot schedule his/her first REACT injection 12 weeks (+30 days) after biopsy or cannot keep his/her scheduled pre-first injection day visit, the Medical Monitor must be notified immediately.

*** **Every attempt should be made to ensure that the second REACT injection is administered 3 months (+60 days) after the first injection**. In the event that the subject cannot schedule his/her second REACT injection 3 months after receiving the first injection or cannot keep his/her scheduled 6-month visit, the Sponsor and the Medical Monitor must be notified immediately.

**** Subjects must not receive a vaccine of any kind within a minimum of 30 and maximum of 90 days before or after biopsy and injection procedures depending on a consult between Investigator and Medical Monitor for evaluation of adverse effects.

a. If the screening assessment falls outside of the 60-day window before renal biopsy, rescreening will be performed as described in Section 6.1.
b. If a subject does not receive a second REACT injection they must complete all visits per protocol except visits day 1, day 7, and day 14 post second REACT injection. On Second REACT Injection Day subjects will still undergo the following assessments: record con meds, vital signs, laboratory tests, and record AE's. Subjects will not receive ultrasound, CT, or undergo the injection procedure.
c. The EOS Visit will take place at least 18 months after the second REACT injection or when the subject is terminated from the study by the Investigator (Section 8.4), or when the subject voluntarily discontinues from the study (Section 4.8).
d. The informed consent form must be signed and dated prior to conducting any study-specific procedures, including those at the Screening Visit.
e. The comprehensive PE and interim PE are described in Section 7.2.2.
f. Vital signs include heart rate, resting blood pressure, respiration rate, and body temperature (Section 7.2.1).
g. Vital signs, including heart rate, blood pressure, and respiration rate, will be measured throughout the procedure. Temperature is not required to be recorded throughout the procedure but should be documented once.
h. Ultrasound will be performed following the in-subject renal biopsy on Day 0 Biopsy and Day 1 Post-Biopsy with the aim of monitoring possible, subclinical AEs. Subsequent ultrasounds will occur on REACT Injection days post procedure, Day 1 and Day 7post injections to monitor for subclinical AEs. If there is a hematoma greater than 2 cm present on Day 0, Day 1 or Day 7 for REACT injections, then an ultrasound should be conducted at Day 14.
i. An MRI study without contrast will be performed at the Screening Visit to determine kidney size and volume. If a subject cannot undergo MRI then CT can be substituted to obtain kidney size and volume. If subject cannot undergo MRI or CT please consult with Medical Monitor for approval for subject to continue in the study.
j. Subjects may be admitted to hospital per site standard practice. Subjects who do not experience complications may be discharged the same day consistent with site standard practice.
k. The REACT preparation will be handled and injected according to procedures described in the Procedure Training Manual.

Table 3: Time and Events Table – Cohort 2 for Subjects Not Meeting Renal Function Redose Trigger for a Second REACT Injection

Clinical Assessment	Screening Visit	Randomization	Renal Biopsy**			Preparation and Shipment of REACT Product	First REACT Injection***			Follow-Up Long-Term [b]				EOS [b]
	Day –60 to –7 [a]		Day –7 to –1*	Day 0* Biopsy +14 days	Day 1 Post Biopsy		Day -21 to -14*	Day 0* REACT Injection +30 days	Day 1 Follow-up	Day 7 Follow-Up	Day 14 ±7 days	Day 28 ±7 days	Months 3,6, 9, 12, 15 ±10 days	Month 18 ±10 days
Obtain Informed Consent [c]	X													
Verify I/E Criteria	X													
Obtain Demographic Data	X													
Obtain Medical History	X													
Record Concomitant Medications	X		X	X	X		X	X	X	X	X	X	X	X
Perform Comprehensive PE [d]	X													
Perform Interim PE [d]			X				X				X	X	X	X
Measure Vital Signs [e]	X		X	X [f]	X [f]		X	X [f]	X	X	X	X	X	X
Conduct Laboratory Tests	X		X	X	X		X	X	X	X	X	X	X	X
Perform 12-Lead ECG	X													X
Perform Ultrasound				X [g]	X [g]			X [g]	X [g]	X [g]	X [g]			
Perform MRI Study	X [h]													X
Admit to Hospital/Discharge				X [i]				X [i]						
Perform Kidney Biopsy				X										
Monitor/Record AEs			X	X	X		X	X	X	X	X	X	X	X
Inject Autologous REACT								X [j]						
CT Scan								X						
Evaluation for Criteria to Administer a Second REACT Injection [b]													Reevaluate up to Month 15 [b]	

Abbreviations: AE = adverse event; COVID-19 = coronavirus disease 2019; CT = computed tomography; ECG = electrocardiogram; EOS = End-of-Study; I/E = inclusion/exclusion; IgG = immunoglobulin G; MRI = magnetic resonance imaging; PE = physical examination; REACT = renal autologous cell therapy.

* If a subject is tested for COVID-19 and is found positive, biopsies and injections will need to be postponed for a minimum of 30 days and maximum of 90 days from onset of symptoms/diagnosis and subjects must be cleared by Investigator and Medical Monitor for proceeding with procedures.

** Subjects must not receive a vaccine of any kind within a minimum of 30 and a maximum of 90 days before or after biopsy and injection procedures depending on a consult between Investigator and Medical Monitor for evaluation of adverse effects.

*** **Every attempt should be made to ensure that the first REACT injection is administered 12 weeks (+30 days) after biopsy.** In the event that the subject cannot schedule his/her first REACT injection 12 weeks (+30 days) after biopsy or cannot keep his/her scheduled pre-first injection day visit, the Medical Monitor must be notified immediately.

a. If the screening assessment falls outside of the 60-day window before renal biopsy, rescreening will be performed as described in Section 6.1.
b. In the event that the subject meets criteria for a second REACT injection at least 3 months after first REACT injection and up to/including Month 15 (Section 4.6.2.1), the study assessments should be modified and follow the "Time and Events Table – Cohort 2 for Subjects Meeting Renal Function Redose Trigger for a Second REACT Injection" (Table 5). An unscheduled visit should be performed to confirm sustainment of trigger. The EOS Visit will take place at least 18 months after the last REACT injection or when the subject is terminated from the study by the Investigator (Section 8.4) or when the subject voluntarily discontinues from the study (Section 4.8).
c. The informed consent form must be signed and dated prior to conducting any study-specific procedures, including those at the Screening Visit.
d. The comprehensive PE and interim PE are described in Section 7.2.2.
e. Vital signs include heart rate, resting blood pressure, respiration rate, and body temperature (Section 7.2.1).
f. Vital signs, including heart rate, blood pressure, and respiration rate, will be measured at regular intervals throughout the procedure. Temperature is not required to be recorded throughout the procedure but should be documented once.
g. Ultrasound will be performed following the in-subject renal biopsy on Day 0 Biopsy and Day 1 Post Biopsy with the aim of monitoring possible, subclinical AEs. Subsequent ultrasounds will occur on REACT Injection days post procedure, Day 1 and Day 7 post injections to monitor for subclinical AEs. If there is a hematoma greater than 2 cm present on Day 0, Day 1, or Day 7 for REACT injections, then an ultrasound should be conducted at Day 14.
h. An MRI study without contrast will be performed at the Screening Visit to determine kidney size and volume. If a subject cannot undergo MRI then CT can be substituted to obtain kidney size and volume. If subject cannot undergo MRI or CT please consult with Medical Monitor for approval for subject to continue in the study.
i. Subjects may be admitted to hospital per site standard practice. Subjects who do not experience complications may be discharged the same day consistent with site standard practice.
j. The REACT preparation will be handled and injected according to procedures described in the Procedure Training Manual.

Table 5: Time and Events Table – Cohort 2 for Subjects Meeting Renal Function Redose Trigger for a Second REACT Injection

Clinical Assessment	**Second REACT Injection [a]		Follow-Up Long-Term				EOS [b]
	Day 0*	Day 1	Day 7	Day 14	Day 28	Months 3,6, 9, 12, 15	Month 18
	REACT Injection	Follow-up	Follow-up	±7 days	±7 days	±10 days	±10 days
Record Concomitant Medications	X	X	X	X	X	X	X
Perform Interim PE [c]				X	X	X	X
Measure Vital Signs [d]	X [e]	X	X	X	X	X	X
Conduct Laboratory Tests	X	X	X	X	X	X	X
Perform 12-Lead ECG							X
Perform Ultrasound	X [f]	X [f]	X [f]	X [f]			
Perform MRI Study							X
Admit to Hospital/Discharge	X [g]						
Monitor/Record AEs	X	X	X	X	X	X	X
Inject Autologous REACT	X [h]						
CT Scan	X						

Abbreviations: Abbreviations: AE = adverse event; COVID-19 = coronavirus disease 2019; CT = computed tomography; ECG = electrocardiogram; EOS = End-of-Study; IgG = immunoglobulin G; MRI = magnetic resonance imaging; PE = physical examination; REACT = renal autologous cell therapy.

* If a subject is tested for COVID-19 and is found positive, biopsies and injections will need to be postponed for a minimum of 30 days and a maximum of 90 days from onset of symptoms/diagnosis and subject must be cleared by Investigator and Medical Monitor for proceeding with procedures.

** Subjects must not receive a vaccine of any kind within a minimum of 30 and a maximum of 90 days before or after biopsy and injection procedures depending on a consult between Investigator and Medical Monitor for evaluation of adverse effects.

a. In the event that the subject meets criteria for a second REACT injection prior to Month 15 (Section 4.6.2.1), the study assessments should be modified and follow the "Time and Events Table – Cohort 2 for Subjects Meeting Renal Function Redose Trigger for a Second REACT Injection" (Table 5). If a subject does not receive the second REACT injection by 60 days of meeting renal function trigger for the second injection, the subject will not undergo the second injection and remain on his/her original follow-up schedule of assessments.

b. The EOS Visit will take place at least 18 months after the second REACT injection or when the subject is terminated from the study by the Investigator (Section 8.4) or when the subject voluntarily discontinues from the study (Section 4.8).

c. The comprehensive PE and interim PE are described in Section 7.2.2.

d. Vital signs include heart rate, resting blood pressure, respiration rate, and body temperature (Section 7.2.1).

e. Vital signs, including heart rate, blood pressure, and respiration rate, will be measured at regular intervals throughout the procedure. Temperature is not required to be recorded throughout the procedure but should be documented once.

f. Ultrasound will be performed following the in-subject renal biopsy on Day 0 Biopsy and Day 1 Post-Biopsy with the aim of monitoring possible, subclinical AEs. Subsequent ultrasounds will occur on REACT Injection days post procedure, Day 1 and Day 7 post injections to monitor for subclinical AEs. If there is a hematoma greater than 2 cm present on Day 0, Day 1 or Day 7 for REACT injections, then an ultrasound should be conducted at Day 14.

g. Subjects may be admitted to hospital per site standard practice. Subjects who do not experience complications may be discharged the same day consistent with site standard practice.

h. The REACT preparation will be handled and injected according to procedures described in the REACT Training Manual.

Appendix 2: Laboratory Time and Events Appendix

Table 2: Laboratory Time and Events Table – Cohort 1

Clinical Assessment	Screening Visit	Renal Biopsy			First REACT Injection			Follow-up First REACT Injection				Second REACT Injection [b]		Follow-Up Long-Term				EOS [c]
	Day –60 to –7 [a]	Day –7 to –1*	Day 0* Biopsy +14 days [g]	Day 1	Day -21 to -14*	Day 0* REACT Injection +30 days [g]	Day 1 Follow-up	Day 7 Follow Up	Day 14 ±7 days	Day 28 ±7 days	Month 3* (pre-2nd injection visit) ±10 days	Day 0* REACT Injection +60days [g]	Day 1 Follow-up	Day 7 Follow Up	Day 14 ±7 days	Day 28 ±7 days	Months 3,6, 9, 12, 15 ±10 days	Month 18 ±10 days
Clinical Chemistry																		
Standard panel	X	X	X	X	X	X	X	X	X	X	X	X	X	X	X	X	X	X
Renal analytes	X	X	X	X	X	X	X	X	X	X	X	X	X	X	X	X	X	X
Electrolyte panel	X	X	X	X	X	X	X	X	X	X	X	X	X	X	X	X	X	X
Lipid panel	X	X	X	X	X	X	X	X	X	X	X	X	X	X	X	X	X	X
Pregnancy test [d]	X	X	X	X	X	X	X	X	X	X	X	X	X	X	X	X	X	X
FSH test [e]	X																	
Serology																		
HIV, HBV, HCV	X																	
Hematology																		
Standard cell counts/indices	X	X	X	X	X	X	X	X	X	X	X	X	X	X	X	X	X	X
Hemoglobin, hematocrit [f]	X	X [f]	X [g]	X	X [f]	X [g]	X	X	X	X	X [f]	X [g]	X	X	X	X	X	X
Coagulation Status																		
Platelet count	X	X			X						X							X
APTT	X	X			X						X							X
PT-INR	X	X			X						X							X
Urine Chemistry																		
Standard (macro panel)	X	X	X	X	X	X	X	X	X	X	X	X	X	X	X	X	X	X
Microalbumin/creatinine ratio	X	X	X	X	X	X	X	X	X	X	X	X	X	X	X	X	X	X
Protein/creatinine ratio	X	X	X	X	X	X	X	X	X	X	X	X	X	X	X	X	X	X
Spot test (micro panel)	X	X [h]	X [h]		X [h]	X [h]	X [h]				X [h]	X [h]	X [h]					
Additional Test																		
HbA_{1c}	X		X		X	X					X	X					X	X
Drugs of abuse	X																	
iPTH	X				X	X					X	X					X	X
NGAL	X	X	X	X	X	X	X	X	X	X	X	X	X	X	X	X	X	X
Research (reserve) samples [i]	X	X	X	X	X	X	X	X	X	X	X	X	X	X	X	X	X	X

Abbreviations: APTT = activated partial thromboplastin time; COVID-19 = coronavirus disease 2019; EOS = End-of-Study; FSH = follicle-stimulating

hormone; HbA_{1c} = glycosylated hemoglobin; HBV = hepatitis B virus; hCG = human chorionic gonadotropin; HCV = hepatitis C virus; HIV = human immunodeficiency virus; IgG = immunoglobulin G; NGAL = neutrophil gelatinase-associated lipocalin; iPTH = intact parathyroid hormone; PT-INR = prothrombin time-international normalized ratio; REACT = Renal Autologous Cell Therapy.

* If a subject is tested for COVID-19 and receives a positive result, biopsies and injections will need to be postponed for a minimum of 30 days and maximum of 90 days from onset of symptoms/diagnosis and the subject must be cleared by Investigator and Medical Monitor for proceeding with procedures.

a. If the screening assessment falls outside of the 60-day window before renal biopsy, rescreening will be performed as described in Section 6.1.
b. If a subject does not receive a second REACT injection they must complete all visits per protocol except visits Day 1, Day 7 and Day 14 post second REACT injection. On Second REACT Injection Day subjects will still undergo the following assessments: record con meds, vital signs, laboratory tests, and record AE's. Subjects will not receive ultrasound, CT, or undergo the injection procedure.
c. The EOS Visit will take place at least 18 months after the second REACT injection or when the subject is terminated from the study by the Investigator (Section 8.4) or when the subject voluntarily discontinues from the study (Section 4.8).
d. The clinic will perform a urine dip-strip pregnancy test. If positive, then a confirmatory serum hCG test will be performed by the central laboratory. A serum sample may be collected for a pregnancy test where a urine sample is not available.
e. Post-menopausal women with a confirmatory FSH test do not have to undergo pregnancy testing throughout the study.
f. At the Day -7 to Day -1 visit before Day 0 for renal biopsy and Day -21 to Day -14 prior to first REACT injection Day 0, and at Month 3 visit prior to second REACT injection Day 0, hemoglobin levels will be verified as >9 g/dL per site standard practices.
g. On Days 0 for renal biopsy and REACT injection(s), hemoglobin and hematocrit will be measured locally before and after the procedure. These samples will be processed by the site's local laboratory to accelerate notification of results and subsequent decisions affecting clinical care. In addition, blood samples for hemoglobin and hematocrit will be sent to the central laboratory where results can be entered into the study database. The Day 0 PRE central laboratory kit is to be used before procedures and the Day 0 Post central laboratory kit should be used after procedures.
h. At Screening, Biopsy Day -7 to -1, day of renal biopsy; Day -21 to -14 visit before the first injection, on the day of, and day after REACT injection(s), microscopic urinalysis will be performed by Labcorp to confirm the absence of infection in addition to urine test stick at the clinical site. On procedure days the urine test stick should be completed prior to procedure only.
i. Research samples (serum/plasma and urine) will be collected, frozen, and stored for the evaluation of novel biomarkers.

Table 4: Laboratory Time and Events Table – Cohort 2 for Subjects Not Meeting Renal Function Redose Trigger for Second REACT Injection

Clinical Assessment	Screening Visit	Renal Biopsy			First REACT Injection			Follow-Up Long-Term [b]				EOS [c]
	Day –60 to –7 [a]	Day –7 to –1*	Day 0* Biopsy +14 days [g]	Day 1 Post Biopsy	Day -21 to -14*	Day 0* REACT Injection +30 days [g]	Day 1 Follow-up	Day 7 Follow-Up	Day 14 ±7 days	Day 28 ±7 days	Months 3,6, 9, 12, 15 ±10 days	Month 18 ±10 days
Clinical Chemistry												
Standard panel	X	X	X	X	X	X	X	X	X	X	X	X
Renal analytes	X	X	X	X	X	X	X	X	X	X	X	X
Electrolyte panel	X	X	X	X	X	X	X	X	X	X	X	X
Lipid panel	X	X	X	X	X	X	X	X	X	X	X	X
Pregnancy test [d]	X	X	X	X	X	X	X	X	X	X	X	X
FSH test [e]	X											
Serology												
HIV, HBV, HCV	X											
Hematology												
Standard cell counts/indices	X	X	X	X	X	X	X	X	X	X	X	X
Hemoglobin, hematocrit [f]	X	X [f]	X [g]	X	X [f]	X [g]	X	X	X	X	X	X
Coagulation Status												
Platelet count	X	X			X							X
APTT	X	X			X							X
PT-INR	X	X			X							X
Urine Chemistry												
Standard (macro panel)	X	X	X	X	X	X	X	X	X	X	X	X
Microalbumin/creatinine ratio	X	X	X	X	X	X	X	X	X	X	X	X
Protein/creatinine ratio	X	X	X	X	X	X	X	X	X	X	X	X
Spot test (micro panel)	X	X [h]	X [h]		X [h]	X [h]	X [h]					
Additional Tests												
HbA_{1c}	X		X		X	X					X	X
Drugs of abuse	X											
iPTH	X				X	X					X	X
NGAL	X	X	X	X	X	X	X	X	X	X	X	X
Research (reserve) samples [i]	X	X	X	X	X	X	X	X	X	X	X	X
Evaluation for Criteria to Administer a Second REACT Injection [b]											Reevaluate up to Month 15 [b]	

Abbreviations: APTT = activated partial thromboplastin time; COVID-19 = coronavirus disease 2019; EOS = End-of-Study; FSH = follicle-stimulating hormone; HbA_{1c} = glycosylated hemoglobin; HBV = hepatitis B virus; hCG = human chorionic gonadotropin; HCV = hepatitis C virus; HIV = human immunodeficiency virus; IgG = immunoglobulin G; NGAL = neutrophil gelatinase-associated lipocalin; iPTH = intact parathyroid hormone; PT-INR = prothrombin time-international normalized ratio; REACT = Renal Autologous Cell Therapy.

* If a subject is tested for COVID-19 and receives a positive result, biopsies and injections will need to be postponed for a minimum of 30 days and a maximum

of 90 days from onset of symptoms/diagnosis and the subject must be cleared by Investigator and Medical Monitor for proceeding with procedures.

a. If the screening assessment falls outside of the 60-day window before renal biopsy, rescreening will be performed as described in Section 6.1.
b. In the event that the subject meets criteria for a second REACT injection at least 3 months after first REACT injection and up to/including Month 15 (Section 4.6.2.1), the study assessments should be modified and follow the "Time and Events Table – Cohort 2 for Subjects Meeting Renal Function Redose Trigger for a Second REACT Injection" (Table 5). An unscheduled visit/s should be performed to confirm sustainment of triggers.
c. The EOS Visit will take place at least 18 months after the last REACT injection or when the subject is terminated from the study by the Investigator (Section 8.4) or when the subject voluntarily discontinues from the study (Section 4.8).
d. The clinic will perform a urine dip-strip pregnancy test. If positive, then a confirmatory serum hCG test will be performed by the central laboratory. A serum sample may be collected for a pregnancy test where a urine sample is not available.
e. Post-menopausal women with a confirmatory FSH test do not have to undergo pregnancy testing throughout the study.
f. At the Day -7 to Day -1 visit before Day 0 for renal biopsy and Day -21 to Day -14 prior to first REACT injection Day 0, hemoglobin levels will be verified as >9 g/dL per site standard practices.
g. On Days 0 for renal biopsy and REACT injection(s), hemoglobin and hematocrit will be measured locally before and after the procedure. These samples will be processed by the site's local laboratory to accelerate notification of results and subsequent decisions affecting clinical care. In addition, blood samples for hemoglobin and hematocrit will be sent to the central laboratory where results can be entered into the study database. The Day 0 PRE central laboratory kit is to be used before procedures and the Day 0 Post central laboratory kit should be used after procedures.
h. At Screening, Biopsy Day -7 to -1, day of renal biopsy; at the Day -21 to -14 visit before the first injection, on the day of, and day after REACT injection, microscopic urinalysis will be performed by Labcorp to confirm the absence of infection in addition to urine test stick at the clinical site. On procedure days the urine test stick should be completed prior to procedure only.
i. Research samples (serum/plasma and urine) will be collected, frozen, and stored for the evaluation of novel biomarkers.

Table 6: Laboratory Time and Events Table – Cohort 2 for Subjects Meeting Renal Function Redose Trigger for Second REACT Injection

Clinical Assessment	Second REACT Injection		Follow-Up Long-Term				EOS [a]
	Day 0* REACT Injection [c]	Day 1 Follow-up	Day 7 Follow-Up	Day 14 ±7 days	Day 28 ±7 days	Months 3,6, 9, 12, 15 ±10 days	Month 18 ±10 days
Clinical Chemistry							
Standard panel	X	X	X	X	X	X	X
Renal analytes	X	X	X	X	X	X	X
Electrolyte panel	X	X	X	X	X	X	X
Lipid panel	X	X	X	X	X	X	X
Pregnancy test [b]	X	X	X	X	X	X	X
FSH test							
Serology							
HIV, HBV, HCV							
Hematology							
Standard cell counts/indices	X	X	X	X	X	X	X
Hemoglobin, hematocrit [c]	X [c]	X	X	X	X	X	X
Coagulation Status							
Platelet count							X
APTT							X
PT-INR							X
Urine Chemistry							
Standard (macro panel)	X	X	X	X	X	X	X
Microalbumin/creatinine ratio	X	X	X	X	X	X	X
Protein/creatinine ratio	X	X	X	X	X	X	X
Spot test (micro panel)	X [d]	X [d]					
Additional Tests							
HbA_{1c}	X					X	X
Drugs of abuse							
iPTH	X					X	X
NGAL	X	X	X	X	X	X	X
Research (reserve) samples [e]	X	X	X	X	X	X	X

Abbreviations: Abbreviations: APTT = activated partial thromboplastin time; COVID-19 = coronavirus disease 2019; EOS = End-of-Study; FSH = follicle-stimulating hormone; HbA_{1c} = glycosylated hemoglobin; HBV = hepatitis B virus; hCG = human chorionic gonadotropin; HCV = hepatitis C virus; HIV = human immunodeficiency virus; IgG = immunoglobulin G; NGAL = neutrophil gelatinase-associated lipocalin; iPTH = intact parathyroid hormone; PT-INR = prothrombin time-international normalized ratio; REACT = Renal Autologous Cell Therapy.

* If a subject is tested for COVID-19 and receives a positive result, biopsies and injections will need to be postponed for a minimum of 30 days and maximum

of 90 days from onset of symptoms/diagnosis and the subject must be cleared by Investigator and Medical Monitor for proceeding with procedures.

a. The EOS Visit will take place at least 18 months after the second REACT injection or when the subject is terminated from the study by the Investigator (Section 8.4) or when the subject voluntarily discontinues from the study (Section 4.8).
b. The clinic will perform a urine dip-strip pregnancy test. If positive, then a confirmatory serum hCG test will be performed by the central laboratory. A serum sample may be collected for a pregnancy test where a urine sample is not available.
c. On Days 0 for REACT injection(s), hemoglobin and hematocrit will be measured locally before and after the procedure. These samples will be processed by the site's local laboratory to accelerate notification of results and subsequent decisions affecting clinical care. In addition, blood samples for hemoglobin and hematocrit will be sent to the central laboratory where results can be entered into the study database. The Day 0 PRE central laboratory kit is to be used before procedures and the Day 0 Post central laboratory kit should be used after procedures.
d. At the Day -21 to -14 visit before the first injection and on the day of, and day after REACT injection(s), microscopic urinalysis will be performed by Labcorp to confirm the absence of infection in addition to urine test stick at the clinical site. On procedure days the urine test stick should be completed prior to procedure only.
e. Research samples (serum/plasma and urine) will be collected, frozen, and stored for the evaluation of novel biomarkers.

Appendix 3: Sample SAS Code for Statistical Analyses

1. Sample SAS Code for annualized change in eGFR for post-first injection period using the mixed effect model

```
ods output solutionf = solutionf;
PROC MIXED data=work method=ML;
class <Subject>;
model <CHG> = <base> <years from the first REACT injection>/ TYPE=UN
ddfm=kr s cl;
/*Type=ARAM(1,1) and then Type=AR(1) and then Type=CS and then Type=VC will
be used if UN does not work*/
Random INT <years from first REACT injection > / type=un
subject=<Subject>;
By <cohort>;
Run;
```

2. SAS code for slope analysis between Pre-injection period and Post-injection period using the full mixed effect model

```
ods output solutionf = solutionf  estimates = estimates;
PROC MIXED data=work method=ML;
class <Subject> <Study Period>;
model <CHG> = <base> <Study Period> <years from reference injection or
screening > <Study Period>*<years from reference injection or
screening >/ ddfm=kr s cl;
Random INT <years from reference injection or screening > / type=un
subject=<Subject>; /*Type=ARMA(1,1) and then Type=AR(1) and then Type=CS and
then Type=VC will be used if UN does not work*/

Estimate 'Slope Diff between Post-last injection period vs. Pre-
injection period' <Study Period>*<years from reference injection or
screening > 1 -1/e cl;
Estimate 'Slope of Post-last injection period ' <years from reference
injection or screening > 1 <Study Period>*<years from reference
injection or screening > 1 0/e cl;
Estimate 'Slope of Pre-injection period' <years from reference
injection or screening > 1 <Study Period> * <years from reference
injection or screening > 0 1/e cl;
By <cohort>;
Run;
```

3. Sample SAS Code for Kaplan-Meier Analysis

```
ods output Quartiles = Quartiles
ProductLimitEstimates = ProductLimitEstimates;
proc lifetest data= work timelist =3 4 5 6 7 8 9 10 11 12 18 reduceout
outsurv=survest;
     time <aval> * <cnsr> (1);
    by <cohort>;
run;
```

Appendix 4: Renal-specific Adverse Events by Preferred Term

Note: The summary below is an example of the preferred terms to be included in renal-specific analyses and additional preferred terms may be added outside the scope of this document. The renal-specific preferred term list might require an update based on the latest MedDRA version prior to database lock.

			**Renal Neoplasms**		
**Procedure-related Bleeding Events (Hemorrhage FMQ)**	**Acute Kidney Injury (FMQ/SMQ-Narrow)**	**Renal and Urinary Tract Infections (FMQ-Narrow)**	**Renal Neoplasms Malignant (HLT)**	**Renal Pelvis and Ureter Neoplasms Malignant (HLT)**	**Renal Neoplasms Benign (HLT)**
Abdominal aortic aneurysm haemorrhage<br>Abdominal haematoma<br>Abdominal wall haematoma<br>Abdominal wall haemorrhage<br>Abnormal withdrawal bleeding<br>Acute haemorrhagic leukoencephalitis<br>Acute haemorrhagic ulcerative colitis<br>Administration site bruise<br>Administration site haematoma<br>Administration site haemorrhage<br>Adrenal haematoma<br>Adrenal haemorrhage<br>Anal fissure haemorrhage<br>Anal haemorrhage<br>Anal ulcer haemorrhage<br>Anastomotic haemorrhage<br>Anastomotic ulcer haemorrhage<br>Anastomotic ulcer haemorrhage, obstructive<br>Aneurysm ruptured<br>Anorectal varices haemorrhage<br>Antepartum haemorrhage<br>Aortic aneurysm rupture	Acute kidney injury<br>Acute phosphate nephropathy<br>Acute prerenal failure<br>Anuria<br>Azotaemia<br>Cardiorenal syndrome<br>Continuous haemodiafiltration<br>Crush syndrome<br>Crystal nephropathy<br>Dialysis<br>Frasier syndrome<br>GRACILE syndrome<br>Haemodialysis<br>Haemofiltration<br>Haemolytic uraemic syndrome<br>Hepatorenal failure<br>Nephritis<br>Nephropathy toxic<br>Oliguria<br>Pancreatorenal syndrome<br>Peritoneal dialysis<br>Postoperative renal failure<br>Postrenal failure<br>Prerenal failure<br>Renal failure	Adenoviral haemorrhagic cystitis<br>Aerococcus urinae infection<br>Bacteriuria<br>Bacterial pyelonephritis<br>Bacterial ureteritis<br>Bacterial urethritis<br>Bladder candidiasis<br>Campylobacter urinary tract infection<br>Candida urethritis<br>Culture urine positive<br>Cystitis<br>Cystitis bacterial<br>Cystitis erosiv<br>Cystitis escherichia<br>Cystitis gonococcal<br>Cystitis helminthic<br>Cystitis klebsiella<br>Cystitis pseudomonal<br>Cystitis viral<br>Cytomegalovirus urinary tract infection	Chromophobe renal cell carcinoma<br>Clear cell papillary renal cell carcinoma<br>Clear cell renal cell carcinoma<br>Clear cell sarcoma of the kidney<br>Denys-Drash syndrome<br>Hereditary leiomyomatosis renal cell carcinoma<br>Hereditary papillary renal carcinoma<br>Metastatic renal cell carcinoma<br>Nephroblastoma<br>Non-renal cell carcinoma of kidney<br>Papillary renal cell carcinoma<br>Renal cancer<br>Renal cancer metastatic<br>Renal cancer recurrent<br>Renal cancer stage I<br>Renal cancer stage II<br>Renal cancer stage III<br>Renal cancer stage IV	Malignant neoplasm of renal pelvis<br>Transitional cell cancer of renal pelvis and ureter metastatic<br>Transitional cell cancer of the renal pelvis and ureter<br>Transitional cell cancer of the renal pelvis and ureter localised<br>Transitional cell cancer of the renal pelvis and ureter recurrent<br>Transitional cell cancer of the renal pelvis and ureter regional<br>Ureteric cancer<br>Ureteric cancer local<br>Ureteric cancer metastatic<br>Ureteric cancer recurrent<br>Ureteric cancer regional	Acquired cystic kidney disease<br>Benign renal neoplasm<br>Congenital cystic kidney disease<br>Congenital renal cyst<br>Mesoblastic nephroma<br>Multilocular cystic nephroma<br>Papillary tumour of renal pelvis<br>Renal adenoma<br>Renal cyst<br>Renal cyst haemorrhage<br>Renal cyst infection<br>Renal cyst ruptured<br>Renal haemangioma<br>Renal hamartoma<br>Renal lipoma<br>Renal oncocytoma<br>Senior-Loken syndrome

			Renal Neoplasms		
**Procedure-related Bleeding Events (Hemorrhage FMQ)**	**Acute Kidney Injury (FMQ/SMQ-Narrow)**	**Renal and Urinary Tract Infections (FMQ-Narrow)**	**Renal Neoplasms Malignant (HLT)**	**Renal Pelvis and Ureter Neoplasms Malignant (HLT)**	**Renal Neoplasms Benign (HLT)**
Aortic annulus rupture	Renal failure acute	Emphysematous cystitis	Renal cell carcinoma		
Aortic dissection rupture	Renal injury		Renal cell carcinoma recurrent		
Aortic intramural haematoma	Renal impairment	Emphysematous pyelonephritis	Renal cell carcinoma stage I		
Aortic perforation	Renal ischaemia	Escherichia pyelonephritis	Renal cell carcinoma stage II		
Aortic rupture	Renal tubular injury	Escherichia urinary tract infection	Renal cell carcinoma stage III		
Aortoenteric fistula	Renal tubular necrosis	Follicular cystitis	Renal cell carcinoma stage IV		
Aponeurosis contusion	Subacute kidney injury	Fungal cystitis	Rhabdoid tumour of the kidney		
Application site bleeding	Traumatic anuria	Funguria			
Application site bruise	Tubulointerstitial nephritis	Genitourinary chlamydia infection			
Application site bruising	Urate nephropathy	Genitourinary tract gonococcal infection			
Application site haematoma	Urine output decreased	Genitourinary tract infection			
Application site haemorrhage		Granuloma inguinale			
Application site purpura		Infected urinoma			
Arterial haemorrhage		Kidney infection			
Arterial haemorrhage NOS		Klebsiella urinary tract infection			
Arterial intramural haematoma		Nitrite urine present			
Arterial perforation		Parasite urine test positive			
Arterial rupture		Perinephric abscess			
Arterial rupture NOS		Providencia urinary tract infection			
Arteriovenous fistula site haematoma		Purple urine bag syndrome			
Arteriovenous fistula site haemorrhage		Pyelocystitis			
Arteriovenous graft site haematoma		Pyelonephritis			
Arteriovenous graft site haemorrhage		Pyelonephritis acute			
Atrial rupture		Pyelonephritis chronic			
Auricular haematoma					
Basal ganglia haematoma					
Basal ganglia haemorrhage					
Basilar artery perforation					
Battle's sign					
Benign familial haematuria					
Biliary-vascular fistula					
Bladder tamponade					

Procedure-related Bleeding Events (Hemorrhage FMQ)	Acute Kidney Injury (FMQ/SMQ-Narrow)	Renal and Urinary Tract Infections (FMQ-Narrow)	Renal Neoplasms		
			Renal Neoplasms Malignant (HLT)	Renal Pelvis and Ureter Neoplasms Malignant (HLT)	Renal Neoplasms Benign (HLT)
Bladder thrombotic tamponade<br>Bleeding peripartum<br>Bleeding tendency<br>Bleeding varicose vein<br>Blood blister<br>Blood in stool<br>Blood loss anemia<br>Blood urine<br>Blood urine present<br>Bloody airway discharge<br>Bloody discharge<br>Bloody drainage<br>Bloody peritoneal effluent<br>Bone contusion<br>Bone marrow haemorrhage<br>Brain contusion<br>Brain stem haematoma<br>Brain stem haemorrhage<br>Brain stem microhaemorrhage<br>Breast haematoma<br>Breast haemorrhage<br>Broad ligament haematoma<br>Bronchial haemorrhage<br>Bronchial varices haemorrhage<br>Bursal haematoma<br>Cardiac contusion<br>Carotid aneurysm rupture<br>Carotid artery perforation<br>Catheter site bruise<br>Catheter site ecchymosis<br>Catheter site haematoma<br>Catheter site haemorrhage<br>Central nervous system haemorrhage<br>Cephalhaematoma<br>Cerebellar haematoma		Pyelonephritis fungal<br>Pyelonephritis mycoplasmal<br>Pyelonephritis viral<br>Pyonephrosis<br>Pyuria<br>Renal abscess<br>Renal cyst infection<br>Renal echinococciasis<br>Renal graft infection<br>Renal syphilis<br>Renal tuberculosis<br>Schistosomiasis<br>Streptococcal urinary tract infection<br>Tuberculosis bladder<br>Tuberculosis of genitourinary system<br>Urachal sinus infection<br>Ureter abscess<br>Ureteritis<br>Urethral abscess<br>Urethral carbuncle<br>Urethral stricture post infection<br>Urethritis<br>Urethritis chlamydial<br>Urethritis gonococcal<br>Urethritis mycoplasmal<br>Urethritis trichomonal<br>Urethritis ureaplasmal<br>Urinary bladder abscess<br>Urinary meatitis<br>Urinary tract abscess<br>Urinary tract candidiasis			

Procedure-related Bleeding Events (Hemorrhage FMQ)	Acute Kidney Injury (FMQ/SMQ-Narrow)	Renal and Urinary Tract Infections (FMQ-Narrow)	Renal Neoplasms		
			Renal Neoplasms Malignant (HLT)	Renal Pelvis and Ureter Neoplasms Malignant (HLT)	Renal Neoplasms Benign (HLT)
Cerebellar haematoma NOS<br>Cerebellar haemorrhage<br>Cerebellar microhaemorrhage<br>Cerebral aneurysm perforation<br>Cerebral aneurysm ruptured syphilitic<br>Cerebral arteriovenous malformation haemorrhagic<br>Cerebral artery perforation<br>Cerebral cyst haemorrhage<br>Cerebral haematoma<br>Cerebral haemorrhage<br>Cerebral haemorrhage foetal<br>Cerebral haemorrhage neonatal<br>Cerebral microhaemorrhage<br>Cervix haematoma uterine<br>Cervix haemorrhage uterine<br>Chest wall haematoma<br>Choroidal haematoma<br>Choroidal haemorrhage<br>Chronic gastrointestinal bleeding<br>Chronic pigmented purpura<br>Ciliary body haemorrhage<br>Clotted haemothorax<br>Coital bleeding<br>Colitis haemorrhagic<br>Colonic haematoma<br>Colonic haemorrhage<br>Conjunctival haemorrhage<br>Contusion<br>Contusion pulmonary<br>Corneal bleeding<br>Coronary artery atheroma haemorrhage<br>Cullen's sign		Urinary tract infection<br>Urinary tract infection bacterial<br>Urinary tract infection enterococcal<br>Urinary tract infection fungal<br>Urinary tract infection neonatal<br>Urinary tract infection pseudomonal<br>Urinary tract infection staphylococcal<br>Urinary tract infection viral<br>Urogenital infection bacterial<br>Urogenital infection fungal<br>Urogenital trichomoniasis<br>Urosepsis<br>Viral haemorrhagic cystitis<br>Viruria<br>Urinary tract candidiasis			

Procedure-related Bleeding Events (Hemorrhage FMQ)	Acute Kidney Injury (FMQ/SMQ-Narrow)	Renal and Urinary Tract Infections (FMQ-Narrow)	Renal Neoplasms		
			Renal Neoplasms Malignant (HLT)	Renal Pelvis and Ureter Neoplasms Malignant (HLT)	Renal Neoplasms Benign (HLT)
Cystitis haemorrhagic<br>Deep dissecting haematoma<br>Diarrhoea haemorrhagic<br>Diverticulitis intestinal haemorrhagic<br>Diverticulum intestinal haemorrhagic<br>Duodenal haemorrhage<br>Duodenal ulcer haemorrhage<br>Duodenal ulcer haemorrhage, obstructive<br>Duodenitis haemorrhagic<br>Dysfunctional uterine bleeding<br>Ear haemorrhage<br>Ecchymosis<br>Encephalitis haemorrhagic<br>Enteritis haemorrhagic<br>Enterocolitis haemorrhagic<br>Epidural haemorrhage<br>Epistaxis<br>Exsanguination<br>Extra-axial haemorrhage<br>Extradural haematoma<br>Extradural haematoma evacuation<br>Extraischaemic cerebral haematoma<br>Extravasation blood<br>Eye contusion<br>Eye haematoma<br>Eye haemorrhage<br>Eye haemorrhage NOS<br>Eyelid bleeding<br>Eyelid contusion<br>Eyelid haematoma<br>Eyelid haemorrhage					

Procedure-related Bleeding Events (Hemorrhage FMQ)	Acute Kidney Injury (FMQ/SMQ-Narrow)	Renal and Urinary Tract Infections (FMQ-Narrow)	Renal Neoplasms		
			Renal Neoplasms Malignant (HLT)	Renal Pelvis and Ureter Neoplasms Malignant (HLT)	Renal Neoplasms Benign (HLT)
Femoral artery perforation<br>Femoral vein perforation<br>Gardner-Diamond syndrome<br>Gastric haemorrhage<br>Gastric mucosal hypertrophy, haemorrhagic<br>Gastric ulcer haemorrhage<br>Gastric ulcer haemorrhage, obstructive<br>Gastric varices haemorrhage<br>Gastritis alcoholic haemorrhagic<br>Gastritis atrophic haemorrhagic<br>Gastritis haemorrhagic<br>Gastritis haemorrhagic aggravated<br>Gastroduodenal haemorrhage<br>Gastroduodenitis haemorrhagic<br>Gastrointestinal angiodysplasia haemorrhagic<br>Gastrointestinal haemorrhage<br>Gastrointestinal haemorrhage NOS<br>Gastrointestinal organ contusion<br>Gastrointestinal polyp haemorrhage<br>Gastrointestinal ulcer haemorrhage<br>Gastrointestinal vascular malformation haemorrhagic<br>Genital contusion<br>Genital haemorrhage<br>Genital haemorrhage NOS<br>Gingival bleeding					

Procedure-related Bleeding Events (Hemorrhage FMQ)	Acute Kidney Injury (FMQ/SMQ-Narrow)	Renal and Urinary Tract Infections (FMQ-Narrow)	Renal Neoplasms		
			Renal Neoplasms Malignant (HLT)	Renal Pelvis and Ureter Neoplasms Malignant (HLT)	Renal Neoplasms Benign (HLT)
Graft haemorrhage<br>Grey Turner's sign<br>Haemarthrosis<br>Haematemesis<br>Haematidrosis<br>Haematochezia<br>Haematocoele<br>Haematoma<br>Haematoma evacuation<br>Haematoma infection<br>Haematoma muscle<br>Haematoma NOS<br>Haematomyelia<br>Haematosalpinx<br>Haematospermia<br>Haematotympanum<br>Haematuria<br>Haematuria aggravated<br>Haematuria traumatic<br>Haemobilia<br>Haemopericardium<br>Haemoperitoneum<br>Haemophilic pseudotumour<br>Haemoptysis<br>Haemorrhage<br>Haemorrhage coronary artery<br>Haemorrhage foetal<br>Haemorrhage in pregnancy<br>Haemorrhage into ovarian cyst<br>Haemorrhage intracranial<br>Haemorrhage neonatal<br>Haemorrhage NOS<br>Haemorrhage NOS aggravated<br>Haemorrhage NOS foetal<br>Haemorrhage NOS neonatal<br>Haemorrhage subcutaneous					

Procedure-related Bleeding Events (Hemorrhage FMQ)	Acute Kidney Injury (FMQ/SMQ-Narrow)	Renal and Urinary Tract Infections (FMQ-Narrow)	Renal Neoplasms		
			Renal Neoplasms Malignant (HLT)	Renal Pelvis and Ureter Neoplasms Malignant (HLT)	Renal Neoplasms Benign (HLT)
Haemorrhage subepidermal<br>Haemorrhage urinary tract<br>Haemorrhagic adrenal infarction<br>Haemorrhagic anemia<br>Haemorrhagic arteriovenous malformation<br>Haemorrhagic ascites<br>Haemorrhagic breast cyst<br>Haemorrhagic cerebral infarction<br>Haemorrhagic cholecystitis<br>Haemorrhagic cyst<br>Haemorrhagic diathesis<br>Haemorrhagic disease of newborn<br>Haemorrhagic disorder<br>Haemorrhagic erosive gastritis<br>Haemorrhagic hepatic cyst<br>Haemorrhagic infarction<br>Haemorrhagic necrotic pancreatitis<br>Haemorrhagic ovarian cyst<br>Haemorrhagic pneumonia<br>Haemorrhagic stroke<br>Haemorrhagic thyroid cyst<br>Haemorrhagic transformation stroke<br>Haemorrhagic tumour necrosis<br>Haemorrhagic urticaria<br>Haemorrhagic varicella syndrome<br>Haemorrhagic vasculitis<br>Haemorrhoidal haemorrhage<br>Haemothorax<br>Hemorrhagic tumour necrosis					

Procedure-related Bleeding Events (Hemorrhage FMQ)	Acute Kidney Injury (FMQ/SMQ-Narrow)	Renal and Urinary Tract Infections (FMQ-Narrow)	Renal Neoplasms		
			Renal Neoplasms Malignant (HLT)	Renal Pelvis and Ureter Neoplasms Malignant (HLT)	Renal Neoplasms Benign (HLT)
Haemorrhoidal bleeding<br>Henoch-Schonlein purpura<br>Henoch-Schonlein purpura nephritis<br>Hepatic haemangioma rupture<br>Hepatic haematoma<br>Hepatic haemorrhage<br>Hereditary haemorrhagic telangiectasia<br>Hereditary renal microhaematuria<br>Hyperfibrinolysis<br>Hyphaema<br>Idiopathic purpura<br>Ileal haemorrhage<br>Iliac artery perforation<br>Iliac artery rupture<br>Iliac vein perforation<br>Implant site bruising<br>Implant site haematoma<br>Implant site haemorrhage<br>Incision site haematoma<br>Incision site haemorrhage<br>Induced abortion haemorrhage<br>Inferior vena cava perforation<br>Infusion site bruising<br>Infusion site haematoma<br>Infusion site haemorrhage<br>Injection site bruising<br>Injection site haematoma<br>Injection site haemorrhage<br>Instillation site bruise<br>Instillation site haematoma<br>Instillation site haemorrhage<br>Intermenstrual bleeding<br>Internal haemorrhage					

Procedure-related Bleeding Events (Hemorrhage FMQ)	Acute Kidney Injury (FMQ/SMQ-Narrow)	Renal and Urinary Tract Infections (FMQ-Narrow)	Renal Neoplasms		
			Renal Neoplasms Malignant (HLT)	Renal Pelvis and Ureter Neoplasms Malignant (HLT)	Renal Neoplasms Benign (HLT)
Intestinal haematoma<br>Intestinal haemorrhage<br>Intestinal polyp haemorrhage<br>Intestinal stoma site bleeding<br>Intestinal varices haemorrhage<br>Intra-abdominal haematoma<br>Intra-abdominal haemorrhage<br>Intra-abdominal haemorrhage NOS<br>Intracerebral haematoma evacuation<br>Intracerebral haematoma evacuation NOS<br>Intracranial epidural haematoma<br>Intracranial haematoma<br>Intracranial haemorrhage<br>Intracranial haemorrhage NOS<br>Intracranial tumour haemorrhage<br>Intraocular haematoma<br>Intraoperative haemorrhage<br>Intrapartum haemorrhage<br>Intraventricular haemorrhage<br>Intraventricular haemorrhage neonatal<br>Intraventricular haemorrhage NOS<br>Iris haemorrhage<br>Jejunal haemorrhage<br>Joint microhaemorrhage<br>Kidney contusion<br>Lacrimal haemorrhage<br>Large intestinal haemorrhage<br>Large intestinal ulcer haemorrhage					

Procedure-related Bleeding Events (Hemorrhage FMQ)	Acute Kidney Injury (FMQ/SMQ-Narrow)	Renal and Urinary Tract Infections (FMQ-Narrow)	Renal Neoplasms		
			Renal Neoplasms Malignant (HLT)	Renal Pelvis and Ureter Neoplasms Malignant (HLT)	Renal Neoplasms Benign (HLT)
Large intestinal ulcer NOS haemorrhage<br>Laryngeal haematoma<br>Laryngeal haemorrhage<br>Lip haematoma<br>Lip haemorrhage<br>Liver contusion<br>Loin pain haematuria syndrome<br>Lower gastrointestinal haemorrhage<br>Lower limb artery perforation<br>Lymph node haemorrhage<br>Majocchi's purpura<br>Mallory-Weiss syndrome<br>Maxillary sinus haematoma<br>Mediastinal haematoma<br>Mediastinal haemorrhage<br>Medical device site bruise<br>Medical device site haematoma<br>Medical device site haemorrhage<br>Melaena<br>Melaena neonatal<br>Meningorrhagia<br>Menometrorrhagia<br>Menorrhagia<br>Mesenteric haematoma<br>Mesenteric haemorrhage<br>Metrorrhagia<br>Mouth haemorrhage<br>Mucocutaneous haemorrhage<br>Mucosal haemorrhage<br>Mucosal haemorrhage NOS<br>Muscle contusion					

			Renal Neoplasms		
Procedure-related Bleeding Events (Hemorrhage FMQ)	Acute Kidney Injury (FMQ/SMQ-Narrow)	Renal and Urinary Tract Infections (FMQ-Narrow)	Renal Neoplasms Malignant (HLT)	Renal Pelvis and Ureter Neoplasms Malignant (HLT)	Renal Neoplasms Benign (HLT)
Muscle haemorrhage<br>Myocardial haemorrhage<br>Myocardial rupture<br>Naevus haemorrhage<br>Nail bed bleeding<br>Nasal septum haematoma<br>Neonatal gastrointestinal haemorrhage<br>Nephritis haemorrhagic<br>Nipple exudate bloody<br>Ocular retrobulbar haemorrhage<br>Oesophageal haemorrhage<br>Oesophageal intramural haematoma<br>Oesophageal ulcer haemorrhage<br>Oesophageal varices haemorrhage<br>Oesophagitis haemorrhagic<br>Operative haemorrhage<br>Optic disc haemorrhage<br>Optic nerve sheath haemorrhage<br>Oral contusion<br>Oral mucosa haematoma<br>Oral mucosal petechiae<br>Orbital haematoma<br>Osteorrhagia<br>Ovarian haematoma<br>Ovarian haemorrhage<br>Palpable purpura<br>Pancreatic contusion<br>Pancreatic haemorrhage<br>Pancreatitis haemorrhagic<br>Papillary muscle haemorrhage					

Procedure-related Bleeding Events (Hemorrhage FMQ)	Acute Kidney Injury (FMQ/SMQ-Narrow)	Renal and Urinary Tract Infections (FMQ-Narrow)	Renal Neoplasms		
			Renal Neoplasms Malignant (HLT)	Renal Pelvis and Ureter Neoplasms Malignant (HLT)	Renal Neoplasms Benign (HLT)
Paranasal sinus haematoma<br>Paranasal sinus haemorrhage<br>Parathyroid haemorrhage<br>Parotid gland haemorrhage<br>Pelvic haematoma<br>Pelvic haematoma obstetric<br>Pelvic haemorrhage<br>Penile contusion<br>Penile haematoma<br>Penile haemorrhage<br>Peptic ulcer haemorrhage<br>Peptic ulcer haemorrhage, obstructive<br>Perforation of great vessels<br>Pericardial haemorrhage<br>Perineal haematoma<br>Periorbital contusion<br>Periorbital haematoma<br>Periorbital haemorrhage<br>Periosteal haematoma<br>Peripartum haemorrhage<br>Peripheral artery aneurysm rupture<br>Peripheral artery haematoma<br>Perirenal haematoma<br>Peritoneal effusion bloody<br>Peritoneal haematoma<br>Peritoneal haemorrhage<br>Periventricular haemorrhage neonatal<br>Petechiae<br>Pharyngeal haematoma<br>Pharyngeal haemorrhage<br>Pituitary haemorrhage<br>Placenta praevia haemorrhage<br>Pleural haemorrhage					

			Renal Neoplasms		
**Procedure-related Bleeding Events (Hemorrhage FMQ)**	**Acute Kidney Injury (FMQ/SMQ-Narrow)**	**Renal and Urinary Tract Infections (FMQ-Narrow)**	**Renal Neoplasms Malignant (HLT)**	**Renal Pelvis and Ureter Neoplasms Malignant (HLT)**	**Renal Neoplasms Benign (HLT)**
Polymenorrhagia<br>Post abortion haemorrhage<br>Post coital bleeding<br>Post procedural contusion<br>Post procedural haematoma<br>Post procedural haematuria<br>Post procedural haemorrhage<br>Post transfusion purpura<br>Post-menopausal bleeding<br>Post-menopausal haemorrhage<br>Postoperative bruise<br>Postoperative haematoma<br>Postoperative haemorrhage<br>Postpartum haemorrhage<br>Post-partum haemorrhage<br>Post-traumatic punctate intraepidermal haemorrhage<br>Premature separation of placenta<br>Procedural haemorrhage<br>Proctitis haemorrhagic<br>Prostatic haemorrhage<br>Pulmonary alveolar haemorrhage<br>Pulmonary contusion<br>Pulmonary haematoma<br>Pulmonary haemorrhage<br>Pulmonary haemorrhage neonatal<br>Puncture site bruise<br>Puncture site haematoma<br>Puncture site haemorrhage<br>Purpura<br>Purpura cerebri<br>Purpura fulminans<br>Purpura neonatal					

Procedure-related Bleeding Events (Hemorrhage FMQ)	Acute Kidney Injury (FMQ/SMQ-Narrow)	Renal and Urinary Tract Infections (FMQ-Narrow)	Renal Neoplasms		
			Renal Neoplasms Malignant (HLT)	Renal Pelvis and Ureter Neoplasms Malignant (HLT)	Renal Neoplasms Benign (HLT)
Purpura nonthrombocytopenic<br>Purpura non-thrombocytopenic<br>Purpura NOS<br>Purpura senile<br>Putamen haemorrhage<br>Radiation associated haemorrhage<br>Rectal bleeding<br>Rectal haemorrhage<br>Rectal ulcer haemorrhage<br>Red blood cells in urine<br>Red blood cells urine<br>Red blood cells urine positive<br>Renal artery perforation<br>Renal cyst haemorrhage<br>Renal haematoma<br>Renal haemorrhage<br>Respiratory tract haemorrhage<br>Respiratory tract haemorrhage neonatal<br>Respiratory tract haemorrhage NOS<br>Retinal aneurysm rupture<br>Retinal bleeding<br>Retinal haemorrhage<br>Retinopathy haemorrhagic<br>Retroperitoneal haematoma<br>Retroperitoneal haemorrhage<br>Retroplacental haematoma<br>Ruptured cerebral aneurysm<br>Schamberg's disease<br>Scleral haemorrhage<br>Scrotal haematocoele<br>Scrotal haematoma<br>Scrotal haemorrhage<br>Shock haemorrhagic					

Procedure-related Bleeding Events (Hemorrhage FMQ)	Acute Kidney Injury (FMQ/SMQ-Narrow)	Renal and Urinary Tract Infections (FMQ-Narrow)	Renal Neoplasms		
			Renal Neoplasms Malignant (HLT)	Renal Pelvis and Ureter Neoplasms Malignant (HLT)	Renal Neoplasms Benign (HLT)
Skin bleeding<br>Skin haemorrhage<br>Skin neoplasm bleeding<br>Skin ulcer haemorrhage<br>Small intestinal haemorrhage<br>Small intestinal ulcer haemorrhage<br>Small intestinal ulcer NOS haemorrhage<br>Soft tissue haemorrhage<br>Spermatic cord haemorrhage<br>Spinal cord haematoma<br>Spinal cord haemorrhage<br>Spinal epidural haematoma<br>Spinal epidural haemorrhage<br>Spinal haematoma<br>Spinal subarachnoid haemorrhage<br>Spinal subdural haematoma<br>Spinal subdural haemorrhage<br>Spleen contusion<br>Splenic artery perforation<br>Splenic haematoma<br>Splenic haemorrhage<br>Splenic varices haemorrhage<br>Splinter haemorrhages<br>Spontaneous haematoma<br>Spontaneous haemorrhage<br>Spontaneous hyphaema<br>Stoma site haemorrhage<br>Stomatitis haemorrhagic<br>Subarachnoid haematoma<br>Subarachnoid haemorrhage<br>Subarachnoid haemorrhage NOS					

Procedure-related Bleeding Events (Hemorrhage FMQ)	Acute Kidney Injury (FMQ/SMQ-Narrow)	Renal and Urinary Tract Infections (FMQ-Narrow)	Renal Neoplasms		
			Renal Neoplasms Malignant (HLT)	Renal Pelvis and Ureter Neoplasms Malignant (HLT)	Renal Neoplasms Benign (HLT)
Subcapsular hepatic haematoma					
Subcapsular renal haematoma					
Subcapsular splenic haematoma					
Subchorionic haematoma					
Subchorionic haemorrhage					
Subcutaneous haematoma					
Subdural haematoma					
Subdural haematoma evacuation					
Subendocardial haemorrhage					
Subgaleal haematoma					
Subgaleal haemorrhage					
Subretinal haematoma					
Sudural haematoma evacuation					
Thrombocytopenic purpura					
Thrombotic thrombocytopenic purpura					
Tongue haematoma					
Tooth pulp haemorrhage					
Traumatic haematoma					
Traumatic intracranial haematoma					
Umbilical haematoma					
Urinary bladder haematoma					
Urinary occult blood positive					
Urticaria haemorrhagica					
Uterine haematoma					
Vaccination site bruising					
Vaccination site haematoma					
Vaccination site haemorrhage					
Vaginal haematoma					
Vaginal haemorrhage					
Vascular access site bruising					
Vascular access site contusion					

Procedure-related Bleeding Events (Hemorrhage FMQ)	Acute Kidney Injury (FMQ/SMQ-Narrow)	Renal and Urinary Tract Infections (FMQ-Narrow)	Renal Neoplasms		
			Renal Neoplasms Malignant (HLT)	Renal Pelvis and Ureter Neoplasms Malignant (HLT)	Renal Neoplasms Benign (HLT)
Vascular access site haematoma<br>Vascular access site haemorrhage<br>Vascular graft haemorrhage<br>Vascular purpura<br>Vessel puncture site bruise<br>Vessel puncture site haematoma<br>Vitreous haematoma<br>Vulval haematoma<br>Vulval haematoma evacuation<br>Withdrawal bleeding irregular<br>Wound haematoma<br>Hepatic artery haemorrhage<br>Heidelberg classification<br>Intracranial haemorrhage neonatal<br>Vaginal tamponade					

docusign

Certificate Of Completion

Envelope Id: 23B2EA6F-80C0-4FEB-B18C-9FCFF5DF15F3

Status: Completed

Subject: Complete with Docusign: regen-007-sap-v1.0-05Jun2025.pdf

Source Envelope:

Document Pages: 100	Signatures: 4	Envelope Originator:
Certificate Pages: 5	Initials: 0	Connie Barysauskas
AutoNav: Enabled		3929 Westpoint Blvd., Suite G
EnvelopeId Stamping: Disabled		Winston Salem, NC 27103
Time Zone: (UTC-05:00) Eastern Time (US & Canada)		connie.barysauskas@prokidney.com
		IP Address: 2601:18c:8400:1

Record Tracking

Status: Original<br>05 June 2025 | 10:59	Holder: Connie Barysauskas<br>connie.barysauskas@prokidney.com	Location: DocuSign

Signer Events / Signature / Timestamp

Signer Events	Signature	Timestamp
Connie Barysauskas<br>connie.barysauskas@prokidney.com<br>AD, Biostatistics<br>ProKidney<br>Security Level: Email, Account Authentication (Required)	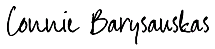<br>Signature Adoption: Pre-selected Style<br>Signature ID:<br>36B9E414-B166-4577-B425-C81A9376A78B<br>Using IP Address: 73.167.156.14<br><br>With Signing Authentication via Docusign password<br>With Signing Reasons (on each tab):<br>I approve this document	Sent: 05 June 2025 | 11:02<br>Viewed: 05 June 2025 | 11:02<br>Signed: 05 June 2025 | 11:05

Electronic Record and Signature Disclosure:
Not Offered via Docusign

Signer Events	Signature	Timestamp
Hongxia Yan<br>hongxia.yan@prokidney.com<br>Principal Biostatistician<br>ProKidney<br>Security Level: Email, Account Authentication (Required)	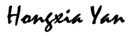<br>Signature Adoption: Pre-selected Style<br>Signature ID:<br>768D9C9B-5558-4A0A-B90D-1D5001C20B3D<br>Using IP Address: 216.122.175.100<br><br>With Signing Authentication via Docusign password<br>With Signing Reasons (on each tab):<br>I am the author of this document	Sent: 05 June 2025 | 11:02<br>Viewed: 05 June 2025 | 11:06<br>Signed: 05 June 2025 | 11:06

Electronic Record and Signature Disclosure:
Accepted: 06 February 2025 | 14:32
ID: 869b48f1-2ffd-4846-b497-b22241e752a3

Signer Events	Signature	Timestamp
Lauren Weintraub<br>lauren.weintraub@prokidney.com<br>Medical Director Contractor<br>ProKidney<br>Security Level: Email, Account Authentication (Required)	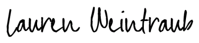<br>Signature Adoption: Pre-selected Style<br>Signature ID:<br>97811429-2063-4775-9C05-0BA6055E7938<br>Using IP Address: 98.204.31.219<br><br>With Signing Authentication via Docusign password<br>With Signing Reasons (on each tab):<br>I approve this document	Sent: 05 June 2025 | 11:02<br>Viewed: 05 June 2025 | 11:23<br>Signed: 05 June 2025 | 11:23
**Electronic Record and Signature Disclosure:**<br>Not Offered via Docusign		
Lillie Wang<br>lillie.wang@prokidney.com<br>Security Level: Email, Account Authentication (Required)	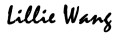<br>Signature Adoption: Pre-selected Style<br>Signature ID:<br>3CB9DFBD-7468-4E8E-9187-54A947148B32<br>Using IP Address: 216.122.175.100<br><br>With Signing Authentication via Docusign password<br>With Signing Reasons (on each tab):<br>I approve this document	Sent: 05 June 2025 | 11:02<br>Viewed: 05 June 2025 | 11:11<br>Signed: 05 June 2025 | 11:13
**Electronic Record and Signature Disclosure:**<br>Accepted: 12 December 2023 | 17:25<br>ID: 5d3e8b62-9a97-439d-aafa-be44b7f7d43f		

In Person Signer Events	Signature	Timestamp

Editor Delivery Events	Status	Timestamp

Agent Delivery Events	Status	Timestamp

Intermediary Delivery Events	Status	Timestamp

Certified Delivery Events	Status	Timestamp

Carbon Copy Events	Status	Timestamp

Witness Events	Signature	Timestamp

Notary Events	Signature	Timestamp

Envelope Summary Events	Status	Timestamps
Envelope Sent	Hashed/Encrypted	05 June 2025 | 11:02
Certified Delivered	Security Checked	05 June 2025 | 11:11
Signing Complete	Security Checked	05 June 2025 | 11:13
Completed	Security Checked	05 June 2025 | 11:23

Payment Events	Status	Timestamps

Electronic Record and Signature Disclosure

ELECTRONIC RECORD AND SIGNATURE DISCLOSURE

From time to time, Prokidney - Part-11 (we, us or Company) may be required by law to provide to you certain written notices or disclosures. Described below are the terms and conditions for providing to you such notices and disclosures electronically through the DocuSign system. Please read the information below carefully and thoroughly, and if you can access this information electronically to your satisfaction and agree to this Electronic Record and Signature Disclosure (ERSD), please confirm your agreement by selecting the check-box next to 'I agree to use electronic records and signatures' before clicking 'CONTINUE' within the DocuSign system.

Getting paper copies

At any time, you may request from us a paper copy of any record provided or made available electronically to you by us. You will have the ability to download and print documents we send to you through the DocuSign system during and immediately after the signing session and, if you elect to create a DocuSign account, you may access the documents for a limited period of time (usually 30 days) after such documents are first sent to you. After such time, if you wish for us to send you paper copies of any such documents from our office to you, you will be charged a $0.00 per-page fee. You may request delivery of such paper copies from us by following the procedure described below.

Withdrawing your consent

If you decide to receive notices and disclosures from us electronically, you may at any time change your mind and tell us that thereafter you want to receive required notices and disclosures only in paper format. How you must inform us of your decision to receive future notices and disclosure in paper format and withdraw your consent to receive notices and disclosures electronically is described below.

Consequences of changing your mind

If you elect to receive required notices and disclosures only in paper format, it will slow the speed at which we can complete certain steps in transactions with you and delivering services to you because we will need first to send the required notices or disclosures to you in paper format, and then wait until we receive back from you your acknowledgment of your receipt of such paper notices or disclosures. Further, you will no longer be able to use the DocuSign system to receive required notices and consents electronically from us or to sign electronically documents from us.

All notices and disclosures will be sent to you electronically

Unless you tell us otherwise in accordance with the procedures described herein, we will provide electronically to you through the DocuSign system all required notices, disclosures, authorizations, acknowledgements, and other documents that are required to be provided or made available to you during the course of our relationship with you. To reduce the chance of you inadvertently not receiving any notice or disclosure, we prefer to provide all of the required notices and disclosures to you by the same method and to the same address that you have given us. Thus, you can receive all the disclosures and notices electronically or in paper format through the paper mail delivery system. If you do not agree with this process, please let us know as described below. Please also see the paragraph immediately above that describes the consequences of your electing not to receive delivery of the notices and disclosures electronically from us.

How to contact Prokidney - Part-11:

You may contact us to let us know of your changes as to how we may contact you electronically, to request paper copies of certain information from us, and to withdraw your prior consent to receive notices and disclosures electronically as follows:
To contact us by email send messages to: Dominique.Ferri@prokidney.com

To advise Prokidney - Part-11 of your new email address

To let us know of a change in your email address where we should send notices and disclosures electronically to you, you must send an email message to us at Dominique.Ferri@prokidney.com and in the body of such request you must state: your previous email address, your new email address. We do not require any other information from you to change your email address.

If you created a DocuSign account, you may update it with your new email address through your account preferences.

To request paper copies from Prokidney - Part-11

To request delivery from us of paper copies of the notices and disclosures previously provided by us to you electronically, you must send us an email to Dominique.Ferri@prokidney.com and in the body of such request you must state your email address, full name, mailing address, and telephone number. We will bill you for any fees at that time, if any.

To withdraw your consent with Prokidney - Part-11

To inform us that you no longer wish to receive future notices and disclosures in electronic format you may:

i. decline to sign a document from within your signing session, and on the subsequent page, select the check-box indicating you wish to withdraw your consent, or you may;

ii. send us an email to Dominique.Ferri@prokidney.com and in the body of such request you must state your email, full name, mailing address, and telephone number. We do not need any other information from you to withdraw consent.. The consequences of your withdrawing consent for online documents will be that transactions may take a longer time to process..

Required hardware and software

The minimum system requirements for using the DocuSign system may change over time. The current system requirements are found here: https://support.docusign.com/guides/signer-guide-signing-system-requirements.

Acknowledging your access and consent to receive and sign documents electronically

To confirm to us that you can access this information electronically, which will be similar to other electronic notices and disclosures that we will provide to you, please confirm that you have read this ERSD, and (i) that you are able to print on paper or electronically save this ERSD for your future reference and access; or (ii) that you are able to email this ERSD to an email address where you will be able to print on paper or save it for your future reference and access. Further, if you consent to receiving notices and disclosures exclusively in electronic format as described herein, then select the check-box next to 'I agree to use electronic records and signatures' before clicking 'CONTINUE' within the DocuSign system.

By selecting the check-box next to 'I agree to use electronic records and signatures', you confirm that:

- You can access and read this Electronic Record and Signature Disclosure; and
- You can print on paper this Electronic Record and Signature Disclosure, or save or send this Electronic Record and Disclosure to a location where you can print it, for future reference and access; and
- Until or unless you notify Prokidney - Part-11 as described above, you consent to receive exclusively through electronic means all notices, disclosures, authorizations, acknowledgements, and other documents that are required to be provided or made available to you by Prokidney - Part-11 during the course of your relationship with Prokidney - Part-11.

A PHASE 2, RANDOMIZED, OPEN-LABEL, REPEAT DOSE, SAFETY AND EFFICACY STUDY OF RENAL AUTOLOGOUS CELL THERAPY (REACT) IN SUBJECTS WITH TYPE 1 or 2 DIABETES AND CHRONIC KIDNEY DISEASE (REGEN-007)

s

Protocol Number:	REGEN-007
IND Number:	16482
Development Phase:	Phase 2
Investigational Therapy Name:	Renal Autologous Cell Therapy (REACT)
Brief Description:	Multi-center, prospective, open-label, double-arm, randomized, redose study whereby eligible subjects will be randomized 1:1 after kidney biopsy to 1 of 2 cohorts. Cohort 1 subjects will receive 2 REACT injections in the biopsied and non-biopsied contralateral kidneys 3 months apart (+60 days). Cohort 2 subjects will receive 1 REACT injection into the biopsied kidney and if a pre-defined trigger is met, will undergo a second REACT injection into the contralateral kidney. Both cohorts will be followed for at least 18 months after the final REACT injection. If a Cohort 2 subject does not meet a trigger, the subject will be followed for at least 18 months after the first injection.
Version Number:	3.0
Date of Issue:	31 January 2023
Sponsor:	ProKidney<br>8020 Arco Corporate Dr. Suite 400<br>Raleigh, NC 27617<br>(919) 294-4521<br>Email: *info@ProKidney.com*
Medical Monitor:	PPD<br>3900 Paramount Pkwy<br>Morrisville, North Carolina 27560, USA
24 Hour Safety Hotline:	IQVIA Safety<br>Phone: +1 (855) 564-2229<br>Fax: +1 (855) 638 1674

This document is a confidential communication from ProKidney. Acceptance of this document constitutes an agreement by the recipient(s) that no unpublished information contained herein will be published or disclosed without prior written approval from ProKidney, except that this document may be disclosed to appropriate Institutional Review Boards (IRBs) or Ethics Committees (EC) under the condition that they are also required to maintain confidentiality.

SIGNATURE PAGE

A Phase 2, Randomized, Open-Label, Repeat Dose, Safety and Efficacy Study of Renal Autologous Cell Therapy (REACT) in Subjects with Type 1 or 2 Diabetes and Chronic Kidney Disease (REGEN-007)
Protocol Version 3.0

By signing below, I agree to the following:

- ✓ I have received and read Protocol REGEN-007: A PHASE 2, RANDOMIZED, OPEN-LABEL, REPEAT DOSE, SAFETY AND EFFICACY STUDY OF RENAL AUTOLOGOUS CELL THERAPY (REACT) IN SUBJECTS WITH TYPE 1 OR 2 DIABETES AND CHRONIC KIDNEY DISEASE (REGEN-007)
- ✓ In my formal capacity as Investigator, I understand that my duties include ensuring the safety of all study subjects as well as conducting the study in accordance with all stipulations of the protocol as specified in both the clinical and administrative sections, including all statements regarding confidentiality.
- ✓ I understand that no deviation from, or changes to the Protocol will take place without prior agreement from the Sponsor and documented approval from the Institutional Review Board or Ethics Committee, except where necessary to eliminate an immediate hazard(s) to the study subjects.
- ✓ This study will be conducted in compliance with the protocol, in accordance with ICH E6 Harmonized Tripartite Guideline (ICH-GCP), in general agreement with the most recent version of the Declaration of Helsinki, and in accordance with all applicable United States and European regulations.
- ✓ I agree to ensure that all staff members at this site who are involved in the conduct of this study understand their obligations in meeting the above commitments.

____
Printed Name of Investigator

______________________________ ______________
Signature of Investigator **Date**

Ashley H. Johns
Printed Name of Sponsor's Representative

Ashley H. Johns 31 January 2023
Signature of Sponsor's Representative **Date**

EMERGENCY CONTACT INFORMATION

Role in Study	Name	Address and Telephone Number
Sponsor	ProKidney	ProKidney<br>8020 Arco Corporate Dr, Suite 400<br>Raleigh, NC 27617<br>Phone: (919) 294-4521<br>Email: info@prokidney.com
Medical Monitor	PPD	PPD<br>3900 Paramount Pkwy<br>Morrisville, North Carolina 27560, USA
24-Hour Emergency Contact	PPD	Phone: +1 (888) 483-7729<br>Fax: +1 (888) 529-3580<br>+1 (919) 654-3836
24-Hour Safety Contact	IQVIA	Phone: +1 (855) 564-2229<br>Fax: +1 (855) 638-1674<br>Email: LS_REACT_SO@IQVIA.com
Manufacturer	ProKidney, LLC	ProKidney, LLC<br>3929 Westpoint Blvd, Suite G<br>Winston-Salem, NC 27103<br>Phone: (336) 448-2843<br>Email: REACT@prokidney.com

PROTOCOL SYNOPSIS

<table>
<tr><td colspan="2">Name of Sponsor/Company: ProKidney</td></tr>
<tr><td colspan="2">Name of Investigational Product: Renal Autologous Cell Therapy (REACT)</td></tr>
<tr><td colspan="2">Title of Study: A Phase 2, Randomized, Open-Label, Repeat Dose, Safety and Efficacy Study of Renal Autologous Cell Therapy (REACT) in Subjects with Type 1 or 2 Diabetes and Chronic Kidney Disease (REGEN-007)</td></tr>
<tr><td colspan="2">Name of Active Ingredient: Renal Autologous Cell Therapy (REACT) consists of autologous selected renal cells (SRC) previously obtained from a participant’s renal biopsy tissue and culture expanded at a ProKidney Good Manufacturing Practices (GMP) facility.</td></tr>
<tr><td colspan="2">Study Center(s): up to 10 sites</td></tr>
<tr><td>Studied Period (months): 29 up to 42 months
• Cohort 1 (up to 29 months): Screening (60 days), biopsy to first REACT injection (12 weeks +30 days), second REACT injection (3 months [+60 days] after the first REACT injection) in contralateral kidney, 18-month follow-up after the last REACT injection.
• Cohort 2 (29 up to 42 months): Screening (60 days), biopsy to first REACT injection (12 weeks +30 days). A possible second REACT injection upon meeting a redose trigger (renal function with sustainment occurring between 3-15 months following the first REACT injection). A second injection would be within at least 30 days of meeting a renal function redose trigger with sustainment with at least 18-month follow-up after last REACT injection.</td><td>Phase of Development: 2</td></tr>
<tr><td colspan="2">Objective:
The objective of this study is to assess the safety, efficacy, and durability of up to 2 REACT injections delivered percutaneously into biopsied and non-biopsied contralateral kidneys on renal function progression in 2 different cohorts of subjects with type 1 diabetes mellitus (T1DM) or type 2 diabetes mellitus (T2DM) diabetes and chronic kidney disease (CKD):
• Cohort 1: two scheduled REACT injections given 3 months apart (+60 days) with at least 18-month follow-up.
• Cohort 2: one scheduled REACT injection with a possible second REACT injection upon meeting a redose trigger (renal function with sustainment occurring between 3-15 months following the first REACT injection). A second injection would be within at least 30 days of meeting a renal function redose trigger with sustainment with at least 18-month follow-up after last REACT injection.
Primary Efficacy Endpoint:
• Change from pre-injection to post-last injection total (acute + chronic) slope of estimated glomerular filtration rate (eGFR) using the 2009 Chronic Kidney Disease Epidemiology Collaboration (CKD-EPI 2009) serum creatinine equation.</td></tr>
</table>

Primary Safety Endpoint:

- Procedure and investigational product-related treatment-emergent adverse events (TEAEs) obtained through at least 18 months after the last REACT injection.

Secondary Efficacy Endpoints:

1. Estimated glomerular filtration rates, determined using CKD-EPI 2009, from first injection to at least 18 months after the last REACT injection.
2. Time from first injection to at least 40% reduction in eGFR, using the 2009 CKD-EPI serum creatinine equation, sustained for 30 days.
3. Time from first injection to eGFR <15 mL/min/1.73m² using the 2009 CKD-EPI serum creatinine equation, sustained for 30 days and/or chronic dialysis, and/or renal transplant.
4. Time from first injection to increase of UACR of at least 30% and of at least 30 mg/g, using the random urine microalbumin/urine creatinine ratio sustained for 90 days.
5. Time from first injection to renal or cardiovascular death.
6. Time from first injection to earliest of the following:
    - At least 40% reduction in eGFR, using the 2009 CKD-EPI serum creatinine equation, sustained for 30 days or
    - eGFR <15 mL/min/1.73m² using the 2009 CKD-EPI serum creatinine equation, sustained for 30 days and/or chronic dialysis, and/or renal transplant or
    - Increase of UACR of at least 30% and of at least 30 mg/g, using the random urine microalbumin/urine creatinine ratio sustained for 90 days or
    - Renal or cardiovascular death.
7. Time from first injection to earliest of the following:
    - At least 40% reduction in eGFR, using the 2009 CKD-EPI serum creatinine equation, sustained for 30 days or
    - eGFR <15 mL/min/1.73m² using the 2009 CKD-EPI serum creatinine equation, sustained for 30 days and/or chronic dialysis, and/or renal transplant or
    - Renal or cardiovascular death.
8. Percent of subjects who have the same or reduced 5-year risk of ESRD and 2 year risk of ESRD at 12 and 18 months after the last injection.

Secondary Safety Endpoint:

- Procedure related death.

Exploratory Endpoints:

1. Changes from baseline in renal volume.
2. Changes from baseline in cortical thickness.
3. Changes from baseline in hemoglobin.
4. Changes from baseline in hematocrit.
5. Changes from baseline in calcium.
6. Changes from baseline in phosphorus.
7. Changes from baseline in parathyroid hormone.
8. Changes from baseline in systolic blood pressure.
9. Changes from baseline in diastolic blood pressure.
10. Changes from baseline in potassium.
11. Changes from baseline in bicarbonate.
12. The time from first injection to all-cause mortality.
13. Incidence of changes in antihypertensive therapy (i.e., increase, decrease).
14. Time to increase in antihypertensive therapy.
15. Incidence of changes in metabolic acidosis therapy (i.e., increase, decrease).
16. Time to increase in metabolic acidosis therapy.
17. Incidence of changes in hyperkalemia therapy (i.e., increase, decrease).
18. Time to increase in hyperkalemia therapy.
19. Incidence of changes in anemia therapy (i.e., increase, decrease).
20. Time to increase in anemia therapy.
21. Incidence of changes in bone and mineral dysmetabolism therapy (i.e., increase, decrease).
22. Time to increase in bone and mineral dysmetabolism therapy.

Methodology: Phase 2, prospective, open-label, randomized, repeat dose study

Number of subjects (planned): up to 50 subjects will be treated with REACT. As this is a Phase 2 exploratory safety and efficacy study, robust statistical analysis will not be performed. Therefore, the sample size proposed for this study is a size typical for an exploratory study, allowing for identification of safety outcomes in a limited population.

Diagnosis and main criteria for inclusion: Male or female who is 30 to 80 years of age, has T1DM or T2DM and CKD, with an eGFR between 20 and 50 mL/min/1.73m^2 inclusive.

Inclusion Criteria: Unless otherwise noted, subjects must satisfy each inclusion criterion to participate in the study. Inclusion criteria will be assessed at the Screening Visit.

1. Male or female aged 30 to 80 years, inclusive, on the date informed consent is signed.
2. Clinical diagnosis of T1DM or T2DM, controlled per institutional standard of care.
3. The subject has a clinical diagnosis of diabetic nephropathy as the underlying cause of renal disease (diagnosis does not have to be confirmed via renal biopsy).
4. The subject has a serum glycosylated hemoglobin (HbA_{1c}) level less than 10% at the Screening Visit.

5. The subject has a documented clinical diagnosis of an eGFR between 20 and 50 mL/min/1.73m^2 inclusive not requiring renal dialysis.
6. The subject has a urinary albumin-to-creatinine ratio (UACR) of greater than or equal to 30 and less than or equal to 5,000 mg/g.
7. The subject has stable blood pressure and is maintained on a stable anti-hypertensive medication regimen, if treatment for hypertension is necessary. If treatment includes an angiotensin-converting enzyme inhibitor (ACEi) or an angiotensin receptor blocker (ARB), that treatment must be the maximum tolerated daily dose for at least 4 weeks prior to randomization or, if the treatment includes a sodium glucose cotransporter 2 inhibitor (SGLT2i) at any dose, for at least 4 weeks prior to randomization.
   Note: A maximum tolerated daily dose of an ACEi or ARB is defined as the maximum tolerated dose for diabetic nephropathy (for agents with an approved indication for diabetic nephropathy in patients with T1DM or T2DM, i.e., losartan and irbesartan) or the maximum tolerated dose for hypertension (for agents without an approved indication for diabetic nephropathy), unless side effects or adverse events (AE) limit the use of the maximum dose. For subjects who are not on a maximum daily dose of an ACEi or ARB, Investigators will be required to document why a higher dose is contraindicated.
8. A minimum of 3 measurements of eGFR (by sCr or cystatin C) must be obtained at least 3 months apart within 24 months prior to the Screening Visit to define the rate of progression of CKD.
9. The subject agrees and is able to refrain from nonsteroidal anti-inflammatory drugs (NSAIDs), including aspirin, clopidogrel, prasugrel, dipyridamole, and other platelet aggregation inhibitors during the period beginning 7 days before through 7 days following the percutaneous renal biopsy and REACT injection(s).
   Note: Aspirin, at a dose of up to 100 mg/day, may be continued if doing so is the standard of care at the investigational site, is accepted for primary prevention of heart disease in subjects with diabetes who are older than 40 years of age or have additional risk factors for cardiovascular disease or stroke, and for whom the perceived benefits of aspirin therapy outweigh the risks associated with treatment.
10. The subject agrees and is able to refrain from oral ingestion of fish oil supplements during the period beginning 7 days before through 7 days following the percutaneous renal biopsy and REACT injection(s).
11. The subject is willing and able to cooperate with all aspects of the protocol.
12. The subject is willing and able to provide signed informed consent.

Exclusion Criteria: Subjects who satisfy any exclusion criterion listed below are not eligible to participate in the study. Exclusion criteria will be assessed at the Screening Visit.

1. The subject has a history of renal transplantation.
2. The subject has a mean systolic blood pressure greater than or equal to 140 mmHg and/or mean diastolic blood pressure greater than or equal to 90 mmHg at screening. Subjects with blood pressure outside of this range prior to biopsy/injection may continue if approved by the Medical Monitor.
3. The subject has hemoglobin levels less than 10 g/dL and is not responsive to the standard medical intervention for CKD-related anemia prior to randomization.

4. The subject appears to be at possibly increased risk of either thromboembolism or bleeding because of abnormal results at the Screening Visit for any of the following tests: activated partial thromboplastin time (APTT), prothrombin time-international normalized ratio (PT-INR), and platelet count.
5. The subject has a bleeding disorder(s) or is maintained on any anticoagulant agents, including fractionated heparin preparations, Coumadin® (warfarin), or direct thrombin inhibitors, that cannot be discontinued for 7 days before and 7 days after biopsy or injections.
6. The subject has a known allergy or contraindication(s), has experienced severe systemic reaction(s) to kanamycin/structurally similar aminoglycoside antibiotic(s), which may be a manufacturing process residual, or has a known hypersensitivity to dimethyl sulfoxide.
7. The subject has a history of anaphylactic or severe systemic reaction(s) or contraindication(s) to human blood products, Dextran-40, or materials of animal origin (e.g., bovine).
8. The subject is not a good candidate to undergo percutaneous REACT injection, in the judgment of the interventionalist or proceduralist who will perform the procedure. This includes confirming the subject has contraindications for undergoing the procedure based on depth of the kidneys, positioning limitations, and if the kidneys are greater than 15 cm from skin surface to kidney capsule, or has only one kidney.
9. The subject has a history of severe systemic reaction(s) or any contraindication to local anesthetics or sedatives.
10. The subject has a history of complicated recurrent urinary tract infections or renal stone disease.
11. The subject has been diagnosed with acute kidney injury within 3 months of the Screening Visit.
12. The subject has any of the following conditions: autosomal dominant and recessive polycystic kidney disease, focal segmental glomerulosclerosis, vasculitis -related CKD, IgA or IgG nephropathy, drug-induced or hypertension-induced CKD and other types of CKD as determined by the Investigator that would interfere with biopsy and REACT injection procedure (such as horseshoe kidney variant and unexplained hydronephrosis), any other documented renal pathology that would interfere with the REACT injection procedure.
    Note: Anatomic abnormalities and benign conditions are not exclusionary if the kidney remains accessible and meets the criteria to receive the REACT injection.
13. The subject has poor diabetes control as evaluated by the Investigator, including, but not limited to, a history of diabetic ketoacidosis (DKA) in the year prior to screening, frequent hypoglycemic episodes, or hypoglycemia unawareness.
14. The subject is awaiting a pancreas transplant.
15. The subject has incapacitating cardiac neurologic, peripheral vascular, or pulmonary disorders as determined by the Principal Investigator.
16. The subject has a history of malignancy within the past 3 years (exceptions: squamous and basal cell carcinomas of the skin and carcinoma of the cervix in situ, or a

malignancy that in the opinion of the Investigator, along with the Medical Monitor, is considered treated with minimal risk of recurrence).

17. The subject has documented clinical diagnosis of chronic hepatic disease (alanine aminotransferase [ALT] or aspartate aminotransferase [AST] greater than 3 times the upper limit of normal) at the Screening Visit.
18. The subject has a positive test result for the hepatitis B virus (HBV) surface antigen, a positive test result for hepatitis C virus (HCV) antibodies, or a positive test result for human immunodeficiency virus (HIV) antibodies.
    Note: At the discretion of the Investigator, a subject who gives a history of a treated and cured HCV infection may be screened with a test for viral ribonucleic acid (RNA) and, if a cure is demonstrated, the subject may be enrolled.
19. The subject has a documented clinical diagnosis of active tuberculosis (TB) requiring treatment.
20. The subject is immunocompromised or is receiving immunosuppressive agents, including individuals treated for chronic glomerulonephritis within 3 months of the Screening Visit.
    Note: Inhaled corticosteroids, chronic low-dose corticosteroids (less than or equal to 7.5 mg prednisone equivalent per day), and brief pulsed corticosteroids for intermittent symptoms (e.g., asthma) are permitted.
21. The female subject is pregnant, lactating (breast feeding), or planning a pregnancy during the course of the study. Or the female subject is of childbearing potential and is not using a highly effective method(s) of birth control, including sexual abstinence. Or the female subject is unwilling to continue using a highly effective method of birth control throughout the duration of the study.
    Note: A highly effective method of birth control is defined as one that results in a low failure rate (i.e., less than one percent per year) when used consistently and correctly, such as implants, injectables, combined oral contraceptives, some intrauterine devices, sexual abstinence, or a vasectomized partner.
22. The subject has a history of active alcohol and/or drug abuse that, in the judgment of the Investigator, would impair the subject's ability to comply with the protocol.
23. The subject's health status would, in the judgment of the Investigator, be jeopardized by participating in the study.
24. The subject has used an investigational product within 3 months prior to the Screening Visit without receiving written consent from the study assigned Medical Monitor.
25. The subject has previously received treatment with REACT.

Investigational Product, Dosage, and Mode of Administration:
Investigational Product: REACT is made from cell culture expanded autologous SRC obtained from each individual subject's kidney biopsy. To manufacture REACT, biopsy tissue from each enrolled subject will be sent to ProKidney, in whose facilities renal cells will be cell culture expanded and SRC selected in compliance with current GMP. REACT is a frozen formulation of SRC in cryopreservation media at a concentration of approximately 100×10^6 cells/mL REACT is stored at less than or equal to -150°C until use.

Dosage: Based on Phase 1 and 2 data to date, the dose of REACT will be 3×10^6 cells/g estimated kidney weight (g KW^{est}). Since the concentration of SRC per mL of REACT is

100 × 10^6 cells/mL, the dosing volume will be 3.0 mL for each 100 g of kidney weight. Using this dosing paradigm, the following table shows the dosing volume and number of SRC to be delivered relative to estimated kidney weight. The maximum volume of REACT injected into the kidney during an injection will be 8.0 mL.

Investigational Product, Dosage and Mode of Administration: Dosage (continued)

Estimated Kidney Weight (g KW^{est})*		Dosing Volume (mL)	SRC Delivered (Number of Cells × 10^6)
**Median Weight (g)**	**Weight Range (g)**		
100	95 – 108	3.0	300
117	109 – 125	3.5	350
133	126 – 141	4.0	400
150	142 – 158	4.5	450
167	159 – 175	5.0	500
183	176 – 191	5.5	550
200	192 – 208	6.0	600
217	209 – 225	6.5	650
233	226 – 241	7.0	700
250	242 – 258	7.5	750
— — —	>259	8.0	800

*Kidney weight will be estimated from the results of an MRI study performed on or after the Screening Visit until Day 0 (renal biopsy). If a subject cannot undergo MRI, then computed tomography (CT) will be substituted to obtain kidney size and volume based on standardized analysis.

Mode of Administration: REACT will be injected into the locoregional kidney cortex using a percutaneous computed tomography (CT) image guided approach. The percutaneous method will employ a standardized interventional guided technique. Proceduralists will receive specific training and credentialing in various components of the REACT administration process. In subjects receiving a second REACT injection, the second injection will be performed in the contralateral kidney (i.e., the kidney that did not receive the first injection).

Reference Therapy or Control Group:

Subjects will be randomized (1:1) to either Cohort 1 or Cohort 2 once subject eligibility is confirmed.

- Cohort 1 (two scheduled REACT injections, at least 3 months apart): Subjects in Cohort 1 will receive their first REACT injection as soon as the REACT product is manufactured into the biopsied kidney and released for shipment to the clinical site. After 3 months (+60 days), a second injection will be given, in the contralateral kidney (i.e., the kidney that was not injected with the first injection). Subjects will be followed every 3 months for at least 18 months after the last REACT injection.
- Cohort 2 (one scheduled REACT injection with trigger-based redose criteria): Subjects in Cohort 2 will receive their first REACT injection as soon as the REACT product is manufactured into the biopsied kidney and released for shipment to the clinical site, then undergo an observation period to see whether a redose trigger is met. They will be followed every 3 months for at least 18 months after the last REACT injection. A trigger for redose will be assessed between 3 months and 15 months after first REACT injection. If one of the following redose triggers is met, they will receive their second REACT injection within at least 30 days of meeting trigger sustainment criteria. The

second injection will be given in the contralateral kidney. If a redose trigger is not met within 15 months after the first REACT injection, subjects will complete their participation in this study having received only one REACT injection.<br>o Redose triggers for Cohort 2:<br>▪ 30-day sustained decline in eGFR by at least 20% from the baseline value confirmed with repeat laboratory tests and/or<br>▪ increase of greater than or equal to 30% and of at least 30 mg/g in UACR from Baseline, using a standard urine chemistry, sustained for at least 30 days with two repeat central laboratory testing 7 days apart at least 30 days after initial event for confirmation.<br><br>In both cohorts, each subject's annual rate of renal decline, based on adequate historical and clinical data obtained at least 24 months prior to Screening Visit, will serve as a comparator for monitoring the rate of progression of renal insufficiency over time.
**Investigational Plan:**<br>**Screening:** Subjects who satisfy all eligibility criteria and provide written informed consent may be enrolled into the study. Subjects should have adequate historical clinical data to provide a reasonable estimate of the rate of progression of CKD (Inclusion Criterion 8). Screening procedures include a full physical exam (PE), electrocardiogram (ECG), and laboratory assessments (hematology, clinical chemistry, and urinalysis). A renal magnetic resonance imaging (MRI) study will be obtained to determine renal dosing by volume analysis and assess for renal masses, location of adjacent organs, and safe needle pathway for percutaneous injection of REACT.<br><br>**Renal Biopsy:** One to seven days prior to undergoing renal biopsy, enrolled subjects will undergo an interim PE. Laboratory tests, including renal function, hemoglobin, and a urine pregnancy test for females of childbearing potential, will also be performed. Eligible subjects satisfying all eligibility criteria will be admitted to the site to undergo an imaged-guided percutaneous kidney biopsy with an automated biopsy device and with ultrasound or CT guidance. A minimum of two cores using a 16-gauge biopsy needle/punch or four cores using an 18-gauge biopsy needle/punch must be collected to provide sufficient material for the manufacture of REACT. One additional pass can be made if a core specimen for local pathology is required by the institution. Subjects who do not experience complications from the biopsy may be discharged the same day consistent with site's standard practice. Each subject's kidney biopsy tissue will be sent to ProKidney. After the biopsy, a renal ultrasound will be performed on Day 0 with the aim of monitoring for possible subclinical AEs (e.g., bleeding). Subjects will return to clinic on Day 1 follow-up post-biopsy to assess for any AEs. If biopsy-related AEs occur, the subject should not be discharged until the AEs have resolved, stabilized, or returned to baseline. If consistent with the site's standard practice, the subject may be discharged the same day of biopsy after no less than 6 hours of observation and monitoring.<br><br>**Randomization:** Investigators will randomize the subject (1:1) to Cohort 1 (up to 25 subjects) or Cohort 2 (up to 25 subjects) via an interactive web randomization system (IWRS).

Randomization will occur at a 1:1 ratio with equal distribution between the cohorts. Since this is an open-label study, neither the study subject, Investigators, site staff, or Sponsor will be blinded to the treatment assignment.<br><br>**<u>REACT Injection:</u>** Fourteen to twenty-one days prior to the first scheduled injection date, subjects will undergo an interim PE and laboratory tests, including renal function and hemoglobin. On the day of the scheduled REACT injection, subjects will be admitted into the hospital/clinical research center. After warming of REACT, the investigational product will be injected into the kidney cortex region using a percutaneous approach with CT image guidance. This procedure will follow a standardized technique and be performed by interventional proceduralists familiar with other similar renal interventions such as mass/cyst biopsy and small tumor ablations who have undergone prior ProKidney training and credentialing. The second REACT injection for Cohort 1 and Cohort 2 will be performed in the contralateral kidney, i.e., the kidney not previously injected with REACT/biopsied. Therefore, each kidney will receive a single injection when two REACT injections are administered. If product- or procedure-related AEs occur following REACT injection, the subject should not be discharged until the AEs have resolved, stabilized, or returned to baseline. If consistent with the site's standard practice, the subject may be discharged the same day as the REACT injection after no less than 4 hours of observation and monitoring.<br><br>**<u>Follow-up:</u>** Subjects will return for follow-up visits after each REACT injection on Day 1, Day 7, Day 14 (±7 days), Day 28 (±7 days), and at Month 3 (±10 days). Subjects in Cohort 1 will receive a second REACT injection 3 months (+60 days) after their first injection. Long-term follow-up visits will continue after the last REACT injection at 3-month intervals (±10 days) starting at Month 3 and continuing until Month 18 End of Study (EOS) Visit.<br><br>Subjects in Cohort 2 will return for follow-up visits after first REACT injection on Day 1, Day 7, Day 14 (±7 days), Day 28 (±7 days), and at Month 3 (±10 days). Long-term follow-up visits will continue after the last REACT injection at 3-month intervals (±10 days) starting at Month 3 and continuing until Month 18 End of Study (EOS) Visit. They may receive a second REACT injection if a redose trigger is met between months 3-15.<br><br>**<u>Safety Monitoring:</u>** Hemorrhage requiring medical or interventional management following renal biopsy and/or REACT injection is a known and foreseeable risk to subjects participating in this study. Therefore, hemoglobin and hematocrit will be measured by the site's local laboratory before and after the biopsy and before and after each injection, along with a renal ultrasound obtained post-procedure. Subjects will also be observed for complications as per the site's local procedures following renal biopsy. After REACT injection, an ultrasound will be performed on Day 0, Day 1, and Day 7, with the aim of monitoring for possible subclinical AEs (e.g., swelling, fluid accumulation). Following REACT injection(s), all subjects will be observed for no less than 4 hours to monitor for complications.
**Data Monitoring Committee:** An independent Data Monitoring Committee (DMC) will be chartered to assist ProKidney to ensure subject safety, especially as it relates to unexpected investigational product-related events. The DMC will consist of members who have expertise

directly related to protocol-specified activities. It will function independently, and its members will have no other engagement with ProKidney. The DMC will meet by teleconference (or other means) at regular intervals, depending on the speed of subject enrollment and the amount of new data generated. The DMC will advise ProKidney on aspects concerning the safety of subjects participating in the clinical trial. Apart from reviewing study data, the DMC will consider feedback from the Sponsor and Investigators. Recommendations of the DMC will be shared by ProKidney with the study centers, Institutional Review Boards/Ethics Committees, and regulatory authorities, as appropriate. Other specific activities, responsibilities, and composition of the DMC will be described in the DMC charter.
**Statistical Analysis Methods**<br>Efficacy: The estimated glomerular filtration rate will be estimated using a linear mixed effects model. The change in total annualized slope from pre-first REACT injection to post-last REACT injection (over at least 18 months post injection) will be compared between the treatment arms.<br><br>A Kaplan-Meier analysis will be utilized for each time-to-event secondary or exploratory efficacy endpoint, by treatment, summarizing the estimated quartiles (where estimable), numbers of events, subjects at risk by time point, and censoring time points.<br><br>Safety: Summaries of incidence rates of TEAEs judged to be procedure-related and investigational product-related, by TEAE analysis period, SOC and PT will be presented.

Table 1: Time and Events Table – Cohort 1

Clinical Assessment	Screening Visit	Randomization	Renal Biopsy****			Preparation and Shipment of REACT Product	First REACT Injection**			Follow-up First REACT Injection				Interval between REACT Injections = 3 Months (+60) Days***	Second REACT Injection [b]		Follow-Up Long-Term				EOS[c]
	Day −60 to −7 [a]		Day −7 to −1*	Day 0* Biopsy +14 days	Day 1 Post Biopsy		Day -21 to -14*	Day 0* REACT Injection +30 days	Day 1	Day 7 Follow-Up	Day 14 ±7 days	Day 28 ±7 days	Month 3* (pre-2nd Injection) ±10 days		Day 0* REACT Injection +60 days	Day 1 Follow-up	Day 7 Follow-Up	Day 14 ±7 days	Day 28 ±7 days	Months 3,6, 9, 12, 15 ±10 days	Month 18 ±10 days
Obtain Informed Consent [d]	X																				
Verify I/E Criteria	X																				
Obtain Demographic Data	X																				
Obtain Medical History	X																				
Record Concomitant Medications	X		X	X	X		X	X	X	X	X	X	X		X	X	X	X	X	X	X
Perform Comprehensive PE[e]	X																				
Perform Interim PE [e]			X				X				X	X	X					X	X	X	X
Measure Vital Signs [f]	X		X	X [g]	X		X	X [g]	X	X	X	X	X		X [g]	X	X	X	X	X	X
Conduct Laboratory Tests	X		X	X	X		X	X	X	X	X	X	X		X	X	X	X	X	X	X
Perform 12-Lead ECG	X																				X
Perform Ultrasound				X [h]	X [h]			X [h]	X [h]	X [h]	X [h]				X [h]	X [h]	X [h]	X [h]			
Perform MRI Study	X [i]																				X
Admit to Hospital/Discharge				X [j]				X [j]							X [j]						
Perform Kidney Biopsy				X																	
Monitor/Record AEs			X	X	X		X	X	X	X	X	X	X		X	X	X	X	X	X	X
Inject Autologous REACT								X [k]							X [k]						
CT Scan								X							X						

Abbreviations: AE = adverse event; COVID-19 = coronavirus disease 2019; CT = computed tomography; ECG = electrocardiogram; EOS = End of Study; I/E = inclusion/exclusion; IgG = immunoglobulin G; MRI = magnetic resonance imaging; PE = physical examination; REACT = renal autologous cell therapy.

* If a subject is tested for COVID-19 and is found positive, biopsies and injections will need to be postponed for a minimum of 30 days and a maximum of 90 days from onset of symptoms/diagnosis and subjects must be cleared by Investigator and Medical Monitor for proceeding with procedures.

** **Every attempt should be made to ensure that the first REACT injection is administered 12 weeks (+30 days) after biopsy.** In the event that the subject cannot schedule his/her first REACT injection 12 weeks (+30 days) after biopsy or cannot keep his/her scheduled pre-first injection day visit, the Medical Monitor must be notified immediately.

*** **Every attempt should be made to ensure that the second REACT injection is administered 3 months (+60 days) after the first injection**. In the event that the subject cannot schedule his/her second REACT injection 3 months after receiving the first injection or cannot keep his/her scheduled 6-month visit, the Sponsor and the Medical Monitor must be notified immediately.

**** Subjects must not receive a vaccine of any kind within a minimum of 30 and maximum of 90 days before or after biopsy and injection procedures depending on a consult between Investigator and Medical Monitor for evaluation of adverse effects.

a. If the screening assessment falls outside of the 60-day window before renal biopsy, rescreening will be performed as described in Section 6.1.
b. If a subject does not receive a second REACT injection they must complete all visits per protocol except visits day 1, day 7, and day 14 post second REACT injection. On Second REACT Injection Day subjects will still undergo the following assessments: record con meds, vital signs, laboratory tests, and record AE’s. Subjects will not receive ultrasound, CT, or undergo the injection procedure.
c. The EOS Visit will take place at least 18 months after the second REACT injection or when the subject is terminated from the study by the Investigator (Section 8.4), or when the subject voluntarily discontinues from the study (Section 4.8).
d. The informed consent form must be signed and dated prior to conducting any study-specific procedures, including those at the Screening Visit.
e. The comprehensive PE and interim PE are described in Section 7.2.2.
f. Vital signs include heart rate, resting blood pressure, respiration rate, and body temperature (Section 7.2.1).
g. Vital signs, including heart rate, blood pressure, and respiration rate, will be measured throughout the procedure. Temperature is not required to be recorded throughout the procedure but should be documented once.
h. Ultrasound will be performed following the in-subject renal biopsy on Day 0 Biopsy and Day 1 Post-Biopsy with the aim of monitoring possible, subclinical AEs. Subsequent ultrasounds will occur on REACT Injection days post procedure, Day 1 and Day 7post injections to monitor for subclinical AEs. If there is a hematoma greater than 2 cm present on Day 0, Day 1 or Day 7 for REACT injections, then an ultrasound should be conducted at Day 14.
i. An MRI study without contrast will be performed at the Screening Visit to determine kidney size and volume. If a subject cannot undergo MRI then CT can be substituted to obtain kidney size and volume. If subject cannot undergo MRI or CT please consult with Medical Monitor for approval for subject to continue in the study.
j. Subjects may be admitted to hospital per site standard practice. Subjects who do not experience complications may be discharged the same day consistent with site standard practice.
k. The REACT preparation will be handled and injected according to procedures described in the Procedure Training Manual.

Table 2: Laboratory Time and Events Table – Cohort 1

Clinical Assessment	Screening Visit	Renal Biopsy			First REACT Injection			Follow-up First REACT Injection				Second REACT Injection [b]		Follow-Up Long-Term				EOS [c]
	Day –60 to –7 [a]	Day –7 to –1*	Day 0* Biopsy +14 days [g]	Day 1	Day -21 to -14*	Day 0* REACT Injection +30 days [g]	Day 1 Follow-up	Day 7 Follow Up	Day 14 ±7 days	Day 28 ±7 days	Month 3* (pre-2nd injection visit) ±10 days	Day 0* REACT Injection +60days [g]	Day 1 Follow-up	Day 7 Follow Up	Day 14 ±7 days	Day 28 ±7 days	Months 3,6, 9, 12, 15 ±10 days	Month 18 ±10 days
Clinical Chemistry																		
Standard panel	X	X	X	X	X	X	X	X	X	X	X	X	X	X	X	X	X	X
Renal analytes	X	X	X	X	X	X	X	X	X	X	X	X	X	X	X	X	X	X
Electrolyte panel	X	X	X	X	X	X	X	X	X	X	X	X	X	X	X	X	X	X
Lipid panel	X	X	X	X	X	X	X	X	X	X	X	X	X	X	X	X	X	X
Pregnancy test [d]	X	X	X	X	X	X	X	X	X	X	X	X	X	X	X	X	X	X
FSH test [e]	X																	
Serology																		
HIV, HBV, HCV	X																	
Hematology																		
Standard cell counts/indices	X	X	X	X	X	X	X	X	X	X	X	X	X	X	X	X	X	X
Hemoglobin, hematocrit [f]	X	X [f]	X [g]	X	X [f]	X [g]	X	X	X	X	X [f]	X [g]	X	X	X	X	X	X
Coagulation Status																		
Platelet count	X	X			X						X							X
APTT	X	X			X						X							X
PT-INR	X	X			X						X							X
Urine Chemistry																		
Standard (macro panel)	X	X	X	X	X	X	X	X	X	X	X	X	X	X	X	X	X	X
Microalbumin/creatinine ratio	X	X	X	X	X	X	X	X	X	X	X	X	X	X	X	X	X	X
Protein/creatinine ratio	X	X	X	X	X	X	X	X	X	X	X	X	X	X	X	X	X	X
Spot test (micro panel)	X	X [h]	X [h]		X [h]	X [h]	X [h]				X [h]	X [h]	X [h]					
Additional Test																		
HbA_{1c}	X		X		X	X					X	X					X	X
Drugs of abuse	X																	
iPTH	X				X	X					X	X					X	X
NGAL	X	X	X	X	X	X	X	X	X	X	X	X	X	X	X	X	X	X
Research (reserve) samples [i]	X	X	X	X	X	X	X	X	X	X	X	X	X	X	X	X	X	X

Abbreviations: APTT = activated partial thromboplastin time; COVID-19 = coronavirus disease 2019; EOS = End-of-Study; FSH = follicle-stimulating

hormone; HbA_{1c} = glycosylated hemoglobin; HBV = hepatitis B virus; hCG = human chorionic gonadotropin; HCV = hepatitis C virus; HIV = human immunodeficiency virus; IgG = immunoglobulin G; NGAL = neutrophil gelatinase-associated lipocalin; iPTH = intact parathyroid hormone; PT-INR = prothrombin time-international normalized ratio; REACT = Renal Autologous Cell Therapy.

* If a subject is tested for COVID-19 and receives a positive result, biopsies and injections will need to be postponed for a minimum of 30 days and maximum of 90 days from onset of symptoms/diagnosis and the subject must be cleared by Investigator and Medical Monitor for proceeding with procedures.

a. If the screening assessment falls outside of the 60-day window before renal biopsy, rescreening will be performed as described in Section 6.1.
b. If a subject does not receive a second REACT injection they must complete all visits per protocol except visits Day 1, Day 7 and Day 14 post second REACT injection. On Second REACT Injection Day subjects will still undergo the following assessments: record con meds, vital signs, laboratory tests, and record AE's. Subjects will not receive ultrasound, CT, or undergo the injection procedure.
c. The EOS Visit will take place at least 18 months after the second REACT injection or when the subject is terminated from the study by the Investigator (Section 8.4) or when the subject voluntarily discontinues from the study (Section 4.8).
d. The clinic will perform a urine dip-strip pregnancy test. If positive, then a confirmatory serum hCG test will be performed by the central laboratory. A serum sample may be collected for a pregnancy test where a urine sample is not available.
e. Post-menopausal women with a confirmatory FSH test do not have to undergo pregnancy testing throughout the study.
f. At the Day -7 to Day -1 visit before Day 0 for renal biopsy and Day -21 to Day -14 prior to first REACT injection Day 0, and at Month 3 visit prior to second REACT injection Day 0, hemoglobin levels will be verified as >9 g/dL per site standard practices.
g. On Days 0 for renal biopsy and REACT injection(s), hemoglobin and hematocrit will be measured locally before and after the procedure. These samples will be processed by the site's local laboratory to accelerate notification of results and subsequent decisions affecting clinical care. In addition, blood samples for hemoglobin and hematocrit will be sent to the central laboratory where results can be entered into the study database. The Day 0 PRE central laboratory kit is to be used before procedures and the Day 0 Post central laboratory kit should be used after procedures.
h. At Screening, Biopsy Day -7 to -1, day of renal biopsy; Day -21 to -14 visit before the first injection, on the day of, and day after REACT injection(s), microscopic urinalysis will be performed by Labcorp to confirm the absence of infection in addition to urine test stick at the clinical site. On procedure days the urine test stick should be completed prior to procedure only.
i. Research samples (serum/plasma and urine) will be collected, frozen, and stored for the evaluation of novel biomarkers.

Table 3: Time and Events Table – Cohort 2 for Subjects Not Meeting Renal Function Redose Trigger for a Second REACT Injection

Clinical Assessment	Screening Visit	Randomization	Renal Biopsy**			Preparation and Shipment of REACT Product	First REACT Injection***			Follow-Up Long-Term [b]				EOS [b]
	Day –60 to –7 [a]		Day –7 to –1*	Day 0* Biopsy +14 days	Day 1 Post Biopsy		Day -21 to -14*	Day 0* REACT Injection +30 days	Day 1 Follow-up	Day 7 Follow-Up	Day 14 ±7 days	Day 28 ±7 days	Months 3,6, 9, 12, 15 ±10 days	Month 18 ±10 days
Obtain Informed Consent [c]	X													
Verify I/E Criteria	X													
Obtain Demographic Data	X													
Obtain Medical History	X													
Record Concomitant Medications	X		X	X	X		X	X	X	X	X	X	X	X
Perform Comprehensive PE [d]	X													
Perform Interim PE [d]			X				X				X	X	X	X
Measure Vital Signs [e]	X		X	X [f]	X [f]		X	X [f]	X	X	X	X	X	X
Conduct Laboratory Tests	X		X	X	X		X	X	X	X	X	X	X	X
Perform 12-Lead ECG	X													X
Perform Ultrasound				X [g]	X [g]			X [g]	X [g]	X [g]	X [g]			
Perform MRI Study	X [h]													X
Admit to Hospital/Discharge				X [i]				X [i]						
Perform Kidney Biopsy				X										
Monitor/Record AEs			X	X	X		X	X	X	X	X	X	X	X
Inject Autologous REACT								X [j]						
CT Scan								X						
Evaluation for Criteria to Administer a Second REACT Injection [b]													**Reevaluate up to Month 15** [b]	

Abbreviations: AE = adverse event; COVID-19 = coronavirus disease 2019; CT = computed tomography; ECG = electrocardiogram; EOS = End-of-Study; I/E = inclusion/exclusion; IgG = immunoglobulin G; MRI = magnetic resonance imaging; PE = physical examination; REACT = renal autologous cell therapy.

* If a subject is tested for COVID-19 and is found positive, biopsies and injections will need to be postponed for a minimum of 30 days and maximum of 90 days from onset of symptoms/diagnosis and subjects must be cleared by Investigator and Medical Monitor for proceeding with procedures.

** Subjects must not receive a vaccine of any kind within a minimum of 30 and a maximum of 90 days before or after biopsy and injection procedures depending on a consult between Investigator and Medical Monitor for evaluation of adverse effects.

*** **Every attempt should be made to ensure that the first REACT injection is administered 12 weeks (+30 days) after biopsy.** In the event that the subject cannot schedule his/her first REACT injection 12 weeks (+30 days) after biopsy or cannot keep his/her scheduled pre-first injection day visit, the Medical Monitor must be notified immediately.

a. If the screening assessment falls outside of the 60-day window before renal biopsy, rescreening will be performed as described in Section 6.1.
b. In the event that the subject meets criteria for a second REACT injection at least 3 months after first REACT injection and up to/including Month 15 (Section 4.6.2.1), the study assessments should be modified and follow the "Time and Events Table – Cohort 2 for Subjects Meeting Renal Function Redose Trigger for a Second REACT Injection" (Table 5). An unscheduled visit should be performed to confirm sustainment of trigger. The EOS Visit will take place at least 18 months after the last REACT injection or when the subject is terminated from the study by the Investigator (Section 8.4) or when the subject voluntarily discontinues from the study (Section 4.8).
c. The informed consent form must be signed and dated prior to conducting any study-specific procedures, including those at the Screening Visit.
d. The comprehensive PE and interim PE are described in Section 7.2.2.
e. Vital signs include heart rate, resting blood pressure, respiration rate, and body temperature (Section 7.2.1).
f. Vital signs, including heart rate, blood pressure, and respiration rate, will be measured at regular intervals throughout the procedure. Temperature is not required to be recorded throughout the procedure but should be documented once.
g. Ultrasound will be performed following the in-subject renal biopsy on Day 0 Biopsy and Day 1 Post Biopsy with the aim of monitoring possible, subclinical AEs. Subsequent ultrasounds will occur on REACT Injection days post procedure, Day 1 and Day 7 post injections to monitor for subclinical AEs. If there is a hematoma greater than 2 cm present on Day 0, Day 1, or Day 7 for REACT injections, then an ultrasound should be conducted at Day 14.
h. An MRI study without contrast will be performed at the Screening Visit to determine kidney size and volume. If a subject cannot undergo MRI then CT can be substituted to obtain kidney size and volume. If subject cannot undergo MRI or CT please consult with Medical Monitor for approval for subject to continue in the study.
i. Subjects may be admitted to hospital per site standard practice. Subjects who do not experience complications may be discharged the same day consistent with site standard practice.
j. The REACT preparation will be handled and injected according to procedures described in the Procedure Training Manual.

Table 4: Laboratory Time and Events Table – Cohort 2 for Subjects Not Meeting Renal Function Redose Trigger for Second REACT Injection

Clinical Assessment	Screening Visit	Renal Biopsy			First REACT Injection			Follow-Up Long-Term [b]				EOS [c]
	Day –60 to –7 [a]	Day –7 to –1*	Day 0* Biopsy +14 days [g]	Day 1 Post Biopsy	Day -21 to -14*	Day 0* REACT Injection +30 days [g]	Day 1 Follow-up	Day 7 Follow-Up	Day 14 ±7 days	Day 28 ±7 days	Months 3,6, 9, 12, 15 ±10 days	Month 18 ±10 days
Clinical Chemistry												
Standard panel	X	X	X	X	X	X	X	X	X	X	X	X
Renal analytes	X	X	X	X	X	X	X	X	X	X	X	X
Electrolyte panel	X	X	X	X	X	X	X	X	X	X	X	X
Lipid panel	X	X	X	X	X	X	X	X	X	X	X	X
Pregnancy test [d]	X	X	X	X	X	X	X	X	X	X	X	X
FSH test [e]	X											
Serology												
HIV, HBV, HCV	X											
Hematology												
Standard cell counts/indices	X	X	X	X	X	X	X	X	X	X	X	X
Hemoglobin, hematocrit [f]	X	X [f]	X [g]	X	X [f]	X [g]	X	X	X	X	X	X
Coagulation Status												
Platelet count	X	X			X							X
APTT	X	X			X							X
PT-INR	X	X			X							X
Urine Chemistry												
Standard (macro panel)	X	X	X	X	X	X	X	X	X	X	X	X
Microalbumin/creatinine ratio	X	X	X	X	X	X	X	X	X	X	X	X
Protein/creatinine ratio	X	X	X	X	X	X	X	X	X	X	X	X
Spot test (micro panel)	X	X [h]	X [h]		X [h]	X [h]	X [h]					
Additional Tests												
HbA_{1c}	X		X		X	X					X	X
Drugs of abuse	X											
iPTH	X				X	X					X	X
NGAL	X	X	X	X	X	X	X	X	X	X	X	X
Research (reserve) samples [i]	X	X	X	X	X	X	X	X	X	X	X	X
Evaluation for Criteria to Administer a Second REACT Injection [b]											**Reevaluate up to Month 15** [b]	

Abbreviations: APTT = activated partial thromboplastin time; COVID-19 = coronavirus disease 2019; EOS = End-of-Study; FSH = follicle-stimulating hormone; HbA_{1c} = glycosylated hemoglobin; HBV = hepatitis B virus; hCG = human chorionic gonadotropin; HCV = hepatitis C virus; HIV = human immunodeficiency virus; IgG = immunoglobulin G; NGAL = neutrophil gelatinase-associated lipocalin; iPTH = intact parathyroid hormone; PT-INR = prothrombin time-international normalized ratio; REACT = Renal Autologous Cell Therapy.

* If a subject is tested for COVID-19 and receives a positive result, biopsies and injections will need to be postponed for a minimum of 30 days and a maximum

of 90 days from onset of symptoms/diagnosis and the subject must be cleared by Investigator and Medical Monitor for proceeding with procedures.

a. If the screening assessment falls outside of the 60-day window before renal biopsy, rescreening will be performed as described in Section 6.1.
b. In the event that the subject meets criteria for a second REACT injection at least 3 months after first REACT injection and up to/including Month 15 (Section 4.6.2.1), the study assessments should be modified and follow the "Time and Events Table – Cohort 2 for Subjects Meeting Renal Function Redose Trigger for a Second REACT Injection" (Table 5). An unscheduled visit/s should be performed to confirm sustainment of triggers.
c. The EOS Visit will take place at least 18 months after the last REACT injection or when the subject is terminated from the study by the Investigator (Section 8.4) or when the subject voluntarily discontinues from the study (Section 4.8).
d. The clinic will perform a urine dip-strip pregnancy test. If positive, then a confirmatory serum hCG test will be performed by the central laboratory. A serum sample may be collected for a pregnancy test where a urine sample is not available.
e. Post-menopausal women with a confirmatory FSH test do not have to undergo pregnancy testing throughout the study.
f. At the Day -7 to Day -1 visit before Day 0 for renal biopsy and Day -21 to Day -14 prior to first REACT injection Day 0, hemoglobin levels will be verified as >9 g/dL per site standard practices.
g. On Days 0 for renal biopsy and REACT injection(s), hemoglobin and hematocrit will be measured locally before and after the procedure. These samples will be processed by the site's local laboratory to accelerate notification of results and subsequent decisions affecting clinical care. In addition, blood samples for hemoglobin and hematocrit will be sent to the central laboratory where results can be entered into the study database. The Day 0 PRE central laboratory kit is to be used before procedures and the Day 0 Post central laboratory kit should be used after procedures.
h. At Screening, Biopsy Day -7 to -1, day of renal biopsy; at the Day -21 to -14 visit before the first injection, on the day of, and day after REACT injection, microscopic urinalysis will be performed by Labcorp to confirm the absence of infection in addition to urine test stick at the clinical site. On procedure days the urine test stick should be completed prior to procedure only.
i. Research samples (serum/plasma and urine) will be collected, frozen, and stored for the evaluation of novel biomarkers.

Table 5: Time and Events Table – Cohort 2 for Subjects Meeting Renal Function Redose Trigger for a Second REACT Injection

Clinical Assessment	**Second REACT Injection [a]		Follow-Up Long-Term				EOS [b]
	Day 0*	Day 1	Day 7	Day 14	Day 28	Months 3,6, 9, 12, 15	Month 18
	REACT Injection	Follow-up	Follow-up	±7 days	±7 days	±10 days	±10 days
Record Concomitant Medications	X	X	X	X	X	X	X
Perform Interim PE [c]				X	X	X	X
Measure Vital Signs [d]	X [e]	X	X	X	X	X	X
Conduct Laboratory Tests	X	X	X	X	X	X	X
Perform 12-Lead ECG							X
Perform Ultrasound	X [f]	X [f]	X [f]	X [f]			
Perform MRI Study							X
Admit to Hospital/Discharge	X [g]						
Monitor/Record AEs	X	X	X	X	X	X	X
Inject Autologous REACT	X [h]						
CT Scan	X						

Abbreviations: Abbreviations: AE = adverse event; COVID-19 = coronavirus disease 2019; CT = computed tomography; ECG = electrocardiogram; EOS = End-of-Study; IgG = immunoglobulin G; MRI = magnetic resonance imaging; PE = physical examination; REACT = renal autologous cell therapy.

* If a subject is tested for COVID-19 and is found positive, biopsies and injections will need to be postponed for a minimum of 30 days and a maximum of 90 days from onset of symptoms/diagnosis and subject must be cleared by Investigator and Medical Monitor for proceeding with procedures.

** Subjects must not receive a vaccine of any kind within a minimum of 30 and a maximum of 90 days before or after biopsy and injection procedures depending on a consult between Investigator and Medical Monitor for evaluation of adverse effects.

a. In the event that the subject meets criteria for a second REACT injection prior to Month 15 (Section 4.6.2.1), the study assessments should be modified and follow the "Time and Events Table – Cohort 2 for Subjects Meeting Renal Function Redose Trigger for a Second REACT Injection" (Table 5). If a subject does not receive the second REACT injection by 60 days of meeting renal function trigger for the second injection, the subject will not undergo the second injection and remain on his/her original follow-up schedule of assessments.

b. The EOS Visit will take place at least 18 months after the second REACT injection or when the subject is terminated from the study by the Investigator (Section 8.4) or when the subject voluntarily discontinues from the study (Section 4.8).

c. The comprehensive PE and interim PE are described in Section 7.2.2.

d. Vital signs include heart rate, resting blood pressure, respiration rate, and body temperature (Section 7.2.1).

e. Vital signs, including heart rate, blood pressure, and respiration rate, will be measured at regular intervals throughout the procedure. Temperature is not required to be recorded throughout the procedure but should be documented once.

f. Ultrasound will be performed following the in-subject renal biopsy on Day 0 Biopsy and Day 1 Post-Biopsy with the aim of monitoring possible, subclinical AEs. Subsequent ultrasounds will occur on REACT Injection days post procedure, Day 1 and Day 7 post injections to monitor for subclinical AEs. If there is a hematoma greater than 2 cm present on Day 0, Day 1 or Day 7 for REACT injections, then an ultrasound should be conducted at Day 14.

g. Subjects may be admitted to hospital per site standard practice. Subjects who do not experience complications may be discharged the same day consistent with site standard practice.

h. The REACT preparation will be handled and injected according to procedures described in the REACT Training Manual.

Table 6: Laboratory Time and Events Table – Cohort 2 for Subjects Meeting Renal Function Redose Trigger for Second REACT Injection

Clinical Assessment	Second REACT Injection		Follow-Up Long-Term				EOS [a]
	Day 0* REACT Injection [c]	Day 1 Follow-up	Day 7 Follow-Up	Day 14 ±7 days	Day 28 ±7 days	Months 3,6, 9, 12, 15 ±10 days	Month 18 ±10 days
Clinical Chemistry							
Standard panel	X	X	X	X	X	X	X
Renal analytes	X	X	X	X	X	X	X
Electrolyte panel	X	X	X	X	X	X	X
Lipid panel	X	X	X	X	X	X	X
Pregnancy test [b]	X	X	X	X	X	X	X
FSH test							
Serology							
HIV, HBV, HCV							
Hematology							
Standard cell counts/indices	X	X	X	X	X	X	X
Hemoglobin, hematocrit [c]	X [c]	X	X	X	X	X	X
Coagulation Status							
Platelet count							X
APTT							X
PT-INR							X
Urine Chemistry							
Standard (macro panel)	X	X	X	X	X	X	X
Microalbumin/creatinine ratio	X	X	X	X	X	X	X
Protein/creatinine ratio	X	X	X	X	X	X	X
Spot test (micro panel)	X [d]	X [d]					
Additional Tests							
HbA_{1c}	X					X	X
Drugs of abuse							
iPTH	X					X	X
NGAL	X	X	X	X	X	X	X
Research (reserve) samples [e]	X	X	X	X	X	X	X

Abbreviations: Abbreviations: APTT = activated partial thromboplastin time; COVID-19 = coronavirus disease 2019; EOS = End-of-Study; FSH = follicle-stimulating hormone; HbA_{1c} = glycosylated hemoglobin; HBV = hepatitis B virus; hCG = human chorionic gonadotropin; HCV = hepatitis C virus; HIV = human immunodeficiency virus; IgG = immunoglobulin G; NGAL = neutrophil gelatinase-associated lipocalin; iPTH = intact parathyroid hormone; PT-INR = prothrombin time-international normalized ratio; REACT = Renal Autologous Cell Therapy.

* If a subject is tested for COVID-19 and receives a positive result, biopsies and injections will need to be postponed for a minimum of 30 days and maximum

of 90 days from onset of symptoms/diagnosis and the subject must be cleared by Investigator and Medical Monitor for proceeding with procedures.

a. The EOS Visit will take place at least 18 months after the second REACT injection or when the subject is terminated from the study by the Investigator (Section 8.4) or when the subject voluntarily discontinues from the study (Section 4.8).
b. The clinic will perform a urine dip-strip pregnancy test. If positive, then a confirmatory serum hCG test will be performed by the central laboratory. A serum sample may be collected for a pregnancy test where a urine sample is not available.
c. On Days 0 for REACT injection(s), hemoglobin and hematocrit will be measured locally before and after the procedure. These samples will be processed by the site's local laboratory to accelerate notification of results and subsequent decisions affecting clinical care. In addition, blood samples for hemoglobin and hematocrit will be sent to the central laboratory where results can be entered into the study database. The Day 0 PRE central laboratory kit is to be used before procedures and the Day 0 Post central laboratory kit should be used after procedures.
d. At the Day -21 to -14 visit before the first injection and on the day of, and day after REACT injection(s), microscopic urinalysis will be performed by Labcorp to confirm the absence of infection in addition to urine test stick at the clinical site. On procedure days the urine test stick should be completed prior to procedure only.
e. Research samples (serum/plasma and urine) will be collected, frozen, and stored for the evaluation of novel biomarkers.

TABLE OF CONTENTS

SIGNATURE PAGE 2

EMERGENCY CONTACT INFORMATION 3

PROTOCOL SYNOPSIS 4

LIST OF TABLES 29

LIST OF FIGURES 29

LIST OF ABBREVIATIONS AND DEFINITIONS OF TERMS 30

1 INTRODUCTION AND BACKGROUND 33

1.1 Chronic Kidney Disease 33

1.2 Current Therapies and Unmet Medical Need 34

1.3 Renal Autologous Cell Therapy (REACT) 35

1.4 Development Rationale 35

1.4.1 Type 1 Diabetes 37

1.5 Summary of Potential Risks and Benefits 37

1.5.1 Potential Risks 37

1.5.2 Identified Risks 48

1.5.3 Potential Benefits 49

2 STUDY OBJECTIVE AND ENDPOINTS 49

2.1 Objective 50

2.1.1 Primary Efficacy Endpoint 50

2.1.2 Primary Safety Endpoint 50

2.2 Secondary Efficacy and Exploratory Endpoints 50

2.2.1 Secondary Efficacy Endpoints 50

2.2.2 Exploratory Endpoints 51

2.2.3 Secondary Safety Endpoints 52

3 INVESTIGATIONAL PRODUCT 52

3.1 Description of Investigational Product 52

3.2 Procurement and Manufacture of REACT 53
3.2.1 Biopsy 53
3.2.2 Culture Expansion and Selection of SRC 53
3.2.3 Formulation....54
3.2.4 REACT Product for Injection 54
3.2.5 REACT Dose 54
3.3 Preliminary Clinical Evidence Supporting 2 REACT Injections 56
3.4 Safety of 2 REACT Injections....57
3.5 Investigational Product Packaging....58
3.6 Investigational Product Label 59
3.7 Investigational Product Transportation....59
3.8 Investigational Product Accountability 59
3.8.1 Investigational Product Handling and Disposal....60
3.8.2 Disposition of Stored Specimens 60
3.8.3 Unused Biopsy Material 60
4 INVESTIGATIONAL PLAN 61
4.1 Study Administrative Structure 61
4.2 Overall Study Design....61
4.3 Number of Subjects 64
4.4 Treatment Assignment....64
4.5 Treatment Compliance....64
4.6 Study Duration....65
4.6.1 Cohort 1 65
4.6.2 Cohort 2 65
4.7 Prohibited and Concomitant Medications 67
4.8 Subject Withdrawal Criteria 67
5 STUDY POPULATION 68
5.1 Inclusion Criteria 68

5.2 Exclusion Criteria 69

6 STUDY VISITS 72

6.1 Screening 72

6.2 Randomization 73

6.3 Biopsy 74

6.4 REACT Injection 75

6.5 Discharge After REACT Injection 76

6.6 Follow-up Visits 77

6.6.1 Cohort 1 77

6.6.2 Cohort 2 Subjects Not Meeting Trigger for Redosing by Month 15 77

6.6.3 Cohort 2 Subjects Meeting Trigger for Redosing Prior to Month 15 78

6.6.4 End-of-Study Visit 78

6.6.5 Study Completion 79

7 STUDY ASSESSMENTS AND PROCEDURES 79

7.1 Demography and Medical History 79

7.2 Clinical Evaluations 80

7.2.1 Vital Signs 80

7.2.2 Physical Examination 80

7.2.3 ECG 80

7.2.4 Concomitant Medications 81

7.2.5 Laboratory Assessments 81

7.2.6 Urine Chemistry 82

7.2.7 Hematology 83

7.2.8 Viral Serology 83

7.2.9 Drugs of Abuse Screen 83

7.2.10 Research Samples 84

7.2.11 Pregnancy Screen 84
7.2.12 Renal Imaging 84
8 SAFETY ASSESSMENTS AND MANAGEMENT 85
8.1 Adverse and Serious Adverse Events 85
8.1.1 Definition of Adverse Events 85
8.2 Adverse Event Intensity and Relationship Assessment 88
8.2.1 Intensity Scale 88
8.2.2 Relationship Assessment 89
8.3 Recording and Reporting Adverse Events 90
8.3.1 Suspected Unexpected Serious Adverse Reactions 91
8.3.2 Pregnancy 91
8.3.3 Data Monitoring Committee 92
8.3.4 Adverse Event Reporting Procedures in Relation to COVID-19 Infection 92
8.4 Stopping Rules for an Individual Subject 92
8.5 Study Suspension or Study Termination 93
8.6 Additional Sponsor Specific Reporting Conventions 94
9 STATISTICAL METHODS AND PLANNED ANALYSES 94
9.1 Sample Size 94
9.2 Criteria for Evaluation 95
9.2.1 Objective and Endpoints 95
9.2.2 Analysis Conventions 95
9.2.3 Efficacy Analysis 96
9.2.4 Safety Analysis 97
10 ETHICAL AND REGULATORY CONSIDERATIONS 99
10.1 Good Clinical Practice 99
10.1.1 Delegation of Principal Investigator Responsibilities 99
10.1.2 Institutional Review Board/Ethics Committee 99

11 DATA HANDLING AND RECORDKEEPING 102

11.1 Data Collection and Review 102

11.1.1 Data Collection 102

11.1.2 Study Monitoring 103

12 QUALITY CONTROL AND QUALITY ASSURANCE 105

13 PUBLICATION POLICY 105

14 LIST OF REFERENCES 106

LIST OF TABLES

Table 1: Time and Events Table – Cohort 1 14
Table 2: Laboratory Time and Events Table – Cohort 1 16
Table 3: Time and Events Table – Cohort 2 for Subjects Not Meeting Renal Function Redose Trigger for a Second REACT Injection 18
Table 4: Laboratory Time and Events Table – Cohort 2 for Subjects Not Meeting Renal Function Redose Trigger for Second REACT Injection...................................20
Table 5: Time and Events Table – Cohort 2 for Subjects Meeting Renal Function Redose Trigger for a Second REACT Injection 22
Table 6: Laboratory Time and Events Table – Cohort 2 for Subjects Meeting Renal Function Redose Trigger for Second REACT Injection....................................23
Table 7: Literature-Published Percutaneous Kidney Biopsy-Related Complications 38
Table 8: Expected Incidence of Adverse Events and Complications of Injection Procedures Similar to the REACT Percutaneous Investigational Product (REACT) Injection..40
Table 9: Anticipated Investigational Product (REACT) Cell Complications...................43
Table 10: RMCL-002 Safety Data – Kidney Biopsy (N=83), REACT Injection (N=126) and REACT Cell Adverse Events...48
Table 11: Investigational Product 53
Table 12: REACT Dosing Relative to Estimated Kidney Weight...................................55
Table 13: Clinical Laboratory Evaluations 81

LIST OF FIGURES

Figure 1: Schematic of Study Design 63

LIST OF ABBREVIATIONS AND DEFINITIONS OF TERMS

The following abbreviations and specialist terms are used in this study protocol.

Abbreviation	Definition
3D	3-dimensional
ACEi	angiotensin-converting enzyme inhibitors
AE	adverse event
ALT	alanine aminotransferase
APKD	autosomal dominant polycystic kidney disease
APTT	activated partial thromboplastin time
ARB	angiotensin receptor blocker
AST	aspartate aminotransferase
BS	Biopsied Set
BUN	blood urea nitrogen
CFR	Code of Federal Regulations
CKD	chronic kidney disease
CKD-EPI 2009	Chronic Kidney Disease Epidemiology Collaboration serum creatinine equation
CO_2	carbon dioxide
COVID-19	coronavirus disease 2019
CRF	Case Report Form
CRP	C-reactive protein
CT	computed tomography
CTCAE	Common Terminology Criteria for Adverse Events
DHHS	Department of Health and Human Services
DKA	diabetic ketoacidosis
DKD	diabetic kidney disease
DMSO	dimethyl sulfoxide
DMC	Data Monitoring Committee
EC	Ethics Committee
ECG	Electrocardiogram
eGFR	estimated glomerular filtration rate
EOS	End-of-Study
EPO	Erythropoietin
ERA-EDTA	European Renal Association-European Dialysis and Transplant Association
ESRD	end-stage renal disease
FDA	Food and Drug Administration
FSH	follicle-stimulating hormone
g KW^{est}	estimated kidney weight
GCP	Good Clinical Practices
GFR	glomerular filtration rate
GGT	gamma-glutamyl transferase
GMP	Good Manufacturing Practices

Abbreviation	Definition
Hb	Hemoglobin
HbA_{1c}	glycosylated hemoglobin
HBV	hepatitis B virus
hCG	human chorionic gonadotrophin
Hct	Hematocrit
HCV	hepatitis C virus
HDL	high-density lipoprotein
HIV	human immunodeficiency virus
I/E	inclusion/Exclusion
ICF	informed consent form
ICH	International Conference on Harmonization
IgG	immunoglobulin G
INR	international normalized ratio
iPTH	intact parathyroid hormone
IRB	Institutional Review Board
ITT	Intent-to-treat
IV	Intravenous
IWRS	interactive web randomization system
LDH	lactate dehydrogenase
LDL	low-density lipoprotein
LMWH	low-molecular-weight heparin
mITT	Modified intent-to-treat
MRI	magnetic resonance imaging
NCI	National Cancer Institute
NGAL	neutrophil gelatinase-associated lipocalin
NHANES	National Health and Nutrition Examination Survey
NKA	Neo-Kidney Augment
NOAC	novel oral anticoagulants
NSAID	nonsteroidal anti-inflammatory
PBS	phosphate buffered saline
PCR	polymerase chain reaction
PE	physical examination
PO_4	Phosphorus
PT	prothrombin time
PT-INR	prothrombin time - international normalized ratio
QA	Quality Assurance
QC	Quality Control
RBC	red blood cell
REACT	Renal Autologous Cell Therapy
RNA	ribonucleic acid

Abbreviation	Definition
RRT	renal replacement therapy
SAE	serious adverse event
SAF	safety analysis set
sCr	serum creatinine
SLGT2i	sodium glucose cotransporter 2 inhibitor
SOP	standard operating procedure
SRC	selected renal cells
SUSAR	serious and unexpected suspected adverse reaction
T1DM	type 1 diabetes mellitus
T2DM	type 2 diabetes mellitus
TB	Tuberculosis
TEAE	treatment-emergent adverse event
UACR	urine albumin/creatinine ratio
US	United States
WBC	white blood cell

1 INTRODUCTION AND BACKGROUND

1.1 Chronic Kidney Disease

Chronic kidney disease is characterized by progressive nephropathy that without therapeutic intervention will worsen until the patient reaches end-stage renal disease (ESRD). [1] The major causes of CKD are diabetes and hypertension. Approximately 23% of diabetics have CKD and for nearly half of all CKD patients receiving dialysis, diabetes is the direct cause of their CKD.[2-6] Other causes are glomerulonephritis caused by post-infectious conditions and lupus; polycystic kidney disease; long-term use of analgesics; atherosclerosis leading to ischemic nephropathy; ureteral obstruction or stricture leading to renal damage; human immunodeficiency virus (HIV) infection; sickle cell disease; heroin abuse; amyloidosis; and chronic kidney infections.

Mortality in CKD patients increases as glomerular filtration rate (GFR) declines, with increased mortality rates observed with progressing stages of CKD. [7,8] In 2016, Medicare patients with CKD experienced a mortality rate of 122.6 per 1,000 patient-years. When adjusted for sex, age, and race, the rate remained more than double the 43.1 per 1,000 patient-years of those without CKD. [9] Death appears to result from cardiovascular events and in several studies of patients with advanced CKD (Stages 4-5) was more common than progression to dialysis. [10,11]

Prevalence of CKD (stages 1-5) in the general United States (US) adult population was 14.8% in 2013-2016, based on National Health and Nutrition Examination Survey (NHANES) data. Prevalence of CKD Stages 3-5 was at 6.9%.[9] Global prevalence analyzed through a systematic review of CKD studies has been estimated at 13.4% for Stages 1-5 and 10.6% for Stages 3-5. [12] In 2016, the European Renal Association-European Dialysis and Transplant Association (ERA-EDTA) registry reported an incidence of renal replacement therapy for ESRD at 121 per million population within 36 countries, with 84% of those patients reported as receiving hemodialysis. [3] Prevalence worldwide is estimated at 8 to 16%. [13]

The greatest cause of ESRD in the US and Europe is diabetes mellitus, and the incidence of CKD continues to increase, primarily due to the increases in the incidence of type 2 diabetes (T2DM).[5] CKD is often accompanied by adverse outcomes owing to underlying comorbidities and/or risk factors including, cardiovascular disease, hypertension and renovascular disease. [14] The prevalence of cardiovascular disease for the Medicare 5% Sample in 2016 was 64.5% among patients aged 66 and older who had CKD, compared to 32.4% among those who did not have CKD. [9] Patients over 65 years of age are 13 times more likely to die from any cause than progress to ESRD and are 6-fold more likely to die from cardiovascular causes than develop

ESRD. [15] Cardiovascular disease accounts for about 50% of premature deaths in the dialysis population. [16] In order to survive, ESRD patients require renal replacement therapy (dialysis or transplantation). Preventing or delaying adverse outcomes of CKD by intervening early in the disease is the primary strategy in CKD management. Nevertheless, early therapeutic approaches have been less than optimal.

1.2 Current Therapies and Unmet Medical Need

At present, there is no cure for CKD. Treatment of patients with CKD is focused on slowing progression, while managing comorbid conditions including cardiovascular disease and preparing for kidney failure and kidney replacement.

Comorbidities: Anemia, mineral and bone disorders, and cardiovascular events are the major comorbidities requiring treatment in CKD patients. Anemia is often treated via recombinant human erythropoietin. Treatment for mineral and bone disorders focus on maintaining phosphorous and calcium homeostasis. [17] Initial treatment restricts dietary phosphorus intake or uses specific phosphate binders when phosphate or parathyroid hormone levels begin to rise. For chronic therapy, calcium-based phosphate binders are the most widely prescribed treatments for management of CKD-associated hyperphosphatemia. Additionally, vitamin D and its related compounds may be administered in cases of documented deficiency to raise serum calcium concentration sufficiently to suppress parathyroid hormone secretion. Increased risk of cardiovascular disease can be a complication of CKD or an independent comorbidity, with the former being probable in advanced CKD cases. [12,18,19] The use of 1) angiotensin-converting enzyme inhibitors (ACEi) and/or angiotensin receptor blockers (ARB) to reduce proteinuria and control hypertension, 2) insulin titration to achieve appropriate glycosylated hemoglobin (HbA_{1c}), and 3) statin therapy to counter dyslipidemia, collectively aim to reduce cardiovascular risk and prevent or slow the progression of kidney failure.

Renal Replacement Therapy: When a patient reaches stage 5 CKD, renal replacement therapy (RRT) (i.e., dialysis or kidney transplant) is indicated. This is often, but not invariably, accompanied with symptoms or signs attributable to kidney failure (serositis, acid-base or electrolyte abnormalities, pruritus); inability to control volume status or blood pressure; a progressive deterioration in nutritional status refractory to dietary intervention; or cognitive impairment. [20] The vast majority of stage 5 individuals receive hemodialysis. [21] Dialysis replaces about 5-15% of kidney function depending on the intensity and frequency of use and helps restore fluid and electrolyte balance when kidneys fail. However, the life-expectancy of an

ESRD patient initiating hemodialysis is only 4-5 years. [9] Additionally, hemodialysis is associated with multiple and serious complications and quality of life reductions such as arteriovenous graft infections, graft revision, and the need to undergo dialysis up to 3 times per week.

Living donor preemptive renal transplantation in adults should be considered when the eGFR is <20 ml/min/1.73 m^2, and there is evidence of progressive and irreversible CKD over the preceding 6–12 months. [22] Kidney transplantation currently remains the most effective form of therapy; however, there is a chronic shortage of organs. If a patient can secure a kidney for transplantation, long-term therapy with immunosuppressive agents is required to prevent rejection. While newer immunosuppressive regimens are more potent leading to a reduction in immune-mediated graft loss, the use of these regimens has resulted in a higher incidence of medication-related problems including increased rates of infection and cancer.

Therefore, there is a critical unmet medical need for new therapies for CKD which could dramatically slow the progression of disease, and significantly delay the need for renal replacement therapy.

1.3 Renal Autologous Cell Therapy (REACT)

ProKidney' s Renal Autologous Cell Therapy (REACT), formerly known as Neo-Kidney Augment (NKA), is an injectable product composed of an autologous population of selected renal cells (SRC) formulated in a gelatin-based hydrogel or cryopreservation solution. In this trial, REACT will be formulated with cryopreservation solution.

Renal cells are obtained from a patient via a standard percutaneous kidney biopsy procedure following standard medical biopsy practice and guidelines, and the SRC population are selected through a defined manufacturing process. Selected renal cells, a renal cell population naturally involved in renal repair and regeneration, are formulated with cryoprotectant. REACT will be injected into the kidney cortex either in the biopsied or contralateral kidney. Access to the kidney is obtained using a standard percutaneous approach.

1.4 Development Rationale

Human renal progenitor cells have been shown to have therapeutic effect in various animal models of CKD. [23-25] Following acute kidney injury, studies have shown that tubular epithelial cells are essential to restoration of renal function. [26] In the absence of progenitor cells, the kidney

tubular epithelium is repaired by remaining resident surviving epithelial cells, leading to the hypothesis that adult renal-derived cells may have the potential to attenuate disease progression. [27,28] Subsequent studies have identified a central role for native tubular epithelia in renal regeneration. [29-32] Implantation of intraparenchymal differentiated adult renal cells has demonstrated regenerative capacity in experimental models of CKD, while also reducing fibrosis and disease progression. Administration of erythropoietin (EPO)-positive enriched primary kidney cells in a rat CKD model resulted in augmented renal function and reduced renal fibrosis, inflammation and oxidative stress. [33] The direct injection of SRC into the kidney cortex in multiple animal models of CKD has been shown to elicit a regenerative response in multiple locations of the nephron through direct engraftment or tissue replacement, and through a putative paracrine mechanism involving the effect of secreted factors and activation of endogenous renal repair mechanisms that are still active in the chronically diseased kidney that has not yet reached ESRD. [29,30,32] In chronic models, treatment with SRC was demonstrated to stabilize and reverse renal function decline and provide significant survival benefit.

Thus, REACT aims to augment renal function by re-introducing the reparative renal cells (i.e., SRC) into the diseased kidney, where the cells migrate to damaged tubules and glomeruli, replacing effete cells of the CKD glomerular-tubular complex. This leads to activation of endogenous renal repair and regeneration mechanisms still active in the chronically diseased kidney.

REACT has been developed for the treatment of CKD, with a specific focus on stage 3b/4 patients at the highest risk of developing ESRD (eGFR less than 50 mL/min/1.73 m^2), in order to delay or prevent RRT (dialysis or kidney transplant). Diabetes is the most prevalent primary cause of CKD, followed by hypertension. [6,34] Patients with either of these conditions can potentially benefit therapeutically from REACT. Other smaller populations with primary conditions leading to renal disease that do not stem from immune disease, genetic disease-, or disease-causing poor blood supply to the kidneys could benefit from REACT: for example, those with kidney disease caused by former urologic obstructions or analgesic nephropathy.

For details of all completed and ongoing clinical studies with REACT, please refer to the Investigator's Brochure.

1.4.1 Type 1 Diabetes

Type 1 diabetes (T1DM), although less prevalent than T2DM, remains a significant risk factor for CKD. Recent data suggest that, over a 50-year period, up to 60% of patients with T1DM may progress to ESRD, and 7% progress to macroalbuminuria. [35] Although tight glycemic control paired with reductions in albuminuria and blood pressure have been associated with a reduced incidence of CKD, a significant proportion of patients with T1DM still progress to diabetic kidney disease (DKD) and ERSD. [36] Therefore, there remains an unmet medical need in this population to reduce the progression of CKD.

1.4.1.1 Safety Precautions for Inclusion of Type 1 Diabetics

Given the complications inherently associated with T1DM, such as hypoglycemic unawareness, hypoglycemia, and diabetic ketoacidosis (DKA), patients with such complications will not be included in the study. [37] Additionally, patients awaiting pancreas transplantation will also be excluded from the study. Excluding patients with T1DM at high risk of complications will help enhance the safety of REACT injection in this new patient population.

In addition to reviewing clinical diabetes control, subjects may be asked to share their glucose logs (finger stick or continuous glucose monitoring systems) with the Investigator to further evaluate the incidence and risk of hypoglycemic events and hypoglycemia unawareness.

1.5 Summary of Potential Risks and Benefits

1.5.1 Potential Risks

ProKidney' s platform utilizes culture expansion and injection of the patient's own (i.e., autologous) renal cells obtained through kidney biopsy. [29,32,38,39] As a result, the risk of cellular rejection and other immunologic responses, commonly seen following allogeneic cell transplantation, is not anticipated. Other formulation-related events are uncommon (see Section 1.5.1.3).

While the safety and efficacy evaluation of REACT is ongoing, the percutaneous administration and standard renal biopsy procedures have defined clinical risks and established procedures to manage those risks are defined in the protocol. In general, the risks associated with REACT treatment can be broadly divided into 3 categories; percutaneous kidney biopsy, preparation of REACT from component materials, and locoregional delivery or injection into the recipient kidney parenchyma. An assessment of potential risks associated with each of these steps is presented below in (Table 7, Table 8, and Table 9).

Table 7: Literature-Published Percutaneous Kidney Biopsy-Related Complications

Adverse Event	Reported/Expected Incidence	Risk Mitigation
**Hematoma**<br>-Perinephric<br>-Subcapsular<br>-Intra-parenchymal<br>-Retroperitoneal	Study Target <10%<br>Ranges reported:<br>4.3% - 17% [40-42]	-Clinical and Laboratory assessment<br>-Assessment of coagulation laboratory values and CKD bleeding risks<br>-Real-time Image guided biopsy (Ultrasound or CT when indicated per proceduralist)<br>-Automated core needle biopsy device<br>-16- or 18-gauge device (largest gauges required)<br>-Post-biopsy tract embolization (when indicated)<br>-Post-procedure observation and 24 hr. follow-up assessment<br>-Immediate post-biopsy and follow-up renal ultrasound evaluation during observation period<br>- Proceduralist Simulation video training with credentialing, training manual guide, and 24/7 access to Proceduralist Network team member.
Blood transfusion	<5% [42,43]	-Inclusion/Exclusion Criteria screening for incapacitating comorbidities<br>-Clinical and Laboratory assessment<br>-Real-time Image guided biopsy (Ultrasound or CT)<br>-Automated core needle biopsy device<br>-16- or 18-gauge device<br>-Post-biopsy tract embolization (if indicated)<br>-Post-procedure observation and 24 hr. follow-up assessment<br>-Post-procedure CBC<br>-Immediate post-biopsy and delayed renal ultrasound evaluation<br>-Proceduralist Simulation video training and credentialing, training manual, 24/7 Proceduralist Network
Radiologic Intervention	<0.5% [40, 43]	See blood transfusion
Nephrectomy	<0.1% [40, 43,44]	See blood transfusion
Death	<0.1% [40-42,45,46]	See blood transfusion
Cystoscopy	<1%	Post-procedure CBC, urinalysis, renal ultrasound-bladder
Hematuria	<4%	Post-procedure urinalysis

Adverse Event	Reported/Expected Incidence	Risk Mitigation
Hospital admission or readmission	<1%	Post-procedure observation and Day 1 clinic visit to determine hospital admission
**Pain***<br>-Nerve injury<br>-Flank and back<br>-Referred to shoulder, abdomen, and groin<br>-Local pain at skin site	<5% [42]	Post-procedure observation and pain assessment/management and 24 hr. follow-up assessment<br>* Pain is subjective and determined by visual or numeric measurements. Studies report pain estimates from 1-12% [42] and dependent on subgroup analysis.
**Infection**<br>-Retroperitoneal<br>-Pyelonephritis<br>-Subcutaneous	<1%	24 hr. and follow-up clinic assessment
Puncture of adjacent structures	<1 %	Real-time imaging observation during biopsy
Arteriovenous fistula	<1% [46]	Immediate post-biopsy and delayed renal ultrasound evaluation
**Biopsy site complications**<br>-Cutaneous bleeding<br>-Delayed bruising<br>-Local infection	<1%	-Standard sterile skin preparation and draping techniques<br>-Pre- and post-biopsy assessment and follow-up
**Intravenous line(s)**<br>-IV infiltration<br>-Infection<br>-Phlebitis<br>-Bruising/swelling	<5%	-Standard sterile skin preparation and IV-line insertion<br>-Pre- and post-biopsy assessment and follow-up
**Bladder symptoms**<br>-Urinary retention<br>-Dysuria	Minor <5%<br>Major <1% see cystoscopy	Pre- and post-biopsy assessment and follow-up
**GI Symptoms**<br>-Abdominal pain<br>-Nausea/vomiting<br>-Diarrhea<br>-Constipation	<1%	Pre- and post-biopsy assessment and follow-up
**Cardiac Symptoms**<br>-Chest pain<br>-Arrythmia<br>-Bradycardia/Tachycardia<br>-Shortness of breath<br>-Peripheral edema<br>-Hypertension<br>-Hypotension	<1%	Pre- and post-biopsy assessment and follow-up
**Cerebrovascular Symptoms**<br>-Strokes<br>-Transient ischemia<br>-Headaches	<1%	Pre- and post-biopsy assessment and follow-up

Adverse Event	Reported/Expected Incidence	Risk Mitigation
**Pulmonary Symptoms**<br>-Pneumothorax<br>-Shortness of breath<br>-Wheezing<br>-Cough<br>-Pneumonia	Pneumothorax <1% [47]	Pre- and post-biopsy assessment and follow-up
**Other Miscellaneous**<br>-Fever<br>-Falls<br>-Light-headedness	<1%	Post-procedure observation and Day 1 clinic visit
**Local Anesthetic**<br>Intravenous moderate sedation and general anesthesia	Allergic reactions: none to date in Phase II<br>Sedation reactions: None to date in Phase II<br>General Anesthesia reactions: None to date in Phase II	-Local anesthetic and moderation sedation medication allergy history and prior experiences<br>-Amount of anesthetic per body weight limit<br>-Minimal sedation provided for subject comfort and to limit motion during cell injection<br>-Post-procedure observation for delayed sedation effects.<br>-Anesthesia risks are multifactorial and dependent on ASA class and assessment

Table 8: Expected Incidence of Adverse Events and Complications of Injection Procedures Similar to the REACT Percutaneous Investigational Product (REACT) Injection

Adverse Event	Reported/Expected Incidence	Risk Mitigation
**Hematoma**<br>-Perinephric<br>-Subcapsular<br>-Intra-parenchymal<br>-Retroperitoneal	Target <10%<br>Ranges reported:<br>4.3% - 17% [40,42]	-Clinical and Laboratory assessment<br>-Assessment of coagulation laboratory values and CKD bleeding risks<br>-Real-time CT guided injection with intra-injection intermittent imaging and end of exam CT scan through bilateral kidneys.<br>-20-gauge outer guide needle<br>-25-gauge inner atraumatic injection needle<br>-Slow injection rate, 1 mL/min<br>-Post-procedure observation and follow-up renal ultrasound evaluation prior to discharge and 24 hr. in-clinic follow-up assessment and renal ultrasound evaluation<br>-Post-procedure CBC and urinalysis<br>-Proceduralist Simulation video training with credentialing, training manual guide, and 24/7 access to Proceduralist Network team member.

Adverse Event	Reported/Expected Incidence	Risk Mitigation
Blood transfusion	<5% [42,43]	-Inclusion/Exclusion Criteria screening for incapacitating comorbidities<br>-Clinical and Laboratory assessment<br>-Real-time Image guided CT IP injection<br>-Post-injection tract embolization (if indicated)<br>-Post-procedure observation and follow-up renal ultrasound evaluation prior to discharge and 24 hr. in-clinic follow-up assessment and renal ultrasound evaluation<br>-Post-procedure CBC and urinalysis<br>-Immediate post-injection CT and delayed renal ultrasound evaluation<br>-Proceduralist Simulation video training and credentialing, training manual, 24/7 Proceduralist Network
Radiologic Intervention	<0.5% [40, 43]	See blood transfusion
Nephrectomy	<0.1% [40, 43,44]	See blood transfusion
Death	<0.1% [40-42,45,46]	See blood transfusion
IP (REACT) extravasation<br>-extracapsular<br>-intra-arterial<br>-intra-venous<br>-urinary collecting system	<5% (predicted)	-Intermittent real-time CT imaging during and after each capsule puncture and cell injection deposit<br>-Slow injection rate, 1 mL/min<br>-Renal ultrasound during post-injection recovery and 24 hr. clinic follow-up.
Cystoscopy	<1%	Post-procedure CBC, urinalysis, renal ultrasound-bladder
Hematuria	<4%	Post-procedure urinalysis. Post-procedure observation and assessment/management and 24 hr. and Day 1-28 follow-up assessments
Hospital admission or readmission	<1%	Post-procedure observation and Day 1 clinic visit to determine hospital admission
**Pain***<br>-Nerve injury<br>-Flank and back<br>-Referred to shoulder, abdomen, and groin<br>-Local pain at skin site	<5% [42]	Post-procedure observation and pain assessment/management and 24 hr. follow-up assessment<br>* Pain is subjective and determined by visual or numeric measurements. Studies report pain estimates from 1-12% [42] and dependent on subgroup analysis.
**Infection**<br>-Retroperitoneal<br>-Pyelonephritis<br>-Subcutaneous	<1%	24 hr. and 7-28 day follow-up clinic assessments
Puncture of adjacent structures	<1 %	Real-time CT imaging observation during and post-injection

Adverse Event	Reported/Expected Incidence	Risk Mitigation
Arteriovenous fistula	<1% [46]	-Immediate post-injection CT scan and delayed renal ultrasound evaluation during observation.<br>-24 hr. and 7-28 day follow-up clinic assessments including 24 hr. ultrasound evaluation.
**Biopsy site complications**<br>-Cutaneous bleeding<br>-Delayed bruising<br>-Local infection	<1%	-Standard sterile skin preparation and draping techniques<br>-Pre- and post-injection assessment and follow-up<br>-24 hr. and 7-28 day follow-up clinic assessments.
**Intravenous line(s)**<br>-IV infiltration<br>-Infection<br>-Phlebitis<br>-Bruising/swelling	<5%	-Standard sterile skin preparation and IV-line insertion<br>-Pre- and post-injection assessment and follow-up<br>-24 hr. and 7-28 day follow-up clinic assessments.
**Bladder symptoms**<br>-Urinary retention<br>-Dysuria	Minor <5%<br>Major <1% see cystoscopy	-Pre- and post-injection assessment and follow-up<br>-24 hr. and 7-28 day follow-up clinic assessments.
**GI Symptoms**<br>-Abdominal pain<br>-Nausea/vomiting<br>-Diarrhea<br>-Constipation	<1%	-Pre- and post-injection assessment and follow-up<br>-24 hr. and 7-28 day follow-up clinic assessments.
**Cardiac Symptoms**<br>-Chest pain<br>-Arrythmia<br>-Bradycardia/Tachycardia<br>-Shortness of breath<br>-Peripheral edema<br>-Hypertension<br>-Hypotension	<5%	-Pre- and post-injection assessment and follow-up<br>-24 hr. and 7-28 day follow-up clinic assessments.
**Cerebrovascular Symptoms**<br>-Strokes<br>-Transient ischemia<br>-Headaches	<1%	-Pre- and post-injection assessment and follow-up.<br>-24 hr. and 7-28 day follow-up clinic assessments.
**Pulmonary Symptoms**<br>-Pneumothorax<br>-Shortness of breath<br>-Wheezing<br>-Cough<br>-Pneumonia	Pneumothorax <1% [47]	-Pre- and post-injection assessment and follow-up.<br>-24 hr. and 7-28 day follow-up clinic assessments.
**Other Miscellaneous**<br>-Fever<br>-Falls<br>-Light-headedness	<1%	24 hr. and 7-28 day follow-up clinic assessments.

Adverse Event	Reported/Expected Incidence	Risk Mitigation
**Local Anesthetic**<br>Intravenous moderate sedation and general anesthesia	Allergic reactions: none to date in Phase II<br>Sedation reactions: none to date in Phase II<br>General Anesthesia reactions: None to date in Phase II	-Local anesthetic and moderation sedation medication allergy history and prior experiences.<br>-Amount of anesthetic per body weight limit.<br>-Minimal sedation provided for subject comfort and to limit motion during cell injection.<br>-Post-procedure observation for delayed sedation effects.<br>-Anesthesia risks are multifactorial and dependent on ASA class and assessment

Table 9: Anticipated Investigational Product (REACT) Cell Complications

Adverse Event	Mitigation	Reported/Expected Incidence
Acute cell immunologic reaction	-No definite causation reported to date.<br>-Incidence is unknown and attribute <1%.<br>-No definite cell adverse causation after 120 REACT injections across all Phase I and II trials.<br>-SUSAR event 1.5 hours post-injection concluded: unlikely cell causation.	-Vital sign monitoring during cell injection<br>-Slow injection rate and documentation of injection time<br>-CT scan at completion of each cell deposit and at end of procedure<br>-Post-procedure observation and assessment/management and 24 hr. and Day 7-28 follow-up assessments<br>-Cell inflammatory markers
Acute cell expansion in kidney<br>-Kidney tissue expansion<br>-Kidney capsule expansion	-No obvious morphologic changes identified during CT scan following injection.<br>-Transient asymptomatic decreased blood pressure observed at time of injection with return to normal pressure.	CT scan at completion of each cell deposit and at end of procedure.
Allergic reaction	Potential reaction to DMSO, Dextran-40, aminoglycosides	-Allergy history<br>-Acute allergy assessment and triage.
Latent cell changes	Expect low potential risk for tumorigenicity	Renal MRI assessment during clinical trial start to end-of-study.
Renal infarct, renal fibrosis or scarring, loss of glomerular filtration rate	Two SUSARs have been reported indicating a potential for renal infarct after the second injection of REACT product. One was associated with renal fibrosis and loss of eGFR	Proceduralists are to avoid areas previously biopsied or REACT injection interventions for supplemental injections in the same kidney.<br><br>Monitor for pain, nausea and other symptoms of acute renal infarct after REACT injections.

Abbreviations: CT = computed tomography; DMSO = dimethyl sulfoxide; MRI = magnetic resonance imaging; REACT = renal autologous cell therapy; SUSAR = serious and unexpected suspected adverse reaction.

1.5.1.1 Management of Anticoagulation Medication During Biopsy and REACT Injection

All anticoagulation medications such as warfarin, low-molecular-weight heparin (LMWH), enoxaparin, anti-platelet medications (aspirin, nonsteroidal anti-inflammatory drugs [NSAIDs], clopidogrel, Persantine), factor Xa inhibitors and other novel oral anticoagulants (NOAC) are discontinued for 7 days prior and 7 days following biopsy and REACT injection. In addition, other herbal, over the counter products (fish oils, omega 3 medications) or other supplementary products, are discontinued for the same time. ProKidney does not endorse anticoagulation bridging protocols with cardiac stents.

Coagulation parameters are obtained before the biopsy and each injection intervention per the protocol study visit schedule and include INR, APTT, prothrombin time, platelets, hemoglobin and hematocrit. The values of each laboratory test are required to be within the accepted range for percutaneous renal procedures. [42,43,47-50]

1.5.1.2 Percutaneous Renal Biopsy

Autologous kidney cells will be obtained from a patient with diabetic CKD via a standard renal biopsy procedure following standard medical biopsy practice [47,51,52] and consistent with standard operating procedures (SOP) at participating clinical trial sites. One renal biopsy (minimum two tissue cores) is required to obtain sufficient renal cortical tissue for the production of REACT. A 16-gauge tissue biopsy measuring approximately 10 mm in length removes 0.01-0.02% of the average total volume of the diseased kidney and usually contains only 0.001% of the total number of glomeruli [53], so will have minimal to no impact on overall kidney function. The tissue biopsy causes minimal morphologic changes causing a small morphology, linear track through the kidney. Lesions associated with renal biopsy are largely undetectable and observed histologically in only 5.5% of serial kidney sections taken 1 month after biopsy. [54]

Renal biopsies performed for suspected renal parenchymal disease are of low risk and most often conducted on an outpatient basis in the US. [55] Biopsy complications have decreased with the use of image guidance, predominantly with ultrasound (CT for difficult cases) and the use of automated biopsy devices. Kidney damage at the biopsy site can include vascular damage and infarction and correlated with the preclinical necropsy descriptions of renal scar formation. The severity of these changes depends on the size and number of vessels damaged during the biopsy procedure. [54] Hemorrhage/hematoma is the most common adverse seen following a routine renal biopsy and occurs from 4-17%, with a higher incidence observed with CT versus ultrasound imaging. Post-biopsy bleeding requiring a blood transfusion vary by study, however large

meta-analysis and regional studies indicate a <5% incidence of blood product administration. The occurrence of post-biopsy interventional radiology angiography/vessel embolization (<0.5%), nephrectomy (< 0.1%) and death (<0.1%) are low. [40,42-44] Microscopic hematuria following biopsy is present in almost all patients with little clinical significance and macroscopic (gross) hematuria occurs in only, <4% of the patients [55,56] and generally resolves by 24-hour post-biopsy. [57]

Less common biopsy complications include blood/clots in the bladder, arteriovenous fistula (1%), urine extravasation (urinoma), puncture of adjacent structures, and conscious sedation reactions. [39,46,58-60]

When performed by qualified interventional proceduralists, a renal biopsy properly targeted toward the lower pole cortex causes limited renal damage. [61] Furthermore, the access track can be embolized with hemostatic biodegradable material, to further reduce acute or delayed risk of significant bleeding.

1.5.1.3 REACT Components

ProKidney's autologous REACT is a frozen formulation of SRC in cryopreservation media at a concentration of approximately 100×10^6 cells/mL ±20%. REACT is stored at less than or equal to -150°C until use.

Selected Renal Cells: A dose-limiting toxicity relevant to human patients was not identified in the *in vivo* studies conducted. No unanticipated findings were observed in multiple animal studies, species, and disease conditions with administration or re-administration of syngeneic or autologous SRC to the chronically diseased kidneys.

REACT is an autologous cellular medicinal product which has the potential to become contaminated and subsequently cause infection in the study subject at the time of administration. This risk is greatly minimized by the use of a Good Manufacturing Practice (GMP)-compliant manufacturing facility and aseptic processing best practices. Prior to the release of the final product from the GMP facility, microbiological testing (bacterial, fungal) is performed and must be negative.

CryoStor CS10 Freeze Medium: The only excipient added is the commercial cryoprotectant CryoStor CS10. CryoStor CS10 is a common and widely used excipient for human cell-based

medicinal products. The excipient is cGMP manufactured with USP grade/highest-quality components. CryoStor CS10 is pre-formulated with 10% DMSO and contains Dextran-40.

Dimethyl Sulfoxide (DMSO): DMSO is a constituent of CryoStor CS10 Freeze Medium and has been used as a medicine and pharmacological agent in humans since the 1960s and its current primary uses are for cell cryopreservation, treatment of interstitial cystitis, and as a penetrating vehicle for various drugs. A recent systematic study investigating the adverse effects associated with DMSO found that most adverse reactions are transient and mild. [62] The dose of DMSO was found to have an important role in the occurrence of adverse reactions, however DMSO was safe to use in small doses. Gastrointestinal and skin reactions were the most common reported adverse reactions to DMSO. In practice, it is commonly recommended to limit exposure of DMSO to 1 g DMSO/kg body weight/day by intravenous delivery (IV). [63] For context, the DMSO content in the REACT final product formulation is ~79-fold lower than the commonly recommended intravenous DMSO limit and ~40-fold lower than a calculated DMSO limit for kidney exposure. To mitigate the risk of reactions to DMSO, patients with known severe reaction or hypersensitivity to DMSO will be excluded from study.

Dextran 40: Dextran 40 is a constituent of CryoStor Freeze Medium CS10 used to formulate the final REACT product. Severe anaphylactoid reactions have been reported with Dextran infusion. In a review of Dextran infusion reactions reported to the Food and Drug Administration (FDA) Adverse Event Reporting System between 1969 and 2004, [64] 90 events classified as severe anaphylactoid over the 37-year period. Although extremely infrequent, concern for consideration of the possibility of a Dextran 40 reaction can be mitigated by the use of Dextran 1 pre-treatment, hydrocortisone, or other corticosteroid premedication, in addition to antihistamine.

If an event occurs which is assessed as related to the investigational product, REACT, then the event will be immediately reviewed by the Investigator and Medical Monitor.

1.5.1.4 REACT Injection into the Kidney

REACT will be administered by a needle (20-gauge outer and 25-gauge inner) and syringe connecting tube delivery system through a small needle system with minimal penetration of the kidney capsule and depositing REACT at multiple locoregional sites within the kidney cortex. The recipient kidney will be injected with REACT containing 100 x 10^6 SRC per mL ±20% of product to deliver 3.0 x 10^6 SRC/g of kidney tissue. A percutaneous technique with CT image guidance will be used to access the kidney for REACT delivery. The percutaneous approach into the kidney has been used for decades with high degrees of safety. The safety profile for other

common renal interventions, that include nephrostomy catheter insertion, renal mass biopsies, cysts/abscess drainages, and small renal cell carcinoma ablations have been published. [60,65] For injection of REACT, the percutaneous needles are small and the procedure less complex, offering the safety advantages of an outpatient procedure with local skin anesthesia, conscious sedation and short recovery period. Similar outpatient renal procedures, such as renal tumor ablations, have shown a low safety risk profile by reducing immobilization and recovery periods compared to inpatient admissions. [66] Safety measures performed during REACT treatment and post-injection follow-up to reduce the potential for excessive bleeding and other adverse events include clinical and laboratory assessment prior to procedure, temporary cessation of anticoagulation medications and follow-up renal ultrasound during the recovery period. [49,67-70] Interventionalists should try to avoid previously biopsied or REACT injected areas of the kidney.

Similar to a standard biopsy procedure, [71] the greatest risk of targeted REACT delivery to the kidney is anticipated to be hemorrhage. No unanticipated tissue reactions were observed across a wide dose range/gram kidney (1.5-15 x 10^6/g) in multiple animal studies. Similar findings have been reported independently in the literature in which intra-renal injections of renal cell homogenates injected into rodent kidneys produced no significant adverse events following injection via a needle delivery. [72] Morphological effects of REACT delivery/biopsy of the kidney are consistent with those reported for repeat kidney biopsies taken from canines - a mature connective tissue track, and no functional deficits associated with these minimal structural changes. [54] In isolated cases, increasing intra-capsular kidney water volume in canines resulted in elevated intra-kidney pressure and were associated with transient increases in kidney weight and systemic blood pressure [73,74]; however, in our pilot canine study where blood pressure was monitored, no adverse effects on blood pressure were observed following volume escalation of up to 6 mL per kidney of REACT.

REACT and SRC injection led to the rodent-specific hemodynamic effects of apnea and bradycardia in the terminal rodent models of CKD; however, these effects were not observed following the injection of SRC in the canine pharmacology study, in two canine pilot studies designed to assess the short-term effects of volume administration on blood pressure during and following REACT injection in the intact mongrel canine, or in GLP toxicology studies. This rodent and model-specific hemodynamic response can be potentially attributed to 1) altered hemodynamics of the severely mass-reduced rodent remnant kidney, [75] 2) transient changes in kidney interstitial pressure administration triggering a central autonomic response, [73,74] and possibly 3) under-perfusion of tissue or acute hypoxia from bleeding following injection to the kidney. Pre-treatment with atropine, [76] a competitive antagonist of the parasympathetic nervous

system, helped mitigate this species-specific volume-induced bradycardia and apnea with the associated death. Apnea, bradycardia and/or death were not observed in the canine pharmacology studies or GLP toxicology studies, further supporting the species and model specificity of the phenomena. Such events have not been reported in the clinical programs to date.

1.5.2 Identified Risks

At this time, there are no specific warnings or precautions associated with the use of REACT. However, warnings and precautions for a renal biopsy and the percutaneous injection procedure must be considered with use of this product. The risks of renal biopsy have been well characterized over the 70+ years this procedure has been developed and used. Percutaneous small needle interventions in the kidney have a shorter history and shown to be safe. Secondary effects of renal bleeding have been described and include anemia, acute kidney injury, hematoma mass effect on the parenchyma causing hypertension and pain. Pain post-biopsy has been reported as <5%, including cutaneous puncture site, musculoskeletal and perinephric referred sources. [42] Table 10 provides a summary of safety data in RMCL-002 as of March 24, 2022 for kidney biopsy and REACT injection bleeding risks.

Table 10: RMCL-002 Safety Data – Kidney Biopsy (N=83), REACT Injection (N=126) and REACT Cell Adverse Events

	Events/Total	%	Published Report
**Kidney Biopsy**			
Major hematoma [a]	1/83	1.2	< 5% all, <0.5%, w/ interventions [30,77]
Minor hematoma [b]	10/83	12	4 – 17% [30,78,79]
Total complications	11/83	13.2	
No complications	72/83	87	
**REACT Injection** [c]			
Major hematoma	1/126	.008	
Minor hematoma	6/126	4.8	
Total complications	7/126	7.255	
No complications	119/126	92.844	
**All Procedure Outcomes**			
All major hematomas	2/209	1.0	
All minor hematomas	16/209	7.7	
No complications	162/209	91.4	
**REACT Cell Product**			
Anaphylaxis, cytokine, et al.	0/209	0	

a. Major hematoma defined by blood transfusion, extended hospitalization, intervention, nephrectomy, death.
b. Minor hematoma defined as small (<2 cm), resolved, stable or smaller compared to prior imaging.
c. No similar locoregional kidney cell injection procedures are published. Complication percentages extrapolated from other renal interventions, e.g., nephrostomy, renal ablations and mass biopsies.

1.5.3 Potential Benefits

To date, clinical studies suggest that treatment with REACT in patients with T2DM and CKD can positively impact renal function by stabilizing eGFR or attenuating the rate of eGFR decline. Other improvements observed with REACT treatment include stabilization and/or reduction in UACR increased kidney cortical thickness, and improved hemoglobin levels, suggestive of a reduced risk of anemia. Please refer to the Investigators Brochure for additional information.

The potential benefits of REACT will be evaluated in the current trial using events involving eGFR, dialysis and UACR (see the primary endpoint defined in Section 2). Therefore, the potential exists for subjects participating in this clinical study to realize therapeutic benefit from REACT treatment, such as a possible reduction in the rate of progression of CKD as indicated by one or more renal function benefits including reduced, stabilized or improved eGFR, albuminuria, anemia, and possible improvement in bone-mineral dysfunction.

2 STUDY OBJECTIVE AND ENDPOINTS

ProKidney is currently developing REACT, which is a cell-based advanced therapy, with the aim of improving renal function in subjects who have T1DM or T2DM with CKD. Therapeutic intervention with REACT is intended to delay the need for RRT (hemodialysis, peritoneal dialysis or transplant) which, based on the current standard of care, is predictable for subjects in Stage CKD 3a, 3b, and 4 who have moderate to severe albuminuria.

The purpose of this study is to assess the safety, efficacy, and durability of up to two REACT injections given 3 months (+60 days) apart and delivered percutaneously into biopsied (1 injection) and non-biopsied (1 injection) contralateral kidneys on renal function progression in subjects with CKD and either T1DM or T2DM. To evaluate this objective, two regimens will be investigated: two scheduled REACT injections (Cohort 1) and one REACT injection followed by renal function trigger-based redosing criteria (Cohort 2).

Each subject’s annual rate of renal decline before screening, based on adequate historical, and clinical data within 24-months prior to the Screening Visit, will serve as the individual subject’s reference to estimate the change of rate of progression of renal insufficiency.

It is proposed that REACT treatment will reduce the rate (slope) of eGFR decline and improve renal function over the at least 18-month period following the last REACT injection.

2.1 Objective

The objective of this study is to assess the safety, efficacy, and durability of up to two REACT injections delivered percutaneously into biopsied and non-biopsied contralateral kidneys on renal function progression in two different cohorts of subjects with T1DM or T2DM and CKD:

- Cohort 1: two scheduled REACT injections given 3 months apart (+60 days) with at least 18-month follow-up.
- Cohort 2: one scheduled REACT injection with a possible second REACT injection upon meeting a redose trigger, second injection would be within at least 30 days of meeting a renal function redose trigger with sustainment (triggered between 3-15 months following the first REACT injection), with at least 18-month follow-up after last REACT injection.

2.1.1 Primary Efficacy Endpoint

The primary efficacy endpoint is as follows:
Change from pre-injection to post-last injection total (acute + chronic) slope of estimated glomerular filtration rate (eGFR) using the 2009 Chronic Kidney Disease Epidemiology Collaboration (CKD-EPI 2009) serum creatinine equation. [80]

2.1.2 Primary Safety Endpoint

The primary safety endpoint is as follows:
Procedure and investigational product-related treatment-emergent adverse events (TEAE) obtained through at least 18 months after the last REACT injection.

2.2 Secondary Efficacy and Exploratory Endpoints

2.2.1 Secondary Efficacy Endpoints

Secondary efficacy endpoints will include the following:

1. Estimated glomerular filtration rates, determined using CKD-EPI 2009, from first injection to at least 18 months after the last REACT injection.
2. Time from first injection to at least 40% reduction in eGFR, using the 2009 CKD-EPI serum creatinine equation, sustained for 30 days.
3. Time from first injection to eGFR <15 mL/min/1.73m² using the 2009 CKD-EPI serum creatinine equation, sustained for 30 days and/or chronic dialysis, and/or renal transplant.
4. Time from first injection to Increase of UACR of at least 30% and of at least 30 mg/g, using the random urine microalbumin/urine creatinine ratio sustained for 90 days.
5. Time from first injection to renal or cardiovascular death.

6. Time from first injection to earliest of the following:
    - At least 40% reduction in eGFR, using the 2009 CKD-EPI serum creatinine equation, sustained for 30 days or
    - eGFR <15 mL/min/1.73m² using the 2009 CKD-EPI serum creatinine equation, sustained for 30 days and/or chronic dialysis, and/or renal transplant or
    - Increase of UACR of at least 30% and of at least 30 mg/g, using the random urine microalbumin/urine creatinine ratio sustained for 90 days or
    - Renal or cardiovascular death.
7. Time from first injection to earliest of the following:
    - At least 40% reduction in eGFR, using the 2009 CKD-EPI serum creatinine equation, sustained for 30 days or
    - eGFR <15 mL/min/1.73m² using the 2009 CKD-EPI serum creatinine equation, sustained for 30 days and/or chronic dialysis, and/or renal transplant or
    - Renal or cardiovascular death.
8. Percent of subjects who have the same or reduced 5 year risk of ESRD and 2 year risk of ESRD at 12 and 18 months after the last injection.

The time for each time-to-event endpoint will be calculated starting on the day of injection.

2.2.2 Exploratory Endpoints

The following exploratory endpoints will be evaluated during this trial:

1. Changes from baseline in renal volume.
2. Changes from baseline in cortical thickness.
3. Changes from baseline in hemoglobin.
4. Changes from baseline in hematocrit.
5. Changes from baseline in calcium.
6. Changes from baseline in phosphorus.
7. Changes from baseline in parathyroid hormone.
8. Changes from baseline in systolic blood pressure.
9. Changes from baseline in diastolic blood pressure.
10. Changes from baseline in potassium.
11. Changes from baseline in bicarbonate.
12. The time from first injection to all-cause mortality.

13. Incidence of changes in antihypertensive therapy (i.e., increase, decrease).
14. Time to increase in antihypertensive therapy.
15. Incidence of changes in metabolic acidosis therapy (i.e., increase, decrease).
16. Time to increase in metabolic acidosis therapy.
17. Incidence of changes in hyperkalemia therapy (i.e., increase, decrease).
18. Time to increase in hyperkalemia therapy.
19. Incidence of changes in anemia therapy (i.e., increase, decrease).
20. Time to increase in anemia therapy.
21. Incidence of changes in bone and mineral dysmetabolism therapy (i.e., increase, decrease).
22. Time to increase in bone and mineral dysmetabolism therapy.

2.2.3 Secondary Safety Endpoints

The secondary safety endpoint will be procedure-related death. However, additional endpoints will be taken into consideration when evaluating safety, namely:

1. Physical examination results
2. Vital signs
3. Safety laboratory analytes
4. 12-lead ECG results
5. MRI results.

3 INVESTIGATIONAL PRODUCT

3.1 Description of Investigational Product

Renal autologous cell therapy product is a cryopreserved suspension of autologous SRC formulated in a cryopreservation solution that has been aseptically dispensed into sterile single-use cryovials. REACT is stored in vapor phase liquid nitrogen (less than -150°C) until needed. Immediately prior to use at the clinical site, the product is rapidly thawed, and the cell suspension is injected into the recipient's kidney with no further manipulations. Table 11 presents an overview of the investigational product. Refer to the Investigator's Brochure for a detailed description of SRC and REACT as well as the manufacturing process.

Table 11: Investigational Product

	Investigational Product
**Product Name**	REACT (Renal autologous cell therapy)
**Dosage Form**	Renal cells obtained from autologous kidney biopsy tissue will be expanded and SRC selected. Selected renal cells will be formulated in a cryopreservation solution (CryoStor Freeze Medium CS10) at a concentration of 100×10^6 cells/mL ±20%. This aseptically manufactured cell preparation will be dispensed into sterile, single-use cryovials and stored in vapor phase liquid nitrogen (< -150°C) until needed
**Unit Dose**	The dose of REACT will be adjusted to 3×10^6 cells/g estimated kidney weight determined by magnetic resonance imaging (MRI)
**Route of Administration**	Percutaneous injection into the cortex of the biopsied kidney. In subjects receiving the 2nd injection, it will be performed in the contralateral kidney (i.e., the kidney that did not receive the 1st injection)
**Physical Description**	Labeled, single-use cryovial of REACT
**Manufacturer**	ProKidney, Winston-Salem, North Carolina, USA
**Labeling (traceability)**	Full traceability of the autologous starting kidney biopsy tissue, through manufacturing into an investigational medicinal product, and return to participant, will be maintained through use of a unique identification sequence, such as the Single European Code Donor Identification Sequence (SEC-DIS), or equivalent, per country specific requirements.

3.2 Procurement and Manufacture of REACT

Renal autologous cell therapy will be manufactured in a GMP facility at ProKidney, LLC (Winston-Salem, North Carolina, USA).

3.2.1 Biopsy

The biopsy material will be collected using standard percutaneous imaged-guided techniques from either the left or right kidney. A minimum of two cores using a 16-gauge biopsy needle/punch or four cores using an 18-gauge biopsy needle/punch must be collected to provide sufficient material for the manufacture of REACT. When the biopsy material is received at ProKidney, the samples will be labeled, and strict documentation measures followed to ensure that product traceability is maintained. If the biopsy material cannot be used, the subject will be discontinued from the study.

3.2.2 Culture Expansion and Selection of SRC

Manufacturing of REACT begins with isolation of cells from the renal biopsy tissue. Renal cells are isolated from the kidney tissue by enzymatic digestion and expanded using standard cell culture techniques over the course of several weeks. The desired population of renal cells are selected by density gradient separation of the expanded cells which yields the SRC population of REACT.

The REACT manufacturing process is designed to deliver a product in approximately 12 weeks from subject biopsy to product injection.

3.2.3 Formulation

3.2.3.1 Active Substance

Selected renal cells are a population of renal cells with regenerative potential manufactured from kidney biopsy tissue obtained from the subject (autologous). Just prior to formulation, the cells are washed with Dulbecco's phosphate buffered saline (PBS) to remove process residuals and the cells are pelleted.

3.2.3.2 Excipients

CryoStor Freeze Medium CS10 is a commercially available GMP manufactured cryoprotectant solution. It is added neat to the washed SRC pellet to resuspend and formulate the final product at a concentration of 100×10^6 cells/mL ±20%. The precise volume of CS10 contained in the cryovial is variable since it is based on a calculation needed to obtain an SRC concentration of 100×10^6 cells/mL ±20%. The maximum dosing volume for injection is 8.0 mL/vial.

3.2.4 REACT Product for Injection

The REACT product will be manufactured and shipped to the clinical site as soon as possible after renal biopsy. It is the responsibility of the site to ensure REACT shipments can be delivered directly to site personnel. REACT will be injected into the biopsied kidney of eligible subjects using a percutaneous image guided approach. The percutaneous method will employ a standardized image guided interventional techniques (such as that utilized in the other kidney intervention cases, such as nephrostomy catheters and treatment renal masses by thermoablation). [66] Interventionalists should try to avoid the same tracks, trajectory, location of prior renal biopsies and REACT injections.

For details of the storage and disposal of renal cells not used to manufacture REACT product, see Section 3.8.3.

3.2.5 REACT Dose

Based on Phase 1 and 2 data to date, the dose of REACT will be 3×10^6 cells/g KW^{est}. Since the concentration of SRC per mL of REACT is 100×10^6 cells/mL ±20%, the dosing volume will be 3.0 mL for each 100 g of kidney weight. Using this dosing regimen, Table 12 shows the dosing

volume and number of SRC to be delivered relative to estimated kidney weight. The maximum volume of REACT injected into the kidney during an injection will be 8.0 mL. The volume of REACT to be administered will be determined by pre-procedure renal MRI or CT volumetric 3-dimensional (3D) evaluation.

Table 12: REACT Dosing Relative to Estimated Kidney Weight

Estimated Kidney Weight (gKW^{est}) [a,b]		REACT Dosing Volume (mL)	SRC Delivered (Number of Cells × 10^6)
Median Weight (g)	Weight Range (g)		
100	95 – 108	3.0	300
117	109 – 125	3.5	350
133	126 – 141	4.0	400
150	142 – 158	4.5	450
167	159 – 175	5.0	500
183	176 – 191	5.5	550
200	192 – 208	6.0	600
217	209 – 225	6.5	650
233	226 – 241	7.0	700
250	242 – 258	7.5	750
— — —	>259	8.0 [c]	800

Abbreviations: CT = computed tomography; g KW^{est} = grams estimated kidney weight; MRI = magnetic resonance imaging; REACT = renal autologous cell therapy; SRC = selected renal cells.

a. The dose of REACT will be 3 $\times 10^6$ cells/g estimated kidney weight.
b. Kidney weight will be estimated from the results of an MRI study performed on or after the Screening Visit until Day 0 (renal biopsy). If a subject cannot undergo MRI then CT will be substituted to obtain kidney size and volume.
c. 8 mL will be the maximum dosing volume (mL).

The dose of REACT will be based on kidney volume calculated from the results of the MRI study performed from the time of the Screening Visit prior to the renal biopsy. In contrast to other methods, measurements of renal volume using MRI are more accurate, and acquire true tomographic data along any orientation without the risk of ionizing radiation or nephrotoxic contrast agents. Renal volume measurements (mL) estimated from MRI are approximately 92 to 97% of dry weight measurements in grams for isolated organs trimmed of perirenal fat. [38] If a subject cannot undergo MRI then CT will be substituted to obtain kidney size and volume measurements per pre-described formulas.

As a conservative approach, the REACT dose will be calculated using a conversion of 1 g equals 1.0 mL. This ensures that subjects will not receive REACT doses higher than those previously tested in animal studies.

3.3 Preliminary Clinical Evidence Supporting 2 REACT Injections

It is planned that all subjects in Cohort 1 will receive two REACT injections, whereas subjects in Cohort 2 will receive at least one REACT injection and may receive a second REACT injection based on renal function triggers being met.

Of importance, all subjects receiving two REACT injections will receive one injection in each kidney. The second REACT injection will be injected in the contralateral kidney (i.e., the kidney not previously injected with REACT). This may result in an enhanced therapeutic REACT effect as, from an anatomical and symmetrical perspective, DKD generally results in bilateral renal dysfunction. Therefore, injection of REACT in both kidneys may further enhance stabilization or renal function.

The scientific rationale for administering two REACT injections, is based on data from non-clinical studies and Phase 2 trials (RMCL-002 and REGEN-003), which indicate that the autologous renal cells in REACT delays progression of CKD by augmenting renal structure and function. [80-86] In non-clinical study BAe200 [29,78], an experimental model of diabetic nephropathy (secondary to metabolic syndrome), demonstrated that two cell injections administered approximately 4 months apart elicited a regenerative response that significantly improved survival (80% in the SRC treated- group compared with 20% in the untreated control group) and stabilized disease progression through improvements in nephron function (glomerular, tubular and collecting duct). This included observations of reduction in urinary proteinuria following the second injection and histomorphology improvements in renal architecture such as reduced fibrosis and inflammatory markers at end of study. [29]

Importantly, the more cells that can be infused, the greater the potential improvement in renal function. However, the total number of cells that can be delivered into a kidney at one time is limited by the size of the kidney, as well as the inelasticity of the renal capsule. Consequently, a second injection, in the same or contralateral kidney, provides a mechanism to administer greater numbers of SRCs, which could enhance or prolong therapeutic effects, as observed in non-clinical studies. Additionally, the presence of diabetic renal dysfunction is bilateral, therefore bilateral delivery of REACT into both kidneys could provide additional therapeutic benefit by targeting the dysfunction present in both kidneys.

Apart from increasing SRC numbers by administering two REACT injections, the duration of effects can be evaluated. The processes by which functional nephrons become disabled in

kidneys with CKD may, over time, adversely affect "new" cells delivered via REACT injection. Consequently, REACT might not result in long-term, therapeutic benefit. Exploring the effects from a second REACT injection, given at an appropriate interval after the first injection, would address this question.

Currently available data from Phase 2 study, RMCL-002 indicates that participants randomized to receive two injections of REACT, in addition to standard of care medications, experience a stabilization in kidney function as measured by average change in eGFR measured during study visits at three, six, nine and twelve months after receipt of their second REACT injection. Similarly, study participants who received two injections of REACT have reduced urinary albuminuria, in comparison to participants who only receive standard of care medications.

Furthermore, during Cohort 2, administration of a second REACT injection will be triggered based on renal function criteria, thereby allowing the evaluation of successive administration of SRCs following renal decline, further elucidating the optimal timing, safety, efficacy, and durability of multiple REACT administrations to prevent need for dialysis.

3.4 Safety of 2 REACT Injections

To assess the safety of administering two doses of REACT into the biopsied kidney, a canine Good Laboratory Practice toxicology study was conducted (CQL001). Similar to the clinical study design, study animals (n=8) underwent renal biopsies at 4 to 6 weeks prior to baseline. Each dose was delivered into both kidneys at baseline and at 3 months; animals were observed for 6 months following the baseline injection. While control animals received PBS, REACT-treated animals received a 2-fold greater dose than that used in clinical study.

Briefly, no detrimental effects of two doses of REACT into the biopsied kidney were observed in comparison to control animals 6 months after baseline treatment. Pathological assessment showed no REACT safety-related (macroscopic or microscopic) findings in either the target organ (kidney) or non-target organs examined. No treatment-related kidney findings were noted following enhanced evaluation of eight areas of each kidney (three stains per area), including assessment and scoring of 150 glomeruli per kidney. All kidneys appeared normal, apart from changes related to injection site scars. There were no signs of renal insufficiency, and no indications of decreased GFR. Detailed information for this study is provided in the Investigator's Brochure.

Preliminary results of the prospective, randomized, double-arm deferred treatment, open-label, repeat dose, multi-center, Phase 2 study (RMCL-002) have suggested a robust safety profile. Additionally, two groups, including a clinical advisory body and a Data and Safety Monitoring Board (DMC) have reviewed the interim data and concluded that the trial should continue to gather additional data for definitive assessments on the potential benefit of REACT on the progression of CKD in T2DM patients.

Under RMCL-002 83 patients have enrolled; 42 randomized to deferred treatment (standard of care for 12 months prior to REACT injection) and 41 to active treatment (immediate REACT injection post randomization).

REGEN-003, a Phase 2, multi-center, prospective, open-label study in the United States enrolled 10 patients with severe, rapidly progressing, pre-dialysis Stage 4 CKD. Patients received up to two injections of REACT into the biopsied kidney, beginning as soon as the REACT product can be manufactured and shipped to the clinical site, followed by a second REACT injection in 6 months (+4 weeks).

Ten patients were enrolled, aged 30 to 65 years, with a diagnosis of T2D and CKD due to diabetic nephropathy. Enrolled patients had severe, rapidly progressing, Stage 4 CKD defined as an eGFR between 14 and 20 mL/min/1.73m^2 and not requiring renal dialysis at screening.

In order to further enhance safety and efficacy of two REACT injections, subjects receiving a second injection will be injected in the contralateral kidney, i.e., the kidney without a previous REACT injection.

3.5 Investigational Product Packaging

The REACT product is formulated as a suspension of SRC cells at a concentration of 100×10^6 cells/mL ±20% in CryoStor Freeze Medium CS10, a commercially available GMP manufactured cryoprotectant solution containing DMSO 10% (v/v). The final formulated product is dispensed aseptically into a cyclo-olefin copolymer cryovial with a plastic elastomer closure. The maximum dosing volume is 8.0 mL/injection, however the specific fill volume of a cryovial is dependent on the required dose volume as determined by the subject's estimated kidney weight. REACT product has been formulated for storage in vapor phase liquid nitrogen (< -150˚C) until needed.

3.6 Investigational Product Label

The REACT product is made from expanded autologous SRC obtained from each individual subject's kidney biopsy and is, therefore, subject-specific. Each package containing the REACT product will have affixed to it a label containing the following: "FOR AUTOLOGOUS USE ONLY". In addition, the label will indicate that REACT is for "Investigational Use Only". Secondary labeling information will specify the volume(s) to be withdrawn from thawed cryovial(s) to achieve subject dosing requirements. For each country participating in the study, labels will comply with the relevant labeling requirements for investigational products in that country.

3.7 Investigational Product Transportation

All biopsy specimens will be transported to ProKidney, LLC using packaging mandated in compliance with all relevant US Department of Transportation/International Civil Aviation Organization/International Air Transport Association regulations and according to individual carrier guidelines. Further details of biopsy specimens can be found in the Study Reference Manual.

Renal autologous cell therapy product has been formulated for storage in vapor phase liquid nitrogen (< -150°C) until needed. Upon request for shipment, REACT product will be transported from ProKidney, LLC to the clinical site. Shipping containers will be monitored for temperature and validated to maintain required temperature for at least several days beyond the maximum expected transportation time between manufacturing facility and the clinical site.

At the clinical site, the REACT product is stored in the shipping container until needed. The REACT product is thawed immediately prior to use and the cell suspension is injected into the subject's kidney without any additional product manipulation.

3.8 Investigational Product Accountability

Investigational product accountability and traceability is ultimately the responsibility of the Investigator once it is released by the Sponsor. However, this responsibility may be delegated to a suitably-qualified individual who has had appropriate study-specific training and whose name is listed on the Delegation of Responsibility Log for this task.

Detailed records will be maintained to allow for accurate accountability of the Investigational drug product in accordance with applicable Sponsor and clinical site procedures. These records

will include details about the transfer of renal biopsy specimens from the clinical site to ProKidney, transfer of REACT drug product from ProKidney to the clinical site, internal site transfers, injection of study drug to autologous subjects, the number of stored specimens remaining at ProKidney, and disposal of unused materials.

3.8.1 Investigational Product Handling and Disposal

Since transmission of HIV and other blood-borne pathogens can occur through contact with contaminated needles, blood, and blood products; appropriate blood and secretion precautions will be employed by all personnel in the shipping and handling of all specimens for this study, as currently recommended by the Centers for Disease Control and Prevention and the National Institutes of Health.

All material containing REACT product will be treated and disposed of as hazardous waste in accordance with governing regulations and clinical site procedures.

3.8.2 Disposition of Stored Specimens

Biopsy material will be stored in the vapor phase of a liquid nitrogen freezer at ProKidney, LLC. Packaged REACT product will be received by the study site and only Investigators or suitably qualified- individuals who have received appropriate study-specific training and whose names are listed on the Delegation of Responsibility Log will have access to these specimens.

3.8.3 Unused Biopsy Material

Renal cells that may have been frozen but not used to manufacture REACT will remain in the vapor phase of a liquid nitrogen freezer at ProKidney until the End-of-Study (EOS) Visit. At that time, if these renal cells are no longer needed, they will be de-identified of all personal information and stored in the vapor phase of a liquid nitrogen freezer for a maximum of 10 years. The aim is to test these renal cells in laboratory research studies.

During the informed consent process, each participant must provide written consent for the storage and future use of autologous cells not used for REACT injection if they consent to the long-term storage of unused specimens. If a participant consents to the use of future cells, ProKidney will only use the cells after all REACT injections have been completed for the participant. In addition, participants may decide at any point during the study not to have their unused renal cells stored. In this case, ProKidney will destroy all known remaining samples attributed to that participant after their injections are complete. All requests for destruction of

samples must be made in writing to info@prokidney.com. Similarly, participants must have the option of having their cells destroyed upon completion of the study.

4 INVESTIGATIONAL PLAN

4.1 Study Administrative Structure

ProKidney is the Sponsor of this study. The following organizations will provide services for this study under contracts with the Sponsor:

- ProKidney, LLC, 3929 Westpoint Blvd. Suite G, Winston-Salem, North Carolina, 27103 USA, is the contract manufacturer of the investigational product, REACT.
- Labcorp (*https://www.Labcorp.com/services/clinical-testing/central-laboratory-services.html*) will provide central laboratory services for the study.
- PPD Inc. (3900 Paramount Pkwy, Morrisville, North Carolina 27560, USA) will provide study management (e.g., CTMS, site monitoring and site payments), data management (e.g., interactive voice response systems [IVRS] / interactive web randomization system [IWRS], electronic data capture database), and medical monitoring, biostatistics, and medical writing services for the study.
- IQVIA RDS Inc., 4820 Emperor Boulevard, Durham, North Carolina, 27703, USA, will provide pharmacovigilance management.

4.2 Overall Study Design

This is a multi-center, prospective, open-label study in which eligible subjects will be randomized (1:1) to one of two treatment groups (Cohort 1 or Cohort 2) once eligibility is determined.

Subjects randomized to Cohort 1 will receive their first REACT injection soon after the REACT product is manufactured and shipped to the clinical site. After 3 months (+60 days), a second injection will be given in the non-biopsied contralateral kidney (i.e., the kidney that was not injected with the first injection). Subjects will be followed every 3 months for at least 18 months after the last REACT injection.

Subjects randomized to Cohort 2 will receive their first REACT injection as soon as the REACT product is manufactured and shipped to the clinical site and will then undergo an observation period to see whether a redose trigger is met. If one of the following redose triggers is met, they will receive their second REACT injection no sooner than 3 months following the first REACT injection and then will be followed every 3 months for at least 18 months after the last REACT

injection. The redose triggers will be evaluated beginning at the month 3 post first REACT injection visit through month 15 post first REACT injection visit. The second injection will be given in the non-biopsied contralateral kidney (i.e., the kidney that was not injected with the first injection). If a redose trigger is not met within 15 months after the first REACT injection, subjects will complete their participation in this study at Month 18 EOS visit having received only 1 REACT injection.

Redose triggers for Cohort 2 are defined below:

1. 30-day sustained decline in eGFR by at least 20% from the baseline value confirmed with repeat laboratory testing and/or
2. increase of greater than or equal to 30% and of at least 30 mg/g in UACR from Baseline, using a standard urine chemistry, sustained for at least 30 days with two repeat central laboratory testing 7 days apart at least 30 days after initial event for confirmation.

A schematic of the study design is presented in Figure 1.

Figure 1: Schematic of Study Design

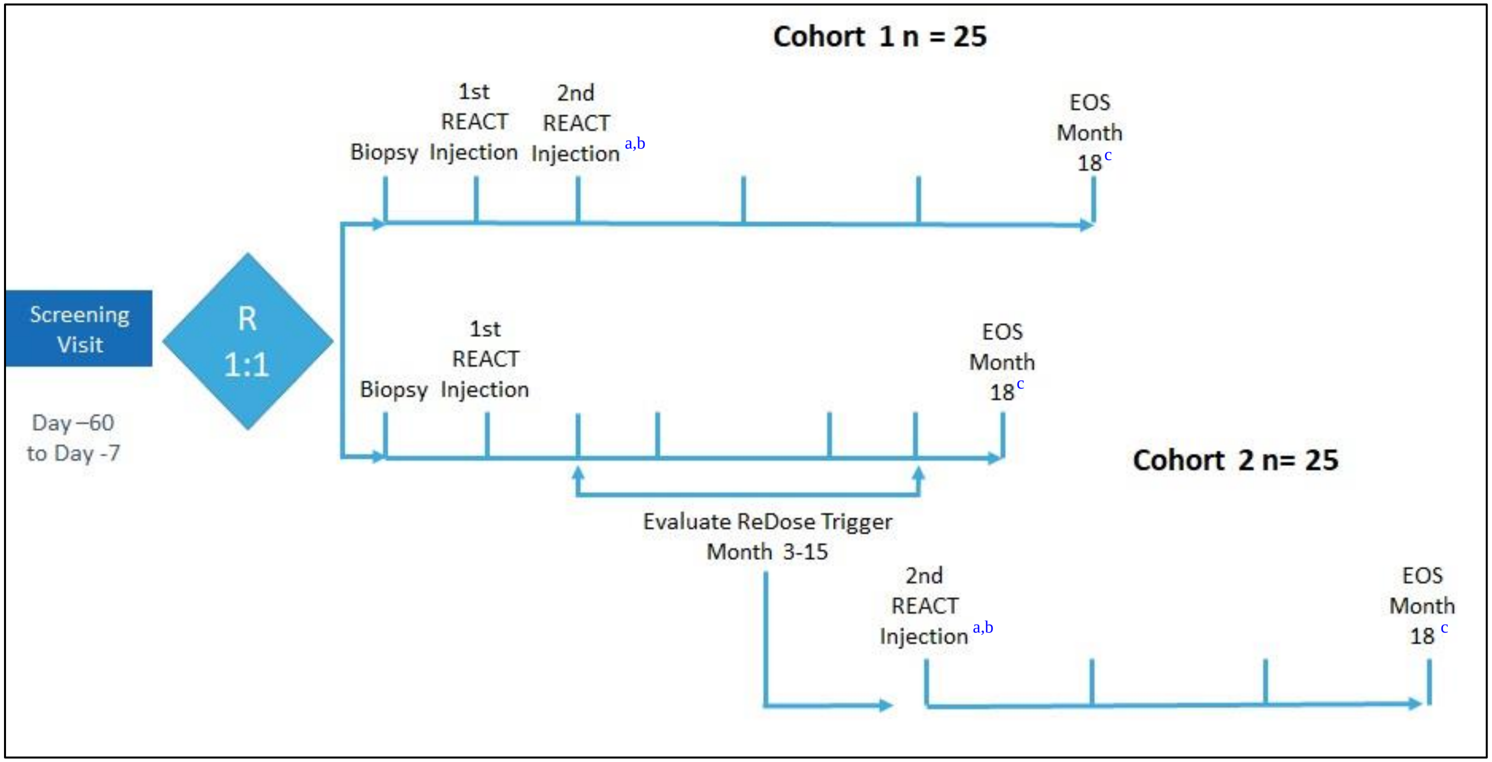

Abbreviations: eGFR = estimated glomerular filtration rate; EOS = End-of-Study Visit; F/U = follow-up; REACT = renal autologous cell therapy.

a. The second REACT injection will be performed in the contralateral kidney (i.e., the kidney not injected with the first REACT injection).
b. The second REACT injection will take place no sooner than 3 months after the first REACT injection.
c. All subjects will be followed for at least 18 months after their last REACT injection.

4.3 Number of Subjects

Approximately 50 subjects who satisfy all inclusion and exclusion criteria and complete the screening procedures will be enrolled and randomized to 1 of 2 cohorts in a 1:1 ratio (25 subjects per cohort) to receive two scheduled REACT injections (Cohort 1) or one REACT injection followed by a triggered redose REACT injection (Cohort 2).

For details regarding the assumptions used to determine the sample size, see Section 9.

4.4 Treatment Assignment

After subject eligibility has been confirmed, the Investigator will randomize the subject to Cohort 1 (up to 25 subjects) or Cohort 2 (up to 25 subjects) via an interactive web randomization system (IWRS). Randomization will occur at a 1:1 ratio with equal distribution between the cohorts. Since this is an open-label study; neither the study subject, Investigators, site staff, nor Sponsor will be blinded to the treatment assignment.

4.5 Treatment Compliance

Subjects in Cohort 1 will receive two REACT injections into biopsied and non-biopsied contralateral kidneys 3 months (+60 days) apart. Subjects in Cohort 2 will receive a first REACT injection followed by a second REACT injection in the non-biopsied contralateral kidney if they meet renal function criteria for redosing. The second REACT injection trigger will be evaluated beginning at the month 3 post first REACT injection visit through Month 15 post first REACT injection visit. The investigational product will be administered percutaneously into the kidneys. REACT product preparation and dosing procedures are specified in this protocol as well as the REGEN-007 Proceduralist Training Manual.

In some cases, a subject or the Investigator may elect to delay or withhold the second REACT injection. For example, if there appears to be any untoward safety risk, or if the subject's health status would, in the judgment of the Investigator, be jeopardized, then a REACT injection should not be administered.

However, if a subject does not receive a second REACT injection they must complete all visits per protocol except visits Day 1, Day 7, and Day 14 post second REACT injection. On Second REACT Injection Day subjects will still undergo the following assessments: record con meds, vital signs, laboratory tests, and record AE's. Subjects will not receive ultrasound, CT, or undergo the injection procedure.

4.6 Study Duration

In this study, eligible subjects will be identified during a screening period of up to 60 days. The duration of treatment for each of the 2 cohorts is described below.

4.6.1 Cohort 1

Subjects in Cohort 1 will undergo screening (up to 60 days) followed by the Biopsy Visit. Assuming 12 weeks (+30 days) from biopsy to first REACT injection with a 3-month (+60 days) period between both REACT injections, the total study duration (including the screening period) will be up to 29 months. All subjects will be followed for at least 18 months after the last REACT injection.

4.6.2 Cohort 2

Subjects in Cohort 2 will undergo screening (up to 60 days) followed by the Biopsy Visit. Assuming 12 weeks (+30 days) from biopsy to first REACT injection. Subjects will be evaluated for a redose trigger starting at Month 3 post first REACT injection through Month 15 post first REACT injection. If no trigger is met within that time frame, they will undergo an EOS visit at Month 18 post first REACT injection. If the subject meets a trigger within the 3 month-15-month post first REACT injection timeframe they will receive their second REACT injection and be followed for an additional 18 months. Total study duration ranges from 29 to 42 months. All subjects will be followed for at least 18 months after the last REACT injection.

4.6.2.1 Criteria for Redosing a Second REACT Injection in Subjects in Cohort 2

Subjects meeting criteria for redosing in Cohort 2 will receive a second REACT injection no sooner than 3 months following the first REACT injection. Criteria for redosing will be assessed beginning at month 3 visit after the first injection up to 15 months after the first injection and are based on renal function as described below:

1. 30-day sustained decline in eGFR by at least 20% from the baseline value confirmed with repeat laboratory testing and/or
2. increase of greater than or equal to 30% and of at least 30 mg/g in UACR from Baseline, using a standard urine chemistry, sustained for at least 30 days with two repeat central laboratory testing 7 days apart at least 30 days after initial event for confirmation.

The 30-day sustained decline in eGFR will be evaluated with the 2009 CKD-EPI serum creatinine equation performed and analyzed at the central laboratory. To reach this criteria for a second REACT injection, two consecutive eGFR measurements at 30 days (+7 days) must be

greater than or equal to 20% below the baseline eGFR value. The baseline eGFR value will be the last measurement taken prior to first REACT injection. Refer to Section 2 for additional details on the method used to calculate this greater than or equal to 20% decline in eGFR from the baseline value. Once an eGFR value is greater than or equal to 20% below the baseline value, a second eGFR measurement will be repeated 30 days (+7 days) after the first eGFR measurement. If this second value is also greater than or equal to 20% below the baseline value, then the subject will have met this criterion for a second REACT injection.

The 30-day increase in UACR of greater than or equal to 30% from Baseline will be evaluated using urinalyses performed at the central laboratory. To reach this criterion for a second REACT injection, two consecutive UACR measurements taken 7 days apart must be at least 30% higher or more than the baseline UACR after 30 days with an absolute value of at least 30 mg/g. The baseline UACR value will be the last measurement prior to first REACT injection. Refer to Section 2 for additional details on the method used to calculate this increase of UACR from the baseline value. Once an UACR is greater than or equal to 30% the baseline value and is greater than or equal to 30 mg/g, a second UACR measurement will be repeated at least 30 days after the initial event and confirmed with two UACR measurements taken 7 days apart. If these confirmatory values are also greater than or equal to 30% the baseline value and is greater than or equal to 30 mg/g, then the subject will have met this criterion for a second REACT injection.

Before proceeding with redosing, the site and CRO will work to produce a package including medical records, AE/SAEs, and laboratory reports for an independent redose adjudication committee to review and confirm the redose trigger event. The Sponsor, Medical Monitor, and independent redose adjudication committee will confirm that the subject has met one of the criteria for the second REACT injection and rule out other potential causes of acute decline in eGFR, such as anatomical, infectious, or drug-related issues. In the event that the Sponsor, Medical Monitor, and/ or independent redose adjudication committee identify particular causes of the decline in eGFR, additional testing (eGFR or otherwise) may be requested to confirm that the subject meets criteria for a second REACT injection (see Section 6.4).

The second REACT injection should be given within 30 days of confirmation of meeting trigger criteria sustainment for injection. If a subject does not receive the second REACT injection within 60 days of meeting renal function trigger for the second injection, the subject will not undergo the second injection and will remain on his/her original follow-up schedule of assessments.

4.7 Prohibited and Concomitant Medications

- The consumption of NSAIDs, including aspirin, clopidogrel, prasugrel, dipyridamole, and other platelet aggregation inhibitors is prohibited during the study beginning 7 days before through 7 days following the percutaneous renal biopsy and REACT injection(s).
- Aspirin, at a dose of up to 100 mg/day, may be continued if doing so is the standard of care at the investigational site, is accepted for primary prevention of heart disease in subjects with diabetes who are older than 40 years of age or have additional risk factors for cardiovascular disease or stroke, and for whom the perceived benefits of aspirin therapy outweigh the risks associated with treatment.
- Intake of fish oil is prohibited during the study beginning 7 days before through 7 days following the percutaneous renal biopsy and REACT injection(s).
- Subjects who are undergoing treatment with an ACE or an ARB must have stable maximum tolerated daily dose initiated at least 4 weeks prior to randomization.
- For subjects who have a bleeding disorder(s) or are maintained on any anticoagulation medications such as warfarin, LMWH, enoxaparin, anti-platelet medications (aspirin, NSAIDs, clopidogrel, Persantine), factor Xa inhibitors and other novel oral NOAC, including other herbal, over the counter products (fish oils, omega 3 medications) or other supplementary products; anticoagulant treatment are to be discontinued for 7 days prior and 7 days following biopsy or REACT injections. ProKidney does not endorse anticoagulation bridging protocols with cardiac stents.
- Medications that interfere with measurements of sCr should be avoided during the study, such as trimethoprim, dronedarone, and cimetidine. If such medications are required based on medical necessity, then the circumstance should be discussed with the Medical Monitor and documented within the Case Report Form (CRF).
- Use of investigational drugs is prohibited during the course of the study, unless preapproved by the Medical Monitor. Investigational drugs are defined as drugs that have not been approved for use by the FDA or other regulatory authorities.
- Subjects must not receive a vaccine of any kind within a minimum of 30 and a maximum of 90 days before or after Biopsy and/or REACT injections depending on a consult between Investigator and Medical Monitor for evaluation of adverse effects.

4.8 Subject Withdrawal Criteria

Subjects may withdraw from the study at any time and for any reason without penalty or prejudice, and without jeopardizing access to future medical care. If the Investigator determines that continuing in the study is no longer in the best interest of the subject, then the Investigator

should withdraw the subject from the study. If a subject voluntarily withdraws from the study, the reason(s) should be documented. Subjects who withdraw from the study or are withdrawn by the Investigator following randomization should undergo procedures specified at the EOS Visit.

- Subjects who withdraw from the study before randomization will be considered screen failures and will be replaced.
- If a subject withdraws from the study after randomization, but before having the renal biopsy, the subject will be replaced.
- If a subject withdraws from the study following the renal biopsy but before the first REACT injection, the subject may be replaced.
- If a subject withdraws from the study after REACT injection but before the end of the follow-up period, the subject cannot be replaced.

Every effort should be made to ensure that subjects who have received a REACT injection return for all subsequent follow-up visits and procedures, including the EOS Visit assessments.

5 STUDY POPULATION

5.1 Inclusion Criteria

Unless otherwise noted, subjects must satisfy each inclusion criterion to participate in the study. Inclusion criteria will be assessed at the Screening Visit.

1. Male or female aged 30 to 80 years, inclusive, on the date informed consent is signed.
2. Clinical diagnosis of T1DM or T2DM, controlled per institutional standard of care.
3. The subject has a clinical diagnosis of diabetic nephropathy as the underlying cause of renal disease (diagnosis does not have to be confirmed via renal biopsy).
4. The subject has a serum glycosylated hemoglobin (HbA_{1c}) level less than 10% at the Screening Visit.
5. The subject has a documented clinical diagnosis of an eGFR between 20 and 50 mL/min/1.73m^2 inclusive not requiring renal dialysis.
6. The subject has a urinary albumin-to-creatinine ratio (UACR) of greater than or equal to 30 and less than or equal to 5,000 mg/g.
7. The subject has stable blood pressure and is maintained on a stable anti-hypertensive medication regimen, if treatment for hypertension is necessary. If treatment includes an angiotensin-converting enzyme inhibitor (ACEi) or an angiotensin receptor blocker (ARB), that treatment must be the maximum tolerated daily dose for at least 4 weeks prior to randomization or, if the treatment includes a sodium glucose cotransporter 2 inhibitor (SGLT2i) at any dose, for at least 4 weeks prior to randomization.

Note: A maximum tolerated daily dose of an ACEi or ARB is defined as the maximum tolerated dose for diabetic nephropathy (for agents with an approved indication for diabetic nephropathy in patients with T1DM or T2DM, i.e., losartan and irbesartan) or the maximum tolerated dose for hypertension (for agents without an approved indication for diabetic nephropathy), unless side effects or adverse events (AE) limit the use of the maximum dose. For subjects who are not on a maximum daily dose of an ACEi or ARB, Investigators will be required to document why a higher dose is contraindicated.

8. A minimum of 3 measurements of eGFR (by sCr or cystatin C) must be obtained at least 3 months apart within 24 months prior to the Screening Visit to define the rate of progression of CKD.
9. The subject agrees and is able to refrain from nonsteroidal anti-inflammatory drugs (NSAIDs), including aspirin, clopidogrel, prasugrel, dipyridamole, and other platelet aggregation inhibitors during the period beginning 7 days before through 7 days following the percutaneous renal biopsy and REACT injection(s).
   Note: Aspirin, at a dose of up to 100 mg/day, may be continued if doing so is the standard of care at the investigational site, is accepted for primary prevention of heart disease in subjects with diabetes who are older than 40 years of age or have additional risk factors for cardiovascular disease or stroke, and for whom the perceived benefits of aspirin therapy outweigh the risks associated with treatment.
10. The subject agrees and is able to refrain from oral ingestion of fish oil supplements during the period beginning 7 days before through 7 days following the percutaneous renal biopsy and REACT injection(s).
11. The subject is willing and able to cooperate with all aspects of the protocol.
12. The subject is willing and able to provide signed informed consent.

5.2 Exclusion Criteria

Subjects who satisfy any exclusion criterion listed below are not eligible to participate in the study. Exclusion criteria will be assessed at the Screening Visit.

1. The subject has a history of renal transplantation.
2. The subject has a mean systolic blood pressure greater than or equal to 140 mmHg and/or mean diastolic blood pressure greater than or equal to 90 mmHg at screening. Subjects with blood pressure outside of this range prior to biopsy/injection may continue if approved by the Medical Monitor.
3. The subject has hemoglobin levels less than 10 g/dL and is not responsive to the standard medical intervention for CKD-related anemia prior to randomization.

4. The subject appears to be at possibly increased risk of either thromboembolism or bleeding because of abnormal results at the Screening Visit for any of the following tests: activated partial thromboplastin time (APTT), prothrombin time-international normalized ratio (PT-INR), and platelet count.
5. The subject has a bleeding disorder(s) or is maintained on any anticoagulant agents, including fractionated heparin preparations, Coumadin® (warfarin), or direct thrombin inhibitors, that cannot be discontinued for 7 days before and 7 days after biopsy or injections.
6. The subject has a known allergy or contraindication(s), has experienced severe systemic reaction(s) to kanamycin/structurally similar aminoglycoside antibiotic(s), which may be a manufacturing process residual, or has a known hypersensitivity to dimethyl sulfoxide.
7. The subject has a history of anaphylactic or severe systemic reaction(s) or contraindication(s) to human blood products, Dextran-40, or materials of animal origin (e.g., bovine).
8. The subject is not a good candidate to undergo percutaneous REACT injection, in the judgment of the interventionalist or proceduralist who will perform the procedure. This includes confirming the subject has contraindications for undergoing the procedure based on depth of the kidneys, positioning limitations, and if the kidneys are greater than 15 cm from skin surface to kidney capsule, or has only one kidney.
9. The subject has a history of severe systemic reaction(s) or any contraindication to local anesthetics or sedatives.
10. The subject has a history of complicated recurrent urinary tract infections or renal stone disease.
11. The subject has been diagnosed with acute kidney injury within 3 months of the Screening Visit.
12. The subject has any of the following conditions: autosomal dominant and recessive polycystic kidney disease, focal segmental glomerulosclerosis, vasculitis-related CKD, IgA or IgG nephropathy, drug-induced or hypertension-induced CKD and other types of CKD as determined by the Investigator that would interfere with biopsy and REACT injection procedure (such as horseshoe kidney variant and unexplained hydronephrosis), any other documented renal pathology that would interfere with the REACT injection procedure.

    Note: Anatomic abnormalities and benign conditions are not exclusionary if the kidney remains accessible and meets the criteria to receive the REACT injection.

13. The subject has poor diabetes control as evaluated by the Investigator, including, but not limited to, a history of diabetic ketoacidosis (DKA) in the year prior to screening, frequent hypoglycemic episodes, or hypoglycemia unawareness.
14. The subject is awaiting a pancreas transplant.
15. The subject has incapacitating cardiac neurologic, peripheral vascular, or pulmonary disorders as determined by the Principal Investigator.
16. The subject has a history of malignancy within the past 3 years (exceptions: squamous and basal cell carcinomas of the skin and carcinoma of the cervix in situ, or a malignancy that in the opinion of the Investigator, along with the Medical Monitor, is considered treated with minimal risk of recurrence).
17. The subject has documented clinical diagnosis of chronic hepatic disease (alanine aminotransferase [ALT] or aspartate aminotransferase [AST] greater than 3 times the upper limit of normal) at the Screening Visit.
18. The subject has a positive test result for the hepatitis B virus (HBV) surface antigen, a positive test result for hepatitis C virus (HCV) antibodies, or a positive test result for human immunodeficiency virus (HIV) antibodies.
    Note: At the discretion of the Investigator, a subject who gives a history of a treated and cured HCV infection may be screened with a test for viral ribonucleic acid (RNA) and, if a cure is demonstrated, the subject may be enrolled.
19. The subject has a documented clinical diagnosis of active tuberculosis (TB) requiring treatment.
20. The subject is immunocompromised or is receiving immunosuppressive agents, including individuals treated for chronic glomerulonephritis within 3 months of the Screening Visit.
    Note: Inhaled corticosteroids, chronic low-dose corticosteroids (less than or equal to 7.5 mg prednisone equivalent per day), and brief pulsed corticosteroids for intermittent symptoms (e.g., asthma) are permitted.
21. The female subject is pregnant, lactating (breast feeding), or planning a pregnancy during the course of the study. Or the female subject is of childbearing potential and is not using a highly effective method(s) of birth control, including sexual abstinence. Or the female subject is unwilling to continue using a highly effective method of birth control throughout the duration of the study.
    Note: A highly effective method of birth control is defined as one that results in a low failure rate (i.e., less than one percent per year) when used consistently and correctly, such as implants, injectables, combined oral contraceptives, some intrauterine devices, sexual abstinence, or a vasectomized partner.

22. The subject has a history of active alcohol and/or drug abuse that, in the judgment of the Investigator, would impair the subject's ability to comply with the protocol.
23. The subject's health status would, in the judgment of the Investigator, be jeopardized by participating in the study.
24. The subject has used an investigational product within 3 months prior to the Screening Visit without receiving written consent from the study assigned Medical Monitor.
25. The subject has previously received treatment with REACT.

6 STUDY VISITS

The schedules of clinical assessments and procedures to be performed during the study are displayed in the Time and Events tables (Table 1, Table 3, and Table 5). Similarly, the schedules of sample collection and clinical laboratory evaluations planned for the study are displayed in the Laboratory Time and Events tables (Table 2, Table 4, and Table 6). Before conducting any study-specific assessments or procedures (including screening), the subject must provide written informed consent in accordance with the International Conference on Harmonization (ICH)-GCP guidelines and 21 Code of Federal Regulations (CFR) Part 50.

Due to the coronavirus disease (COVID-19) pandemic that began in March 2020, subject follow-up visits may be conducted remotely or via home health services to ensure the safety of the subject. Any procedures listed in the schedule of events that are not completed due to COVID-19 pandemic restrictions will be documented as protocol deviations. Clinical laboratory evaluations are permitted to be completed by a local laboratory convenient for the subject but will not be collected by the Sponsor for inclusion in efficacy analyses. If a clinical laboratory measure obtained at a local laboratory result would be considered a "trigger" it will be repeated at the central laboratory. If central laboratory result confirms the trigger has been met then the central laboratory result will be used in the analysis.

6.1 Screening

Subjects who satisfy the eligibility criteria and provide written informed consent may be enrolled into the study. The subject should have adequate, historical clinical data to provide a reasonable estimate of the rate of progression of CKD (see Section 5.1, Inclusion Criterion 8). Screening procedures include a full physical examination, measurement of vital signs, electrocardiogram (ECG), and laboratory assessments (hematology, clinical chemistry, serology, coagulation, urine chemistry, urine pregnancy test for females of childbearing potential, samples for research, and

additional tests). For details of all assessments performed at the Screening Visit for Cohort 1 see Table 1 and for Cohort 2, see Table 3.

All screening assessments should take place in a timeframe that will allow for scheduling of the renal biopsy within 60 days (+14 days) of the subject signing the Informed Consent Form.
An MRI study without contrast must be performed at the Screening Visit to determine kidney size and volume. If a subject cannot undergo an MRI, then CT can be substituted to obtain kidney size and volume.

To qualify for study enrollment, the subject's eGFR must be between 20 and 50 mL/min/1.73 m^2 inclusive, at the Screening Visit determined by the central laboratory using the 2009 CKD-EPI serum creatinine equation. [80]

If a subject does not meet a specific eligibility criterion, but the Investigator believes that the subject would be an excellent candidate for the clinical study, then that criterion may be reassessed one time during the screening period. In general, a subject who does not qualify during the 60-day screening window and is considered a screen fail may be rescreened once as long as the Investigator has sufficient clinical justification. If the Investigator has any questions concerning the appropriateness of rescreening a subject, then the Investigator should contact the Medical Monitor.

If, for whatever reason, the biopsy cannot be conducted within 60 days (+14 days) of the Screening Visit, then the Investigator and Medical Monitor should discuss and agree upon the need for repeating the screening assessments on a case-by-case basis. For example, laboratory assessments performed between 1 and 7 days before the renal biopsy might be used in place of the screening assessments to satisfy final eligibility criteria. In other cases, it may not be appropriate or necessary to repeat the clinical diagnostic procedures, including ECG or MRI studies.

6.2 Randomization

After successful screening, the site will be able to use the IWRS system to randomize the subject (1:1) into one of two cohorts of 25 subjects each: Cohort 1 (two scheduled REACT injections) and Cohort 2 (one REACT injection followed by a possible second REACT injection to be administered based on renal function trigger).

As this is an open-label study, neither the study subject, Investigators, site staff, or Sponsor will be blinded to the treatment assignment.

6.3 Biopsy

The biopsy should be scheduled within 60 days (+14 days) of signing the Informed Consent Form for all subjects.

Subjects must not receive a vaccine of any kind within a minimum of 30 and a maximum of 90 days before or after biopsy procedure depending on a consult between Investigator and Medical Monitor for evaluation of adverse effects.

Subjects will report to the site 1 to 7 days before undergoing a renal biopsy for pre-biopsy assessments, including an interim physical examination, measurement of vital signs, ECG, and renal MRI (if not completed during or after the Screening Visit). Subjects will undergo an ECG at the Screening Visit and EOS visit. For details of all assessments performed at the Day -7 to Day -1 visit, see Table 1 for Cohort 1 and Table 3 for Cohort 2.

Laboratory tests, including hematology, clinical chemistry, coagulation, urine chemistry, urine pregnancy test for females of childbearing potential, samples for research, and additional tests, will also be performed at the Day -7 to Day -1 visit. Serology tests are performed during the Screening Visit (Day -60 to -7). For details of all laboratory assessments performed at the Day -7 to Day -1 visit, see Table 2 for Cohort , Table 4 and Table 6 for Cohort 2.

Central laboratory assessments should be collected pre and post biopsy procedure along with local laboratory assessments pre and post biopsy procedure according to Table 2, Table 4, and Table 6.

If a subject is tested for COVID-19 and is found positive, biopsies and injections will be postponed for a minimum of 30 days and a maximum of 90 days from onset of symptoms/diagnosis. Biopsies and injections will be resumed only after the subject is cleared by the Investigator and Medical Monitor for proceeding with procedures.

After admission to the site on the Biopsy Visit (Day 0), the biopsy should be performed as described in Section 3.2.1.

A minimum of two biopsy cores, each measuring 1.5 cm in length, collected using a 16-gauge needle (or four biopsy cores, each measuring 1.5 cm in length, collected using an 18-gauge needle) under sterile conditions from each enrolled subject should be sent to ProKidney using the shipping container provided by the Sponsor and according to procedures detailed in the Proceduralist Training Manual. If the biopsy cannot be used to manufacture REACT, or if the subject decides not to have a biopsy, the subject should be discontinued from the study.

A safety ultrasound will be performed following the renal biopsy on Day 0 and Day 1 with the aim of monitoring possible subclinical AEs (e.g., swelling, renal hematoma, fluid accumulation). Subjects who do not experience complications from the biopsy may be discharged the same day consistent with site standard practice. Otherwise, the subject should remain in the hospital overnight for observation. The subject may be discharged on the day after the biopsy after an observation period of 6 hours as long as any biopsy-related AEs have resolved, stabilized, or returned to baseline.

The day following the Biopsy Visit, subjects will return to the clinic for a follow-up evaluation. During the Day 1 Follow-Up Visit, concomitant medications as well as AEs will be recorded and laboratory assessments will be performed per the Time and Events tables.

6.4 REACT Injection

It is planned that subjects in Cohort 1 will receive two REACT injections 3 months (+60 days) apart and subjects in Cohort 2 will receive at least one, but possibly up to two REACT injections at least 3 months apart based on whether they reach the trigger to redose based on renal function. The first injection for Cohort 1 and Cohort 2 should be scheduled 12 weeks (+30 days) following biopsy. On Day 0 (REACT Injection), subjects will report to the site and undergo assessments prior to autologous REACT injection. Subjects will be injected with autologous REACT using a percutaneous approach as discussed in Section 3.2.4. If a subject is tested for COVID-19 and is found positive, biopsies and injections will be postponed for a minimum of 30 days and a maximum of 90 days from onset of symptoms/diagnosis. Biopsies and injections will be resumed after the subject is cleared by the Investigator and Medical Monitor for proceeding with procedures.

Subjects must not receive a vaccine of any kind within a minimum of 30 and a maximum of 90 days before or after biopsy procedure depending on a consult between Investigator and Medical Monitor for evaluation of adverse effects.

For subjects randomized to Cohort 1, **every attempt should be made to ensure that the second REACT injection is administered 3 months (+60 days) after the first injection**. In the event that a subject in Cohort 1 cannot schedule his/her second REACT injection 3 months (+60 days) after receiving the first injection or cannot keep his/her scheduled 3-month visit, the Sponsor and the Medical Monitor must be notified immediately. The expectation is that the subject and the site will accommodate the Sponsor's preference that the series of two REACT injections should be administered no more than 3 months (+60 days) apart. The second injection will be given in the contralateral kidney (i.e., the kidney that was not injected with the first injection). In some cases, a subject or the Investigator may decide to postpone or withhold the second REACT injection. For example, if there appears to be any untoward safety risk, or rapid deterioration of renal function, development of uncontrolled diabetes, uncontrolled hypertension, or development of a malignancy or an intercurrent infection, then the second REACT injection should not be administered. If a subject does not receive a second REACT injection they must complete all visits per protocol except visits Day 1, Day 7, and Day 14 post second REACT injection. On Second REACT Injection Day subjects who will not receive a second REACT injection will still undergo the following assessments: record con meds, vital signs, laboratory tests, and record AE's. These subjects will not receive ultrasound, CT, or undergo the injection procedure on Second REACT Injection Day.

For subjects in Cohort 2, subjects meeting redose triggers based on renal function will receive their second REACT injection no sooner than 3 months following the first REACT injection and then followed every 3 months for at least 18 months after the last REACT injection. The second injection will be given in the contralateral kidney (i.e., the kidney that was not injected with the first injection) approximately 30 days after the subject had met the trigger including sustainment for redosing. If a redose trigger is not met between 3 - 15 months after the first REACT injection, subjects will complete their participation in this trial only having received one REACT injection. In the event that the subject is unable to receive his/her second REACT injection within 60 days of meeting trigger criteria, then he/she must continue follow-up his/her regular follow-up visits and will not receive a second REACT injection.

Vital signs will be monitored during the REACT injection procedure according to Section 7.2.1.1.

6.5 Discharge After REACT Injection

Following the REACT injection(s), safety ultrasounds will be performed on Day 0, Day 1, Day 7, and optional Day 14 with the aim of monitoring possible subclinical AEs (e.g., swelling,

hematoma, fluid accumulation). If there is a hematoma greater than 2 cm present on Day 0,Day 1, or Day 7 for REACT injections, then an ultrasound should be conducted at Day 14. If product or procedure-related AEs occur following REACT injection, the subject should not be discharged until the AEs have resolved, stabilized, or returned to baseline. If consistent with the site's standard practice, the subject may be discharged the same day as the REACT injection after no less than 4 hours of observation and monitoring.

6.6 Follow-up Visits

The follow-up visit schedules for Cohorts 1 and 2 are described below. If a subject is unable to attend in-person visits based on hospital policies (for instance due to COVID-19 positivity or signs, symptoms of or a contact with a positive COVID-19 person), follow-up may be conducted using a telephone call, remote or home visits, as appropriate.

6.6.1 Cohort 1

Following administration of the first REACT injection on Day 0, subjects will return to the site for follow-up visits on Days 1, 7, 14 (±7 days), 28 (±7 days), and Month 3 (±10 days). There will be an interval of 3 months (+60 days) between the first and second REACT injections. After administration of the second REACT injection, subjects will return to the site for follow-up visits on Days 1, 7, 14 (±7 days), 28 (±7 days), and Month 3 (±10 days). Long-term follow-up visits will continue at 3-month intervals (±10 days) starting at Month 3 until Month 18 after the last REACT injection. For details of the assessments performed at each study visit for subjects in Cohort 1, see Table 1 and Table 2. Every attempt should be made to ensure that the second REACT injection is administered 3 months after the first injection.

If a subject does not receive a second REACT injection, they must complete all visits per protocol except visits Day 1, Day 7, and Day 14 post second REACT injection. On Second REACT injection Day subjects will still undergo the following assessments: record con meds, vital signs, laboratory tests, and record AE's. Subjects will not receive ultrasound, CT, or undergo the injection procedure.

6.6.2 Cohort 2 Subjects Not Meeting Trigger for Redosing by Month 15

Subjects will return to the site following the first REACT injection on Day 0 for follow-up visits on Days 1, 7, 14 (±7 days), 28 (±7 days), and Month 3 (±10 days) and every 3 months (±10 days) thereafter until Month 18 (±10 days). Subjects not meeting the trigger for redosing by Month 15 will also follow this schedule of follow-up visits (i.e., subjects not meeting criteria for redosing

on or before Month 15 will not receive a second REACT injection and will continue follow-up visits until Month 18. For details of the assessments performed at each study visit for subjects in Cohort 2 not meeting criteria for redosing, see Table 3, and subjects meeting redose criteria see Table 4.

In the event a subject meets criteria for redosing up to and including the Month 15 visit, a second REACT injection will be planned, and the schedule of follow-up visits will occur per Table 4.

6.6.3 Cohort 2 Subjects Meeting Trigger for Redosing Prior to Month 15

Subjects meeting criteria for redosing beginning at Month 3 up to and including Month 15 following the first REACT injection will undergo confirmatory laboratory testing per Section 4.6.2.1. Once criteria for redosing have been verified by ProKidney, the Medical Monitor, and independent event reviewer, CRO will notify the site that they can proceed with scheduling second REACT injection. Sites should schedule the second REACT injection approximately 30 days following confirmation of the subject meeting redosing trigger.

After administration of the second REACT injection, subjects will return to the site for follow-up visits on Days 1, 7, 14 (±7 days), 28 (±7 days), and Month 3 (±10 days). Long-term follow-up visits will continue at 3-month intervals (±10 days) starting at Month 3 until Month 18 after the last REACT injection. For details of the assessments performed at each study visit for subjects in Cohort 2 meeting criteria for redosing, see Table 5 and Table 6.

If a subject does not receive the second REACT injection within 60 days of meeting renal function trigger for the second injection, the subject will not undergo the second injection and will remain on his/her original follow-up schedule of assessments.

6.6.4 End-of-Study Visit

This section describes situations in which a subject will undergo the EOS Visit, for example, due to premature discontinuation from the study or completion of all protocol-specified follow-up visits.

- If a subject discontinues from the study after undergoing randomization but before biopsy, then that subject should complete all EOS assessments except for the MRI study. These imaging studies should only be completed if, in the judgment of the Investigator, one and/or the other would provide critical safety information needed for subject care. If the subject is experiencing an investigational product- or study procedure-related SAE, then

the subject will not be discontinued until the SAE has resolved, stabilized, or returned to baseline.

- If a subject discontinues from the study after biopsy but before REACT injection then that subject should complete all EOS assessments except for the MRI study. These imaging studies should only be completed if, in the judgment of the Investigator, one and/or the other would provide critical safety information needed for subject care. If the subject is experiencing an investigational product- or study procedure related SAE, then the subject will not be discontinued until the SAE has resolved, stabilized, or returned to baseline.
- If a subject discontinues from the study after undergoing one or two REACT injections but before completing all of the protocol-specified follow-up visits, then he/she should have the EOS Visit at the time of discontinuation. For these subjects, the Investigator will determine whether or not it is clinically prudent to perform the MRI study. If the subject is experiencing an investigational product- or study procedure-related SAE, then the subject will not be discontinued until the SAE has resolved, stabilized, or returned to baseline.
- If a subject completes all of the protocol-specified follow-up visits, he/she will undergo all EOS assessments at least 18 months after the last REACT injection. If the subject is experiencing an investigational product- or study procedure-related SAE, then the subject will not be discontinued until the SAE has resolved, stabilized, or returned to baseline.

6.6.5 Study Completion

Completion of the study is defined as the time when the last subject completes the EOS Visit, or when the last subject is considered lost to follow-up, withdraws consent, or dies.

7 STUDY ASSESSMENTS AND PROCEDURES

7.1 Demography and Medical History

Demographic characteristics will be obtained for each subject at the Screening Visit.

All CKD-related medical history and all other significant medical history will be recorded in the CRF beginning at the Screening Visit. Throughout the study, medical conditions that are still ongoing will be regularly updated in the CRF.

7.2 Clinical Evaluations

7.2.1 Vital Signs

Vital signs to be measured include systolic/diastolic blood pressure, heart rate, respiration rate, and temperature. Blood pressure will be measured after the subject has been seated for a minimum of 5 minutes. At the Screening Visit (Day -60 to Day -7), three blood pressure measurements will be taken and the average of the three measurements (for systolic and diastolic pressure independently) will be used to satisfy entry criteria in the study and will be documented into the CRF.

7.2.1.1 Vital Signs During the REACT Injection

Vital signs, including heart rate, blood pressure, oxygen saturation, and respiration rate, will be measured at regular intervals throughout the REACT injection procedure. These values must be entered into EDC at a minimum of 5-minute intervals during the procedure. Temperature does not need to be recorded throughout the procedure but should be documented once.

7.2.2 Physical Examination

The Investigator will perform the physical examination (PE). As outlined in the Study Reference Manual, the comprehensive examination will assess all pertinent body systems. The interim PE must include at least the following components: general appearance, chest/respiratory, cardiovascular, abdominal, and neurologic. If clinically indicated, a full PE may be performed. The subject’s weight and body mass index (BMI) will be measured at every visit that includes a comprehensive or interim PE. The subject’s height will be taken at the Screening Visit. The BMI will be calculated as kg/m^2. Only clinically significant abnormalities will be recorded in the CRF.

7.2.3 ECG

A 12-lead ECG will be obtained after the subject has been resting on their back for 5 minutes with the blood pressure cuff applied but not inflated at the level of the heart. The ECG recordings will be assessed by the Investigator and the results entered into the CRF. The ECGs will be performed during screening and at the EOS Visit. Electrocardiographic changes will be recorded if clinically significant as determined by the Investigator and the ECG electronic interpretation included in the record.

7.2.4 Concomitant Medications

Concomitant medications will be recorded in the CRF at each study visit, including any CKD-specific medications as well as medications that may affect renal hemodynamics and/or sCr measurements. In addition, any medications used to treat an AE that is documented in the CRF will be recorded. Surgical medications used during the biopsy procedure or the REACT injection procedure do not need to be captured in the CRF unless their use falls outside of expected dosages and/or frequencies of administration.

7.2.5 Laboratory Assessments

Planned clinical laboratory evaluations are listed in Table 13. All analyses will be conducted by a central laboratory, except as noted. The schedules for collecting biological samples during the study are shown in the Laboratory Time and Events tables in Table 2, Table 4, and Table 6.

All labs should be collected in the morning while participant has fasted for at least 8 hours. Urine collection should be an early morning void, either first or second void, for UACR measurements needed per protocol specified time point.

Table 13: Clinical Laboratory Evaluations

Clinical Chemistry	Hematology	Urine Chemistry
Alkaline phosphatase	Hematocrit [a]	Albumin
ALT	Hemoglobin [a,b]	β2-microglobulin
AST	RBC count & indices	Creatinine and protein
β2-microglobulin	WBC count & differential	Protein & albumin/creatinine ratio
Bilirubin	**Pregnancy**	Microscopic analysis (test stick) [c]
Creatinine kinase	hCG (serum) – confirmatory	Pregnancy (dipstick) [d]
FSH (females only)	**Serology**	**Urine Macro Panel**
GGT	HBV surface antigen	pH
LDH	HCV antibody	Ketones
	HCV RNA PCR - confirmatory [e]	Protein
	HIV antibodies	Blood
		Glucose
**Renal Analytes**	**Coagulation Status**	**Additional Tests**
Albumin	APTT	HbA_{1c}
BUN	PT-INR	iPTH
Calcium	Platelet count	NGAL
CO_2, total		Research samples [f]
Creatinine	**Lipid Panel**	**Drug of Abuse Screen**
Cystatin C	Cholesterol	Amphetamine
CRP	LDL	Barbiturates
eGFR (calculated)	HDL	Benzodiazepines
Glucose	LDL:HDL ratio	Cocaine

Phosphorus<br>Potassium<br>Sodium<br>Chloride<br>Bicarbonate	Triglycerides	Opiates<br>Tetrahydrocannabinol<br>Phencyclidine

Abbreviations: ALT = alanine aminotransferase; APTT = activated partial thromboplastin time; AST = aspartate aminotransferase; BUN = blood urea nitrogen; CO_2 carbon dioxide; CRP = C-reactive protein; eGFR = estimated glomerular filtration rate; FSH = follicle-stimulating hormone; GGT = gamma-glutamyl transferase; HbA_{1c} = glycosylated hemoglobin; HBV = hepatitis B virus; hCG = human chorionic gonadotropin; HCV = hepatitis C virus; HDL = high-density lipoprotein; HIV = human immunodeficiency virus; LDH = lactate dehydrogenase; LDL = low-density lipoprotein; NGAL = neutrophil gelatinase-associated lipocalin; PCR = polymerase chain reaction; iPTH = intact parathyroid hormone; PT-INR = prothrombin time-international normalized ratio; RBC = red blood cell; RNA = ribonucleic acid; WBC = white blood cell.

a. Must be measured locally in addition to centrally on Days 0 for renal biopsy and REACT injection(s).
b. Must be measured locally in addition to centrally within 48 hours before Days 0 for renal biopsy and REACT injection(s). All other measurements will be performed at the central laboratory.
c. Performed locally. If abnormal, microscopic analysis will be performed by the central laboratory.
d. Pregnancy test (dipstick) will be performed locally.
e. To be performed locally at the Investigator's discretion.
f. Refer to the Laboratory Manual for additional details.

7.2.5.1 eGFR

The estimated glomerular filtration rate will be calculated using the 2009 CKD-EPI serum creatinine equation and analyzed by the central laboratory. [80] The specific assay for measuring creatinine will be defined by the Sponsor, and the samples will be analyzed by the central laboratory (aligned with real analyte assessments are shown in the Laboratory Time and Events tables (Table 4, Table 5, and Table 6). For comparison to each subject's historical values, it may be necessary to perform a second analysis at the site laboratory used to generate the historical data. The need for any additional assays conducted locally by the site laboratory will be defined at the Site Initiation Visit.

7.2.5.2 Historical eGFR Measurements

To evaluate the rate of decline in eGFR prior to and after REACT injections, subject's medical records will be reviewed and all sCr and Cystatin C measurements collected within 24 months of the subject signing consent will be documented in the eCRF.

7.2.6 Urine Chemistry

Urine will be collected and analyzed via standard panel. The schedules for collecting each type of urine sample are shown in the Laboratory Time and Events tables (Table 4, Table 5, and Table 6).

Spot urine collections will be used for dipstick urinalysis (test stick) assessments and performed locally. Microscopic analysis will be completed if a test stick is positive and will be conducted by the central laboratory. To provide a comprehensive picture of protein and albumin excretion, both total protein and albumin will be assessed in all samples.

Urine collection should be an early morning void, either first or second void, for UACR measurements needed per protocol specified time point.

7.2.7 Hematology

Hemorrhage following biopsy and REACT injection is a known and foreseeable risk to subjects participating in this study. Therefore, hemoglobin and hematocrit will be measured before and after each procedure at the site's local laboratory. In addition to the local laboratory results hemoglobin and hematocrit will also be measured before and after each procedure by the central laboratory. The local laboratory results are to be used for day of procedure safety evaluations. If hemoglobin is less than or equal to 9 g/dL at these time points, biopsies or injections must not proceed without consulting the Medical Monitor and proceduralist performing the biopsy and REACT injections. Other bleeding parameters (e.g., APTT, PT-INR, platelets) also will be measured throughout the study and depending upon the cohort.

7.2.8 Viral Serology

The biopsy cores obtained from each subject will be used for the expansion and selection of SRC. Contamination with HIV, HBC, and/or hepatitis C virus (HCV) would prevent the Sponsor from manufacturing REACT product for that subject. Therefore, each subject will undergo testing for viral blood-borne pathogens, including HIV antibodies, hepatitis B virus surface antigen, and HCV antibody.

7.2.9 Drugs of Abuse Screen

Consistent with Exclusion Criterion 22, subjects are not eligible to participate in the study if they have "a history of active alcohol and/or drug abuse that, in the judgment of the Investigator, would impair the subject's ability to comply with the protocol." Therefore, subjects will undergo testing for drugs of abuse.

7.2.10 Research Samples

Research samples (serum/plasma and/or urine) will be collected, frozen, and stored for the future research. Participating subjects can opt out of long-term storage of their unused specimens during the informed consent process or at any time during the study.

7.2.11 Pregnancy Screen

A qualitative urine pregnancy test will be performed locally at the site using a dip-strip. If the test is positive, then a confirmatory serum human chorionic gonadotrophin (hCG) test will be performed by the central laboratory. A serum sample may be collected for a pregnancy test where a urine sample is not available. If site practices do not accept the results of a dip-strip, then a urine sample should be sent to the central laboratory for analysis. Post-menopausal women with a confirmatory follicle-stimulating hormone (FSH) test do not have to undergo pregnancy testing throughout the study. To be considered confirmatory, the FSH level must be greater than 30 IU/L.

7.2.12 Renal Imaging

7.2.12.1 Ultrasound

Renal safety ultrasounds will be performed following the in-subject renal biopsy on Day 0 and Day 1 and following the in-subject REACT injection on Day 0, Day 1, Day 7 and optional Day 14 with the aim of monitoring for post-procedure subclinical AEs, primarily renal hematoma formation. If there is a hematoma greater than 2 cm present on Day 0 or Day 1 for REACT injections, then an ultrasound should be conducted at Day 14. An ultrasound may be conducted at other times, in the judgment of the Investigator, if needed for additional safety evaluation(s) and follow-up of acute changes in the kidney. Renal safety ultrasounds will be recorded to include resistance indices, renal size with longitudinal and transverse (medial to lateral and anterior to posterior) dimensions in millimeters and the presence of any post-procedure subclinical AEs as applicable. Findings from the safety ultrasound will be recorded on the appropriate CRF.

7.2.12.2 Computerized Tomography

Non-contrast CT must be used during the REACT injection procedures, according to the usual imaging standards of care at the clinical site. All CTs will be completed non-contrast.

7.2.12.3 Magnetic Resonance Imaging

An MRI study without contrast will be performed during the screening period before randomization to determine kidney size and volume. During the Site Initiation Visit, the MRI process will be defined for each site, depending on the MRI equipment available. A 1.5-T or higher magnetic strength unit is recommended. Open bore scanners are optional if needed. MRI imaging studies with appropriate sequences will be performed to determine kidney volume (for dosing calculations), assess for renal cysts or other masses, and identify adjacent viscera and lungs. MRI will be performed without injection of contrast agent to avoid potential nephrotoxicity. Renal volume measurements will be performed and may be calculated, for example, using a fast 3D gradient-echo sequence, volumetric interpolated breath-hold examination (VIBE), with spatial resolution of $2 \times 1.4 \times 1.2$ mm. Imaging parameters will be recorded in the source documents. For all subjects, MRIs will be completed at the Screening Visit and Month 18/EOS. All indeterminate renal masses identified on the initial screening MRI require further imaging evaluation prior to biopsy procedure at the direction of the Principal Investigator. The use of non-nephrotoxic gadolinium contrast agents for indeterminate mass assessment will be determined by the PI and Imaging Specialist.

If a subject cannot undergo MRI, then CT can be substituted to obtain kidney size and volume.

8 SAFETY ASSESSMENTS AND MANAGEMENT

8.1 Adverse and Serious Adverse Events

8.1.1 Definition of Adverse Events

An AE is a change from baseline medical status at study entry and typified by the development of an untoward medical condition (including abnormal ancillary diagnostic, imaging, physical examination, or laboratory values found clinically significant by the principal investigator) or the deterioration of a pre-existing medical condition following signing of informed consent (regardless of whether deterioration is expected for the natural history of disease state). A pre-existing condition is a clinical condition (including a condition being treated) that is diagnosed before the subject signs the informed consent form (ICF) and prior to randomization. Pre-existing conditions are documented as part of the subject’s medical history within the medical history CRF in the EDC.

The Investigator is responsible for ensuring that all AEs observed by the Investigator or reported by the subject that occur from the day of randomization through at least 18 months after the final

injection of REACT are monitored and recorded in the subject's AE/SAE CRF (as applicable) provided by the Sponsor or its designee.

Treatment-emergent AEs are defined as any AE that started after the first injection of REACT or started prior to the first injection but increased in severity or frequency after the first injection of REACT.

Unscheduled visits may be performed at any time during the study as judged necessary by the Investigator to assess and conduct follow-up on AEs. Evaluations and procedures to be performed at unscheduled visits will be at the Investigator's discretion in consultation with the Sponsor and may be based on those listed in the Time and Events tables.

The Investigator's clinical judgment is used to determine whether a subject is to be removed from treatment due to an AE.

An AE is considered unexpected if it is not consistent in nature or severity with information contained in the Reference Safety Information section of the current version of the Investigator's Brochure provided by the Sponsor.

8.1.1.1 Definition of Serious Adverse Events

A serious adverse event (SAE) will be defined as any AE meeting one or more of the following criteria:

- Results in death
- Is life-threatening
- Requires in-patient hospitalization or prolongation of existing hospitalization that deviates from institutional standard of care or practice
- Results in persistent or significant disability or incapacity
- Results in a congenital abnormality or birth defect
- Is an important medical event that may jeopardize the subject or may require medical intervention to prevent one of the outcomes listed above.

All AEs that occur after randomization through end-of-study, whether or not they are related to study procedures or the investigational product, must be recorded in the subject's AE/SAE CRF (as applicable) provided by the Sponsor or its designee.

8.1.1.2 Other Significant Adverse Events

Significant events of particular clinical importance include SAEs and AEs leading to premature discontinuation of subjects from the study. These events will be recorded in the subjects' medical records as well as the AE/SAE CRF (as applicable) provided by the Sponsor or its designee. Narratives of these events may be prepared for inclusion in the Clinical Study Report.

The following sections describe "AEs of special interest" concerning REACT procedure related events. Subjects should be carefully monitored for the occurrence of these potential AEs.

8.1.1.2.1 Procedure-Related Events

- **Post-procedure pain**: If the subject experiences pain following the biopsy or REACT injection, the management will be determined per site standard of care and based upon clinical assessment. More severe pain in the flank or abdomen requires ultrasonography to exclude significant perirenal hemorrhage. If severe pain occurs, administration of opiates may be necessary. If analgesic doses higher than the maximum authorized doses are required to alleviate pain, then the Investigator must perform additional clinical evaluations to ascertain the probable cause(s) of excessive pain.
- **Post-Procedure Hemorrhage and related AEs**: Following renal biopsy and REACT injection procedures, subjects undergo regular hemoglobin and blood pressure monitoring. Subjects will be confined to bed and monitored for maintenance of normal coagulation indices. If bleeding occurs and the subject is hypotensive despite bed rest, a blood transfusion may be considered. If the bleeding is still not controlled, surgery may be considered. In rare cases, renal angiography may be performed to identify the source of bleeding and vessel embolization can be performed during the same procedure to manage bleeding.
- **Other Post-procedure complications**: In very rare cases, other organs (such as liver, gallbladder, and lungs) may be penetrated during the biopsy procedure. In these cases, appropriate treatment and follow-up may be discussed with consulting surgeons.
- **Post-Procedure Death**: Deaths resulting from renal biopsies occur in <0.01% of subjects. Adherence to strict inclusion/exclusion criteria will ensure that subjects who may be predisposed to uncontrolled or excessive bleeding will not be enrolled in this study.

8.1.1.2.2 Product-Related Events

No REACT product-related events are expected to occur. This assumption is based on the autologous nature of REACT (i.e., REACT is manufactured from renal cells isolated from the same subject to which they are being returned), the lack of product-related events observed in non-clinical animal studies and clinical studies thus far. However, subjects will be extensively monitored throughout the course of the study for any unexpected events.

If an event occurs that is assessed as possibly related or related to the investigational product, REACT, then the event will be immediately reviewed by the Principal Investigator and Medical Monitor.

8.2 Adverse Event Intensity and Relationship Assessment

8.2.1 Intensity Scale

Intensity will be assessed by the Investigator using the US National Cancer Institute (NCI) Common Terminology Criteria for Adverse Events (CTCAE) version 5.0, (refer to https://ctep.cancer.gov/protocoldevelopment/electronic_applications/docs/CTCAE_v5_Quick_Reference_8.5x11.pdf).
If the AE is not included in the CTCAE, then the Investigator will determine the intensity of the AE according to the following criteria:

- **Grade 1:** Mild; asymptomatic or mild symptoms; clinical or diagnostic observations only; intervention not indicated.
- **Grade 2:** Moderate; minimal, local or noninvasive intervention indicated; limiting age-appropriate instrumental Activities of Daily Living (ADL).
- **Grade 3:** Severe or medically significant but not immediately life-threatening; hospitalization or prolongation of hospitalization indicated; disabling; limiting self-care ADL.
- **Grade 4:** Life-threatening consequences; urgent intervention indicated.
- **Grade 5:** Death related to AE.

If the intensity (grade) changes within a day, the maximum intensity (grade) should be recorded. If the intensity (grade) changes over a longer period of time, the changes should be recorded as separate events (having separate onset and stop dates for each grade).

It is important to distinguish between serious and severe AEs. Severity is a measure of intensity whereas seriousness is defined by the criteria under Section 8.1.1.1. Therefore, an AE of severe intensity may not necessarily meet the criteria for seriousness. AEs assessed with an intensity of Grade 4 or above should be reported as serious within the AE/SAE CRF.

8.2.2 Relationship Assessment

An Investigator listed on Form FDA 1572 may make the determination of relationship to the study procedure (treatment) or investigational product for each AE. The Investigator should judge whether there is a reasonable possibility that the AE may have been caused by the study procedure (treatment) or investigational product. If no valid reason exists for suggesting a relationship, then the AE should be classified as "not related." If there is any valid reason, even if undetermined, for suspecting a possible cause and-effect relationship, then the AE should be considered "possibly related" or "related" to the study procedure (treatment) or investigational product.

Definitions of relatedness categories are as follows:

- **Not Related:** Exposure to the study treatment or investigational product did not occur, or the occurrence of the AE is not reasonably related in time, or the AE is considered unlikely to be related to the study treatment or investigational product.
- **Unlikely Related:** Exposure to the study treatment or investigational product and the AE were not closely related in time, and/or the AE could be explained more consistently by causes other than exposure to the study treatment or investigational product.
- **Possibly Related:** Exposure to the study treatment or investigational product and the AE were reasonably related in time, and the AE could be explained equally well by causes other than exposure to the study treatment or investigational product.
- **Related:** Exposure to the study treatment or investigational product and the AE were reasonably related in time, and the AE was more likely explained by exposure to the study treatment or investigational product than by other causes, or the study treatment or investigational product was the most likely cause of the AE.

For the purpose of safety analyses, all AEs judged by the Investigator to be "possibly related" or "related" will be considered treatment-related AEs.

8.3 Recording and Reporting Adverse Events

Adverse events spontaneously reported by the subject and/or reported in response to an open question from study personnel, or revealed by observation, or documented via laboratory reports, imaging reports, consultation notes, survey instruments and other data collection tools, will be recorded in the subject's AE/SAE CRF (as applicable) provided by the Sponsor or its designee.

Adverse events will be reported using standard medical terminology, whenever possible. A clinically significant change in laboratory values or vital signs must be reported as an AE unless the abnormal change constitutes an SAE and/or leads to discontinuation of treatment or withdrawal from the study.

For each AE, the Investigator will record the start date, the stop date, seriousness criteria (as applicable), the intensity (severity) of each reportable event, the Investigator's judgment of the relationship to the study procedure or investigational product, the action taken, and whether the event resulted in discontinuation of treatment or withdrawal from the study. Follow-up information on any SAE may be requested by the Sponsor or its designee.

All SAEs and non-serious AEs of special interest (defined in Section 8.1.1.2) must be promptly reported by the Investigator to the Sponsor, or its designee via EDC reporting, within 24 hours from the time when the Investigator first becomes aware of the event. All SAEs and non-serious AEs of special interest (as applicable) must be reported within the AE/SAE CRF of the EDC. SAEs should be reported whether or not they are considered causally related to the study procedure or investigational product.

The investigator, or designated site personnel must complete the AE/SAE CRF within the EDC. The information collected will include subject number, a narrative description of the event, and an assessment by the Investigator as to the intensity of the event and relatedness to the investigational product or study procedures. The Investigator must complete, sign and date the SAE pages, and verify the accuracy of the information recorded against the corresponding source documents. Follow-up information on the SAE may be requested by the Sponsor or its designee.

Back-up paper SAE report forms (provided by the Sponsor, or its designee to each clinical site) should be used in the event that the EDC down. Once the EDC is available, the site will be responsible for adding the SAE details.

Contact information for reporting SAEs appears below:

**IQVIA 24 Hour Safety Hotline**	Phone: +1 (855) 564-2229<br>Fax: +1 (855) 638-1674<br>Email: *LS_REACT_SO@IQVIA.com*

The Sponsor or its designee is responsible for notifying the FDA and other regulatory authorities of certain AEs. It is the Investigator's responsibility to notify the IRB/EC of all SAEs that occur at the site.

8.3.1 Suspected Unexpected Serious Adverse Reactions

If there are suspected, unexpected, serious adverse reactions (SUSARs) associated with the use of the investigational product, the Sponsor, or its designee, will notify the FDA and other regulatory authorities (as applicable), and all participating Investigators on an expedited basis and required time frames in accordance with applicable regulations. It is the responsibility of the Investigator to promptly notify the IRB/EC and other appropriate institutional regulatory bodies of all suspected unexpected serious adverse reactions involving risk to subjects.

8.3.2 Pregnancy

Pregnancy is neither an AE nor an SAE, unless a complication relating to the pregnancy occurs. All reports of congenital abnormalities/birth defects are SAEs. Spontaneous miscarriages should be reported and handled as SAEs. However, elective abortions without complications should not be handled as AEs.

All pregnancies experienced by female subjects enrolled in this study are to be reported in the same time frame as SAEs using the Pregnancy CRF in the EDC. If the EDC is down for any reason the backup paper pregnancy form should be utilized. Once the EDC is available, the site is responsible for adding the pregnancy details to the Pregnancy CRF. The Investigator should also collect information on the pregnancies of female partners of male subjects enrolled in this study. The course of all pregnancies, including perinatal and neonatal outcome, regardless of whether the subject has discontinued participation in the study, will be followed until resolution, including follow-up of the health status of the newborn to 6 weeks of age.

The effects of administration of the investigational product on the pregnant female or the developing fetus are unknown. Therefore, female subjects of childbearing potential, or male

subjects with female partners of childbearing potential, who are planning a pregnancy during the course of the study, or who are not using a highly effective method(s) of birth control, or who are unwilling to continue using a highly effective method of birth control throughout the duration of the study are not eligible to participate in the study.

8.3.3 Data Monitoring Committee

An independent DMC will be chartered to ensure the safety of subjects enrolled in this study, especially as it relates to unexpected investigational product-related events. The DMC will consist of members who have expertise directly related to protocol-specified activities. It will function independently, and its members will have no other engagement with the Sponsor. The DMC will meet by teleconference at regular intervals, depending on the speed of subject enrollment and the amount of new data generated. The DMC will advise the Sponsor on aspects concerning the safety of subjects participating in the clinical study. Apart from reviewing study data, the DMC will consider feedback from the Sponsor and Investigators. Other specific activities and responsibilities of the DMC will be described in the DMC charter.

8.3.4 Adverse Event Reporting Procedures in Relation to COVID-19 Infection

Subjects with a positive COVID-19 diagnosis should be reported as having an AE in the CRF in accordance with the following guidance:

- Subjects with a positive COVID-19 diagnosis and exhibiting symptoms while recovering at home should be recorded as having an AE.
- Subjects with a positive COVID-19 diagnosis who are asymptomatic should be recorded as having an AE.
- Subjects with a positive COVID-19 diagnosis resulting in hospitalization, care in a triage facility set up by a hospital or resulting in death should be recorded as having an SAE.

8.4 Stopping Rules for an Individual Subject

The Investigator, Medical Monitor, DMC, and Sponsor may remove any subject from the study for the following:

- Any clinical AE, laboratory abnormality, intercurrent illness, or other medical condition or situation whereby continued participation in the study would not be in the best interest of the subject.
- Development of any exclusion criterion prior to REACT injection(s).

If a subject is terminated from the study, EOS assessments should be conducted at the last visit.

If any of the following events occur, no additional subjects can receive REACT injection at the individual site until review by the DMC has been completed:

- Occurrence of bleeding requiring transfusion in greater than 20% of subjects
- Occurrence of major bleeding requiring nephrectomy or other surgical intervention
- Occurrence of death related to the biopsy or REACT injection procedure
- Occurrence of bleeding requiring angiographic interventional embolization in greater than 10% of subjects.

If any of the following events occur, no additional subjects can receive REACT injections at any of the sites until review by the DMC has been completed:

- An SAE that is rated as severe or life-threatening upon final MedWatch report deemed as directly caused by REACT product
- Death of an enrolled subject upon final MedWatch report deemed as directly caused by REACT product
- Similar SAEs in more than one subject at a single site or across multiple sites as observed by the Safety Team, that are caused by REACT.

8.5 Study Suspension or Study Termination

As long as subjects are randomized and participating in the study, including follow-up, the Medical Monitor, DMC, IRB/EC, Sponsor, FDA and other regulatory agencies will review serious, unexpected, procedure-related and product-related AEs.

Enrollment in the study will be suspended if any subject develops an apparent tumor, whether in the kidney or at a remote anatomic site. The Medical Monitor will review and then determine whether or not study amendment or termination is needed. The DMC and Sponsor will review and then determine whether or not study amendment or termination is needed. If a decision is made to suspend or terminate the study, the Sponsor will notify the Investigators/sites as well as the IRB/EC and regulatory authorities, as required.

The DMC, IRB/EC, Sponsor, Investigator(s), FDA, and other regulatory authorities, as part of their duties to ensure human subject protection, may suspend the study at any time due to concerns for the safety of study subjects. Once the clinical study is halted (i.e., no new enrollments and no further REACT injections), a report will be submitted to the IRB/EC.

8.6 Additional Sponsor Specific Reporting Conventions

End stage renal disease (ESRD) should always be reported as a serious adverse event by the Principal Investigator (or designee) and should be reported following a sustained eGFR of less than 15 ml/min/1.73m^2 for a period of 30 days or more.

Upon commencement/initiation of dialysis, dialysis should be reported to the Sponsor (or its designee) via the dialysis CRF within the EDC. Dialysis is a procedure and should not be reported as a SAE, however the rationale for dialysis should always be reported as a SAE (i.e., Dialysis was commenced for fluid overload, fluid overload should be reported as a SAE and dialysis should be recorded on the dialysis CRF).

If dialysis is reported on the dialysis CRF and a corresponding SAE has not been reported:

- The Sponsor (or designee) will urgently query investigative sites to report the corresponding SAE/rationale for initiation of dialysis on the appropriate CRF or paper SAE report form.
- If the corresponding SAE is unknown (rationale for initiation of dialysis), the Sponsor or designee will instruct the investigational site to report dialysis itself as a SAE.

Renal transplantation is a medical/surgical intervention and should not be reported as a SAE, however the rationale for the transplant should always be reported as a SAE (i.e., transplant performed due to end stage renal disease). Details for the renal transplantation should be captured in the EDC system on the transplant CRF.

In general, procedures should not be reported as SAEs, however if the rationale for the procedure is unknown the procedure itself should be reported as default. Investigational sites should make all efforts to acquire necessary information for SAE reporting.

9 STATISTICAL METHODS AND PLANNED ANALYSES

9.1 Sample Size

Approximately 50 subjects will be treated with REACT. As this is a Phase 2 exploratory safety and efficacy study, no formal statistical testing will be performed.

9.2 Criteria for Evaluation

9.2.1 Objective and Endpoints

Section 2 presents the objective and endpoints.

9.2.2 Analysis Conventions

A statistical analysis plan will be prepared and finalized before the final analysis. That document will provide further details regarding the definitions of endpoints and analysis methodology to address the study objective.

All analyses will be descriptive in nature. Continuous variables will be listed, and summarized by treatment using mean, SD, minimum, maximum, and quartiles. Categorical variables will be listed and summarized using frequencies and percentages.

9.2.2.1 Analysis Sets

Intent-to-Treat (ITT): all participants randomized. Participants in the ITT will be analyzed as randomized. The ITT will be used as a sensitivity analysis of all efficacy endpoints if this analysis set is different than the mITT.

Modified Intent-to-Treat (mITT): all subjects randomized who received at least one REACT injection. Subjects in the mITT will be analyzed as randomized. The mITT will be used for the analysis of all efficacy endpoints.

Safety Set (SAF): all subjects randomized who received at least one REACT injection. Subjects in the SAF will be analyzed as treated.

Biopsied Set (BS): all participants who had a biopsy, but did not receive any REACT injections.

9.2.2.2 Subgroup Analyses

The treatments will be compared with respect to safety and efficacy, grouping subjects by baseline measurements:

- CKD stage (3a, 3b, or 4)
- Diabetes mellitus type (1 or 2)
- Albuminuria categories (A2, A3).

Other subgroup analyses may be performed, as will be specified in the statistical analysis plan.

9.2.2.3 Interim Analysis

No interim analysis is planned.

9.2.2.4 Multiplicity

The analyses will not generate p-values. Confidence intervals will not be generated.

9.2.2.5 Missing Data Handling

All analyses will incorporate observed values only, without imputations for missing data.

9.2.2.6 Subject Disposition

Subject disposition (enrolled, randomized cohort, successful biopsy, number of REACT injections received, withdrawn pre-injection and reason, withdrawn post-injection and reason for withdrawal, lost to follow-up, completed study) will be summarized for the ITT.

9.2.2.7 Demographic and Other Baseline Characteristics

Demographic and other baseline characteristics will be summarized for the ITT.

9.2.3 Efficacy Analysis

All efficacy analyses will utilize the mITT. If the ITT analysis set is different than the mITT analysis set then a sensitivity analysis for the efficacy endpoints will be conducted using the ITT.

9.2.3.1 Primary Efficacy Analysis

For the primary efficacy endpoint (the change in rate of renal function progression from pre-injection to post-last injection), will be calculated as change in total slope. The pre-injection and post-last injection annualized slope will be estimated using a class of mixed effects model adjusting for baseline GFR and other covariates, as appropriate.[88] To account for between-participant variability in GFR trajectories, random slopes and intercepts will be included. Furthermore, greater variation in individual GFR measurements at higher GFR may be accounted for using a power of the mean (POM) model. Non-uniform treatment effects in which treatments slowed progression by a greater extent among participants with faster GFR decline than for participants with slower GFR decline may also be accounted for by allowing for

different between- participant slope variances. Further details are included in the statistical analysis plan.

For definitions of endpoints, refer to Section 2.1.1.

9.2.3.2 Secondary Efficacy Analyses

Secondary Analyses of Primary Efficacy Endpoint

Estimated glomerular filtration rates will be calculated in a similar fashion as the primary efficacy endpoint as detailed in Section 9.2.3.1.

Analyses of Secondary and Exploratory Efficacy Endpoints

A Kaplan-Meier plot will be generated for each time-to-event secondary, by treatment, along with estimated quartiles (where estimable), numbers of events, subjects at risk by time point, and censoring time points.

A table will summarize each secondary or exploratory endpoint other than time-to-event endpoints.

5-year risk of ESRD and 2 year risk of ESRD at baseline, 12 and 18 months post-last injection is calculated using the Regionally Calibrated North America 8 variable equation. [89]

9.2.4 Safety Analysis

9.2.4.1 Adverse Events

Adverse events will be coded using the Medical Dictionary for Regulatory Activities (MedDRA), Version 23.0 or later, by System Organ Class (SOC) and Preferred Term (PT). Adverse events will be graded using NCI CTCAE, Version 5.0 or later.

An AE will be considered a treatment-emergent AE (TEAE) if it started or worsened in intensity after the first REACT injection on Day 0 and up to at least 18 months after the last REACT injection.

For summaries of TEAEs, the following TEAE analysis periods will be defined:

- Pre-biopsy Period: from the date of randomization to the start day of biopsy.
- Pre-injection Period: from the start day of biopsy to the first REACT injection,

- Inter-injection Period: from the first REACT injection to either the second REACT injection or (if no such second injection was delivered) study withdrawal or completion.
- Post-second-injection Period: from the second REACT injection to study withdrawal or completion. This period will not apply to subjects for whom only one REACT injection was delivered.

Summaries (frequency counts and percentages) of AEs will be presented by SOC and PT in each of the defined periods will be reported by cohort.

Additional summaries will include, but are not limited to, AEs judged to be related to the procedure (biopsy and/or injection) and/or investigational product, AEs by intensity, AEs leading to discontinuation, SAEs, and deaths.

Adverse events that started during or after the biopsy and before the date of the first REACT injection will be summarized using the ITT. Adverse events that started during or after the biopsy, and before the date of the first REACT injection will be summarized for the ITT and BS. Treatment-emergent summaries will utilize the SAF.

9.2.4.2 Laboratory Evaluations

Observed and change from baseline laboratory data will be summarized by cohort and visit. Laboratory abnormalities will be defined using the NCI CTCAE grading scheme where available, or the laboratory normal ranges for parameters without NCI CTCAE grading. The results of laboratory testing for renal function, specifically sCr, Cystatin C, Blood Urea Nitrogen (BUN), urinary albumin, and eGFR, are of particular interest for this study.

Treatment-emergent laboratory abnormalities will be presented by cohort in shift tables split by same time periods as for AEs (see Section 9.2.4.1).

9.2.4.3 Other Safety Evaluations

Other safety parameters, such as vital signs, and renal ultrasound and ECG evaluations, will be summarized.

Concomitant medications will be summarized by frequency counts and percentages. Other summaries, such as shift tables for the worst treatment-emergent value compared with baseline, may also be produced.

9.2.4.4 Biopsy and REACT Injection(s)

Biopsy and REACT injection data will be listed.

9.2.4.5 Definition of Baseline

Except where indicated, baseline will be defined as the last non missing measurement taken prior to the first REACT injection.

10 ETHICAL AND REGULATORY CONSIDERATIONS

10.1 Good Clinical Practice

This study will be conducted in compliance with the protocol, in accordance with the ICH E6 Harmonized Tripartite Guideline, in general agreement with the most recent version of the Declaration of Helsinki, and in accordance with all applicable US and European regulations.

10.1.1 Delegation of Principal Investigator Responsibilities

The Investigator will ensure that all persons assisting with the study are adequately informed about GCP requirements, the protocol, any amendments to the protocol, and the study treatments as well as their study-related duties and functions. The Investigator will maintain a list providing the names, positions, signatures, and initials of sub-investigators as well as other appropriately qualified personnel, including those authorized to make entries and corrections on the CRF.

10.1.2 Institutional Review Board/Ethics Committee

The final study protocol, Investigator's Brochure, subject ICF, subject recruitment materials (if applicable), and patient-reported questionnaires/surveys (if applicable), including respective version dates, must be approved or given a favorable opinion in writing by an IRB/EC as appropriate. A copy of the written approval must be provided to the Sponsor. This documentation should clearly mention the approval/favorable opinion of the protocol, the subject ICF, and subject recruitment materials (if applicable), and patient-reported questionnaires/surveys (if applicable) along with the respective version dates. The written approval and a list of current membership, or Department of Health and Human Services (DHHS) Assurance Number, or letter from the IRB/EC stating that the membership list is on file, must be provided to the Sponsor prior to the release of clinical study supplies to the investigational site and commencement of the study. If any member of the IRB/EC has direct participation in this clinical study, written notification regarding his or her abstinence from voting must also be obtained.

The Investigator must inform the IRB/EC of any amendment to the protocol, provide updates about the ongoing study at intervals specified by the respective IRB/EC, and submit final study reports to the IRB/EC. In addition, the IRB/IEC must approve all advertising used to recruit subjects for the study along with any written information to be provided to subjects (e.g., diaries, calendars, patient-reported surveys) and updates to the ICF.

It is the Investigator's responsibility to notify the IRB/EC of all SAEs that occur at his or her site. The Sponsor is responsible for notifying the relevant regulatory authorities of certain safety events, including unexpected, serious, drug-related adverse reactions that occur during the clinical study. The Investigator is responsible for notifying its IRB/EC of these unanticipated SAEs.

Initial IRB/EC approval, and all materials approved by the IRB/EC for this study including the subject consent form and recruitment materials must be maintained by the Investigator and made available for inspection.

10.1.2.1 Subject Informed Consent

Informed consent is a process that is initiated prior to the individual's agreeing to participate in the study and continuing throughout the individual's study participation. The Investigator will ensure that potential participants (and their families) are given full and adequate oral and written information about the nature, purpose, possible risk(s), and benefit(s) of the study. Institutional Review Board/Ethics Committee approved consent forms explaining study procedures and the investigational product/intervention(s) will be provided. The Investigator will explain the research study and answer any questions that may arise. Potential participants should have sufficient time to discuss the study, ask questions, and process the information during the consent process before deciding whether or not to participate.

The rights and welfare of potential subjects will be protected by emphasizing that the quality of their medical care will not be adversely affected should they decide not to participate in the research study. Those individuals who agree to enroll must sign and date the current version of the ICF prior to starting any study-related procedures, including screening. Subjects may withdraw consent at any time throughout the course of the clinical study without penalty or prejudice toward future medical care.

The acquisition of informed consent will be documented in the participant's medical records, as required by 21 CFR 312.62. The ICF will be signed and personally dated by the participant and

the person who conducted the informed consent discussion. A copy of the signed ICF must be given to the subject while the original signed ICF must be retained by the Investigator.

10.1.2.2 Subject Confidentiality

Individual subject medical information obtained as a result of this study is considered confidential and disclosure to third parties is prohibited. Such medical information may be given only after approval of the subject to his/her physician or to other appropriate medical personnel responsible for the subject's well-being. The Sponsor affirms the subject's right to protection against invasion of privacy. Only a subject identification number and/or initials (where allowed by local or national regulations) will identify subject data retrieved by the Sponsor. However, the Investigator is required to allow the Sponsor, its designated representative(s), the IRB/EC, and when necessary, representatives of the regulatory health authorities to review and/or to copy any medical records relevant to the study.

10.1.2.3 Substantial Amendment to the Protocol

A substantial amendment to the protocol must be agreed upon in writing by the Sponsor, then submitted to and approved by the respective regulatory authority before the amendment can be implemented. It is the responsibility of the Sponsor or its designee to ensure compliance with the appropriate regulatory requirements.

Written approval of a protocol amendment is not required prior to implementation of changes that eliminate an immediate hazard to the study subject; however, approval must be obtained as soon as possible thereafter. Any protocol amendment also must be signed by the Investigator, who will provide a copy to the IRB/EC.

10.1.2.4 Protocol Deviations

A protocol deviation is any noncompliance with the protocol or GCP requirements. The noncompliance may be attributed to the subject, the Investigator, or the site staff. All protocol deviations will be documented and reported by the monitor during the course of the study as described in the Clinical Operations Plan. The CRO will work with the site to develop and implement any corrective actions to address protocol deviations, as appropriate.

Note: serious breaches of the protocol that are likely to significantly affect the safety of a subject or the integrity of the data generated must be reported to the Sponsor, IRB/EC, and regulatory authorities.

Although, in principle, no deviations from or changes to the protocol are permitted, under emergency circumstances protocol deviations may proceed without prior approval from the Sponsor, IRB/EC and regulatory authorities to protect the rights, safety, and well-being of study subjects.

11 DATA HANDLING AND RECORDKEEPING

11.1 Data Collection and Review

11.1.1 Data Collection

The Sponsor or its designee will provide the clinical sites with access to CRFs for each subject. A CRF must be completed for every subject who provides written, informed consent and has undergone at least one protocol-specified, study-specific assessment. The CRF completion instructions will be provided to the site. The Investigator or designated representative should complete the CRFs as soon as possible after the information has been collected. The Investigator is responsible for ensuring the accuracy, completeness, and timeliness of the data reported in a subject’s CRF. Source documentation supporting the CRF data should indicate the subject’s participation in the study and document the dates and details of study procedures, AEs, and subject status.

When a subject completes the study, the Investigator must review and sign the CRF indicating that he/she has reviewed the completed CRF and pertinent clinical data for that subject and that, to the best of his/her knowledge, all data recorded in the CRF accurately reflect the subject’s clinical performance in the study.

All clinical study information is recorded, processed, handled, and stored by the sponsor and or designated vendors, in such a way that it can be accurately reported, interpreted, and verified while the confidentiality of records and the personal data of the subjects remain protected in accordance with the applicable law on personal data protection. Appropriate technical and organizational measures have been implemented to protect information and personal data processed against unauthorized or unlawful access, disclosure, dissemination, alteration, or destruction or accidental loss. Data collection on participants does include basic demographic information such as age, gender, ethnicity and existing comorbidities for the purposes identification of any imbalance between cohorts after randomization as well as for monitoring for any desirable or undesirable product effects within sub-groups of the study population.

11.1.2 Study Monitoring

Before an investigational site can enter a subject into the study, a representative of the Sponsor or its designee will visit the investigational study site to do the following:

- Determine the adequacy of the facilities.
- Discuss with the Investigator(s) and other personnel their responsibilities under the protocol as well as the responsibilities of the Sponsor. This will be documented in a Clinical Trial Agreement between the Sponsor and the Investigator.

During the study, a study monitor appointed by the Sponsor or its designee will have regular contact with the site and conduct on-site and/or remote monitoring visits as described in the Study Monitoring Plan. Monitoring and auditing procedures, approved by the Sponsor, will ensure that the study is conducted in accordance with the protocol and regulatory requirements while ensuring the safety of all study subjects. Apart from on-site visits, the monitor will maintain frequent communication with the Investigator and site personnel via letters, e-mails, telephone, and fax to address actions needed by site personnel.

Duties of the monitor include, but are not limited to the following:

- On-site review of the CRFs for completeness and clarity
- Confirmation that the facilities remain acceptable
- Confirmation that investigational product accountability checks are being performed
- Confirmation that AEs and SAEs are properly documented in the CRFs, and that any SAEs have been forwarded to the Sponsor or its designee and the IRB/EC
- Recording and reporting of any protocol deviations not previously sent to the Sponsor, the IRB/EC, and regulatory authorities, as required
- Clarification of administrative matters.

The monitor will perform source data verification, including a comparison of the data in the CRF with the subject's medical records at the hospital or clinic, and other records relevant to the study. Source data verification requires direct access to all original records for each subject. The review of medical records will be performed in a manner that ensures subject confidentiality.

Regulatory authorities and the IRB/EC may request access to all source documents for on-site inspection or an audit. Likewise, the Sponsor or its designee may conduct a quality assurance (QA) audit to ensure compliance with GCP and all applicable regulatory requirements. Direct

access to source documents must be guaranteed by the Investigator, who will provide support at all times for these activities.

11.1.2.1 Audits and Inspections

The purpose of an audit or inspection by the Sponsor, regulatory authorities, the IRB/EC, or other appropriate institutional bodies is to systematically and independently examine all study-related activities and documents to determine whether these activities were conducted, and the data were recorded, analyzed, and accurately reported, in accordance with the protocol, ICH-GCP, and any applicable regulatory requirements. The Investigator and the site/institution must provide support at all times for on-site audits or inspections by providing direct access to all source documents, CRFs, and other study documentation. The Investigator must notify the Sponsor or its designee immediately if contacted by a regulatory agency about an inspection.

In addition, the Sponsor or its designee will be allowed to conduct site visits to the investigational facilities for the purpose of monitoring any aspect of the study. The Investigator agrees to allow the monitor to inspect the drug storage area, drug accountability records, subject charts, study source documents, and other records relative to study conduct.

11.1.2.2 Retention of Records

The Investigator agrees to keep records and those documents that include (but are not limited to) the following: the identification of all consented subjects; medical records; study-specific source documents; source worksheets; all original signed and dated ICFs; records of any body fluids or tissue samples retained; query responses; and detailed records of investigational product accountability to enable evaluations or audits from regulatory authorities, the Sponsor, or its designees.

These documents are to be retained until at least 2 years after the last approval of a marketing application in an ICH region and until there are no pending or contemplated marketing applications in an ICH region, or at least 2 years have elapsed since the formal discontinuation of clinical development of the investigational therapy, or a longer document retention timeframe according to country-specific requirements and investigator agrees to keep these records as applicable. If the Investigator cannot meet this obligation, he/she must ask the Sponsor for permission to make alternative arrangements; details of these arrangements should be documented. If the Investigator withdraws from the responsibility of retaining the study records,

custody must be transferred to a person willing to accept the responsibility. The Sponsor must be notified in writing if a custodial change occurs.

12 QUALITY CONTROL AND QUALITY ASSURANCE

Independent Quality Assurance (QA) and clinical Quality Control (QC) systems are implemented and maintained using written SOPs to ensure that the study is conducted and data are generated, documented (recorded), and reported in compliance with the protocol, ICH-GCP, and the applicable regulatory requirements.

To ensure compliance with GCP and all applicable regulatory requirements, the Sponsor may conduct a quality assurance audit. See Section 11.1.2.1 for more details regarding the audit process.

13 PUBLICATION POLICY

All information regarding the investigational therapy is the confidential property of the Sponsor. The Investigator agrees to use this information to conduct the study and will not use it for other purposes without written approval from the Sponsor. It is understood that there is an obligation to provide the Sponsor with complete data obtained during the study. The Sponsor retains full rights over any invention, discovery, or innovation, patentable or not, that may occur during the conduct of the clinical study.

It is anticipated that the results of this study will be presented at scientific meeting(s) and/or published in a peer-reviewed journal(s). A Publications Committee, composed of Investigators participating in the study and representatives from the Sponsor, will oversee the publication and presentation of study results, which will reflect the experience of all participating clinical sites.

The International Committee of Medical Journal Editors has adopted a trials-registration policy requiring that all clinical studies be registered in a public database (such as ClinicalTrials.gov and clinicaltrialsregister.eu) as a condition for publication in member journals. It is the responsibility of the Sponsor to register this study in an acceptable clinical trial registry on or before the onset of subject enrollment.

14 LIST OF REFERENCES

1. Magee C, Grieve DJ, Watson CJ, Brazil DP. Diabetic nephropathy: a tangled web to unweave. Cardiovasc Drugs Ther 2017;31(5-6):579-92.
2. Cavanaugh KL. Diabetes management issues for patients with chronic kidney disease. Clinical Diabetes 2007;25(3):90-7.
3. Kramer A, Pippias M, Noordzij M, et al. The European Renal Association - European Dialysis and Transplant Association (ERA-EDTA) Registry Annual Report 2016: a summary. Clin Kidney J 2019;12(5):702-20.
4. Lu Y, Stamm C, Nobre D, et al. Changing trends in end-stage renal disease patients with diabetes. Swiss Med Wkly 2017;147:w14458.
5. Postma MJ, de Zeeuw D. The economic benefits of preventing end-stage renal disease in patients with type 2 diabetes mellitus. Nephrol Dial Transplant 2009;24(10):2975-83.
6. ERA. 2009 European Renal Association, European Dialysis and Transplant Association. ERA-EDTA Registry 2009.
7. Lees JS, Welsh CE, Celis-Morales CA, et al. Glomerular filtration rate by differing measures, albuminuria and prediction of cardiovascular disease, mortality and end-stage kidney disease. Nat Med 2019;25(11):1753-60.
8. United States Renal Data System. Chapter 3: morbidity and mortality in patients With CKD. Am J Kidney Dis 2017;69(3):S67-S92.
9. Saran R, Robinson B, Abbott KC, et al. US Renal Data System 2018 Annual Data Report: epidemiology of kidney eisease in the United States. Am J Kidney Dis 2019;73(3 Suppl 1):A7-A8.
10. Keith DS, Nichols GA, Gullion CM, Brown JB, Smith DH. Longitudinal follow-up and outcomes among a population with chronic kidney disease in a large managed care organization. Arch Intern Med 2004;164(6):659-63.
11. Rahman M, Pressel S, Davis BR, et al. Cardiovascular outcomes in high-risk hypertensive patients stratified by baseline glomerular filtration rate. Ann Intern Med 2006;144(3):172-80.
12. Hill NR, Fatoba ST, Oke JL, et al. Global prevalence of chronic kidney disease - a systematic review and meta-analysis. PLoS One 2016;11(7):e0158765.
13. Jha V, Garcia-Garcia G, Iseki K, et al. Chronic kidney disease: global dimension and perspectives. Lancet 2013;382(9888):260-72.
14. Khan SS, Kazmi WH, Abichandani R, Tighiouart H, Pereira BJ, Kausz AT. Health care utilization among patients with chronic kidney disease. Kidney Int 2002;62(1):229-36.

15. Dalrymple LS, Katz R, Kestenbaum B, et al. Chronic kidney disease and the risk of end-stage renal disease versus death. J Gen Intern Med 2011;26(4):379-85.
16. Kumar S, Bogle R, Banerjee D. Why do young people with chronic kidney disease die early? World J Nephrol 2014;3(4):143-55.
17. Lee GH, Benner D, Regidor DL, Kalantar-Zadeh K. Impact of kidney bone disease and its management on survival of patients on dialysis. J Ren Nutr 2007;17(1):38-44.
18. Gerstein HC, Mann JF, Yi Q, et al. Albuminuria and risk of cardiovascular events, death, and heart failure in diabetic and nondiabetic individuals. JAMA 2001;286(4):421-6.
19. Stenvinkel P. Chronic kidney disease: a public health priority and harbinger of premature cardiovascular disease. J Intern Med 2010;268(5):456-67.
20. Levin A, Stevens PE, Bilous RW, et al. Kidney Disease: Improving Global Outcomes (KDIGO) CKD Work Group. KDIGO 2012 clinical practice guideline for the evaluation and management of chronic kidney disease. Kidney Int Suppl 2013;3(1):1-150.
21. Dhingra RK, Young EW, Hulbert-Shearon TE, Leavey SF, Port FK. Type of vascular access and mortality in U.S. hemodialysis patients. Kidney Int 2001;60(4):1443-51.
22. Stevens PE, Levin A, Kidney Disease: Improving Global Outcomes Chronic Kidney Disease Guideline Development Work Group M. Evaluation and management of chronic kidney disease: synopsis of the kidney disease: improving global outcomes 2012 clinical practice guideline. Ann Intern Med 2013;158(11):825-30.
23. Pleniceanu O, Harari-Steinberg O, Omer D, et al. Successful introduction of human renovascular units into the mammalian kidney. J Am Soc Nephrol 2020;31(12):2757-72.
24. Romagnani P, Remuzzi G. Renal progenitors in non-diabetic and diabetic nephropathies. Trends Endocrinol Metab 2013;24(1):13-20.
25. Sagrinati C, Netti GS, Mazzinghi B, et al. Isolation and characterization of multipotent progenitor cells from the Bowman's capsule of adult human kidneys. J Am Soc Nephrol 2006;17(9):2443-56.
26. Lin F, Moran A, Igarashi P. Intrarenal cells, not bone marrow-derived cells, are the major source for regeneration in postischemic kidney. J Clin Invest 2005;115(7):1756-64.
27. Humphreys BD, Czerniak S, DiRocco DP, Hasnain W, Cheema R, Bonventre JV. Repair of injured proximal tubule does not involve specialized progenitors. Proc Natl Acad Sci U S A 2011;108(22):9226-31.
28. Humphreys BD, Valerius MT, Kobayashi A, et al. Intrinsic epithelial cells repair the kidney after injury. Cell Stem Cell 2008;2(3):284-91.
29. Kelley R, Bruce A, Spencer T, et al. A population of selected renal cells augments renal function and extends survival in the ZSF1 model of progressive diabetic nephropathy. Cell transplantation 2013;22(6):1023-39.

30. Kelley R, Bruce A, Wallace S, et al. Enhanced renal cell function in dynamic 3D culture systems. KIDSTEM International Conference. Liverpool, England. September 17-19, 2008.
31. Kelley R, Spencer T, Werdin E, et al. Intra-renal transplantation of bioactive renal cells preserves renal functions and extends survival in the ZSF1 model of progressive diabetic nephropathy. 71st scientific sessions of American Diabetes Association. San Diego, CA. June 24-28, 2011.
32. Kelley R, Werdin ES, Bruce AT, et al. Tubular cell-enriched subpopulation of primary renal cells improves survival and augments kidney function in rodent model of chronic kidney disease. Am J Physiol Renal Physiol 2010;299(5):F1026-39.
33. Yamaleyeva LM, Guimaraes-Souza NK, Krane LS, et al. Cell therapy with human renal cell cultures containing erythropoietin-positive cells improves chronic kidney injury. Stem Cells Transl Med 2012;1(5):373-83.
34. United States Renal Data System. National Institute of Diabetes and Digestive and Kidney Diseases. US Renal Data System 2012 Annual Data Report: atlas of chronic kidney disease and end-stage renal disease in the United States. Bethesda: MD: National Institutes of Health 2011.
35. Costacou T, Orchard TJ. Cumulative kidney complication risk by 50 years of type 1 diabetes: the effects of sex, age, and calendar year at onset. Diabetes Care 2017:dc171118.
36. Perkins BA, Bebu I, de Boer IH, et al. Risk factors for kidney disease in type 1 diabetes. Diabetes Care 2019;42(5):883-90.
37. DiMeglio LA, Evans-Molina C, Oram RA. Type 1 diabetes. Lancet 2018;391(10138):2449-62.
38. Bakker J, Olree M, Kaatee R, et al. Renal volume measurements: accuracy and repeatability of US compared with that of MR imaging. Radiology 1999;211(3):623-8.
39. Huang CW, Chou KJ, Fang HC, et al. Unusual cause of postrenal biopsy anuria in a renal transplant patient. Am J Med Sci 2011;341(3):250-2.
40. Corapi KM, Chen JL, Balk EM, Gordon CE. Bleeding complications of native kidney biopsy: a systematic review and meta-analysis. Am J Kidney Dis 2012;60(1):62-73.
41. Palsson R, Short SAP, Kibbelaar ZA, et al. Bleeding complications after percutaneous native kidney biopsy: results from the Boston Kidney Biopsy Cohort. Kidney Int Rep 2020;5(4):511-8.
42. Poggio ED, McClelland RL, Blank KN, et al. Systematic review and meta-analysis of native kidney biopsy complications. Clin J Am Soc Nephrol 2020;15(11):1595-602.

43. Halimi JM, Gatault P, Longuet H, et al. Major bleeding and risk of death after percutaneous native kidney biopsies: a French nationwide cohort study. Clin J Am Soc Nephrol 2020;15(11):1587-94.
44. Al Turk AA, Estiverne C, Agrawal PR, Michaud JM. Trends and outcomes of the use of percutaneous native kidney biopsy in the United States: 5-year data analysis of the Nationwide Inpatient Sample. Clin Kidney J 2018;11(3):330-6.
45. Korbet SM, Volpini KC, Whittier WL. Percutaneous renal biopsy of native kidneys: a single-center experience of 1,055 biopsies. Am J Nephrol 2014;39(2):153-62.
46. Whittier WL, Korbet SM. Timing of complications in percutaneous renal biopsy. J Am Soc Nephrol 2004;15(1):142-7.
47. Society of Interventional Radiology Practice Parameter for the Performance of Percutaneous Nephrostomy (ACR-SIR-SPIR). 2016. https://www.acr.org/-/media/ACR/Files/Practice-Parameters/percutaneous-nephros.pdf.
48. MacGinley R, Champion De Crespigny PJ, Gutman T, et al. KHA-CARI Guideline recommendations for renal biopsy. Nephrology (Carlton) 2019;24(12):1205-13.
49. Patel IJ, Rahim S, Davidson JC, et al. Society of Interventional Radiology consensus guidelines for the periprocedural management of thrombotic and bleeding risk in patients undergoing percutaneous image-guided interventions-part II: recommendations endorsed by the Canadian Association for Interventional Radiology and the Cardiovascular and Interventional Radiological Society of Europe. J Vasc Interv Radiol 2019;30(8):1168-84.
50. Gilbert T. Guidelines for the management of patients on oral anticoagulation and antiplatelet therapy undergoing percutaneous image-guided needle procedures: 2018 revision. https://3dnv4u2b0e1l3hxjzp4dxoh3-wpengine.netdna-ssl.com/wp-content/uploads/2019/02/2018-Quality-Institute-Anticoagulant_drug_guideline-12_13_18-2.pdf. Accessed March 15, 2021.
51. Hogan JJ, Mocanu M, Berns JS. The native kidney biopsy: update and evidence for best practice. Clin J Am Soc Nephrol 2016;11(2):354-62.
52. Luciano RL, Moeckel GW. Update on the native kidney biopsy: core curriculum 2019. Am J Kidney Dis 2019;73(3):404-15.
53. Oberholzer M, Torhorst J, Perret E, Mihatsch MJ. Minimum sample size of kidney biopsies for semiquantitative and quantitative evaluation. Nephron 1983;34(3):192-5.
54. Groman RP, Bahr A, Berridge BR, Lees GE. Effects of serial ultrasound-guided renal biopsies on kidneys of healthy adolescent dogs. Vet Radiol Ultrasound 2004;45(1):62-9.
55. Manno C, Bonifati C, Torres DD, Campobasso N, Schena FP. Desmopressin acetate in percutaneous ultrasound-guided kidney biopsy: a randomized controlled trial. Am J Kidney Dis 2011;57(6):850-5.

56. Khajehdehi P, Junaid SM, Salinas-Madrigal L, Schmitz PG, Bastani B. Percutaneous renal biopsy in the 1990s: safety, value, and implications for early hospital discharge. Am J Kidney Dis 1999;34(1):92-7.
57. Walker PD. The renal biopsy. Arch Pathol Lab Med 2009;133(2):181-8.
58. Arora K, Punia RS, D'Cruz S. Comparison of diagnostic quality of kidney biopsy obtained using 16G and 18G needles in patients with diffuse renal disease. Saudi J Kidney Dis Transpl 2012;23(1):88-92.
59. Ham YR, Moon KR, Bae HJ, et al. A case of urine leakage: an unusual complication after renal biopsy. Chonnam Med J 2011;47(3):181-4.
60. Remer EM, Papanicolaou N, Casalino DD, et al. ACR Appropriateness Criteria® on renal failure. The American Journal of Medicine 2014;127(11):1041-8.e1.
61. Lees GE, Cianciolo RE, Clubb FJ, Jr. Renal biopsy and pathologic evaluation of glomerular disease. Top Companion Anim Med 2011;26(3):143-53.
62. Kollerup Madsen B, Hilscher M, Zetner D, Rosenberg J. Adverse reactions of dimethyl sulfoxide in humans: a systematic review. F1000Res 2018;7:1746.
63. Cox MA, Kastrup J, Hrubisko M. Historical perspectives and the future of adverse reactions associated with haemopoietic stem cells cryopreserved with dimethyl sulfoxide. Cell Tissue Bank 2012;13(2):203-15.
64. Zinderman CE, Landow L, Wise RP. Anaphylactoid reactions to Dextran 40 and 70: reports to the United States Food and Drug Administration, 1969 to 2004. J Vasc Surg 2006;43(5):1004-9.
65. Gunn AJ, Parikh NS, Bhatia S. Society of Interventional Radiology quality improvement standards on percutaneous ablation in renal cell carcinoma. J Vasc Interv Radiol 2020;31(2):195-201 e3.
66. Salagierski M, Salagierski MS. Radiofrequency ablation: a minimally invasive approach in kidney tumor management. Cancers (Basel) 2010;2(4):1895-900.
67. Bakdash K, Schramm KM, Annam A, Brown M, Kondo K, Lindquist JD. Complications of percutaneous renal biopsy. Semin Intervent Radiol 2019;36(2):97-103.
68. Davidson JC, Rahim S, Hanks SE, et al. Society of Interventional Radiology consensus guidelines for the periprocedural management of thrombotic and bleeding risk in patients undergoing percutaneous image-guided interventions-part I: review of anticoagulation agents and clinical considerations: endorsed by the Canadian Association for Interventional Radiology and the Cardiovascular and Interventional Radiological Society of Europe. J Vasc Interv Radiol 2019;30(8):1155-67.

69. Estcourt LJ, Malouf R, Doree C, Trivella M, Hopewell S, Birchall J. Prophylactic platelet transfusions prior to surgery for people with a low platelet count. Cochrane Database Syst Rev 2018;9:CD012779.
70. Glance LG, Blumberg N, Eaton MP, et al. Preoperative thrombocytopenia and postoperative outcomes after noncardiac surgery. Anesthesiology 2014;120(1):62-75.
71. Eiro M, Katoh T, Watanabe T. Risk factors for bleeding complications in percutaneous renal biopsy. Clin Exp Nephrol 2005;9(1):40-5.
72. Cain H, Egner E, Redenbacher M. Increase of mitosis in the tubular epithelium following intrarenal doses of various kidney homogenates and hemogenate fractions in the rat. Virchows Arch B Cell Pathol 1976;22(1):55-72.
73. Montgomery AV, Davis JC, Jr., Prine JM, Swann HG. The intrarenal pressure; its relation to age, weight, blood pressure, and sex. J Exp Med 1950;92(6):637-42.
74. Swann HG, Prine JM, Moore V, Rice RD. The intrarenal pressure during experimental renal hypertension. J Exp Med 1952;95(5):281-91.
75. Brenner BM. Nephron adaptation to renal injury or ablation. Am J Physiol 1985;249(3 Pt 2):F324-37.
76. Field JM, Hazinski MF, Sayre MR, et al. Part 1: executive summary: 2010 American Heart Association guidelines for cardiopulmonary resuscitation and emergency cardiovascular care. Circulation 2010;122(18 Suppl 3):S640-56.
77. Wallace S, Bruce A, Choudhury S, et al. Quantitative ex-vivo characterization of human renal cell population dynamics via high-content image-based analysis (HCA). ISCT Annual Meeting. Philadelphia, PA. May 26, 2010.
78. Kelley R, Werdin E, Bruce A, et al. Bioactive renal cells augment renal function in the ZSF-1 model of diabetic nephropathy. TERMIS America Meeting. Orlando, FL. December 5-8, 2010.
79. Presnell SC, Bruce AT, Wallace SM, et al. Isolation, characterization, and expansion methods for defined primary renal cell populations from rodent, canine, and human normal and diseased kidneys. Tissue Eng Part C Methods 2011;17(3):261-73.
80. Levey A, Stevens L. Estimating GFR Using the CKD Epidemiology Collaboration(CKD-EPI) Creatinine Equation: More Accurate GFR Estimates, Lower CKD Prevalence Estimates, and Better Risk Predictions. Am J Kidney Disease. 2010 April; 55(4): 622-627.
81. Atala A, Bauer SB, Soker S, Yoo JJ, Retik AB. Tissue-engineered autologous bladders for patients needing cystoplasty. Lancet 2006;367(9518):1241-6.

82. Bruce AT, Ilagan RM, Guthrie KI, et al. Selected renal cells modulate disease progression in rodent models of chronic kidney disease via NF-kappaB and TGF-beta1 pathways. Regen Med 2015;10(7):815-39.
83. Jayo MJ, Jain D, Ludlow JW, et al. Long-term durability, tissue regeneration and neo-organ growth during skeletal maturation with a neo-bladder augmentation construct. Regen Med 2008;3(5):671-82.
84. Jayo MJ, Jain D, Wagner BJ, Bertram TA. Early cellular and stromal responses in regeneration versus repair of a mammalian bladder using autologous cell and biodegradable scaffold technologies. J Urol 2008;180(1):392-7.
85. Jayo MJ, Watson DD, Wagner BJ, Bertram TA. Tissue engineering and regenerative medicine: role of toxicologic pathologists for an emerging medical technology. Toxicol Pathol 2008;36(1):92-6.
86. Bruce A, Cox B, Watts B, Wallace S, Presnell S. Hypoxic exposure of cultured human renal cells induces mediators of cell migration and attachment and facilitates the repair of tubular cell monolayers in vitro. FASEB J 2011;25(S1):121.6-.6.
87. Levey A, Stevens L. Estimating GFR Using the CKD Epidemiology Collaboration (CKD-EPI) Creatinine Equation: More Accurate GFR Estimates, Lower CKD Prevalence Estimates, and Better Risk Predictions. Am J Kidney Disease. 2010 April; 55(4): 622-627.
88. Vonesh E, Tighiouart H, Ying J, et al. Mixed effects models for slope-based endpoints in clinical trials for chronic kidney disease. Stat Med. 2019 September; 38(22): 4218-4239.
89. Tangri N, Grams ME, Levey AS, et al. Multinational Assessment of Accuracy of Equations for Predicting Risk of Kidney Failure: A Meta-analysis. JAMA. 2016; 315(2):164–174. doi:10.1001/jama.2015.18202.
